# Direct synthesis of aryl-annulated [*c*]carbazoles by gold(i)-catalysed cascade reaction of azide-diynes and arenes†
†Electronic supplementary information (ESI) available: Experimental details and spectral data are provided. CCDC 1860899 and 1860900 (**6aF-Me_2_**), 1860901 and 1860902 (**7aF-Me_2_**), and 1860904 and 1860905 (**11A-Me_2_**). For ESI and crystallographic data in CIF or other electronic format see DOI: 10.1039/c8sc03525c


**DOI:** 10.1039/c8sc03525c

**Published:** 2018-09-10

**Authors:** Yuiki Kawada, Shunsuke Ohmura, Misaki Kobayashi, Wataru Nojo, Masaki Kondo, Yuka Matsuda, Junpei Matsuoka, Shinsuke Inuki, Shinya Oishi, Chao Wang, Tatsuo Saito, Masanobu Uchiyama, Takanori Suzuki, Hiroaki Ohno

**Affiliations:** a Graduate School of Pharmaceutical Sciences , Kyoto University , Sakyo-ku , Kyoto 606-8501 , Japan . Email: hohno@pharm.kyoto-u.ac.jp; b Department of Chemistry , Faculty of Science , Hokkaido University , Sapporo 060-0810 , Japan; c Graduate School of Pharmaceutical Sciences , The University of Tokyo , 7-3-1 Hongo, Bunkyo-ku , Tokyo 113-0033 , Japan; d Cluster of Pioneering Research (CPR) , Advanced Elements Chemistry Laboratory , RIKEN, 2-1 Hirosawa , Wako , Saitama 351-0198 , Japan

## Abstract

The gold-catalysed annulation of conjugated alkynes bearing an azido group with arenes gave annulated [*c*]carbazoles. Using benzene, pyrrole, and indole derivatives as the nucleophiles, benzo[*c*]-, pyrrolo[2,3-*c*]-, and indolo[2,3-*c*]carbazoles were produced, respectively. The reaction proceeded through pyrrole and benzene ring construction accompanied by the formation of two carbon–carbon and one carbon–nitrogen bond and the cleavage of two aromatic C–H bonds. The mechanism of the reaction with pyrrole was investigated by density functional theory calculations. *N*,*N*′-dimethylated indolo[2,3-*c*]carbazole showed dual ultraviolet-visible-near-infrared and fluorescence spectral changes upon electrolysis.

## Abstract 2

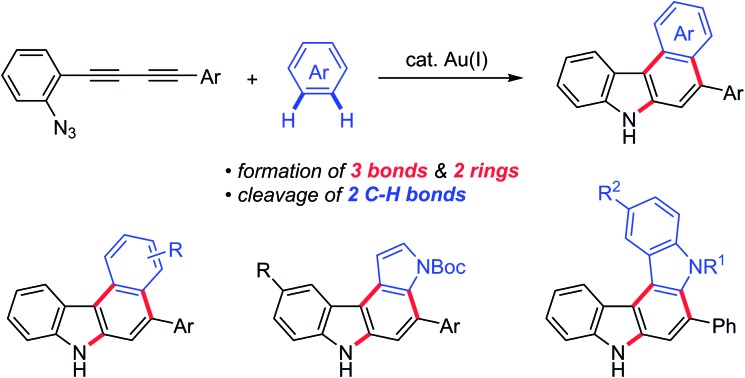
The gold-catalysed annulation of conjugated alkynes bearing an azido group with arenes gave annulated [*c*]carbazoles.

## Introduction

**Fig. 1:**
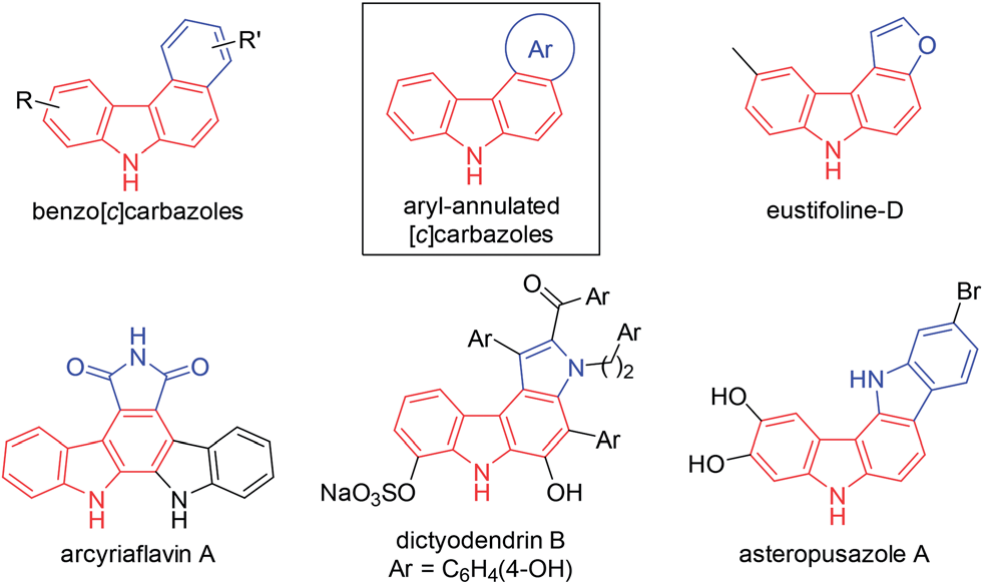
Aryl-annulated [*c*]carbazoles.

Carbazoles are important structural motifs that are found in a variety of organic molecules of current interest (Fig. 1).^1^ Benzo[*c*]carbazoles are commonly used in organic light-emitting diodes (OLEDs) owing to their charge-transport properties and thermal stability.^2^ Heteroaryl-annulated [*c*]carbazoles are the core structures of various bioactive natural products, such as eustifoline-D (furo[2,3-*c*]carbazole), arcyriaflavin A and dictyodendrins (pyrrolo[*c*]carbazole), and asteropusazole A (indolo[3,2-*c*]carbazole).1*a* Thus, the development of efficient synthetic methods for preparing benzo[*c*]carbazoles and their heteroaromatic congeners from readily accessible starting materials is an active pursuit in organic chemistry.

**Scheme 1:**
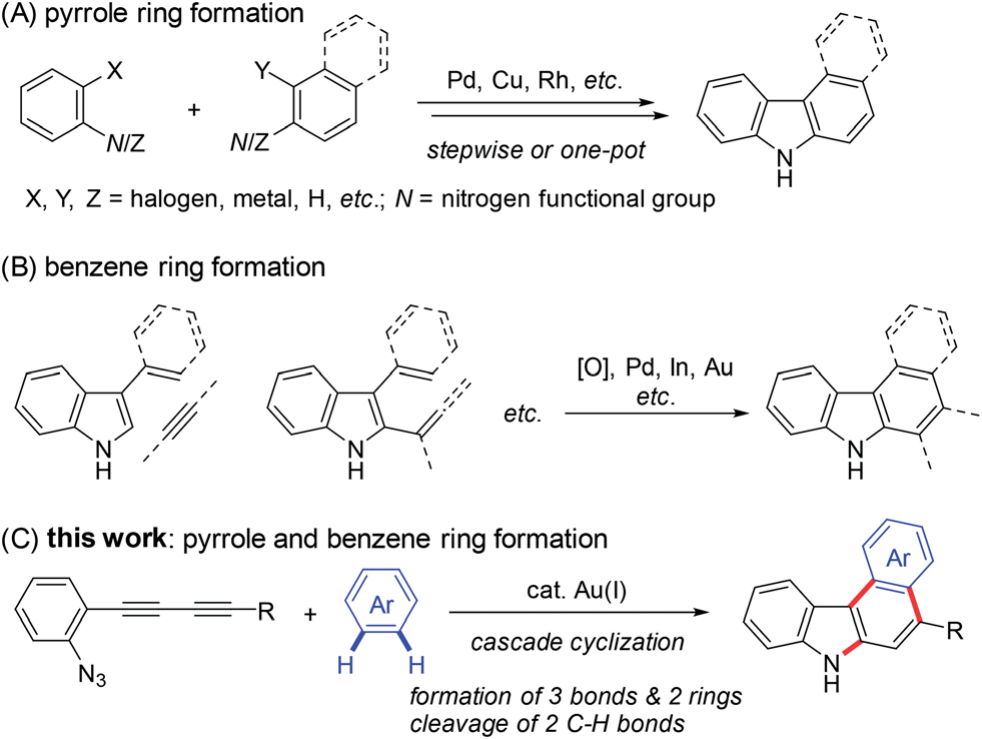
General synthetic approaches to carbazoles including aryl-annulated carbazoles and this work.

The general synthetic approaches to carbazoles including aryl-annulated [*c*]carbazoles are shown in Scheme 1A and B.1a,^3^ Pyrrole ring formation based on a combination of carbon–nitrogen and carbon–carbon bond formation provides an efficient route to carbazole synthesis (Scheme 1A).^4^,^5^ Reliable coupling reactions such as the Suzuki–Miyaura, Buchwald, and oxidative coupling reactions can be employed for this purpose. Benzene ring formation using vinyl- or aryl-substituted indoles including Diels–Alder-type reactions,^6^ hydroarylation,^7^ and related reactions^8^ leads to various carbazoles including aryl-annulated carbazoles (Scheme 1B). However, the double cyclisation approach for synthesising aryl-annulated [*c*]carbazoles has not been investigated until recently.^9^–^11^ We expected that the gold carbenoid-based cascade cyclisation of conjugated diynes would directly provide aryl-annulated [*c*]carbazoles in a single operation *via* the sequential cleavage of two aromatic C–H bonds (Scheme 1C).

**Scheme 2:**
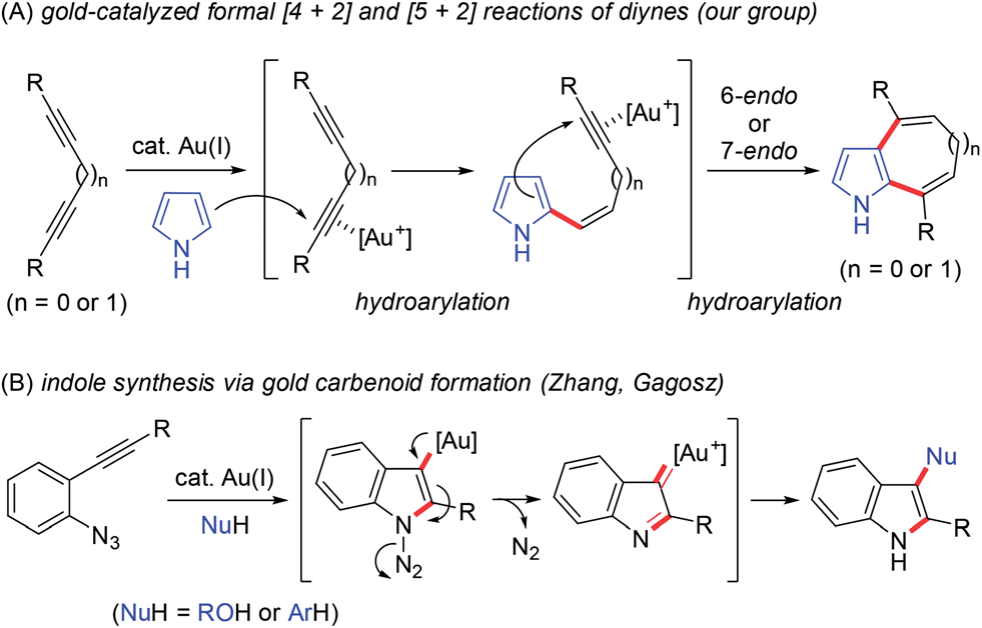
Related studies.

Homogenous gold catalysis has emerged as a powerful tool for atom-economical transformations.^12^ The π-acidity of gold catalysts enables the activation of C–C multiple bonds to promote various transformations. Recent investigations using diynes in gold-catalysed reactions have revealed that both conjugated and unconjugated diynes are useful precursors of complex molecules.^13^ For example, we recently reported a gold-catalysed formal [4 + 2] reaction between 1,3-diynes and pyrroles for synthesising 4,7-disubstituted indoles (Scheme 2A, *n* = 0).14*a* This reaction proceeded through a double hydroarylation cascade involving the initial intermolecular hydroarylation of 1,3-diyne at the 2-position of pyrrole, followed by intramolecular hydroarylation. When using skipped diynes as substrates, the formal [5 + 2] reaction efficiently proceeded to produce 1,6-dihydrocyclohepta[*b*]pyrrole derivatives,14*b* which can be considered as homologs of 4,7-disubstituted indoles (Scheme 2A, *n* = 1).

**Scheme 3:**
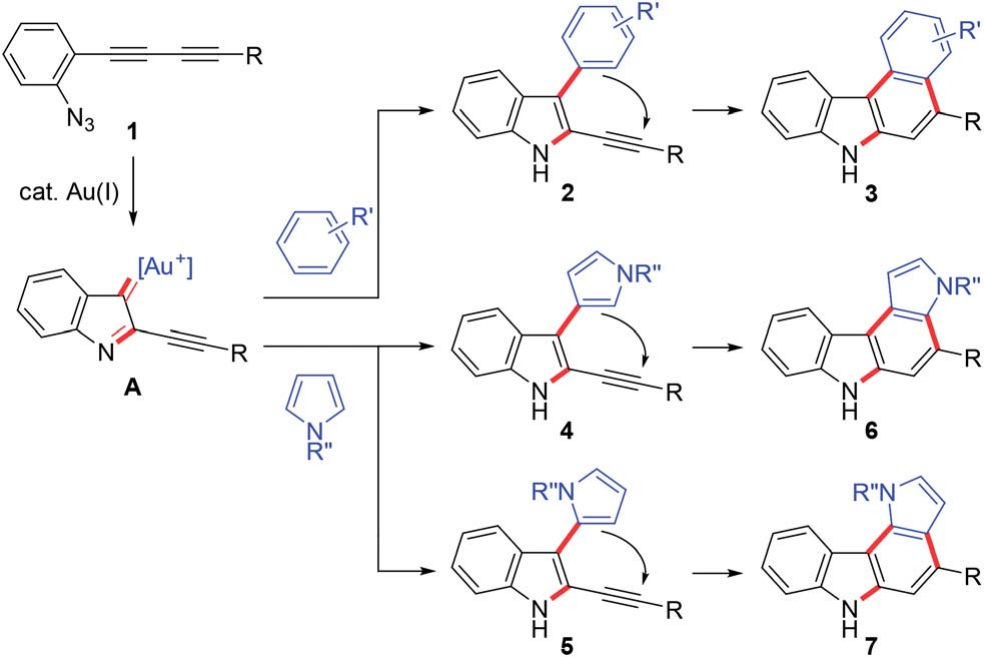
Possible reaction pathways.

We then turned our attention to synthesising aryl-annulated [*c*]carbazoles based on gold-carbenoid formation^15^ using conjugated diynes. In 2011, Gagosz16*a* and Zhang16*b* independently reported the gold(i)-catalysed synthesis of indoles bearing an electron-donating group at the 3-position (Scheme 2B).^17^ The reaction can be rationalised by the formation of a gold carbenoid intermediate followed by a nucleophilic reaction at the carbenoid moiety. As the coupling partners, alcohols and arenes can be used for the reaction to produce 3-substituted indoles. We envisaged that incorporating gold carbenoid chemistry into diyne cyclisation would provide direct access to aryl-annulated [*c*]carbazoles (Scheme 3). Thus, the gold(i)-mediated nucleophilic attack of the azido group of diyne **1** on the proximal alkyne followed by the elimination of nitrogen would produce gold carbenoid species **A**. The electrophilic aromatic substitution of benzene-type arenes with **A** would produce intermediate **2**. Finally, intramolecular hydroarylation toward alkynes^18^ would occur to produce benzo[*c*]carbazole **3**. The challenge of this strategy is controlling the regioselectivity when using pyrrole-type heteroarenes as the coupling partner: whereas the first nucleophilic attack at the pyrrole 3-position would produce pyrrolo[2,3-*c*]carbazole **6**, the first nucleophilic attack at the pyrrole 2-position would give pyrrolo[3,2-*c*]carbazole **7**, through 3-pyrrolylindole intermediates **4** and **5**, respectively. Attention should also be given to the regioselectivity in the second arylation in the reaction with the benzene-type nucleophile (**2** to **3**).

Herein, we report a full account of our study on the direct synthesis of aryl-annulated [*c*]carbazoles by the regioselective gold-catalysed annulation of conjugated diynes and arenes such as benzene, pyrrole, and indole derivatives.^19^ Computational investigations for elucidating the mechanism as well as redox and fluorescence properties of the pyrrolo[2,3-*c*]carbazoles are also presented.

## Results and discussion

### Reaction with benzene derivatives

**Scheme 4:**
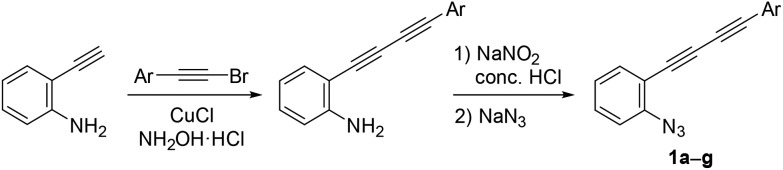
Preparation of azide-diynes.

Azido-substituted diynes **1** were easily prepared through Cadiot–Chodkiewicz coupling^20^ between 2-ethynylaniline and bromoalkynes (Scheme 4). The resulting anilines bearing a conjugated diyne moiety were converted to **1a–g***via* the Sandmeyer reaction with sodium azide (see ESI†).

**Fig. 2:**
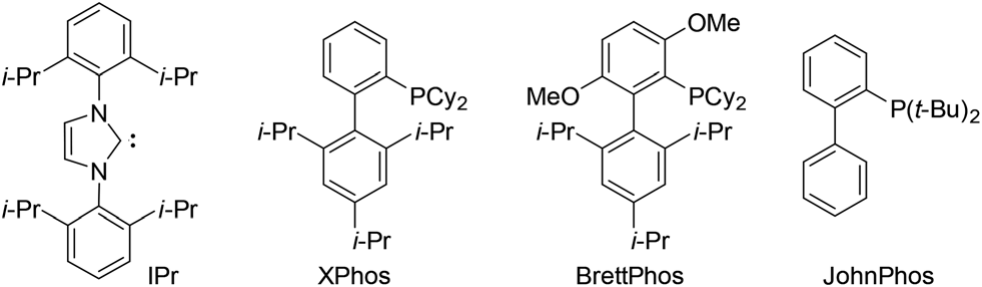
Structures of screened ligands.

We initially screened a variety of different gold catalysts (5 mol%) for the synthesis of benzo[*c*]carbazole using diyne **1a** and anisole **8A** (10 equiv.) (Table 1, entries 1–5). Ph_3_PAuSbF_6_ in 1,2-dichloroethane (DCE) did not promote even the first arylation of the desired transformation (entry 1). IPr, XPhos, and BrettPhos (Fig. 2) were ineffective ligands for bis-cyclisation; however, 3-phenylindole intermediate **2aA** was formed in 36–65% yields (entries 2–4). Fortunately, the annulation reaction was promoted by JohnPhosAu(MeCN)SbF_6_ (entry 5) to provide the desired fused carbazole **3aA** in 44% yield. Next, we examined the choice of reaction solvent using JohnPhosAu(MeCN)SbF_6_ as the catalyst. The reaction using benzene, propan-2-ol, and 1,4-dioxane gave the monocyclisation product **2aA** (63–87% yields) without forming carbazole **3aA** (entries 6–8). Carrying out the reaction in 1,1,2,2-tetrachloroethane (TCE) at 140 °C increased the yield of **3aA** to 55% (entry 9). In this case, the decomposition of JohnPhosAu(MeCN)SbF_6_ at high reaction temperature was anticipated. Thus, the first arylation was conducted at 80 °C and, after the disappearance of the starting material and formation of **2aA** (monitored by TLC), the reaction temperature was raised to 140 °C for the second arylation, giving rise to a higher yield of fused carbazole **3aA** (75% yield, entry 10). Finally, an examination of the stoichiometry revealed that the reaction using excess anisole (as solvent) and 5 mol% BrettPhosAu(MeCN)SbF_6_ at 140 °C improved the yield to 86% (entry 12), whereas the reaction at 80 °C did not reach completion (entry 11). Thus, we used the conditions shown in entry 10 (10 equiv. of arene, condition A) and entry 12 (arene as the solvent, condition B) for further investigations of benzo[*c*]carbazole synthesis.

**Table 1:** Reaction optimization using anisole^*a*^

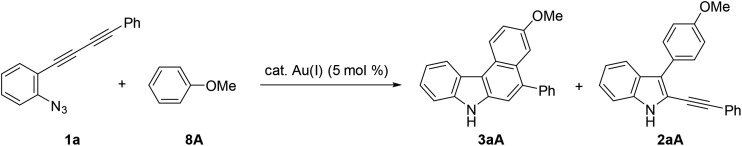					
Entry	Catalyst^*b*^	Solvent^*c*^	Temperature (time)	Yield^*d*^ (%)	
				**3aA**	**2aA**
1	Ph_3_PAuCl/AgSbF_6_	DCE	80 °C (44 h)	0	0
2	IPrAuNTf_2_	DCE	80 °C (24 h)	0	36
3	XPhosAuCl/AgNTf_2_	DCE	80 °C (21 h)	0	57
4	BrettPhosAu(MeCN)SbF_6_	DCE	80 °C (30 h)	0	65
5	JohnPhosAu(MeCN)SbF_6_	DCE	80 °C (26 h)	44	26
6	JohnPhosAu(MeCN)SbF_6_	Benzene	80 °C (10 h)	0	78
7	JohnPhosAu(MeCN)SbF_6_	Propan-2-ol	80 °C (10 h)	0	63
8	JohnPhosAu(MeCN)SbF_6_	1,4-Dioxane	80 °C (10 h)	0	87
9	JohnPhosAu(MeCN)SbF_6_	TCE	140 °C (13 h)	55	0
**10**	**JohnPhosAu(MeCN)SbF**_**6**_	**TCE (condition A)**	**80 °C (1 h), 140 °C (16 h)**	**75**	**0**
11	BrettPhosAu(MeCN)SbF_6_	Anisole	80 °C (15 h)	13	28
**12**	**BrettPhosAu(MeCN)SbF**_**6**_	**Anisole (condition B)**	**140 °C (19.5 h)**	**86**	**0**

^*a*^Reactions were carried out using **1a** (1 equiv.), **8A** (10 equiv.), and the gold catalyst (5 mol%).

^*b*^The ligand structures are shown in Fig. 2. BrettPhosAu(MeCN)SbF_6_, JohnPhosAu(MeCN)SbF_6_, and IPrAuNTf_2_ were prepared in advance. The other catalysts were prepared *in situ* by mixing the AuCl ligand with AgNTf_2_ or AgSbF_6_.

^*c*^DCE = 1,2-dichloroethane, TCE = 1,1,2,2-tetrachloroethane.

^*d*^Isolated yields.

Using these optimised reaction conditions, we then explored the scope of the reaction. Variation of the substitution on the aryl moiety of nucleophile **8** was initially investigated (Table 2). 1,2-Dimethoxybenzene (**8B**) and 1,3-dimethoxybenzene (**8C**) served as suitable nucleophiles for gold-catalysed annulation when used as the solvent (condition B) to give benzo[*c*]carbazoles **3aB** (quant) and **3aC** (95%) in excellent yields. In these cases, the reaction using 10 equiv. of nucleophile (condition A) also permitted 70% and 40% yields of **3aB** and **3aC**, respectively. The reaction with benzodioxole (**8D**) afforded pentacyclic benzo[*c*]carbazole (**3aD**) in 76% yield. Less nucleophilic *o*-xylene (**8E**) also gave the corresponding benzo[*c*]carbazole (**3aE**) in moderate yield (42%), although an increased loading of the gold catalyst (20 mol%) was required. Benzene and toluene did not provide fused carbazoles owing to their lower reactivities. In all cases using arenes **8A–E**, the cascade reaction proceeded in a regioselective manner: the first arylation occurred at the *para*-position of the electron-donating substituent of **8**, and the second hydroarylation occurred at the less-sterically-hindered carbon of the introduced aryl group. We next investigated the reaction using various diynes **1b–g** under condition B.^21^ A methyl substituent at the *ortho*-, *meta*-, or *para*-position of the terminal phenyl group was tolerated, producing the corresponding benzo[*c*]carbazoles (**3bA–3dA**) in good to excellent yields (70–94%). Similarly, the reaction of **1e–g** bearing an electron-donating or -withdrawing group (Cl, NO_2_, or OMe) at the *para*-position gave the desired products **3eA–3gA** (43–74% yield). The lower yield of the nitro derivative **3fA** can be attributed to the less efficient coordination ability of the electron-deficient alkyne(s) to the gold catalyst, which would decrease the probability of the catalyst being activated.

**Table 2:** Scope of benzo[*c*]carbazole synthesis^*a*^

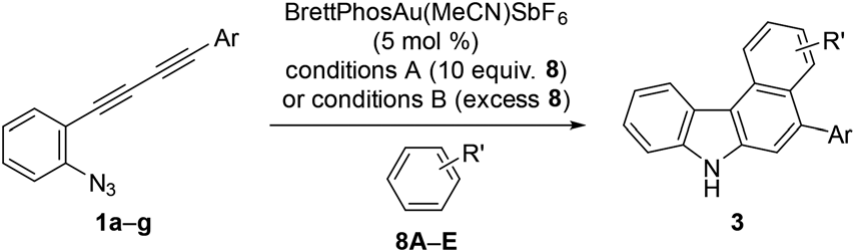
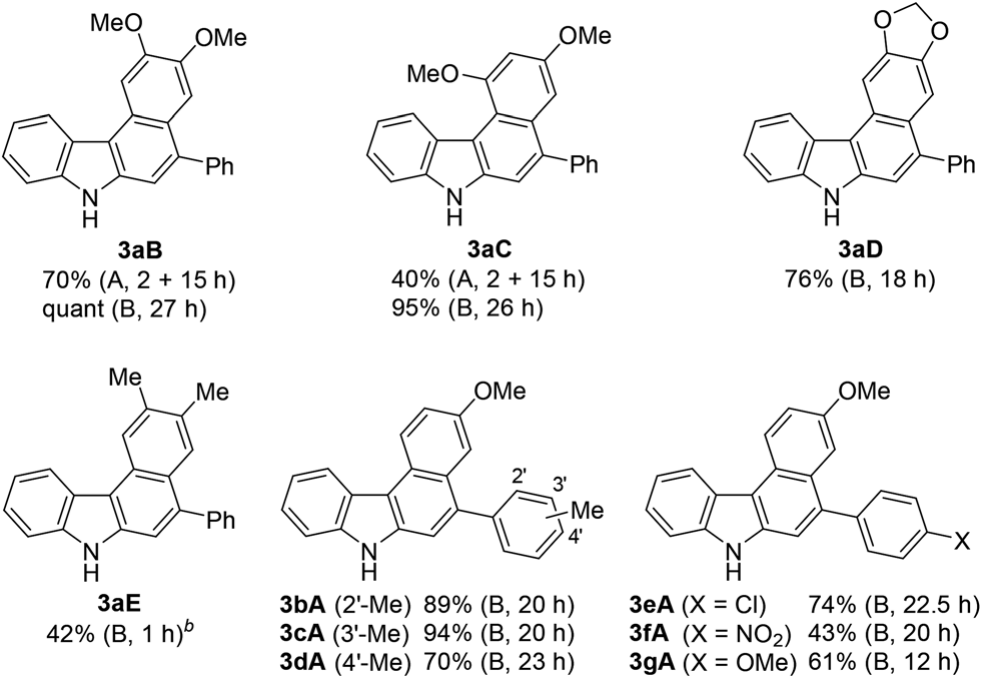

^*a*^Reaction conditions: **1a**, **8**, and the gold catalyst (5 mol%). The reaction conditions employed (condition A or B) and reaction time are shown in parentheses.

^*b*^Catalyst loading was increased to 20 mol%.

### Reaction with pyrrole and indole derivatives

Next, we investigated the synthesis of pyrrolocarbazoles by the reaction with pyrroles **9** (Table 3). The gold-catalysed reaction of conjugated diyne **1a** with NH-pyrrole **9A** produced an isomeric mixture of two annulation products **6aA** and **7aA** in *ca.* 62% yield, along with several unidentified minor products (entry 1). In this case, pyrrolo[3,2-*c*]carbazole **7aA** was obtained as the major isomer (**6aA** : **7aA** = 25 : 75). This result can be readily understood by the more nucleophilic nature of the C2-position of NH-pyrrole than that of the C3-position.^22^ Expecting that the regioselectivity of nucleophilic attack could be controlled by the steric and electronic factors of pyrrole, we subsequently evaluated the impact of substitution at the pyrrole nitrogen (entries 2–6). As expected, regioselectivity was significantly affected by the *N*-substituent: *N*-Boc pyrrole **9F** showed the highest regioselectivity to produce pyrrolo[2,3-*c*]carbazole **6aF** (**6** : **7** = 92 : 8, entry 6), whereas *N*-benzylpyrrole **9B** preferentially produced the corresponding [3,2-*c*]-isomer **7aB** (**6** : **7** = 18 : 82, entry 2).

**Table 3:** Optimisation of the pyrrole structure^*a*^

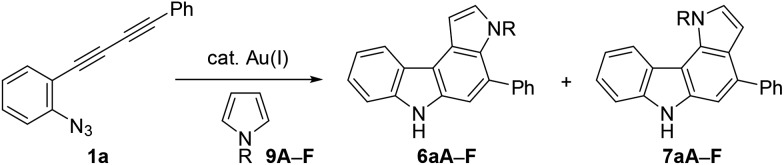					
Entry	Pyrrole	R	Time (h)	Yield^*b*^ (%)	Ratio^*c*^ (**6** : **7**)
1	**9A**	H	8	<62%^*d*^	25 : 75
2	**9B**	Bn	10	62%	18 : 82
3^*e*^	**9C**	Ts	0.5	34%	58 : 42
4	**9D**	CO_2_Me	1.5	62%	81 : 19
5	**9E**	Piv	1.5	60%	82 : 18^*f*^
6	**9F**	Boc	1.5	60%	92 : 8

^*a*^Reaction conditions: **9** (5 equiv.), BrettPhosAu(MeCN)SbF_6_ (5 mol%), DCE, and 80 °C.

^*b*^Combined isolated yields.

^*c*^Determined by ^1^H NMR spectroscopy.

^*d*^Contained small amounts of impurities.

^*e*^Reaction carried out in TCE at 140 °C using 10 mol% of the catalyst.

^*f*^Separation of the minor isomer from other by-products was difficult.

**Fig. 3:**
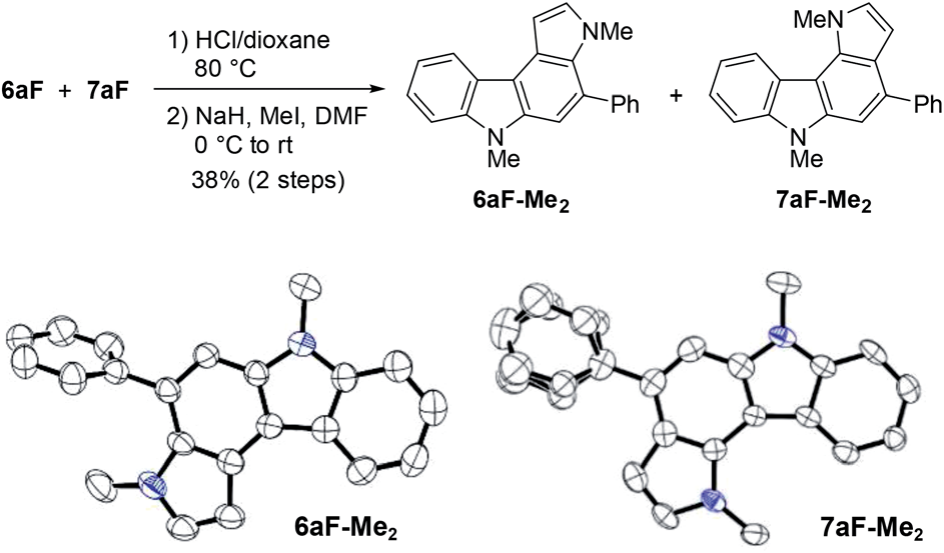
Synthesis and X-ray structures of dimethylated pyrrolocarbazoles. The phenyl group in the latter adopted two orientations in the crystal structure.

The structural elucidation of **6aF** and **7aF** was unambiguously made by X-ray crystallographic analyses of the methylation products **6aF-Me_2_** and **7aF-Me_2_** (Fig. 3). The pyrrolocarbazole moiety adopted a planar geometry as expected, and the twist angle of the phenyl group was 71.1° (for **6aF-Me_2_**) and 26.2–44.0° (for **7aF-Me_2_**).^23^ The larger twist angle of the phenyl group in **6aF-Me_2_** was attributed to the presence of an *N*-methyl group in close proximity to the phenyl group.

We then optimised the reaction conditions for pyrrolocarbazole formation using diyne **1a**, *N*-Boc-pyrrole **9F** (5 equiv.), and various gold catalysts (5 mol%) (Table 4). Whereas Ph_3_PAuCl/AgNTf_2_ showed low reactivity (<5% yield, entry 1), other gold complexes bearing IPr, JohnPhos, XPhos, or BrettPhos as the ligand resulted in the formation of pyrrolo[2,3-*c*]carbazole **6aF** in sufficient regioselectivities (>91 : 9) and moderate yields (55–62%, entries 2–5). Using the most efficient ligand BrettPhos in terms of regioselectivity (**6** : **7** = 94 : 6, entry 5), two other silver salts were tested (AgSbF_6_ and AgOTf, entries 6 and 7, respectively); however, the regioselectivity was not improved. The use of a gold complex prepared in advance slightly improved the reactivity (reaction completed within 0.5 h) and regioselectivity (**6** : **7** = 95 : 5, entries 8 and 9). Solvent screening and investigations of reaction temperature did not improve the yields and product ratios (see ESI†), whereas the reaction at 80 °C was found to be acceptable (entry 10). From these results, we used the conditions shown in entry 8 (condition C) and entry 10 (condition D) for further studies.

**Table 4:** Reaction optimisation using *N*-Boc-pyrrole^*a*^

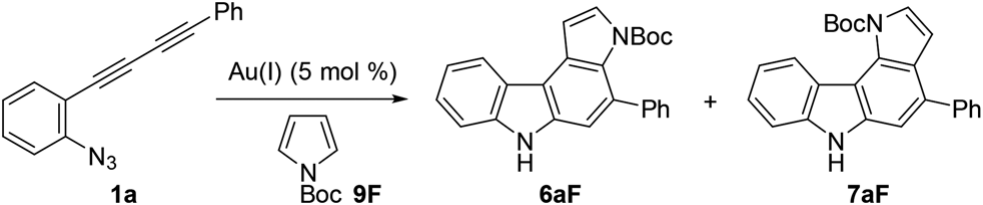				
Entry	Catalyst	Time (h)	Yield^*b*^ (%)	Ratio^*c*^ (**6** : **7**)
1	Ph_3_PAuCl/AgNTf_2_	24	<5^*d*^	87 : 13
2	IPrAuCl/AgNTf_2_	1	60	91 : 9
3	JohnPhosAuCl/AgNTf_2_	1	56	92 : 8
4	XPhosAuCl/AgNTf_2_	1	62	93 : 7
5	BrettPhosAuCl/AgNTf_2_	1	55	94 : 6
6	BrettPhosAuCl/AgSbF_6_	3	51	89 : 11
7	BrettPhosAuCl/AgOTf	20	<12^*d*^	75 : 25
8	BrettPhosAu(MeCN)SbF_6_, (TCE, 110 °C: condition C)	0.5	58	95 : 5
9	BrettPhosAuNTf_2_	0.5	58	95 : 5
10	BrettPhosAu(MeCN)SbF_6_ (DCE, 80 °C: condition D)^*e*^	1.5	60	92 : 8

^*a*^Reaction conditions: **9F** (5 equiv.), gold catalyst (5 mol%), TCE, and 110 °C.

^*b*^Combined isolated yields.

^*c*^Determined by ^1^H NMR spectroscopy.

^*d*^Contained small amounts of impurities.

^*e*^The reaction was conducted in DCE at 80 °C.

We subsequently investigated the scope of pyrrolo[2,3-*c*]carbazole formation (Table 5). The conjugated diynes **1b–i** bearing electron-donating or -withdrawing substituents on both the aryl groups reacted smoothly with pyrrole **9F** to afford the corresponding carbazoles **6bF–6iF** under condition C. The position of the methyl group or introduction of chloro or methoxy substituents at the terminal aryl group did not significantly affect the reaction, and the desired annulation products were efficiently produced (**6** : **7** = 95 : 5). The regioselectivity was slightly decreased when using electron-deficient diyne **1f** substituted by a nitro group. Diynes **1h** and **1i** substituted by a cyano or methoxy group at the *para*-position to the azido group also showed relatively low selectivities (**6** : **7** = 81 : 19–91 : 9).

**Table 5:** Scope of pyrrolo[2,3-*c*]carbazole synthesis^*a*^

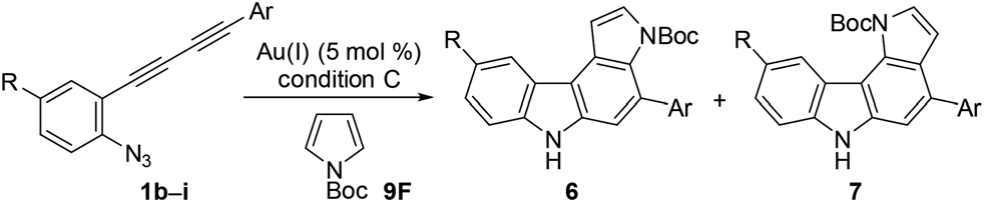
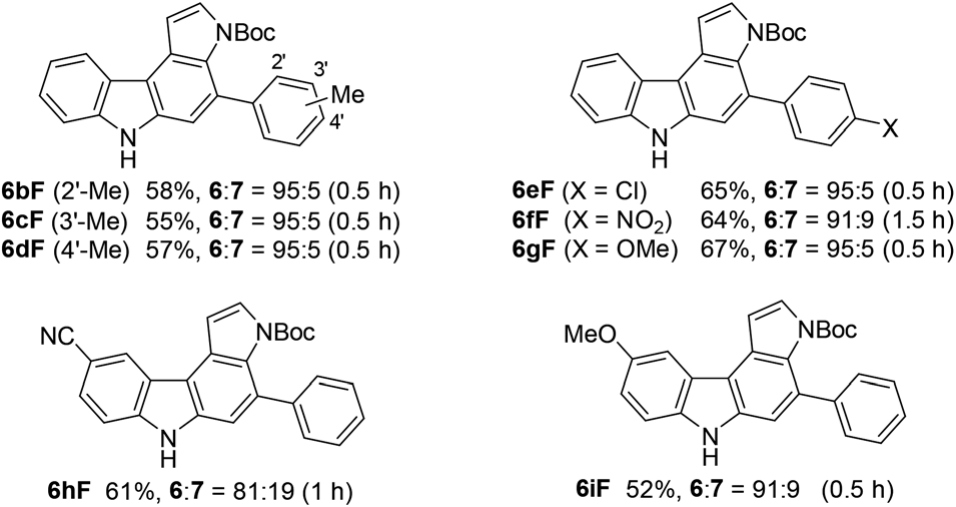

^*a*^Reaction conditions: **9F** (5 equiv.), BrettPhosAu(MeCN)SbF_6_ (5 mol%), TCE, and 110 °C (condition C).

We then applied indole derivatives as the nucleophile for the annulation reaction (Table 6). The reactions of azide-diyne **1a** with N-protected indoles **10A–C** (R^1^ = Boc, Piv, or CO_2_Et) under condition C regioselectively gave the indolo[2,3-*c*]carbazoles **11A–C** as well as several unidentified by-products. The structure of **11A** was confirmed by X-ray analysis after cleavage of the *N*-Boc group and dimethylation,^24^ similar to the cases of **6aF** and **7aF** (Fig. 3). Indoles possess reactive sites other than the desired 2- and 3-positions, which may cause undesired side reactions.16*b* Thus, the introduction of an electron-withdrawing group at the 5-position of indole was examined. As expected, indoles **10D–F** bearing a bromo, chloro, or ethoxycarbonyl group at the 5-position reacted more efficiently to afford indolo[2,3-*c*]carbazoles **11D–F** in better yields (50–67%) under condition D.

**Table 6:** Scope of indolo[2,3-*c*]carbazole synthesis^*a*^

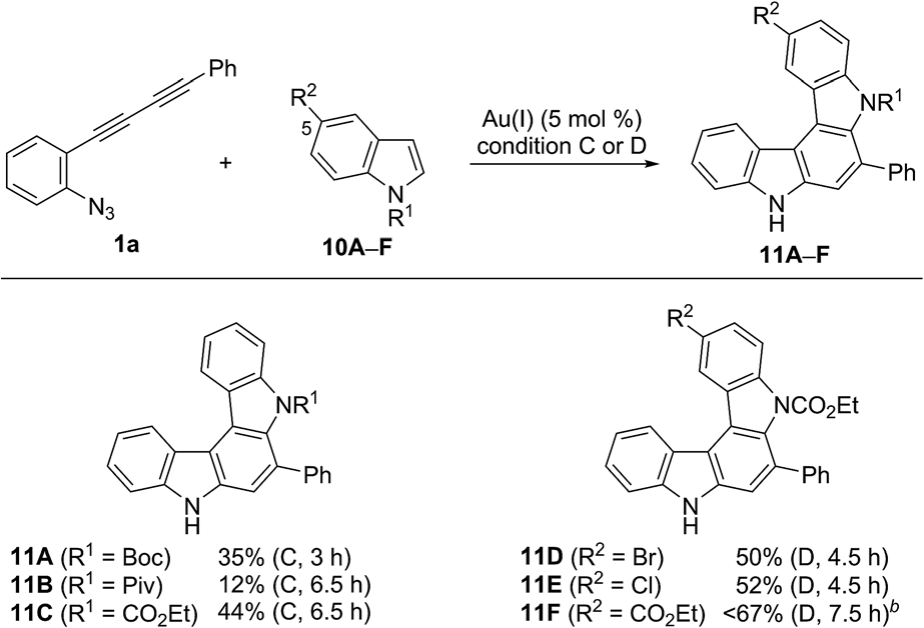

^*a*^Reaction conditions: **10** (5 equiv.) and BrettPhosAu(MeCN)SbF_6_ (5 mol%). The reaction conditions employed (condition C or D) and reaction time are shown in parentheses.

^*b*^Contained small amounts of impurities.

### Reaction mechanism

**Scheme 5:**
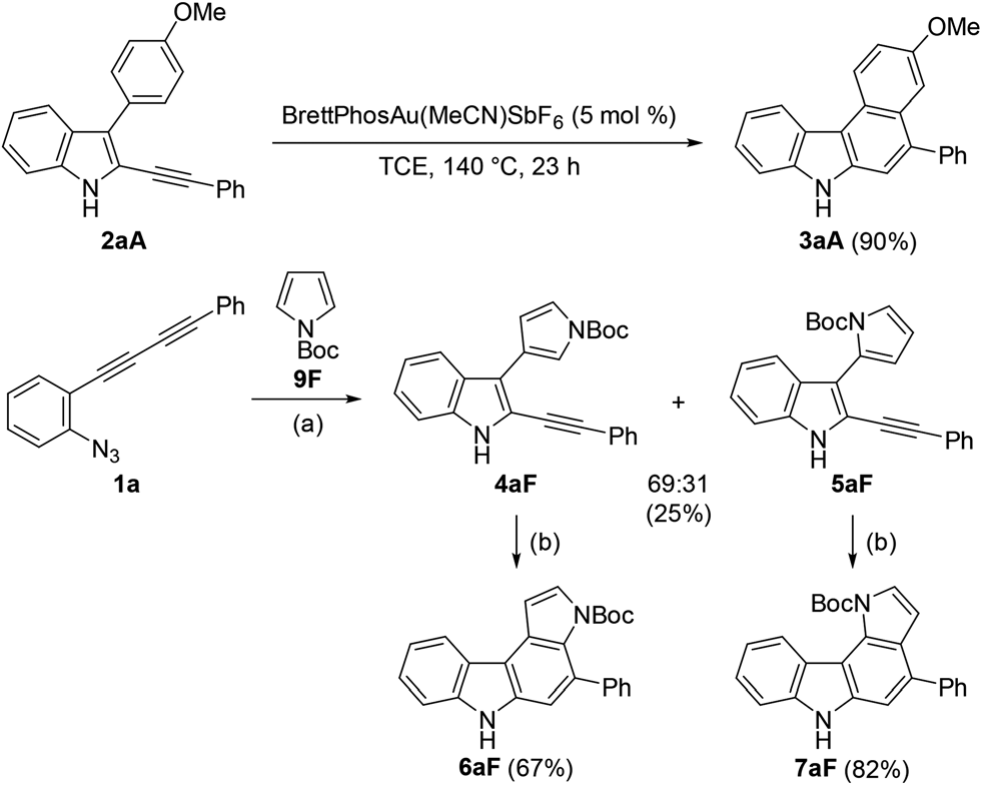
Hydroarylation of the monocyclisation intermediates. Reaction conditions: (a) **9F** (5 equiv.), BrettPhosAu(MeCN)SbF_6_ (5 mol%), DCE, 60 °C, and 1.5 h. (b) BrettPhosAu(MeCN)SbF_6_ (5 mol%), TCE, 110 °C, and 0.5 h.

Although the nucleophilicity of arenes including heteroarenes was well investigated previously, their relative reactivity with *N*-Boc-pyrrole is not well understood.^25^ To better understand the reaction mechanism as well as the relative reactivities of arenes employed in this study, several further experiments were performed. First, the exposure of **2aA** (obtained during the reaction optimisations shown in Table 1) to the gold-catalysed reaction conditions led to its complete conversion to the corresponding benzo[*c*]carbazole **3aA** in 90% yield (Scheme 5). Second, the reaction of **1a** with pyrrole **9F** was intentionally stopped before it reached completion, which afforded alkyne-substituted indoles **4aF** and **5aF** in 25% yield (**4** : **5** = 69 : 31) along with the recovered starting material. These alkynylindoles were consumed completely under gold-catalysed reaction conditions to produce pyrrolocarbazoles **6aF** and **7aF** in 67% and 82% yield, respectively. These results strongly indicated that the reactions proceeded through a stepwise nucleophilic attack of the arenes on the gold carbenoid followed by intramolecular hydroarylation, as per our intended reaction pathway.

**Scheme 6:**
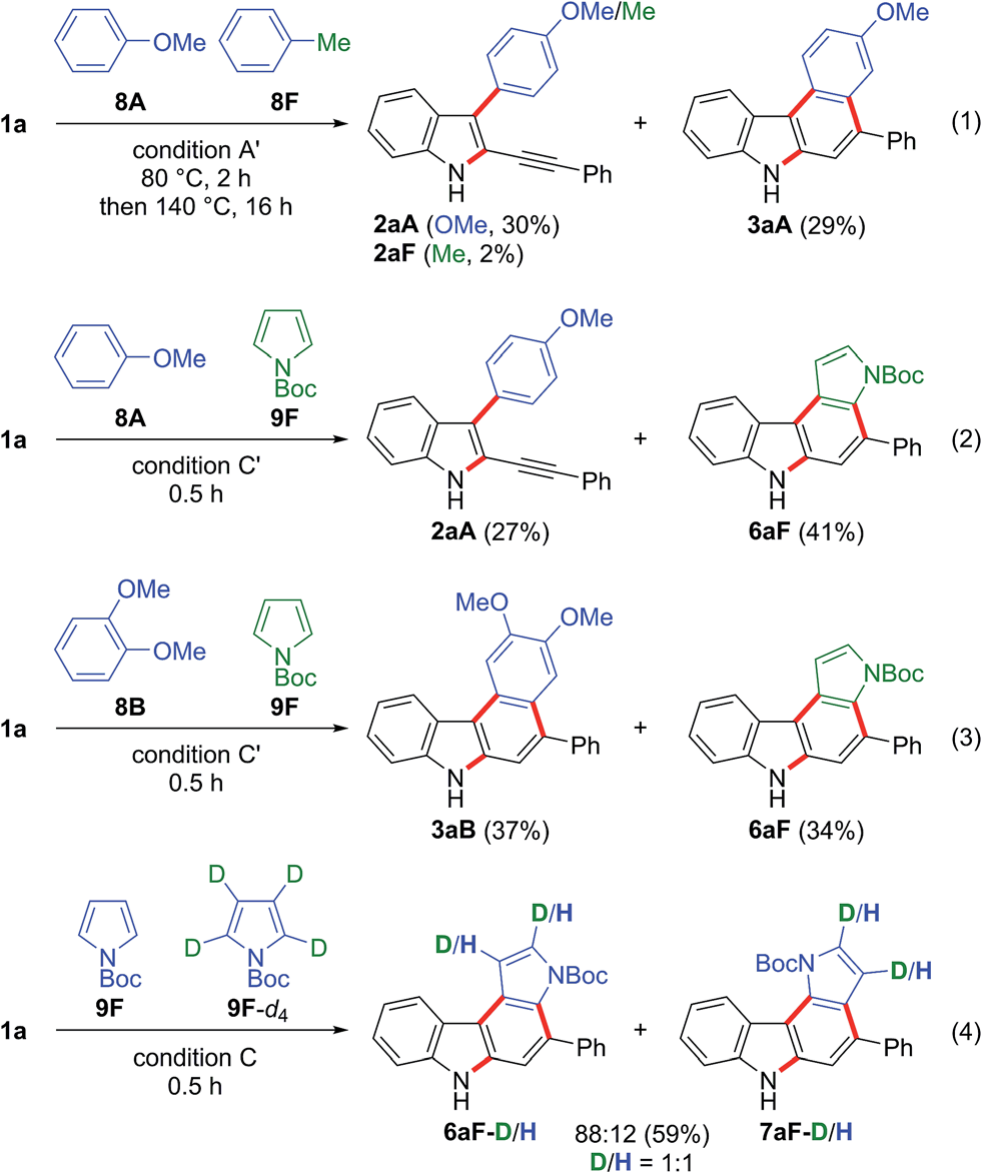
Competition experiments between different nucleophiles. Condition A′: nucleophiles (10 equiv. each), JohnPhosAu(MeCN)SbF_6_ (10 mol%), TCE, 80 °C and then 140 °C. Condition C/C′: nucleophiles (2.5 equiv. each for condition C; 5 equiv. each for condition C′), BrettPhosAu(MeCN)SbF_6_ (5 mol%), TCE, and 110 °C.

Competition experiments using two different arenes were then carried out (Scheme 6). The gold-catalysed reaction of **1a** with anisole **8A** (10 equiv.) and toluene **8F** (10 equiv.) gave the anisole-derived products **2aA** (30%) and **3aA** (29%) along with a small amount of toluene derivative **2aF** (2%) (eqn (1) in Scheme 6). Thus, the first arylation was highly dependent on the nucleophilicity of the arene.^25^ The competition between anisole **8A** (5 equiv.) and *N*-Boc-pyrrole **9F** (5 equiv.) led to the formation of the anisole-derived monocyclised product **2aA** (27%) and pyrrole-derived biscyclised product **6aF** (41%), the latter being the preferred product (eqn (2) in Scheme 6). This result suggested that *N*-Boc-pyrrole **9F** was a slightly more efficient partner in the first arylation than anisole **8A**, and that anisole-derived intermediate **2aA** was significantly less reactive for the second arylation than the pyrrole-derived intermediate. The competition reaction using dimethoxybenzene **8B** and *N*-Boc-pyrrole **9F** gave the biscyclisation products **3aB** (37%) and **6aF** (34%) in comparable yields (eqn (3) in Scheme 6). This result suggested that the second arylation was accelerated by the additional methoxy group located at the *para*-position to the reacting carbon.^26^ We then examined the kinetic isotope effect (eqn (4) in Scheme 6). The competition reaction using *N*-Boc-pyrroles **9F** (2.5 equiv.) and **9F**-*d*_4_ (2.5 equiv.) under condition C gave the corresponding pyrrolocarbazoles **6aF** and **7aF**, where the D/H ratios were 1 : 1 in both products. Thus, deprotonation was not the rate-determining step for the formation of these products. This result suggested that electrophilic aromatic substitution was more likely for the first arylation than C–H insertion.^27^

**Fig. 4:**
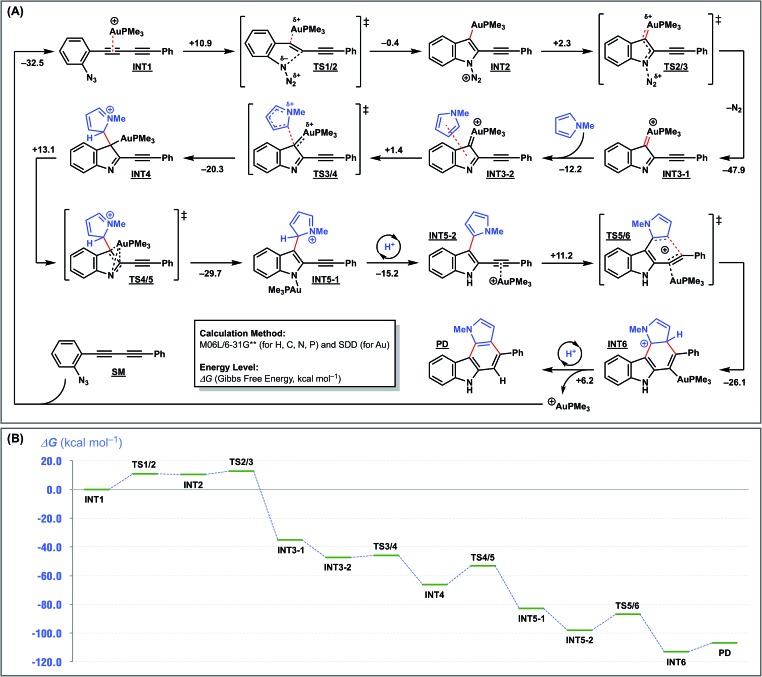
DFT calculations for cyclisation of **1a** with *N*-methylpyrrole [M06L/6-31G** (H, C, N, P) & SDD (Au)].

To further elucidate the reaction mechanism, we undertook density functional theory (DFT) calculations. The calculations were conducted at the M06L/6-31G** (for H, C, N, and P) and SDD (for Au) levels using the formation of pyrrolo[2,3-*c*]carbazole from **1a** and *N*-methylpyrrole as the model reaction (Fig. 4A). As previously proposed,^16^ the reaction is initiated by the intramolecular nucleophilic attack of the azide group on the activated alkyne through **TS1/2** to form an indolyl-gold intermediate **INT2** with a small barrier of 10.9 kcal mol^–1^ and a rather large endothermicity (10.5 kcal mol^–1^ higher than **INT1**). This unfavourable energy loss is compensated for by successive reaction(s). **INT2** ejects nitrogen to form a gold carbenoid intermediate **INT3-1** with a large stabilisation energy (45.6 kcal mol^–1^). Next, the key arylation step occurs by the intermolecular nucleophilic attack of *N*-methylpyrrole on the gold carbenoid **INT3-2** through **TS3/4**, with a small barrier of 1.4 kcal mol^–1^, to produce **INT4**. The gold rearrangement from C to N, with a reasonable barrier of 16.6 kcal mol^–1^, gives an *N*-aurated indole intermediate **INT5-1**. This occurs with the simultaneous re-aromatisation, protodeauration, and re-complexation of the gold catalyst with the internal acetylene, and exothermically provides the pyrrole-substituted indole intermediate **INT5-2**. Finally, 6-*endo-dig* cyclisation of **INT5-2** is promoted by the gold catalyst to produce pyrrolo[2,3-*c*]carbazole (**PD**), which regenerates the active gold catalyst. The entire reaction profile is illustrated in Fig. 4B. All the transition states have reasonable energy barriers (1.4–13.1 kcal mol^–1^). The overall exothermicity is very large because of the formation of one C–N bond, two C–C bonds, and two aromatic rings. This provides the driving force for the overall reaction.

### Electrochemical investigations of pyrrolo[2,3-*c*]carbazoles

**Fig. 5:**
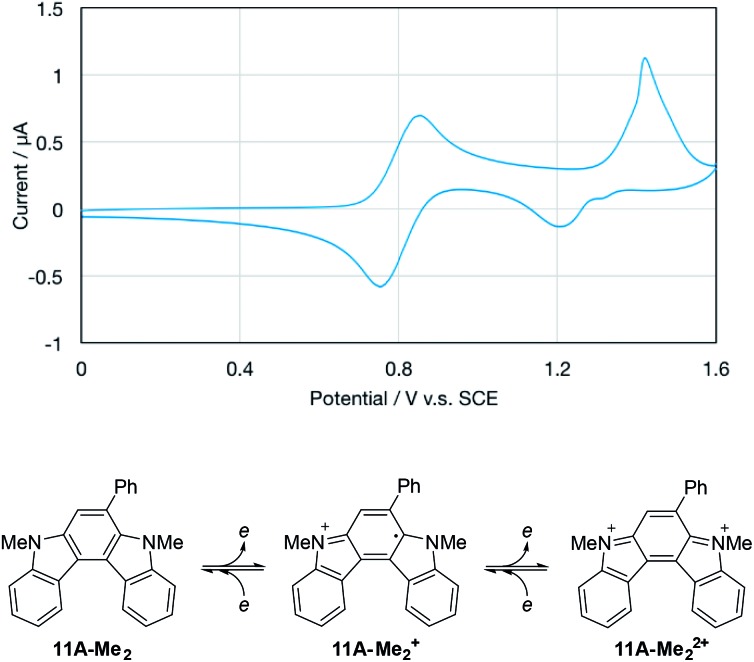
Cyclic voltammogram of **11A-Me_2_** in CH_2_Cl_2_ (upper panel) and the possible redox pathway (lower panel). The irregular peak shape for the second oxidation wave may have been related to partial adsorption of the doubly-charged species on the electrode.

From the viewpoint of the electronic structure, pyrrolo[2,3-*c*]carbazoles **6** and indolo[2,3-*c*]carbazoles **11** could be considered as π-fused 1,4-phenylenediamines. Thus, their cation radicals would be generated as persistent species in the π-extended form9c–e,^28^ of Wurster's blue.^29^ According to voltammetric analyses in CH_2_Cl_2_ (Table 7), the oxidation process of pyrrolo[2,3-*c*]carbazole **6aF-H** was irreversible as in the isomer pyrrolo[3,2-*c*]carbazole **7aF-H**. Substitution with methyl groups at the reactive position (N–H of pyrrole) failed to stabilise the cation radical species, as shown by the irreversible oxidation wave for **6aF-Me_2_**. This was despite the electron-donating nature of the substituents marginally facilitating electrochemical oxidation, as indicated by the less positive oxidation potentials. By fusing the benzene nucleus in **6aF-Me_2_** to furnish the indolo[2,3-*c*]carbazole skeleton, the cation radical species could attain enough persistency. **11A-Me_2_** underwent reversible two-stage one-electron oxidation processes, as shown by the voltammogram (Fig. 5). The redox pathway can be postulated as shown in the scheme, similar to that for 1,4-phenylenediamine.

**Table 7:** Oxidation potentials of pyrrolocarbazoles ^*a*^

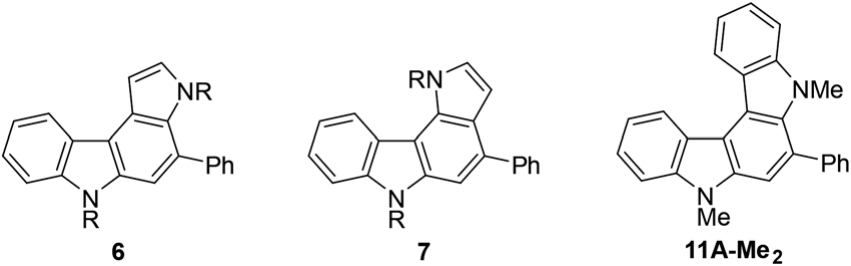			
Entry	Compound	R	Oxidation potential^*a*^
1	**6aF-H**	H	+0.85
2	**6aF-Me_2_**	Me	+0.79
3	**7aF-H**	H	+0.73
4	**7aF-Me**_2_	Me	+0.75
5	**11A-Me**_2_	—	+0.80^*b*^

^*a*^ *E*/*V vs.* SCE, CH_2_Cl_2_ containing 0.1 M Bu_4_NPF_6_, Pt electrode, and 100 mV s^–1^. *E*^ox^ = *E*^pa^ – 0.03 V (for entries 1–5). *E*(Fc/Fc^+^) = +0.53 V under similar conditions.

^*b*^Reversible redox reaction was observed.

**Fig. 6:**
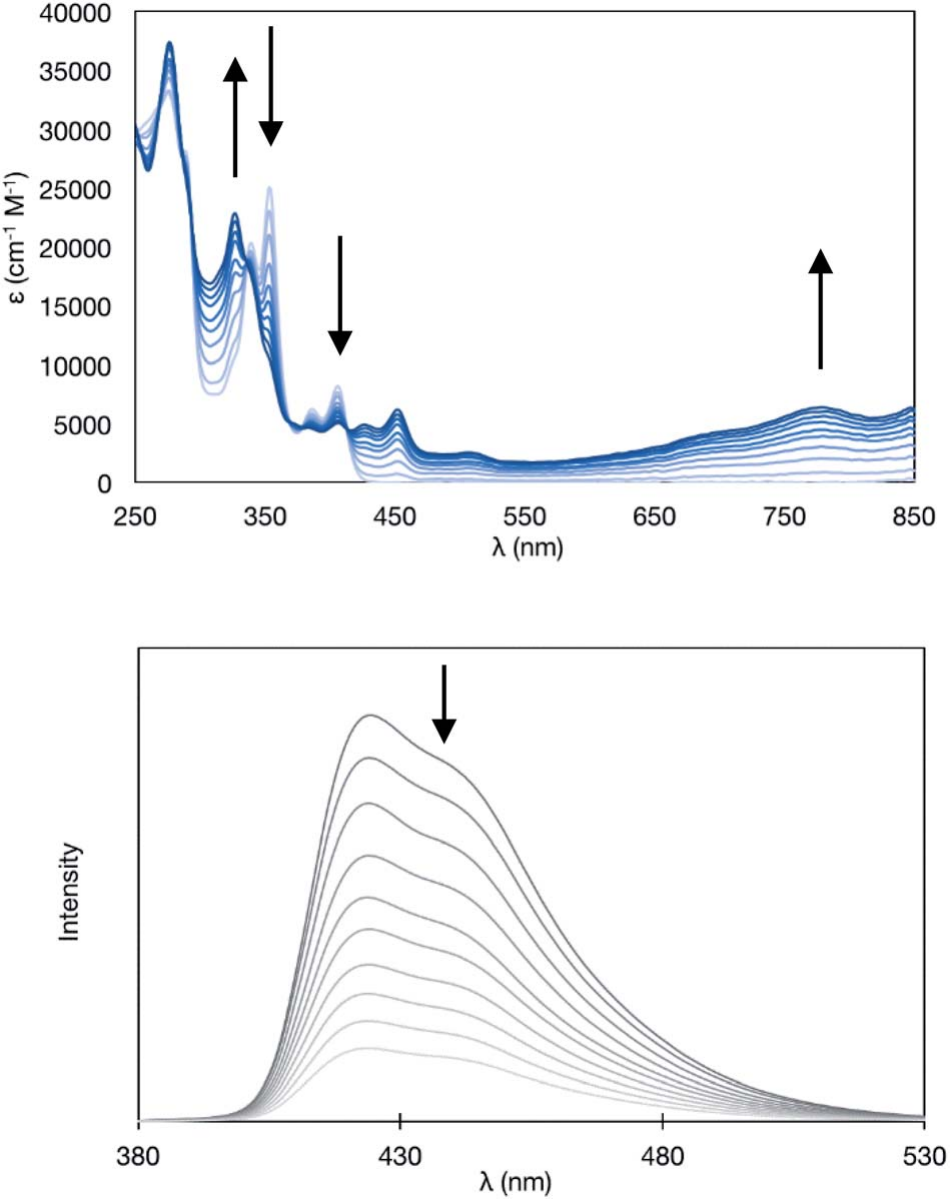
Continuous changes in UV-Vis-NIR (upper panel) and fluorescence (lower panel) spectra upon constant current electrochemical oxidation of **11A-Me_2_** [in CH_2_Cl_2_ (7.1 × 10^–6^M) containing 0.05 M Bu_4_NPF_6_ (20 μA, every 8 min)].

Upon the electrochemical oxidation of **11A-Me_2_** in CH_2_Cl_2_, the colourless solution turned green, which demonstrated its electrochromic nature. A continuous change in ultraviolet-visible-near-infrared (UV-Vis-NIR) absorption was accompanied by several isosbestic points, indicating that **11A-Me_2_** was cleanly oxidised into the corresponding cation radical species (Fig. 6). Wurster's blue exhibits absorption only in the visible region (*λ* < 700 nm). Thus, the observed red shift was induced through π-extension by the fusion of the indole rings. The carbazole skeleton gives rise to fluorescence properties,^30^ so the electrolysis of **11A-Me_2_** also caused a change in the fluorescence (FL) spectrum [*λ*_em_ 424, 442 (sh) nm in CH_2_Cl_2_ (*λ*_ex_ 354 nm)]. The steady decrease in fluorescence with increasing electrochemical oxidation time could be rationalised by the non-fluorescent nature of its cation radical. Such dual electrochromism in which changes occur in both UV-Vis-NIR and FL spectra is rare,^30^ but was also realised in our previous study on benzo[*g*]indolo[2,3-*c*]carbazole derivatives,9c–e which were synthesised through a different mode of the gold(i)-catalysed cascade reaction.9*a* Thus, the gold-catalysed synthesis of annulated carbazoles is a powerful tool for exploring the little developed category of advanced electrochromic systems.

## Conclusions

We have developed a strategy for synthesising aryl-annulated [*c*]carbazoles through the gold-catalysed cascade cyclisation of azido-diynes. The reaction with electron-rich benzenes such as anisole and xylene gave benzo[*c*]carbazoles *via* the functionalisation of two benzene C–H bonds. The use of *N*-Boc-pyrrole and indoles as a coupling partner regioselectively produced their corresponding heteroaryl-annulated carbazoles, namely pyrrolo[2,3-*c*]carbazoles and indolo[2,3-*c*]carbazoles, respectively. The reaction proceeded through the intramolecular nucleophilic attack of azide on the proximal alkyne to form a gold carbenoid species, nucleophilic attack of arenes on the carbenoid, and subsequent 6-*endo-dig* cyclisation of the introduced arene to the other alkyne. This proposed reaction mechanism was well supported by the results of DFT calculations, competition experiments, and deuterium-labeling experiments. An *N*,*N*′-dimethylated derivative of indolo[2,3-*c*]carbazole showed dual UV-Vis-NIR and fluorescence spectral changes on electrolysis, which demonstrates the potential utility of this reaction in materials chemistry.

## Conflicts of interest

There are no conflicts to declare.

## Supplementary Material

Supplementary information

Crystal structure data
