# Supplementary material for: Direct synthesis of aryl-annulated [c]carbazoles by gold(i)-catalysed cascade reaction of azide-diynes and arenes
Source: Chem Sci. 2018 Sep 10;9(44):8416–25. doi: 10.1039/c8sc03525c (PMC6244455; doi:10.1039/c8sc03525c)
Supplement: Supplementary file 1 [file SC-009-C8SC03525C-s001.pdf]

Electronic Supplementary Material (ESI) for Chemical Science.
This journal is © The Royal Society of Chemistry 2018

Supporting information

Direct synthesis of aryl-annulated [*c*]carbazoles by gold(I)-catalysed cascade reaction of azide-diynes and arenes

Yuiki Kawada,[†,#] Shunsuke Ohmura,[†,#] Misaki Kobayashi,[†] Wataru Nojo,[‡] Masaki Kondo,[§,¶] Yuka Matsuda,[†] Junpei Matsuoka,[†] Shinsuke Inuki,[†] Shinya Oishi,[†] Chao Wang,[§,¶] Tatsuo Saito,[§] Masanobu Uchiyama,[*,§,¶] Takanori Suzuki,[*,‡] and Hiroaki Ohno[*,†]

† Graduate School of Pharmaceutical Sciences, Kyoto University, Sakyo-ku, Kyoto 606-8501, Japan

‡ Department of Chemistry, Faculty of Science, Hokkaido University, Sapporo 060-0810, Japan

§ Graduate School of Pharmaceutical Sciences, The University of Tokyo, 7-3-1 Hongo, Bunkyo-ku, Tokyo 113-0033, Japan

¶ Cluster of Pioneering Research (CPR), Advanced Elements Chemistry Laboratory, RIKEN, 2-1 Hirosawa, Wako, Saitama 351-0198, Japan.

# These authors contributed equally to this work.

E-mail: uchiyama@mol.f.u-tokyo.ac.jp (MU); tak@sci.hokudai.ac.jp (TS); hohno@pharm.kyoto-u.ac.jp (HO)

Table of Contents

Reaction Optimisation Using *N*-Boc-pyrrole ······ S2
Experimental Section ······ S3
1. General Methods ······ S3
2. Preparation of the Cyclisation Precursors ······ S3
3. Gold-Catalysed Cascade Cyclisation ······ S11
4. Preparation of the Catalyst ······ S29
5. References ······ S29

NMR Spectra ······ S30

Table S1. Reaction Optimisation Using *N*-Boc-pyrrole [a]

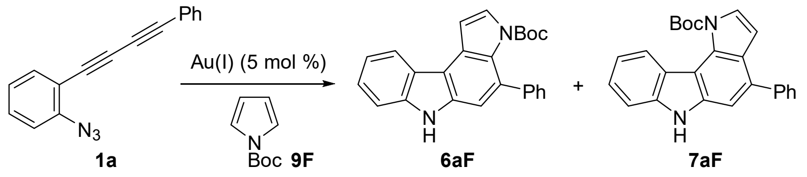

Entry	Catalyst	Solvent	Temperature (°C)	Condition	Time (h)	Yield [b] (%)	Ratio [c] (**6**:**7**)
1	$Ph_3PAuCl/AgNTf_2$	TCE	110		24	<5[d]	87 : 13
2	$IPrAuCl/AgNTf_2$	TCE	110		1	60	91 : 9
3	$JohnPhosAuCl/AgNTf_2$	TCE	110		1	56	92 : 8
4	$XPhosAuCl/AgNTf_2$	TCE	110		1	62	93 : 7
5	$BrettPhosAuCl/AgNTf_2$	TCE	110		1	55	94 : 6
6	$BrettPhosAuCl/AgSbF_6$	TCE	110		3	51	89 : 11
7	BrettPhosAuCl/AgOTf	TCE	110		20	<12[d]	75 : 25
8	**BrettPhosAu(MeCN)SbF_6**	**TCE**	**110**	**C**	**0.5**	**58**	**95 : 5**
9	$BrettPhosAuNTf_2$	TCE	110		0.5	58	95 : 5
10	**BrettPhosAu(MeCN)SbF_6**	**DCE**	**80**	**D**	**1.5**	**60**	**92 : 8**
11	BrettPhosAu(MeCN)SbF_6	toluene	80		24	37	88 : 12
12	BrettPhosAu(MeCN)SbF_6	MeCN	80		30	<48[d]	87 : 13
13	BrettPhosAu(MeCN)SbF_6	EtOH	reflux		24	<36[d]	52 : 48
14	BrettPhosAu(MeCN)SbF_6	AcOH	80		2	<49[d]	53 : 47
15	BrettPhosAu(MeCN)SbF_6	DCE	RT		72	59	72 : 28
16	BrettPhosAu(MeCN)SbF_6	DCE	50		10	62	90 : 10
17	BrettPhosAu(MeCN)SbF_6	TCE	140		0.25	51	91 : 9

[a] Reaction conditions: **9F** (5 equiv.), gold catalyst (5 mol %). [b] Combined isolated yields. [c] Determined by ^{1}H NMR spectroscopy. [d] Contained small amounts of impurities.

Experimental Section

1. General Methods

IR spectra were determined on a JASCO FT/IR-4100 spectrometer. Exact mass (HRMS) spectra were recorded on JMS-HX/HX 110A mass spectrometer or Shimadzu LC-ESI-IT-TOF-MS equipment. ^{1}H NMR spectra were recorded using a JEOL AL-400 or JEOL AL-500 spectrometer at 500 MHz frequency. Chemical shifts are reported in δ (ppm) relative to Me_4Si (in $CDCl_3$) as internal standard. ^{13}C NMR spectra were recorded using a JEOL AL-500 and referenced to the residual solvent signal. Melting points were measured by a hot stage melting points apparatus (uncorrected). For column chromatography, silica gel (Wakogel C-200: Wako Pure Chemical Industries, Ltd), and amine silica gel (CHROMATOREX NH-DM1020: Fuji Silysia Chemical Ltd) were employed.

2. Preparation of the Cyclisation Precursors

2-Ethynylaniline (S1)[1,2]

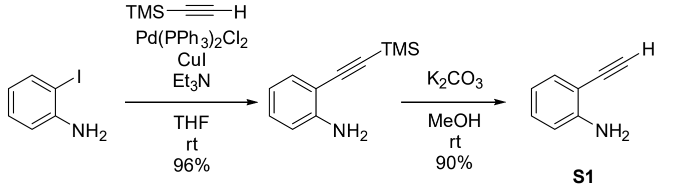

S1

A mixture of 2-iodoaniline (4.38 g, 20.0 mmol), TMS acetylene (3.39 mL, 24.0 mmol), $PdCl_2(PPh_3)_2$ (337 mg, 0.48 mmol), CuI (91.4 mg, 0.48 mmol), and Et_3N (13.9 mL, 100 mmol) in THF (20 mL) was stirred at room temperature under Ar for 3 h. The mixture was filtered through a pad of Celite and concentrated in vacuo. The residue was purified by column chromatography on silica gel (hexane/EtOAc = 20/1) to give 2-[(trimethylsilyl)ethynyl]-aniline[2] (3.65 g, 96%) as an orange oil. To a solution of this oil (1.70 g, 9.00 mmol) in MeOH (30 mL) was added K_2CO_3 (410 mg, 2.97 mmol). The reaction mixture was stirred at room temperature for 2 h and concentrated in vacuo. The residue was diluted with H_2O. The organic layer was washed with H_2O and brine, dried over $MgSO_4$, filtered, and concentrated in vacuo to give **S1** (951 mg, 90%) as a brown oil. The spectral data were in good agreement with those previously reported.[2]

(Bromoethynyl)benzene (S2)[1,3]

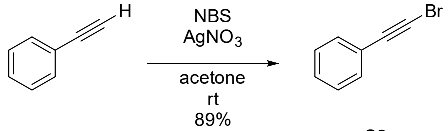

S2

To a solution of ethynylbenzene (1.27 mL, 10.0 mmol) in acetone (50 mL) were added NBS (1.96 g, 11.0 mmol) and $AgNO_3$ (170 mg, 1.00 mmol). The mixture was stirred at room temperature for 7 h. The mixture was diluted with *n*-hexane and filtered through a pad of silica gel. The filtrate was concentrated in vacuo to give **S2** (1.74 g, 89%) as a brown oil. The spectral data were in good agreement with those previously reported.[3]

2-(Phenylbuta-1,3-diyn-1-yl)aniline (S3)[1,4a]

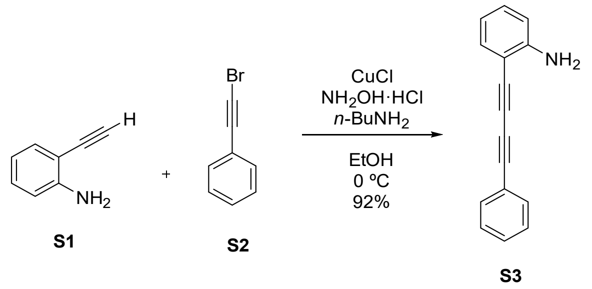

To a mixture of **S1** (1.06 g, 9.09 mmol), CuCl (45.0 mg, 0.455 mmol), $NH_2OH \cdot HCl$ (253 mg, 3.64 mmol), and *n*-$BuNH_2$ (2.26 mL, 22.8 mmol) in dry EtOH (22.8 mL) was added a solution of **S2** (2.14 g, 11.8 mmol) in dry EtOH (4.55 mL) via dropping funnel at 0 ºC under Ar.[4b] The mixture was stirred at room temperature for 1.5 h and concentrated in vacuo. The residue was diluted with Et_2O. The organic layer was washed with saturated aqueous NH_4Cl, H_2O, and brine, dried over Na_2SO_4, filtered, and concentrated in vacuo. The filtrate was purified by column chromatography on silica gel (hexane/EtOAc = 10/1) to give **S3** (1.80 g, 92%) as a light yellow powder. The spectral data were in good agreement with those previously reported[4a]: ^{1}H NMR (500 MHz, $CDCl_3$) δ: 4.32 (s, 2H), 6.69-6.70 (m, 2H), 7.16 (dd, J = 7.5, 1.5 Hz, 1H), 7.32-7.39 (m, 4H), 7.52-7.54 (m, 2H); ^{13}C NMR (125 MHz, $CDCl_3$) δ: 73.9, 78.7, 78.9, 82.6, 105.7, 114.2, 117.7, 121.5, 128.3 (2C), 129.0, 130.5, 132.2 (2C), 132.8, 149.5.

1-Azido-2-(phenylbuta-1,3-diyn-1-yl)benzene (1a)[1,5]

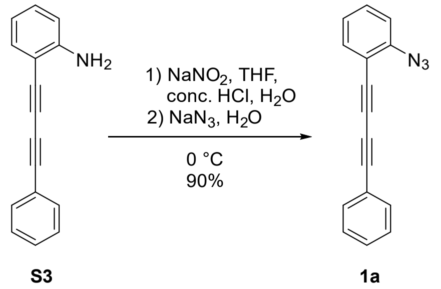

A solution of **S3** (1.09 g, 5.00 mmol) in THF/H_2O/conc. HCl (1/1/1, 10.0 mL) was cooled to 0 ºC. To the solution was added $NaNO_2$ (690 mg, 10.0 mmol) in H_2O (10.0 mL) via dropping funnel at 0 ºC. After the mixture was stirred at 0 °C for 15 min, NaN_3 (650 mg, 10.0 mmol) in H_2O (10.0 mL) was slowly added to the mixture at 0 °C,[6] and the mixture was stirred for 3 h. The reaction mixture was quenched with H_2O. The resulting mixture was extracted with EtOAc twice. The combined organic layer was washed with brine, dried over $MgSO_4$, filtered, and concentrated in vacuo. The residue was purified by column chromatography on silica gel (hexane/EtOAc = 20/1) to give **1a** (1.09 g, 90%) as an light yellow powder: mp 93–96 ºC; IR (neat) 2130, 2100; ^{1}H NMR (500 MHz, $CDCl_3$) δ: 7.11 (t, J = 7.0 Hz, 1H), 7.15 (d, J = 8.5 Hz, 1H), 7.33-7.41 (m, 4H), 7.50-7.54 (m, 3H); ^{13}C NMR (125 MHz, $CDCl_3$) δ: 73.6, 77.0, 79.3, 83.3, 113.8, 118.5, 121.6, 124.6, 128.4 (2C), 129.3, 130.4 132.5 (2C), 134.4, 142.6; HRMS (ESI) calcd for $C_{16}H_{10}N_3$ [M+H]$^+$: 244.0869, found 244.0867.

2-(*o*-Tolylbuta-1,3-diyn-1-yl)aniline (S5)

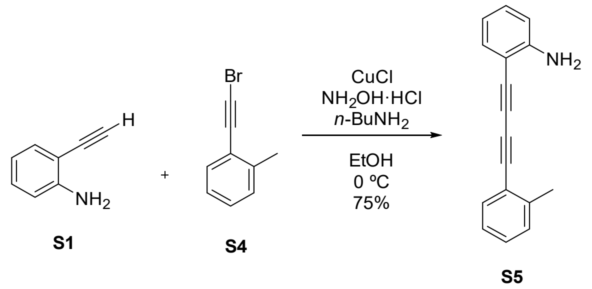

By a procedure identical with that described for the preparation of **S3**, **S1** (451 mg, 3.85 mmol) was converted to **S5** (671 mg, 75%) by the reaction with **S4** (983 mg, 5.04 mmol): yellow powder; mp 100–101.5 ºC; IR (neat) 3479, 3392, 2211, 2137; ^{1}H NMR (400 MHz, $CDCl_3$) δ: 2.49 (s, 3H), 4.32 (s, 2H), 6.67-6.71 (m, 2H), 7.14-7.18 (m, 2H), 7.21-7.28 (m, 2H) 7.35 (d, J = 8.0 Hz, 1H), 7.49 (d, J = 7.6 Hz, 1H); ^{13}C NMR (125 MHz, $CDCl_3$) δ: 20.7, 77.4, 79.1 (2C), 81.7, 106.2, 114.4, 117.9, 121.6, 125.7, 129.1, 129.6, 130.6, 132.8, 133.1, 141.6, 149.5; HRMS (ESI) calcd for $C_{17}H_{14}N$ $[M+H]^+$: 232.1126, found 232.1114

1-Azido-2-(*o*-tolylbuta-1,3-diyn-1-yl)benzene (1b)

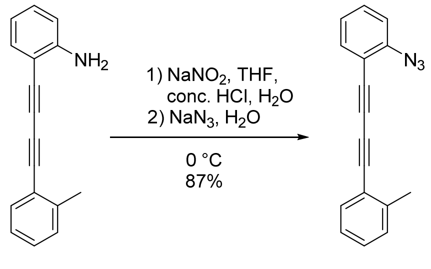

By a procedure identical with that described for the preparation of **1a**, **S5** (550 mg, 2.38 mmol) was converted to **1b** (530 mg, 87%): light yellow powder: mp 61–63 ºC; IR (neat) 2127, 2094; ^{1}H NMR (500 MHz, $CDCl_3$) δ: 2.49 (s, 3H), 7.09-7.17 (m, 3H), 7.20-7.28 (m, 2H), 7.38 (ddd, J = 7.9, 7.9, 1.5 Hz, 1H), 7.49-7.52 (m, 2H); ^{13}C NMR (125 MHz, $CDCl_3$) δ: 20.7, 77.1, 77.5, 79.4, 82.4, 113.9, 118.5, 121.4, 124.6, 125.6, 129.3, 129.6 (2C), 130.3, 134.4, 141.8, 142.5; HRMS (FAB) calcd for $C_{17}H_{12}N_3$ $[M+H]^+$: 258.1031, found 258.1026.

2-(*m*-Tolylbuta-1,3-diyn-1-yl)aniline (S7)

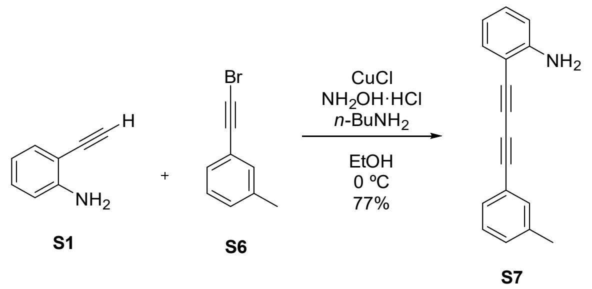

By a procedure identical with that described for the preparation of **S3**, **S1** (421 mg, 3.59 mmol) was converted to **S7** (637 mg, 77%) by the reaction with **S6** (912 mg, 4.67 mmol: yellow powder; mp 67–69 ºC; IR (neat) 3476, 3378, 2207, 2136; ^{1}H NMR (500 MHz, $CDCl_3$) δ: 2.34 (s, 3H), 4.32 (s, 2H), 6.66-6.70 (m, 2H), 7.13-7.26 (m, 3H),

7.32-7.35 (m, 3H); ^{13}C NMR (125 MHz, $CDCl_3$) δ: 21.2, 73.5, 78.4, 79.1, 82.9, 106.2, 114.4, 117.9, 121.6, 128.3, 129.5, 130.1, 130.6, 133.0, 133.1, 138.2, 149.5; HRMS (ESI) calcd for $C_{17}H_{14}N$ $[M+H]^+$: 232.1126, found 232.1127

1-Azido-2-(*m*-tolylbuta-1,3-diyn-1-yl)benzene (1c)

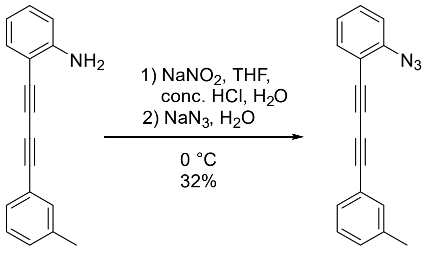

By a procedure identical with that described for the preparation of **1a**, **S7** (500 mg, 2.16 mmol) was converted to **1c** (180 mg, 32%): beige powder; mp 70–71 ºC; IR (neat) 2126, 2094; ^{1}H-NMR ($CDCl_3$) δ: 2.34 (s, 3H), 7.09-7.12 (m, 1H), 7.15 (d, *J* = 7.5 Hz, 1H), 7.19 (d, *J* = 8.0 Hz, 1H), 7.21 (dd, *J* = 8.0, 8.0 Hz, 1H), 7.33-7.35 (m, 2H), 7.38 (ddd, *J* = 8.0, 8.0, 1.5 Hz, 1H), 7.50 (dd, *J* = 8.0, 1.5 Hz, 1H); ^{13}C NMR (125 MHz, $CDCl_3$) δ: 21.7, 73.0, 76.6, 79.5, 83.6, 114.0, 118.5 (2C), 124.7, 129.2 (2C), 130.3, 132.4 (2C), 134.4, 139.8, 142.5; HRMS (FAB) calcd for $C_{17}H_{12}N_3$ $[M+H]^+$: 258.1031, found 258.1037.

2-(*p*-Tolylbuta-1,3-diyn-1-yl)aniline (S9)[4a]

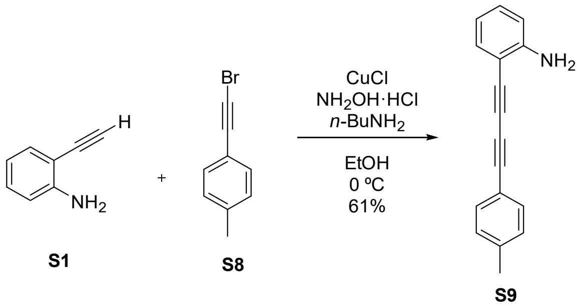

By a procedure identical with that described for the preparation of **S3**, **S1** (805 mg, 6.87 mmol) was converted to **S9** (966 mg, 61%) by the reaction with **S8** (2.14 g, 11.8 mmol): yellow powder. The spectral data were in good agreement with those previously reported.[4a]

1-Azido-2-(*p*-tolylbuta-1,3-diyn-1-yl)benzene (1d)

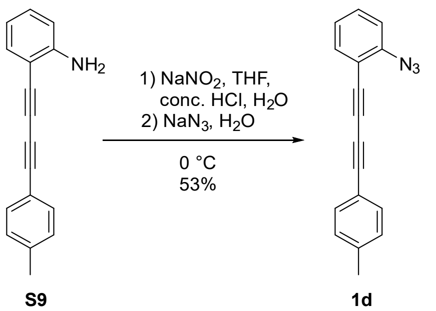

By a procedure identical with that described for the preparation of **1a**, **S9** (966 mg, 4.18 mmol) was converted to **1d** (570 mg, 53%): yellow powder; mp 100–102 ºC; IR (neat) 2116, 2092; ^{1}H NMR (500 MHz, $CDCl_3$) δ: 2.37 (s,

3H), 7.08-7.15 (m, 1H), 7.13-7.15 (m, 3H), 7.36-7.39 (m, 1H), 7.42-7.44 (m, 2H), 7.50 (dd, J = 7.7, 1.4 Hz, 1H); ^{13}C NMR (125 MHz, CDCl$_3$) δ: 21.6, 73.0, 76.6, 79.5, 83.6, 114.0, 118.5 (2C), 124.6, 129.2 (3C), 130.2, 134.4 (2C), 139.8, 142.5; HRMS (FAB) calcd for $C_{17}H_{12}N_3$ [M+H]$^+$: 258.1031, found 258.1026.

2-[(4-Chlorophenyl)buta-1,3-diyn-1-yl]aniline (S11)

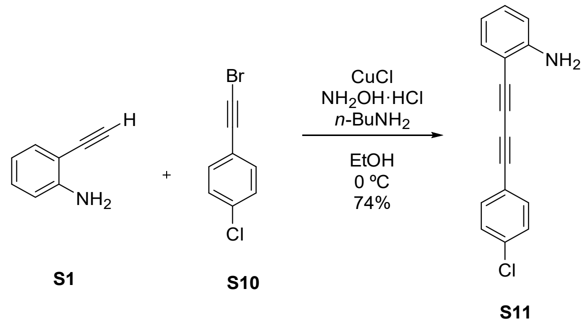

By a procedure identical with that described for the preparation of **S3**, **S1** (389 mg, 3.32 mmol) was converted to **S11** (617 mg, 74%) by the reaction with **S10** (932 mg, 4.32 mmol): yellow powder; mp 134-136 ºC; IR (neat) 3477, 3378, 2206, 2139; ^{1}H NMR (400 MHz, CDCl$_3$) δ: 4.31 (s, 2H), 6.67-6.71 (m, 2H), 7.17 (dd, J = 8.8, 8.0 Hz, 1H), 7.31-7.35 (m, 3H), 7.45 (dd, J = 6.7, 2.0 Hz, 2H); ^{13}C NMR (125 MHz, CDCl$_3$) δ: 74.9, 78.8, 79.2, 81.4, 105.9, 114.4, 118.0, 120.4, 128.9 (2C), 130.8, 133.1 (2C), 133.5, 135.3, 149.6; HRMS (ESI) calcd for $C_{16}H_{11}ClN$ [M+H]$^+$: 252.0580, found 252.0575.

1-Azido-2-[(4-chlorophenyl)buta-1,3-diyn-1-yl]benzene (1e)

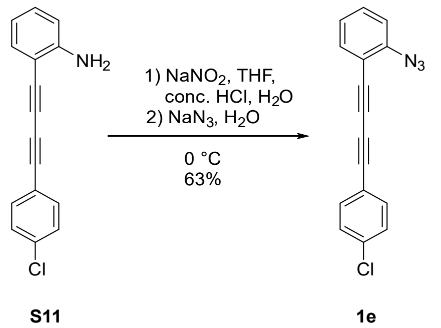

By a procedure identical with that described for the preparation of **1a**, **S11** (500 mg, 1.99 mmol) was converted to **1e** (351 mg, 63%): white powder; mp 144–145 ºC; IR (neat) 2129, 2097; ^{1}H NMR (500 MHz, CDCl$_3$) δ: 7.09-7.16 (m, 2H), 7.31 (d, J = 8.6 Hz, 2H), 7.37-7.41 (m, 1H), 7.45 (d, J = 8.6 Hz, 2H), 7.50 (dd, J = 8.0, 1.1 Hz, 1H); ^{13}C NMR (125 MHz, CDCl$_3$) δ: 74.6, 77.5, 79.0, 82.0, 113.6, 118.5, 120.1, 124.7, 128.8 (2C), 130.5, 133.7 (2C), 134.4, 135.5, 142.7; HRMS (FAB) calcd for $C_{16}H_9ClN_3$ MH$^+$): 278.0485, found 278.0481.

2-[(4-Nitrophenyl)buta-1,3-diyn-1-yl]aniline (S13)

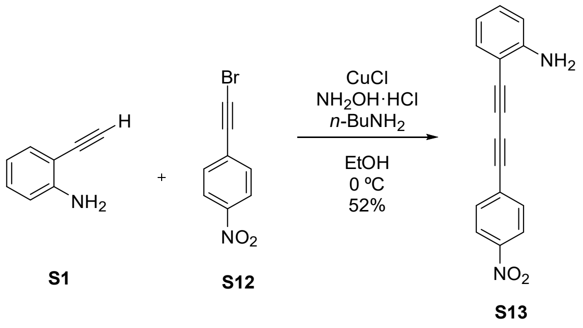

By a procedure identical with that described for the preparation of **S3**, **S1** (562 mg, 4.80 mmol) was converted to **S13** (653 mg, 52%) by the reaction with **S12** (1.42 g, 6.24 mmol): orange powder; mp 215–217 ºC; IR (neat) 3499, 3397, 2127, 2097, 1591; ^{1}H NMR (500 MHz, $CDCl_3$) δ: 4.34 (s, 2H), 6.69-6.72 (m, 2H), 7.21 (ddd, J = 7.8, 7.8, 1.0 Hz, 1H), 7.36 (d, J = 6.9 Hz, 1H), 7.66 (d, J = 8.5 Hz, 2H), 8.23 (d, J = 8.5Hz, 2H); ^{13}C NMR (125 MHz, $CDCl_3$) δ: 78.4, 79.0, 80.4, 91.2, 105.3, 114.5 (2C), 118.1, 123.7, 128.9, 131.3, 133.0 (2C), 133.2, 133.4, 149.9; HRMS (FAB^{+}) calcd for $C_{16}H_{11}N_2O_2$ $[M+H]^+$: 263.0821, found 263.0825.

1-Azido-2-[(4-nitrophenyl)buta-1,3-diyn-1-yl]benzene (1f)

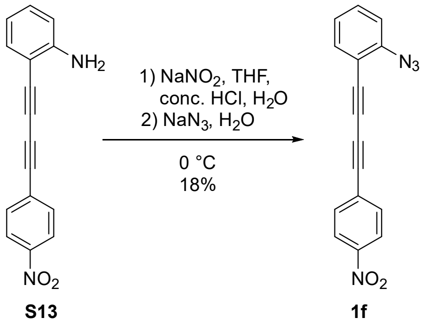

By a procedure identical with that described for the preparation of **1a**, **S13** (550 mg, 2.10 mmol) was converted to **1f** (108 mg, 18%): yellow powder; mp 168–171 ºC; IR (neat) 2131, 2100, 1518; ^{1}H NMR (500 MHz, $CDCl_3$) δ: 7.14 (dd, J = 7.6, 7.6 Hz, 1H), 7.18 (d, J = 7.6 Hz, 1H), 7.41-7.45 (m, 1H), 7.53 (dd, J = 7.6, 1.2 Hz, 1H), 7.66-7.71 (m, 2H), 8.20-8.25 (m, 2H); ^{13}C NMR (125 MHz, $CDCl_3$) δ: 78.4, 78.6, 79.9, 80.7, 113.1, 118.6, 123.7 (2C), 124.7, 128.6, 131.0, 133.2, 134.5 (2C), 143.0, 147.5; HRMS (FAB) calcd for $C_{16}H_9N_4O_2$ $[M+H]^+$: 289.0726, found 289.0722.

2-[(4-Methoxyphenyl)buta-1,3-diyn-1-yl]aniline (S15)[4a]

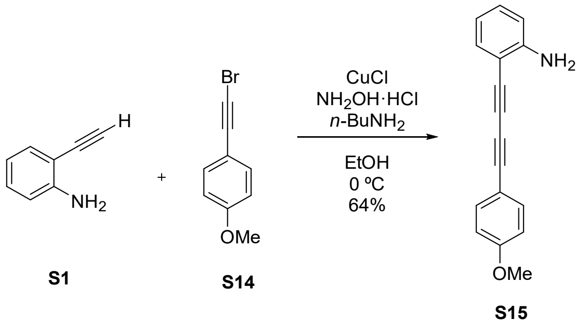

By a procedure identical with that described for the preparation of **S3**, **S1** (972 mg, 8.30 mmol) was converted to **S15** (1.31 g, 64%) by the reaction with **S14** (2.61 g, 12.4 mmol). The spectral data were in good agreement with those previously reported.[4a]

1-Azido-2-[(4-methoxyphenyl)buta-1,3-diyn-1-yl]benzene (1g)

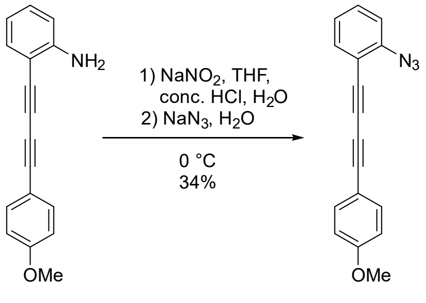

By a procedure identical with that described for the preparation of **1a**, **S15** (495 mg, 2.00 mmol) was converted to **1g** (188 mg, 34%): orange solid: mp 89–90 ºC; IR (neat) 2127, 2096; ^{1}H NMR (500 MHz, $CDCl_3$) δ: 3.83 (s, 3H), 6.86 (d, J = 7.6 Hz, 2H), 7.08-7.15 (m, 2H), 7.37 (ddd, J = 7.9, 7.9, 1.7 Hz, 1H), 7.46-7.51 (m, 3H); ^{13}C NMR (125 MHz, $CDCl_3$) δ: 55.3, 72.5, 76.4, 77.2, 79.6, 83.6, 113.5, 114.1, 118.5 (2C), 124.6, 130.2, 134.2, 134.3 (2C), 142.5, 160.4; HRMS (FAB) calcd for $C_{17}H_{12}N_3O$ $[M+H]^+$: 274.0980, found 274.0981.

4-Amino-3-(phenylbuta-1,3-diyn-1-yl)benzonitrile (S17)

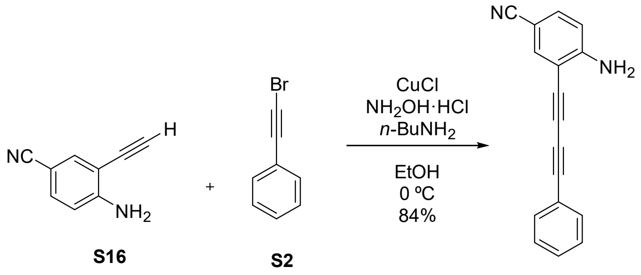

By a procedure identical with that described for the preparation of **S3**, **S16** (325 mg, 2.29 mmol) was converted to **S17** (463 mg, 84%) by the reaction with **S2** (538 mg, 2.97 mmol): white solid: mp 181–182 ºC; IR (neat) 3473, 3355, 2216, 2127, 2097; ^{1}H NMR (500 MHz, $CDCl_3$) δ: 4.83 (br s, 2H), 6.70 (d, J = 8.6 Hz, 1H), 7.34-7.41 (m, 4H), 7.54 (dd, J = 8.6, 1.7 Hz, 2H), 7.62 (d, J = 1.7 Hz, 1H); ^{13}C NMR (125 MHz, $CDCl_3$) δ: 73.2, 75.6, 80.5, 83.8, 100.4, 114.1, 119.0, 121.3, 128.5 (3C), 129.6, 132.5 (2C), 133.9, 137.4, 152.4; HRMS (FAB^+) calcd for $C_{17}H_{11}N_2$ $[M+H]^+$: 243.0922, found 243.0919.

4-Azido-3-(phenylbuta-1,3-diyn-1-yl)benzonitrile (1h)

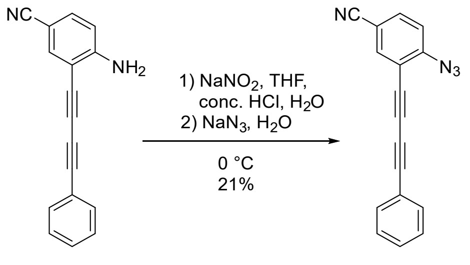

By a procedure identical with that described for the preparation of **1a**, **S17** (242 mg, 1.00 mmol) was converted to **1h** (56.4 mg, 21%): orange powder; mp 138–140 ºC; IR (neat) 2226, 2122, 2084; ^{1}H NMR (500 MHz, $CDCl_3$) δ: 7.23 (d, *J* = 8.6 Hz, 1H), 7.35-7.43 (m, 3H), 7.54-7.56 (m, 2H), 7.63 (dd, *J* = 8.3, 1.7 Hz, 1H), 7.77 (d, *J* = 1.7 Hz, 1H); ^{13}C NMR (125 MHz, $CDCl_3$) δ: 73.0, 74.3, 81.6, 84.7, 108.5, 115.3, 117.4, 119.4, 121.1, 128.5 (2C), 129.8, 132.6 (2C), 133.2, 137.9, 146.9; HRMS (FAB) calcd for $C_{17}H_9N_4$ $[M+H]^+$: 269.0827, found 269.0826.

4-Methoxy-2-(phenylbuta-1,3-diyn-1-yl)aniline (S19)

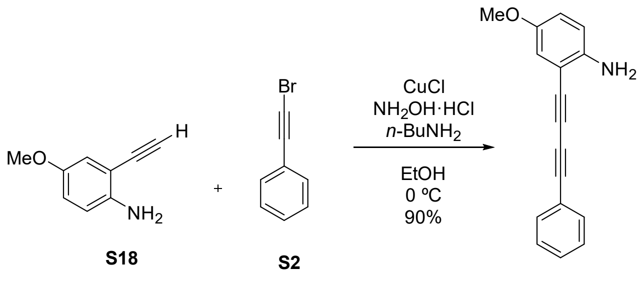

By a procedure identical with that described for the preparation of **S3**, **S18** (452 mg, 3.07 mmol) was converted to **S19** (680 mg, 90%) by the reaction with **S2** (723 mg, 3.99 mmol): yellow crystals; mp 85–87 ºC; IR (neat) 3456, 3367, 2209, 2125; ^{1}H NMR (500 MHz, $CDCl_3$) δ: 3.73 (s, 3H), 4.05 (s, 2H), 6.65 (d, *J* = 8.7 Hz, 1H), 6.80 (dd, *J* = 8.7, 2.9 Hz, 1H), 6.87 (d, *J* = 2.9 Hz, 1H), 7.32-7.39 (m, 3H), 7.52 (dd, *J* = 7.9, 2.4 Hz, 2H); ^{13}C NMR (125 MHz, $CDCl_3$) δ: 55.8, 73.8, 78.6, 79.0, 82.8, 106.6, 116.1, 118.8, 121.8, 128.4 (3C), 129.2, 132.4 (2C), 144.0, 151.7; HRMS (ESI) calcd for $C_{17}H_{14}NO$ $[M+H]^+$: 248.1075, found 248.1088.

1-Azido-4-methoxy-2-(phenylbuta-1,3-diyn-1-yl)benzene (1i)

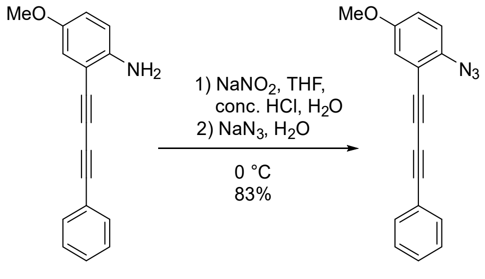

By a procedure identical with that described for the preparation of **1a**, **S19** (495 mg, 2.00 mmol) was converted to **1i** (454 mg, 83%): pale yellow solid; mp 75–77 ºC; IR (neat) 2121, 2088; ^{1}H NMR (500 MHz, $CDCl_3$) δ: 3.79 (s, 3H), 6.95 (dd, *J* = 8.6, 2.9 Hz, 1H), 7.01 (d, *J* = 2.9 Hz, 1H), 7.05 (d, *J* = 8.6 Hz, 1H), 7.32-7.40 (m, 3H), 7.52-7.55

(m, 2H); ^{13}C NMR (125 MHz, $CDCl_3$) δ: 55.7, 73.5, 76.9, 79.1, 83.4, 114.4, 117.6, 118.1, 119.7, 121.5, 128.4 (2C), 129.4, 132.5 (2C), 135.3, 156.3; HRMS (FAB) calcd for $C_{17}H_{12}N_3$ $[M+H]^+$: 274.0980, found 274.0981.

Diethyl 1*H*-Indole-1,5-dicarboxylate (10F)

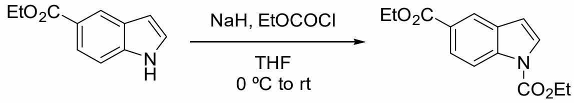

To a suspension of NaH (60% dispersion in paraffin liquid; 160 mg, 4.00 mmol) in THF (2.0 mL) at 0 ºC was added **S20** (378 mg, 2.00 mmol), and the mixture was stirred at 0 °C for 10 min under Ar. To the reaction mixture was added dropwise $ClCO_2Et$ (0.286 mL, 3.00 mmol). After being stirred at room temperature for 2 h, the reaction mixture was diluted with H_2O and extracted with EtOAc. The combined organic layer was dried over Na_2SO_4 and filtered and concentrated in vacuo. The residue was purified by column chromatography (hexane/EtOAc = 30/1) to give **10F** (453 mg, 87%) as a white solid: mp 73–74 ºC; IR (neat) 1742, 1710; ^{1}H NMR (500 MHz, $CDCl_3$) δ: 1.42 (t, J = 7.2 Hz, 3H), 1.48 (t, J = 7.2 Hz, 3H), 4.40 (q, J = 7.2 Hz, 2H), 4.51 (q, J = 7.2 Hz, 2H), 6.67 (d, J = 3.5 Hz, 1H), 7.67 (d, J = 3.5 Hz, 1H), 8.04 (dd, J = 8.7, 1.7 Hz, 1H), 8.21 (d, J = 9.3 Hz, 1H), 8.30-8.31 (m, 1H); ^{13}C NMR (125 MHz, $CDCl_3$) δ: 14.3 (2C), 60.8, 63.5, 108.3, 114.7, 123.1, 125.2, 125.7, 126.7, 130.2, 137.8, 150.7, 166.9; HRMS (ESI) calcd for $C_{14}H_{16}NO_4$ $[M+H]^+$: 262.1079, found 262.1066.

3. Gold-Catalysed Cascade Cyclisation

General Procedure for Synthesis of Benzo[*c*]carbazole: Synthesis of 3-Methoxy-5-phenyl-7*H*-benzo[*c*]carbazole (3aA)

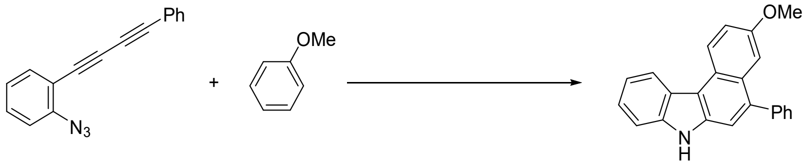

Condition A (Table 1, Entry 10): To a solution of **1a** (48.6 mg, 0.200 mmol) and **8A** (216 mg, 2.00 mmol) in DCE (1.00 mL) was added [JohnPhosAu(MeCN)SbF_6] (7.70 mg, 10.0 μmol) at 80 °C. The mixture was stirred at 80 °C in pre-heated bath for 1 h. After disappearance of **1a** on TLC, the reaction mixture was allowed to warm to 140 °C and stirred at this temperature for 16 h. The mixture was concentrated in vacuo. The residue was purified by column chromatography on silica gel (hexane/EtOAc = 20/1) to give **3aA** (48.3 mg, 75%).

Condition B (Table 1, Entry 12): To a solution of **1a** (48.6 mg, 0.200 mmol) in anisole (**8A**) (1.00 mL) was added [BrettPhosAu(MeCN)SbF_6] (10.1 mg, 10.0 μmol) at 140 °C. The mixture was stirred at 140 °C in pre-heated bath for 19.5 h and concentrated in vacuo. The residue was purified by column chromatography on silica gel (hexane/EtOAc = 20/1) to give **3aA** (55.6 mg, 86%) as a white solid. Recrystallization from EtOAc–hexane gave pure **3aA**.

Compound **3aA**: colorless crystals; mp 168–172 °C; IR (neat): 3404; ^{1}H NMR (500 MHz, $CDCl_3$) δ: 3.80 (s, 3H), 7.37-7.42 (m, 3H), 7.44-7.47 (m, 2H), 7.51-7.60 (m, 6H), 8.39 (s, 1H), 8.55 (d, J = 8.0 Hz, 1H), 8.78 (d, J = 9.7 Hz, 1H); ^{13}C NMR (125 MHz, $CDCl_3$) δ: 55.3, 107.6, 111.1, 114.0, 115.3, 117.8, 120.1, 121.9, 123.6, 124.3, 124.9,

125.3, 127.3, 128.3 (2C), 128.9, 130.1 (2C), 135.5, 138.6, 138.7, 141.4, 155.4; HRMS (ESI) calcd for $C_{23}H_{18}NO$ $[M+H]^+$ 324.1383, found 324.1379.

3-(4-Methoxyphenyl)-2-(phenylethynyl)-1*H*-indole (2aA) (Table 1, Entry 4).

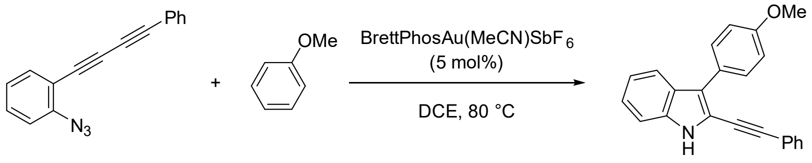

1a **8A** **2aA**

To a solution of **1a** (48.6 mg, 0.200 mmol) and anisole (**8A**) (216 mg, 2.00 mmol) in 1,2-DCE (1.00 mL) was added [BrettPhosAu(MeCN)SbF_6] (10.1 mg, 10.0 μmol) at 80 °C. The mixture was stirred at 80 °C in pre-heated bath for 30 h and concentrated in vacuo. The residue was purified by column chromatography on silica gel (hexane/EtOAc = 20/1) to give **2aA** as brown solid (42.1 mg, 65%). Recrystallization from EtOAc–hexane gave pure **2aA**: colorless crystals; mp 97–107 °C; IR (neat): 3409, 2205; ^{1}H NMR (500 MHz, $CDCl_3$) δ: 3.89 (s, 3H), 7.06 (d, J = 8.6 Hz, 2H), 7.17 (dd, J = 7.4, 7.4 Hz, 1H), 7.28 (dd, J = 7.4, 7.4 Hz, 1H), 7.34-7.37 (m, 4H), 7.47-7.49 (m, 2H), 7.80 (d, J = 8.6 Hz, 2H), 7.82 (d, J = 8.0 Hz, 1H), 8.26 (s, 1H); ^{13}C NMR (125 MHz, $CDCl_3$) δ: 55.3, 82.3, 94.3, 110.9, 114.0 (2C), 115.5, 120.1, 120.6, 122.4, 122.7, 123.9, 126.1, 126.7, 128.4 (3C), 130.0 (2C), 131.3 (2C), 136.0, 158.4; HRMS (ESI) calcd for $C_{23}H_{18}NO$ $[M+H]^+$ 324.1383, found 324.1378.

2,3-Dimethoxy-5-phenyl-7*H*-benzo[*c*]carbazole (3aB).

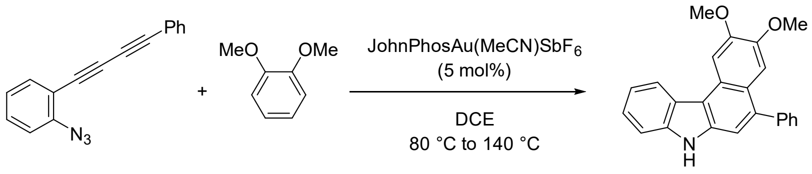

1a **8B** **3aB**

According to the general procedure described for the preparation of **3aA**, **1a** (48.6 mg, 0.200 mmol) was converted into **3aB** (49.9 mg, 70%) (condition A; 80 °C, 2 h then 140 °C, 15 h). The reaction of **1a** (48.6 mg, 0.200 mmol) and **2B** (1.00 mL) under condition B (140 °C, 27 h) gave **3aB** (70 mg, quant) as a white solid. Recrystallization from EtOAc–hexane gave pure **3aB**: colorless crystals; mp 245–257 °C; IR (neat): 3332; ^{1}H NMR (500 MHz, $CDCl_3$) δ: 3.82 (s, 3H), 4.20 (s, 3H), 7.38-7.40 (m 3H), 7.44 (dd, J = 7.4 Hz, 2H), 7.50-7.57 (m, 5H), 8.13 (s, 1H), 8.34 (s, 1H), 8.46 (d, J = 8.0 Hz, 1H); ^{13}C NMR (125 MHz, $CDCl_3$) δ: 55.7, 55.9, 103.3, 107.3, 111.1, 111.5, 114.7, 120.0, 121.4, 122.6, 123.6, 124.2, 125.7, 127.2, 128.3 (2C), 130.1 (2C), 136.2, 138.4, 138.8, 141.7, 146.7, 149.9; HRMS (ESI) calcd for $C_{24}H_{20}NO_2$ $[M+H]^+$ 354.1489, found 354.1489.

1,3-Dimethoxy-5-phenyl-7*H*-benzo[*c*]carbazole (3aC).

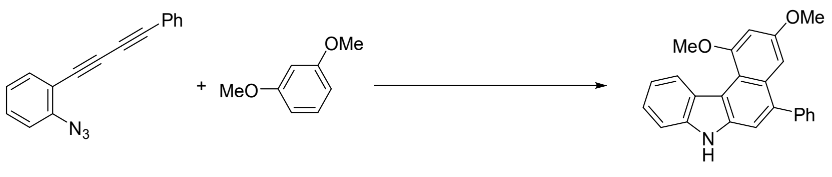

1a **8C** **3aC**

According to the general procedure (condition A; 80 °C, 2 h then 140 °C, 15 h) described for the preparation of **3aA**, **1a** (48.6 mg, 0.200 mmol) was converted into **3aC** (28.3 mg, 40%). The reaction of **1a** (48.6 mg, 0.200 mmol) and **8C** (1.00 mL) under the condition B (140 °C, 26 h) gave **3aC** (67.3 mg, 95%) as a brown solid. Recrystallization from EtOAc–hexane gave pure **3aC**: colorless crystals; mp 205–210 °C; IR (neat): 3396; ^{1}H NMR (400 MHz, $CDCl_3$) δ: 3.73 (s, 3H), 4.14 (s, 3H), 6.77 (d, J = 2.3 Hz, 1H), 6.94 (d, J = 2.3 Hz, 1H), 7.25-7.29 (dd, 1H), 7.36-7.51 (m, 8H), 8.29 (s, 1H), 8.87 (d, J = 8.1 Hz, 1H); ^{13}C NMR (125 MHz, $CDCl_3$) δ: 55.1, 55.2, 98.4, 99.5, 110.5, 114.4, 114.7, 118.0, 119.3, 124.3, 124.3, 126.7, 127.2, 128.2 (2C), 130.0 (2C), 130.2, 136.0, 138.4, 139.1, 141.9, 155.6, 156.9; HRMS (ESI) calcd for $C_{24}H_{20}NO_2$ [M+H]$^+$ 354.1489, found 354.1490.

5-Phenyl-7*H*-[1,3]dioxolo[4',5':4,5]benzo[1,2-*c*]carbazole (3aD).

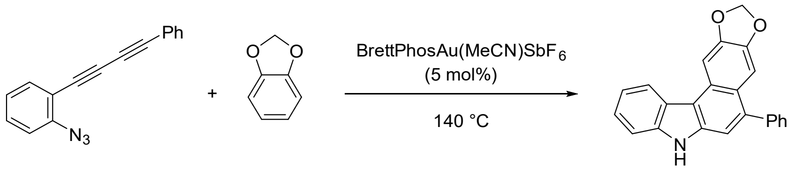

1a **8D** **3aD**

According to the general procedure (condition B; 140 °C, 18 h) described for the preparation of **3aA**, **1a** (48.6 mg, 0.200 mmol) was converted into **3aD** (51.1 mg, 76%) as a white solid. Recrystallization from EtOAc–hexane gave pure **3aD**: colorless crystals; mp 192–198 °C; IR (neat): 3411; ^{1}H NMR (500 MHz, $CDCl_3$) δ: 6.07 (s, 2H), 7.31 (s, 1H), 7.37 (dd, J = 7.7, 7.7 Hz, 1H), 7.41 (s, 1H), 7.43-7.46 (m, 2H), 7.48-7.55 (m, 5H), 8.18 (s, 1H), 8.30 (s, 1H), 8.47 (d, J = 8.0 Hz, 1H); ^{13}C NMR (125 MHz, $CDCl_3$) δ: 100.9, 101.1, 104.6, 111.0, 111.5, 115.4, 120.1, 121.6, 123.7, 123.9, 124.3, 126.8, 127.2, 128.3 (2C), 130.1 (2C), 136.2, 138.8, 139.0, 141.6, 145.2, 148.1; HRMS (ESI) calcd for $C_{23}H_{16}NO_2$ [M+H]$^+$ 338.1176, found 338.1173.

2,3-Dimethyl-5-phenyl-7*H*-benzo[*c*]carbazole (3aE).

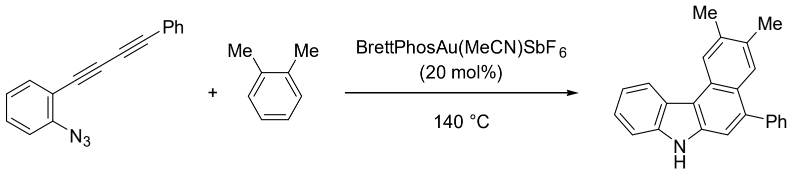

1a **8E** **3aE**

According to the general procedure (condition B; 140 °C, 1 h) described for the preparation of **3aA**, **1a** (48.6 mg, 0.200 mmol) was converted into **3aE** (26.7 mg, 42%) as a white solid. In the case, the reaction was conducted using 20 mol % BrettPhosAu(MeCN)SbF_6 (40.4 mg, 0.04 mmol). Recrystallization from EtOAc–hexane gave pure **3aE**: colorless crystals; mp 232–242 °C; IR (neat): 3422; ^{1}H NMR (500 MHz, $CDCl_3$) δ: 2.38 (s, 3H), 2.58 (s, 3H), 7.37-7.40 (m, 1H), 7.42-7.47 (m, 3H), 7.50-7.58 (m, 5H), 7.73 (s, 1H), 8.29 (s, 1H), 8.60 (d, J = 7.4 Hz, 2H); ^{13}C NMR (125 MHz, $CDCl_3$) δ: 20.3, 20.7, 110.0, 112.7, 114.5, 120.0, 122.0, 123.5, 124.0, 124.1, 126.5, 127.1 (2C), 128.2 (2C), 128.9, 130.3 (2C), 132.3, 136.3, 136.4, 138.6, 139.0, 141.6; HRMS (ESI) calcd for $C_{24}H_{20}N$ [M+H]$^+$ 322.1590, found 322.1589.

3-Methoxy-5-(*o*-tolyl)-7*H*-benzo[*c*]carbazole (3bA)

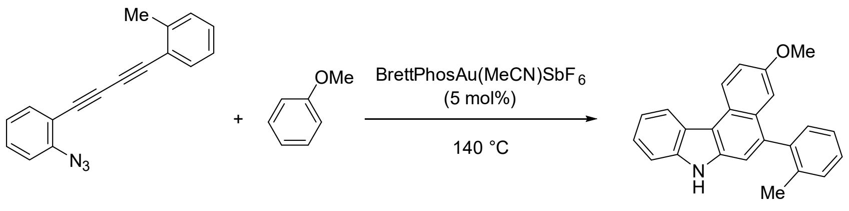

1b **8A** **3bA**

According to the general procedure (condition B; 140 °C, 20 h) described for the preparation of **3aA**, **1d** (51.4 mg, 0.200 mmol) was converted into **3bA** (60.3 mg, 89%) as a colorless oil: IR (neat): 3407; ^{1}H NMR (500 MHz, $CDCl_3$) δ: 2.05 (s, 3H), 3.73 (s, 3H), 6.93 (d, *J* = 2.9 Hz, 1H), 7.31-7.32 (m, 2H), 7.36-7.39 (m, 4H), 7.41 (s, 1H), 7.42-7.45 (m, 1H), 7.53 (d, *J* = 8.0 Hz, 1H), 8.28 (s, 1H), 8.54 (d, *J* = 7.4 Hz, 1H), 8.76 (d, *J* = 9.2 Hz, 1H); ^{13}C NMR (125 MHz, $CDCl_3$) δ: 20.1, 55.2, 107.4, 111.1, 113.7, 115.2, 117.7, 120.1, 121.8, 123.7, 124.3, 124.8, 125.0, 125.7, 127.7, 129.2, 129.9, 130.4, 135.5, 137.0, 138.2, 138.6, 140.7, 155.5; HRMS (ESI) calcd for $C_{24}H_{20}NO$ $[M+H]^+$ 338.1539, found 338.1538.

3-Methoxy-5-(*m*-tolyl)-7*H*-benzo[*c*]carbazole (3cA)

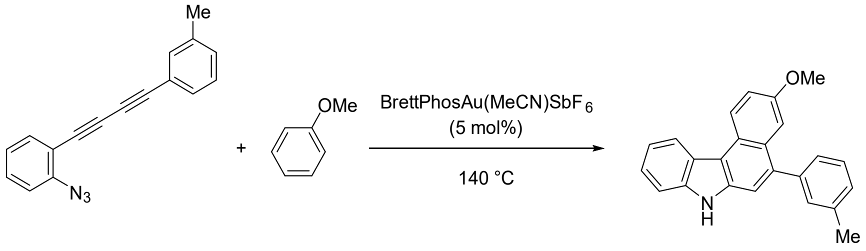

1c **8A** **3cA**

According to the general procedure (condition B; 140 °C, 20 h) described for the preparation of **3aA**, **1c** (51.4 mg, 0.200 mmol) was converted into **3cA** (63.6 mg, 94%) as a colorless oil: IR (neat): 3411; ^{1}H NMR (500 MHz, $CDCl_3$) δ: 2.46 (s, 3H), 3.80 (s, 3H), 7.27 (d, *J* = 7.4 Hz, 1H), 7.36-7.39 (m, 4H), 7.41-7.42 (m, 2H), 7.44 (dd, *J* = 7.4, 7.4 Hz, 1H), 7.53 (s, 1H), 7.55 (d, *J* = 8.0 Hz, 1H), 8.33 (s, 1H), 8.54 (d, *J* = 7.4 Hz, 1H), 8.77 (d, *J* = 9.2 Hz, 1H); ^{13}C NMR (125 MHz, $CDCl_3$) δ: 21.5, 55.3, 107.7, 111.1, 113.9, 115.2, 117.8, 120.1, 121.9, 123.7, 124.3, 124.8, 125.3, 127.2, 128.0, 128.2, 128.9, 130.8, 135.5, 138.0, 138.7, 138.8, 141.4, 155.4; HRMS (ESI) calcd for $C_{24}H_{20}NO$ $[M+H]^+$ 338.1539, found 338.1538.

3-Methoxy-5-(*p*-tolyl)-7*H*-benzo[*c*]carbazole (3dA)

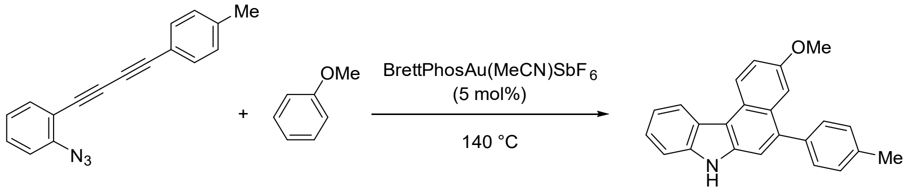

1d **8A** **3dA**

According to the general procedure (condition B; 140 °C, 20 h) described for the preparation of **3aA**, **1b** (51.4 mg, 0.200 mmol) was converted into **3dA** (47.0 mg, 70%). Recrystallization from EtOAc–hexane gave pure **3dA**: colorless crystals; mp 162–170 °C; IR (neat): 3407; ^{1}H NMR (500 MHz, $CDCl_3$) δ: 2.47 (s, 3H), 3.79 (s, 3H), 7.30

(d, J = 8.0 Hz, 2H), 7.34-7.40 (m, 2H), 7.41-7.46 (m, 5H), 7.48 (d, J = 8.0 Hz, 1H), 8.22 (s, 1H) 8.52 (d, J = 8.0 Hz, 1H), 8.75 (d J = 9.2 Hz, 1H); ^{13}C NMR (125 MHz, $CDCl_3$) δ: 21.3, 55.3, 107.6, 111.1, 114.0, 117.8, 120.0, 121.9, 123.6, 124.3, 124.8, 125.2, 128.9, 129.0 (2C), 130.0 (2C), 130.1, 135.5, 136.9, 138.4, 138.6, 138.7, 155.4; HRMS (ESI) calcd for $C_{24}H_{20}NO$ $[M+H]^+$ 338.1539, found 338.1539.

5-(4-chlorophenyl)-3-methoxy-7*H*-benzo[*c*]carbazole (3eA)

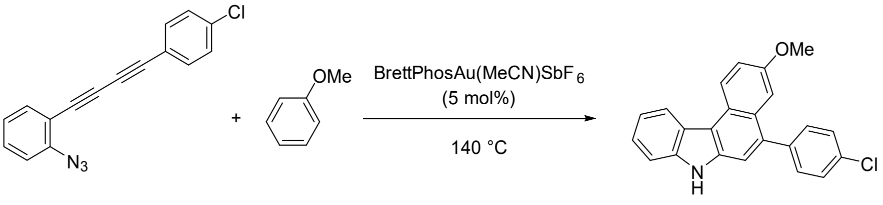

1e **8A** **3eA**

According to the general procedure (condition B; 140 °C, 22.5 h) described for the preparation of **3aA**, **1e** (55.5 mg, 0.200 mmol) was converted into **3eA** (53.5 mg, 74%). Recrystallization from EtOAc–hexane gave pure **3eA**: colorless crystals; mp 170–177 °C; IR (neat): 3406; ^{1}H NMR (500 MHz, $CDCl_3$) δ: 3.11 (s, 3H), 7.30 (d, J = 2.9 Hz, 1H), 7.36-7.40 (m, 3H), 7.43-7.51 (m, 6H), 8.23 (s, 1H), 8.52 (d, J = 7.4 Hz, 1H), 8.76 (d, J = 9.2 Hz, 1H); ^{13}C NMR (125 MHz, $CDCl_3$) δ: 55.3, 107.1, 111.1, 114.0, 115.5, 118.0, 120.2, 121.9, 123.5, 124.5, 124.9, 125.2, 128.5 (2C), 128.6, 131.4 (2C), 133.2, 135.3, 137.1, 138.7, 139.8, 155.5; HRMS (ESI) calcd for $C_{23}H_{17}ClNO$ $[M+H]^+$ 358.0993, found 358.0991.

3-Methoxy-5-(4-nitrophenyl)-7*H*-benzo[*c*]carbazole (3fA)

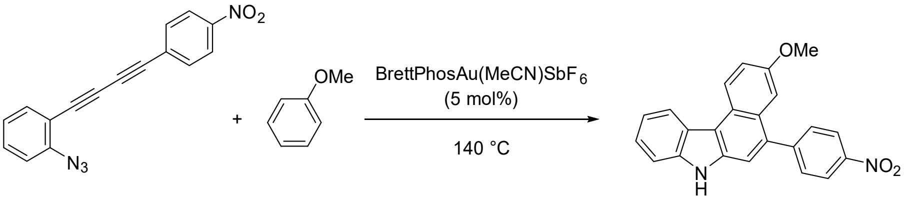

1f **8A** **3fA**

According to the general procedure (condition B; 140 °C, 23 h) described for the preparation of **3aA**, **1f** (57.6 mg, 0.200 mmol) was converted into **3fA** (32.0 mg, 43%). Recrystallization from EtOAc–hexane gave pure **3fA**: colorless crystals; mp 244–248 °C; IR (neat): 3343, 1498, 1344; ^{1}H NMR (600 MHz, $CDCl_3$) δ: 3.82 (s, 3H), 7.39-7.45 (m, 3H), 7.49 (dd, J = 7.4 Hz, 1H), 7.57 (s, 1H), 7.60 (d, J = 7.7 Hz, 1H), 7.77 (d, J = 8.8 Hz, 2H), 8.40 (d, J = 8.8 Hz, 2H), 8.43 (s, 1H), 8.56 (d, J = 7.7 Hz, 1H), 8.81 (d, J = 8.8 Hz, 1H); ^{13}C NMR (125 MHz, $CDCl_3$) δ: 55.4, 106.8, 111.3, 114.3, 116.5, 118.3, 120.4, 122.2, 123.4, 123.7 (2C), 125.0, 125.2, 125.4, 128.1, 131.0 (2C), 135.2, 135.8, 139.0, 147.2, 148.6, 155.9; HRMS (ESI) calcd for $C_{23}H_{17}N_2O_3$ ($[M–H]^-$) 367.1088, found 367.1088.

3-Methoxy-5-(4-methoxyphenyl)-7*H*-benzo[*c*]carbazole (3gA)

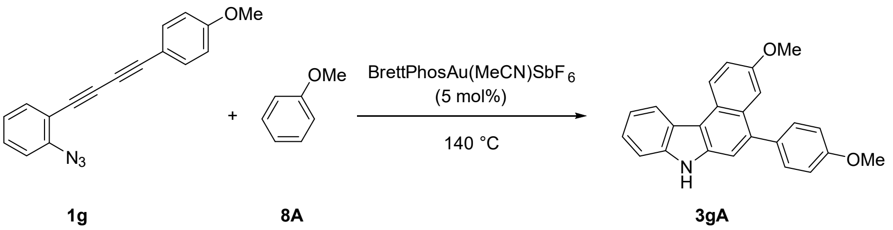

1g **8A** **3gA**

According to the general procedure (condition B; 140 °C, 12 h) described for the preparation of **3aA**, **1g** (54.6 mg, 0.200 mmol) was converted into **3gA** (42.8 mg, 61%) as a brown solid: mp 175–185 °C; IR (neat): 3406; ^{1}H NMR (500 MHz, $CDCl_3$) δ: 3.78 (s, 3H), 3.88 (s, 3H), 7.00 (d, J = 8.6 Hz, 2H), 7.33-7.45 (m, 8H), 8.19 (s, 1H), 8.51 (d, J = 8.0 Hz, 1H), 8.74 (d, J = 8.6 Hz, 1H); ^{13}C NMR (125 MHz, $CDCl_3$) δ: 55.3, 55.3, 107.5, 111.1, 113.7 (2C), 114.0, 115.0, 117.8, 120.0, 121.8, 123.6, 124.2, 124.8, 125.2, 129.0, 131.1 (2C), 133.7, 135.5, 138.2, 138.7, 155.4, 158.8; HRMS (ESI) calcd for $C_{24}H_{20}NO_2$ $[M+H]^+$ 354.1489, found 354.1489.

4-Phenyl-3,6-dihydropyrrolo[2,3-*c*]carbazole (6aA) and Its [3,2-*c*]-Isomer (7aA) (Table 3, Entry 1)[1]

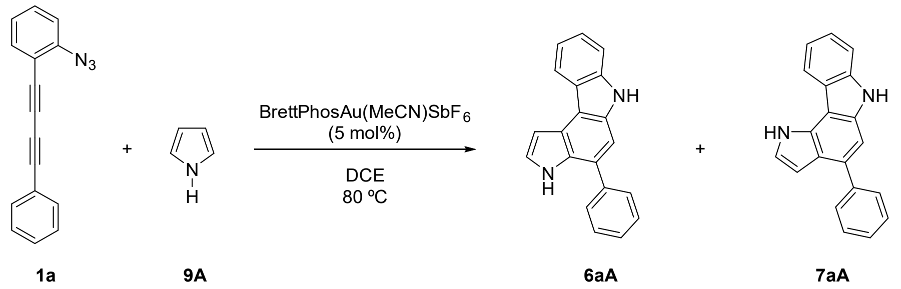

To a solution of **1a** (24.3 mg, 0.100 mmol) and **9A** (0.0347 mL, 0.5 mmol) in DCE (0.5 mL) was added [BrettPhosAu(MeCN)SbF_6] (5.05 mg, 5.00 μmol) at 80 ºC. The mixture was stirred at 80 ºC in pre-heated bath for 8 h and concentrated in vacuo. The residue was purified by column chromatography (hexane/EtOAc = 5/1) to give a mixture of **6aA** and **7aA** containing a small amount of impurities (17.6 mg, <62%; **6aA**:**7aA** = 25:75). These isomers were separated by column chromatography on amine silica gel (hexane/$CHCl_3$ = 2/1) to give, in the order of elution, **6aA** and **7aA**.

Compound **6aA**: brown viscous oil; IR (neat) 3406; ^{1}H NMR (500 MHz, $CDCl_3$) δ: 7.15 (dd, J = 2.5, 1.5 Hz, 1H), 7.24 (s, 1H), 7.28-7.32 (m, 1H), 7.35 (dd, J = 3.0, 3.0 Hz, 1H), 7.38-7.45 (m, 3H), 7.52 (dd, J = 8.0, 8.0 Hz, 2H), 7.66-7.69 (m, 2H), 8.04 (s, 1H), 8.28 (d, J = 7.0 Hz, 1H), 8.54 (s, 1H); ^{13}C NMR (125 MHz, $CDCl_3$) δ: 101.4, 106.2, 110.5, 113.7, 119.2, 121.1, 121.3, 123.6, 124.18, 124.23, 125.0, 127.4, 128.5 (2C), 128.9, 129.2 (2C), 134.9, 139.1, 139.7; HRMS (ESI) calcd for $C_{20}H_{15}N_2$ $[M+H]^+$: 283.1230, found 283.1231.

Compound **7aA**: white solid; mp 231–234 ºC; IR (neat) 3395; ^{1}H NMR (500 MHz, $CDCl_3$) δ: 6.87 (dd, J = 3.5, 2.5 Hz, 1H), 7.31-7.34 (m, 3H), 7.39-7.44 (m, 2H), 7.51-7.54 (m, 3H), 7.78-7.79 (m, 2H), 8.08 (d, J = 6.5 Hz, 1H), 8.27 (s, 1H), 8.79 (s, 1H); ^{13}C NMR (125 MHz, $CDCl_3$) δ: 103.4, 104.6, 106.8, 110.7, 119.5, 120.2, 120.3, 121.7 (2C), 124.3, 127.0, 128.5 (2C), 129.1 (2C), 130.2, 133.7, 137.1, 138.7, 141.6; HRMS (ESI) calcd for $C_{20}H_{15}N_2$ $[M+H]^+$: 283.1230, found 283.1231.

3-Benzyl-4-phenyl-3,6-dihydropyrrolo[2,3-*c*]carbazole (6aB) and Its [3,2-*c*]-Isomer (7aB) (Table 3, Entry 2)[1]

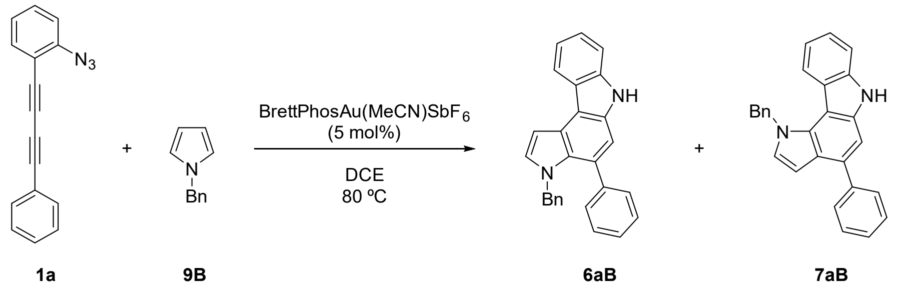

To a solution of **1a** (24.3 mg, 0.100 mmol) and **9B** (78.6 mg, 0.500 mmol) in DCE (0.5 mL) was added [BrettPhosAu(MeCN)SbF_6] (5.05 mg, 5.00 μmol) at 80 ºC. The mixture was stirred at 80 ºC in pre-heated bath for 10 h and concentrated in vacuo. The residue was purified by column chromatography (hexane/EtOAc = 10/1) to give a **6aB** (4.20 mg, 11%) and **7aB** (19.0 mg, 51%) in the order of elution, **6aB** and **7aB**; (**6aB**:**7aB** = 18:82).

Compound **6aB**: yellow solid; mp 163–168 ºC; IR (neat) 3728; ^{1}H NMR (500 MHz, $CDCl_3$) δ: 5.00 (s, 2H), 6.47 (m, 2H), 7.05 (s, 1H), 7.06-7.13 (m, 3H), 7.20 (d, J = 3.0 Hz, 1H), 7.22-7.28 (m, 5H), 7.30-7.35 (m, 2H), 7.41 (dt, J = 8.0, 1.0 Hz, 1H), 7.48 (d, J = 8.0 Hz, 1H), 8.06 (s, 1H), 8.30 (d, J = 8.0 Hz, 1H); ^{13}C NMR (125 MHz, $CDCl_3$) δ: 52.2, 100.2, 108.7, 110.5, 113.8, 119.2, 121.2, 123.4, 123.7, 124.2, 125.8 (2C), 126.6, 126.9, 127.1, 127.6 (2C), 128.2 (2C), 128.7, 129.9 (2C), 130.8, 134.0, 138.8, 139.2, 140.6; HRMS (ESI) calcd for $C_{27}H_{21}N_2$ $[M+H]^+$: 373.1699, found 373.1700.

Compound **7aB**: white solid; mp 173–178 ºC; IR (neat) 3728; ^{1}H NMR (500 MHz, $CDCl_3$) δ: 6.02 (s, 2H), 6.84 (d, J = 3.0 Hz, 1H), 7.00-7.04 (m, 2H), 7.24-7.33 (m, 7H), 7.39-7.42 (m, 2H), 7.50 (t, J = 8.0 Hz, 2H), 7.75 (d, J = 7.5 Hz, 2H), 8.01 (d, J = 8.5 Hz, 1H), 8.23 (s, 1H); ^{13}C NMR (125 MHz, $CDCl_3$) δ: 53.2, 103.6, 104.8, 106.9, 110.6, 119.3, 121.0, 121.7, 122.6, 123.9, 126.5 (2C), 126.6, 127.0, 127.5, 128.4 (2C), 128.9 (2C), 129.2 (2C), 132.6, 134.1, 138.0, 138.7 (2C), 141.3; HRMS (ESI) calcd for $C_{27}H_{21}N_2$ $[M+H]^+$: 373.1699, found 373.1702.

4-Phenyl-3-tosyl-3,6-dihydropyrrolo[2,3-*c*]carbazole (6aC) and Its [3,2-*c*]-Isomer (7aC) (Table 3, Entry 3)[1]

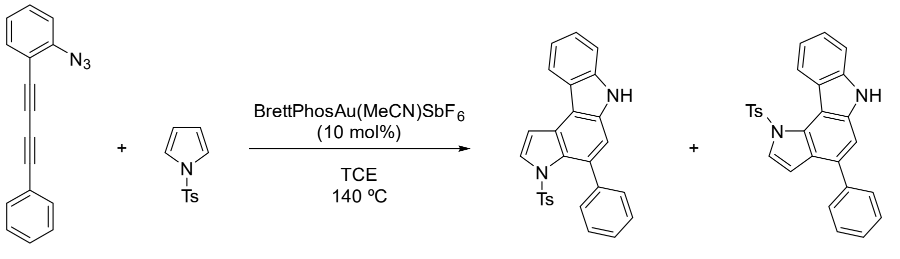

To a solution of **1a** (24.3 mg, 0.100 mmol) and **9C** (111 mg, 0.500 mmol) in TCE (0.5 mL) was added [BrettPhosAu(MeCN)SbF_6] (10.1 mg, 10.0 μmol) at 140 ºC. The mixture was stirred at 140 ºC in pre-heated bath for 30 min and concentrated in vacuo. The residue was purified by column chromatography (hexane/EtOAc = 5/1) to give a mixture of **6aC** and **7aC** (14.7 mg, 34%; **6aC**:**7aC** = 58:42). These isomers were separated by column chromatography on silica gel (toluene) to give, in the order of elution, **6aC** and **7aC**.

Compound **6aC**: brown viscous oil; IR (neat) 3736; ^{1}H NMR (500 MHz, $CDCl_3$) δ: 2.24 (s, 3H), 6.98 (d, J = 8.5 Hz, 2H), 7.18-7.21 (m, 3H), 7.27-7.31 (m, 2H), 7.37-7.47 (m, 7H), 7.80 (d, J = 4.0 Hz, 1H), 8.13-8.14 (m, 2H); ^{13}C NMR (125 MHz, $CDCl_3$) δ: 21.5, 110.0, 110.8, 111.6, 114.1, 119.8, 121.1, 122.8, 125.4, 126.5 (2C), 127.0, 127.4, 127.7 (2C), 128.9, 129.1 (2C), 129.3 (2C), 130.2, 132.0, 134.4, 136.6, 139.6, 141.8, 144.0; HRMS (ESI) calcd for $C_{27}H_{21}N_2O_2S$ $[M+H]^+$: 437.1318, found 437.1318.

Compound **7aC**: white viscous oil; IR (neat) 3390; ^{1}H NMR (500 MHz, $CDCl_3$) δ: 2.24 (s, 3H), 6.98 (d, J = 8.6 Hz, 2H), 7.19 (t, J = 7.2 Hz, 3H), 7.29 (dt, J = 12.4, 3.3 Hz, 2H), 7.37-7.39 (m, 3H), 7.42 (t, J = 7.4 Hz, 1H), 7.45-7.47 (m, 3H), 7.80 (d, J = 4.0 Hz, 1H), 8.14 (d, J = 7.4 Hz, 2H); ^{13}C NMR (125 MHz, $CDCl_3$) δ: 21.5, 109.3, 110.0, 112.6, 114.2, 119.2, 121.7, 125.5, 125.7, 125.9, 126.8 (2C), 127.3, 128.3, 128.6 (2C), 128.9 (2C), 129.0 (2C), 132.4, 133.1, 133.6, 139.6, 139.7, 140.1, 144.3; HRMS (ESI) calcd for $C_{27}H_{21}N_2O_2S$ $[M+H]^+$: 437.1318, found 437.1317.

Methyl 4-Phenylpyrrolo[2,3-*c*]carbazole-3(6*H*)-carboxylate (6aD) and Its [3,2-*c*]-Isomer (7aD) (Table 3, Entry 4)

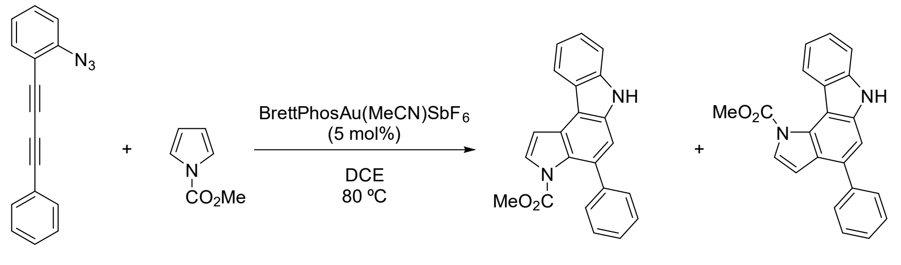

To a solution of **1a** (24.3 mg, 0.100 mmol) and **9D** (62.3 mg, 0.500 mmol) in DCE (0.5 mL) was added [BrettPhosAu(MeCN)SbF_6] (5.05 mg, 5.00 μmol) at 80 ºC. The mixture was stirred at 80 ºC in pre-heated bath for 1.5 h and concentrated in vacuo. The residue was purified by column chromatography (hexane/EtOAc = 5/1) to give a mixture of **6aD** and **7aD** (21.2 mg, 62%; **6aD**:**7aD** = 81:19). These isomers were separated by column chromatography on amine silica gel (hexane/$CHCl_3$ = 1/2) to give, in the order of elution, **6aD** and **7aD**.

Compound **6aD**: white powder; mp 173–175 ºC; IR (neat) 3380, 1751; ^{1}H NMR (500 MHz, $CDCl_3$) δ: 3.25 (s, 3H), 7.28 (d, *J* = 3.5 Hz, 1H), 7.31-7.37 (m, 3H), 7.42-7.53 (m, 6H), 7.80 (d, *J* = 3.4 Hz, 1H), 8.21 (s, 1H), 8.25 (d, *J* = 8.0 H, 1H); ^{13}C NMR (125 MHz, $CDCl_3$) δ: 53.2, 106.2, 110.2, 110.7, 114.1, 119.6, 121.3, 123.1, 125.1, 125.8, 126.6, 127.2, 127.4, 128.5 (2C), 128.7 (2C), 136.4, 139.6, 139.6, 143.1, 152.0; HRMS (ESI) calcd for $C_{22}H_{17}N_2O_2$ $[M+H]^+$: 341.1290, found 341.1284.

Compound **7aD**: orange viscous oil; IR (neat) 3398, 1734; ^{1}H NMR (500 MHz, $CDCl_3$) δ: 4.12 (s, 3H), 6.88 (d, *J* = 4.6 Hz, 1H), 7.27-7.30 (m, 1H), 7.41-7.47 (m, 3H), 7.49-7.53 (m, 3H), 7.63 (d, *J* = 3.5 Hz, 1H), 7.66 (d, *J* = 8.0 Hz, 2H), 7.94 (d, *J* = 8.0 Hz, 1H), 8.36 (s, 1H); ^{13}C NMR (125 MHz, $CDCl_3$) δ: 54.2, 107.8, 109.0, 110.2, 110.4, 119.2, 122.0, 123.3, 124.9, 125.3, 125.5, 127.2, 128.6 (2C), 129.2 (2C), 129.9, 133.4, 139.3, 139.5, 140.6, 152.2; HRMS (ESI) calcd for $C_{22}H_{17}N_2O_2$ $[M+H]^+$: 341.1290, found 341.1290

2,2-Dimethyl-1-(4-phenylpyrrolo[2,3-*c*]carbazol-3(6*H*)-yl)propan-1-one (6aE) (Table 3, Entry 5)

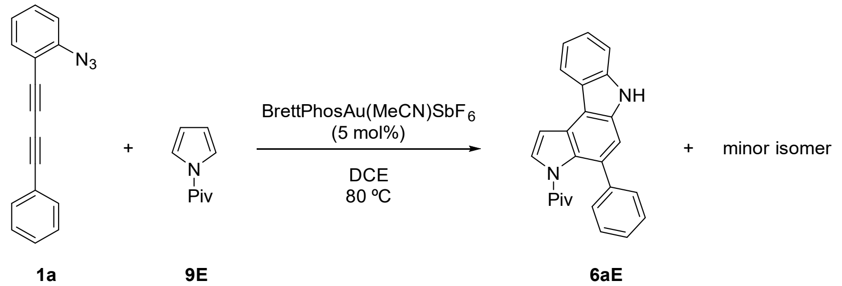

To a solution of **1a** (24.3 mg, 0.100 mmol) and **9E** (75.6 mg, 0.500 mmol) in DCE (0.5 mL) was added [BrettPhosAu(MeCN)SbF_6] (5.05 mg, 5.00 μmol) at 80 ºC. The mixture was stirred at 80 ºC in pre-heated bath for 1.5 h and concentrated in vacuo. The residue was purified by column chromatography (hexane/EtOAc = 5/1) to give an inseparable mixture of **6aE** and minor isomer (21.9 mg, 60%; **6aE**: minor isomer = 82:18): orange viscous oil; IR (neat) 3393, 1695; ^{1}H NMR (500 MHz, $CDCl_3$) δ: 1.37 (s, 9H), 7.23 (d, *J* = 3.4 Hz, 1H), 7.25 (s, 1H), 7.32 (dd, *J* = 7.2, 5.4 Hz, 2H), 7.42-7.49 (m, 6H), 7.74 (d, *J* = 4.0 Hz, 1H), 8.23 (d, *J* = 9.7 Hz, 2H); ^{13}C NMR (125 MHz, $CDCl_3$; including amide rotamers) δ: 28.8 and 29.3 (totally 3C), 41.6, 105.6, 107.9, 108.3, 109.9, 110.1, 119.1, 122.0, 122.5,

123.5, 124.6, 124.9, 125.7, 126.6, 127.1, 127.4, 128.5 (2C), 129.3 (2C), 131.3, 139.2, 140.8, 177.9; HRMS (ESI) calcd for $C_{25}H_{23}N_2O$ $[M+H]^+$: 367.1810, found 367.1801.

***tert*-Butyl 4-Phenylpyrrolo[2,3-*c*]carbazole-3(6*H*)-carboxylate (6aF) and Its [3,2-*c*]-Isomer (7aF) (Table 3, Entry 6)**[1]

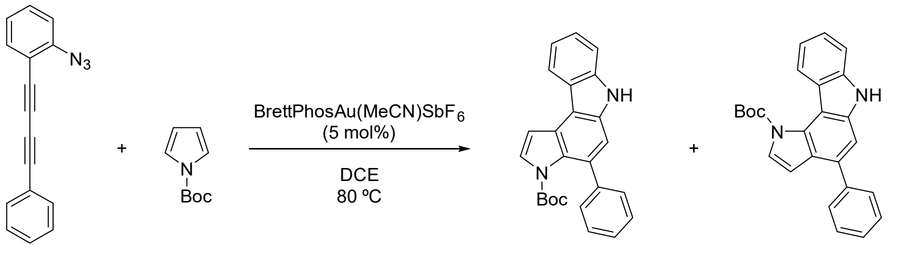

1a **9F** **6aF** **7aF**

To a solution of **1a** (24.3 mg, 0.100 mmol) and **9F** (83.6 mg, 0.500 mmol) in DCE (0.5 mL) was added [BrettPhosAu(MeCN)SbF_6] (5.05 mg, 5.00 μmol) at 80 ºC. The mixture was stirred at 80 ºC in pre-heated bath for 1.5 h and concentrated in vacuo. The residue was purified by column chromatography (hexane/EtOAc = 10/1) to give a mixture of **6aF** and **7aF** (23.1 mg, 60%; **6aF**:**7aF** = 92:8). These isomers were separated by reverse-column chromatography on silica gel (MeCN/0.1% TFA aq.) to give, in the order of elution, **6aF** and **7aF**.

Compound **6aF**: pale yellow solid; mp 173–178 ºC; IR (neat) 3397, 1742; ^{1}H NMR (500 MHz, $CDCl_3$) δ: 1.30 (s, 9H), 7.21 (d, J = 3.5 Hz, 1H), 7.29 (s, 1H), 7.32 (t, J = 8.0 Hz, 2H), 7.40-7.46 (m, 4H), 7.57 (d, J = 8.0 Hz, 2H), 7.73 (d, J = 4.0 Hz, 1H), 8.16 (s, 1H), 8.24 (d, J = 8.0 Hz, 1H); ^{13}C NMR (125 MHz, $CDCl_3$) δ: 27.6 (3C), 83.5, 105.5, 110.1, 110.7, 113.8, 119.5, 121.2, 123.2, 124.9, 125.9, 126.6, 127.4 (2C), 127.5, 128.5 (2C), 128.8, 129.1, 136.4, 139.5, 142.7, 149.6; HRMS (ESI) calcd for $C_{25}H_{23}N_2O_2$ $[M+H]^+$: 383.1754, found 383.1755.

Compound **7aF**: white viscous oil; IR (neat) 3592, 1747; ^{1}H NMR (500 MHz, $CDCl_3$) δ: 1.72 (s, 9H), 6.84 (d, J = 3.5 Hz, 1H), 7.28 (ddd, J = 8.0, 6.5, 1.5 Hz, 1H), 7.38-7.44 (m, 4H), 7.50 (t, J = 7.0 Hz, 2H), 7.56 (d, J = 4.0 Hz, 1H), 7.65 (d, J = 7.0 Hz, 2H), 7.98 (d, J = 8.5 Hz, 1H), 8.27 (s, 1H); ^{13}C NMR (125 MHz, $CDCl_3$) δ: 28.2 (3C), 83.6, 107.6, 108.1, 110.0, 110.4, 119.0, 122.1, 123.2, 124.7, 125.4, 125.9, 127.1, 128.5 (2C), 129.2 (2C), 129.9, 133.3, 139.2, 139.5, 140.8, 150.0; HRMS (ESI) calcd for $C_{25}H_{23}N_2O_2$ $[M+H]^+$: 383.1754, found 383.1754.

***tert*-Butyl 4-(*o*-Tolyl)pyrrolo[2,3-*c*]carbazole-3(6*H*)-carboxylate (6bF) and Its [3,2-*c*]-Isomer (7bF) (Table 5)**

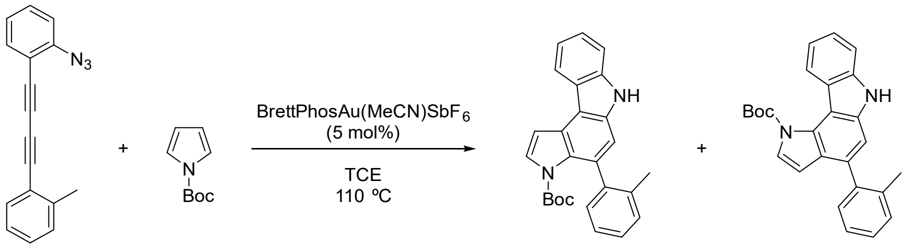

1b **9F** **6bF** **7bF**

To a solution of **1b** (25.7 mg, 0.100 mmol) and **9F** (83.6 mg, 0.500 mmol) in TCE (0.5 mL) was added [BrettPhosAu(MeCN)SbF_6] (5.05 mg, 5.00 μmol) at 110 ºC. The mixture was stirred at 110 ºC in pre-heated bath for 0.5 h and concentrated in vacuo. The residue was purified by column chromatography (hexane/EtOAc = 10/1) to give a mixture of **6bF** and **7bF** (22.8 mg, 58%; **6bF**:**7bF** = 95:5). These isomers were separated by column chromatography on amine silica gel (toluene) to give, in the order of elution, **6bF** and **7bF**.

Compound **6bF**: pale yellow solid; mp 154–156 ºC; IR (neat) 3734, 1730; ^{1}H NMR (500 MHz, $CDCl_3$) δ: 1.32 (s, 9H), 2.17 (s, 3H), 7.20 (d, J = 3.4 Hz, 1H), 7.21 (s, 1H), 7.23-7.26 (m, 3H), 7.30 (dd, J = 7.5, 7.5 Hz, 1H), 7.34-7.37 (m, 1H), 7.42 (dd, J = 7.5, 7.5 Hz, 1H), 7.47 (d, J = 8.0 Hz, 1H), 7.67 (d, J = 3.4 Hz, 1H), 8.13 (br s, 1H), 8.25 (d, J = 7.4 Hz, 1H); ^{13}C NMR (125 MHz, $CDCl_3$) δ: 20.2, 27.6 (3C), 83.1, 104.9, 110.5, 110.7, 113.8, 119.5, 121.2, 123.3, 124.9, 125.2, 125.6, 126.7, 128.0, 128.1, 128.4, 129.0, 129.9, 135.5, 135.9, 139.5, 142.1, 149.1; HRMS (FAB) calcd for $C_{26}H_{25}N_2O_2$ [M+H]$^+$: 397.1911, found 397.1921.

Compound **7bF**: white solid; mp 96–98 ºC; IR (neat) 3707, 1732; ^{1}H NMR (500 MHz, $CDCl_3$) δ: 1.73 (s, 9H), 2.17 (s, 3H), 6.36 (d, J = 4.0 Hz, 1H), 7.23 (s, 1H), 7.28 (m, 2H), 7.32-7.34 (m, 3H), 7.40 (ddd, J = 7.4, 7.4, 1.1 Hz, 1H), 7.44 (d, J = 7.4 Hz, 1H), 7.49 (d, J = 4.0 Hz, 1H), 8.01 (d, J = 8.0 Hz, 1H), 8.26 (s, 1H); ^{13}C NMR (125 MHz, $CDCl_3$) δ: 20.3, 28.2 (3C), 83.5, 108.1, 108.3, 110.0, 110.2, 119.0, 122.1, 124.1, 124.6, 125.1, 125.5, 126.0, 127.5, 129.4, 130.1, 130.3, 133.1, 136.4, 138.9, 139.4, 140.2, 150.1; HRMS (FAB) calcd for $C_{26}H_{25}N_2O_2$ [M+H]$^+$: 397.1911, found 397.1912.

***tert*-Butyl 4-(*m*-Tolyl)pyrrolo[2,3-*c*]carbazole-3(6*H*)-carboxylate (6cF) and Its [3,2-*c*]-Isomer (7cF) (Table 5)**

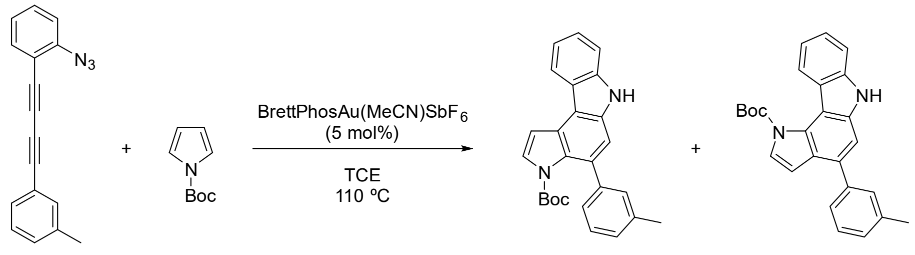

1c 9F 6cF 7cF

To a solution of **1c** (25.7 mg, 0.100 mmol) and **9F** (83.6 mg, 0.500 mmol) in TCE (0.5 mL) was added [BrettPhosAu(MeCN)SbF_6] (5.05 mg, 5.00 μmol) at 110 ºC. The mixture was stirred at 110 ºC in pre-heated bath for 0.5 h and concentrated in vacuo. The residue was purified by column chromatography (hexane/EtOAc = 10/1) to give a mixture of **6cF** and **7cF** (21.7 mg, 55%; **6cF**:**7cF** = 95:5). These isomers were separated by column chromatography on amine silica gel (hexane/toluene = 1/5) to give, in the order of elution, **6cF** and **7cF**.

Compound **6cF**: pale yellow solid; mp 185–186 ºC; IR (neat) 3413, 1732; ^{1}H NMR (500 MHz, $CDCl_3$) δ: 1.30 (s, 9H), 2.37 (s, 3H), 7.13 (d, J = 7.4 Hz, 1H), 7.20 (d, J = 3.4 Hz, 1H), 7.26-7.32 (m, 3H), 7.36-7.42 (m, 4H), 7.73 (d, J = 3.4 Hz, 1H), 8.13 (br s, 1H), 8.23 (d, J = 8.0 Hz, 1H); ^{13}C NMR (125 MHz, $CDCl_3$) δ: 21.5, 27.5 (3C), 83.5, 105.4, 110.1, 110.7, 113.7, 119.5, 121.2, 123.2, 124.5, 124.9, 125.8, 127.4, 127.5, 128.1, 128.6, 128.9, 129.1, 136.4, 137.9, 139.5, 142.6, 149.7; HRMS (FAB) calcd for $C_{26}H_{25}N_2O_2$ [M+H]$^+$: 397.1911, found 397.1920.

Compound **7cF**: pale yellow viscous oil; IR (neat) 3394, 1743; ^{1}H NMR (500 MHz, $CDCl_3$) δ: 1.72 (s, 9H), 2.46 (s, 3H), 6.84 (d, J = 4.0 Hz, 1H), 7.23 (d, J = 7.4 Hz, 1H), 7.25-7.29 (m, 1H), 7.37 (s, 1H), 7.38-7.47 (m, 5H), 7.55 (d, J = 3.4 Hz, 1H), 7.98 (d, J = 8.0 Hz, 1H), 8.25 (br s, 1H); ^{13}C NMR (125 MHz, $CDCl_3$) δ: 21.6, 28.2 (3C), 83.6, 107.5, 108.2, 110.0, 110.3, 119.0, 122.1, 123.2, 124.7, 125.3, 125.9, 126.3, 127.9, 128.4, 129.9, 129.9, 133.4, 138.1, 139.2, 139.5, 140.7, 150.0; HRMS (FAB) calcd for $C_{26}H_{25}N_2O_2$ [M+H]$^+$: 397.1911, found 397.1909.

***tert*-Butyl 4-(*p*-Tolyl)pyrrolo[2,3-*c*]carbazole-3(6*H*)-carboxylate (6dF) and Its [3,2-*c*]-Isomer (7dF) (Table 5)**

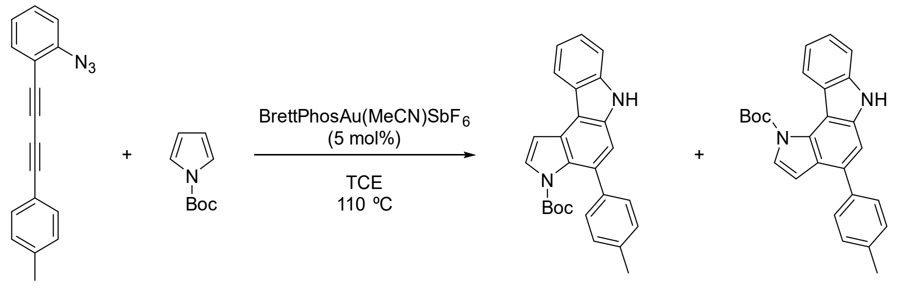

To a solution of **1d** (24.3 mg, 0.100 mmol) and **9F** (83.6 mg, 0.500 mmol) in TCE (0.5 mL) was added [BrettPhosAu(MeCN)SbF_6] (5.05 mg, 5.00 μmol) at 110 ºC. The mixture was stirred at 110 ºC in pre-heated bath for 0.5 h and concentrated in vacuo. The residue was purified by column chromatography (hexane/EtOAc = 10/1) to give a mixture of **6dF** and **7dF** (22.6 mg, 57%; **6dF**:**7dF** = 95:5). These isomers were separated by column chromatography on amine silica gel (toluene) to give, in the order of elution, **6dF** and **7dF**.

Compound **6dF**: pale yellow solid; mp 170–172 ºC; IR (neat) 3402, 1736; ^{1}H NMR (500 MHz, $CDCl_3$) δ: 1.31 (s, 9H), 2.41 (s, 3H), 7.21 (d, *J* = 3.4 Hz, 1H), 7.24 (d, *J* = 8.0 Hz, 2H), 7.29-7.32 (m, 2H), 7.40-7.43 (m, 1H), 7.46-7.49 (m, 3H), 7.72 (d, *J* = 4.0 Hz, 1H), 8.16 (s, 1H), 8.23 (d, *J* = 7.4 Hz, 1H); ^{13}C NMR (125 MHz, $CDCl_3$) δ: 21.2, 27.6 (3C), 83.4, 105.4, 109.9, 110.6, 113.7, 119.5, 121.2, 123.3, 124.8, 125.8, 127.3 (2C), 127.7, 128.8, 129.1, 129.2 (2C), 136.2, 136.5, 139.5, 139.8, 149.6; HRMS (FAB) calcd for $C_{26}H_{25}N_2O_2$ [M+H]$^+$: 397.1911, found 397.1912.

Compound **7dF**: white solid; mp 164–166 ºC; IR (neat) 3415, 1742; ^{1}H NMR (500 MHz, $CDCl_3$) δ: 1.72 (s, 9H), 2.45 (s, 3H), 6.84 (d, *J* = 3.4 Hz, 1H), 7.27 (ddd, *J* = 7.4, 7.4, 1.7 Hz, 1H), 7.32 (d, *J* = 8.0 Hz, 2H), 7.36 (s, 1H), 7.38-7.43 (m, 2H), 7.55 (t, *J* = 3.7 Hz, 3H), 7.98 (d, *J* = 8.0 Hz, 1H), 8.25 (s, 1H); ^{13}C NMR (125 MHz, $CDCl_3$) δ: 21.2, 28.2 (3C), 83.5, 107.4, 108.2, 110.0, 110.2, 119.0, 122.1, 123.2, 124.6, 125.3, 125.9, 129.1 (2C), 129.3 (2C), 129.9, 133.3, 136.9, 137.9, 139.3, 139.5, 150.0; HRMS (FAB) calcd for $C_{26}H_{25}N_2O_2$ [M+H]$^+$: 397.1911, found 397.1923.

***tert*-Butyl 4-(4-Chlorophenyl)pyrrolo[2,3-*c*]carbazole-3(6*H*)-carboxylate (6eF) and Its [3,2-*c*]-Isomer (7eF) (Table 5)**

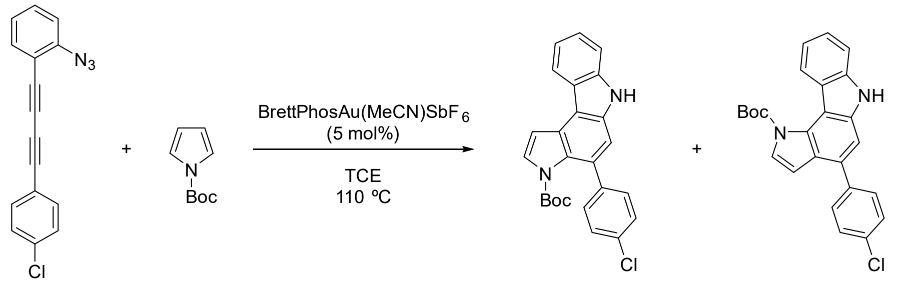

To a solution of **1e** (27.8 mg, 0.100 mmol) and **9F** (83.6 mg, 0.500 mmol) in TCE (0.5 mL) was added [BrettPhosAu(MeCN)SbF_6] (5.05 mg, 5.00 μmol) at 110 ºC. The mixture was stirred at 110 ºC in pre-heated bath for 1.5 h and concentrated in vacuo. The residue was purified by column chromatography (hexane/EtOAc = 10/1) to give a mixture of **6eF** and **7eF** (27.2 mg, 65%; **6eF**:**7eF** = 95:5). These isomers were separated by column chromatography on amine silica gel (toluene) to give, in the order of elution, **6eF** and **7eF**.

Compound **6eF**: pale yellow solid; mp 185–187 ºC; IR (neat) 3395, 1740; ^{1}H NMR (500 MHz, $CDCl_3$) δ: 1.37 (s, 9H), 7.21 (d, *J* = 3.4 Hz, 1H), 7.26 (s, 1H), 7.32 (dd, *J* = 7.3, 7.3, 1.0 Hz, 1H), 7.39 (dd, *J* = 6.3, 1.7 Hz, 2H), 7.43 (ddd, *J* = 6.5, 6.5, 1.0 Hz, 1H), 7.48 (m, 3H), 7.72 (d, *J* = 4.0 Hz, 1H), 8.18 (s, 1H), 8.24 (d, *J* = 7.4 Hz, 1H); ^{13}C NMR (125 MHz, $CDCl_3$) δ: 27.7 (3C), 83.6, 105.6, 110.0, 110.8, 114.1, 119.6, 121.3, 123.1, 125.1, 125.9, 127.4, 128.5 (2C), 128.7 (2C), 129.1, 132.3, 136.4, 139.6, 141.3, 149.3; HRMS (FAB) calcd for $C_{25}H_{22}ClN_2O_2$ $[M+H]^+$: 417.1364, found 417.1373.

Compound **7eF**: white solid; mp 103–105 ºC; IR (neat) 3394, 1747; ^{1}H NMR (500 MHz, $CDCl_3$) δ: 1.72 (s, 9H), 6.77 (d, *J* = 3.4 Hz, 1H), 7.25-7.31 (m, 1H), 7.31 (s, 1H), 7.41 (d, *J* = 3.4 Hz, 2H), 7.45-7.47 (m, 2H), 7.55-7.57 (m, 3H), 7.98 (d, *J* = 8.0 Hz, 1H), 8.25 (s, 1H); ^{13}C NMR (125 MHz, $CDCl_3$) δ: 28.2 (3C), 83.7, 107.4, 107.7, 110.1, 110.6, 119.1, 122.0, 123.0, 124.9, 125.6, 126.0, 128.7 (2C), 123.0, 130.4 (2C), 131.9, 133.1, 139.1, 139.3, 139.5, 150.0; HRMS (FAB) calcd for $C_{25}H_{22}ClN_2O_2$ $[M+H]^+$: 417.1364, found 417.1367.

***tert*-Butyl 4-(4-Nitrophenyl)pyrrolo[2,3-*c*]carbazole-3(6*H*)-carboxylate (6fF) and Its [3,2-*c*]-Isomer (7fF) (Table 5)**

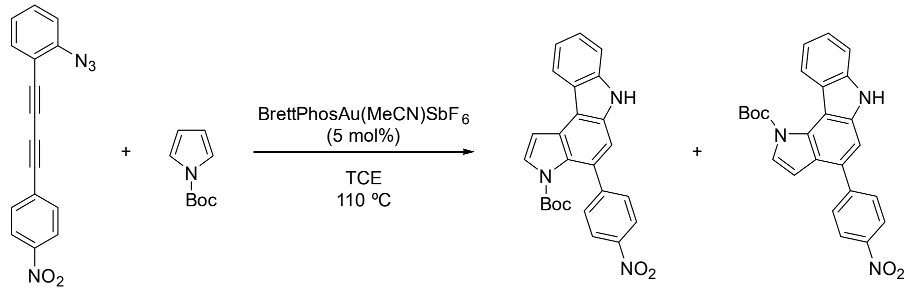

1f **9F** **6fF** **7fF**

To a solution of **1f** (28.8 mg, 0.100 mmol) and **9F** (83.6 mg, 0.500 mmol) in TCE (0.5 mL) was added [BrettPhosAu(MeCN)SbF_6] (5.05 mg, 5.00 μmol) at 110 ºC. The mixture was stirred at 110 ºC in pre-heated bath for 1.5 h and concentrated in vacuo. The residue was purified by column chromatography (hexane/EtOAc = 5/1) to give a mixture of **6fF** and **7fF** (27.4 mg, 64%; **6fF**:**7fF** = 91:9). These isomers were separated by column chromatography on amine silica gel (hexane/toluene = 1/5) to give, in the order of elution, **6fF** and **7fF**.

Compound **6fF**: yellow solid; mp 186–188 ºC; IR (neat) 3396, 1738, 1514; ^{1}H NMR (500 MHz, $CDCl_3$) δ: 1.40 (s, 9H), 7.24 (d, *J* = 3.4 Hz, 1H), 7.31 (s, 1H), 7.34 (dd, *J* = 7.3, 1.5 Hz, 1H), 7.46-7.51 (m, 2H), 7.66-7.68 (m, 2H), 7.73 (d, *J* = 3.4 Hz, 1H), 8.26-8.29 (m, 4H); ^{13}C NMR (125 MHz, $CDCl_3$) δ: 27.8 (3C), 83.8, 105.9, 110.2, 110.9, 115.0, 119.9, 121.5, 122.9, 123.6 (2C), 125.6, 126.1, 126.1, 127.1, 128.1 (2C), 129.0, 136.2, 139.8, 146.2, 149.1, 149.8; HRMS (FAB) calcd for $C_{25}H_{22}N_3O_4$ $[M+H]^+$: 428.1605, found 428.1614.

Compound **7fF**: orange solid; mp 189–191 ºC; IR (neat) 3415, 1744, 1508; ^{1}H NMR (500 MHz, $CDCl_3$) δ: 1.74 (s, 9H), 6.77 (d, *J* = 4.0 Hz, 1H), 7.29-7.32 (m, 1H), 7.40 (s, 1H), 7.44-7.46 (m, 2H), 7.61 (d, *J* = 4.0 Hz, 1H), 7.80 (dd, *J* = 6.6, 2.0 Hz, 2H), 7.99 (d, *J* = 8.0 Hz, 1H), 8.35-8.37 (m, 3H); ^{13}C NMR (125 MHz, $CDCl_3$) δ: 28.1 (3C), 84.0, 107.2, 107.8, 110.2, 111.4, 119.3, 121.8, 122.8, 123.9 (2C), 125.3, 126.1, 126.2, 129.8 (2C), 130.1, 130.4, 139.0, 139.7, 146.8, 147.6, 149.8; HRMS (FAB) calcd for $C_{25}H_{22}N_3O_4$ $[M+H]^+$: 428.1605, found 428.1606.

tert-Butyl 4-(4-Methoxyphenyl)pyrrolo[2,3-_c_]carbazole-3(6_H_)-carboxylate (6gF) and Its [3,2-_c_]-Isomer (7gF) (Table 5)

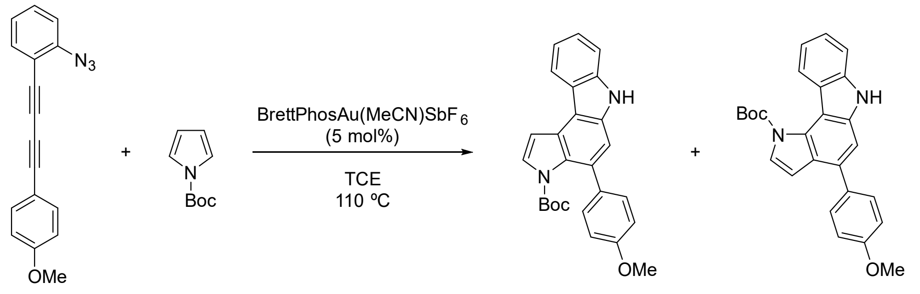

1g **9F** **6gF** **7gF**

To a solution of **1g** (27.3 mg, 0.100 mmol) and **9F** (83.6 mg, 0.500 mmol) in TCE (0.5 mL) was added [BrettPhosAu(MeCN)SbF_6] (5.05 mg, 5.00 μmol) at 110 ºC. The mixture was stirred at 110 ºC in pre-heated bath for 0.5 h and concentrated in vacuo. The residue was purified by column chromatography (hexane/EtOAc = 5/1) to give a mixture of **6gF** and **7gF** (27.8 mg, 67%; **6gF**:**7gF** = 95:5). These isomers were separated by column chromatography on amine silica gel (toluene) to give, in the order of elution, **6gF** and **7gF**.

Compound **6gF**: white solid; mp 118–120 ºC; IR (neat) 3397, 1743; ^{1}H NMR (500 MHz, $CDCl_3$) δ: 1.34 (s, 9H), 3.82 (s, 3H), 6.93 (dd, J = 6.6, 2.0 Hz, 2H), 7.20 (d, J = 4.0 Hz, 1H), 7.24 (s, 1H), 7.30 (ddd, J = 7.3, 7.3, 1.3 Hz, 1H), 7.39-7.44 (m, 2H), 7.47 (dd, J = 6.3, 2.3 Hz, 2H), 7.71 (d, J = 3.4 Hz, 1H), 8.17 (s, 1H), 8.23 (d, J = 7.4 Hz, 1H); ^{13}C NMR (125 MHz, $CDCl_3$) δ: 27.7 (3C), 55.4, 83.4, 105.5, 109.8, 110.7, 113.5, 114.0 (2C), 119.4, 121.1, 123.2, 124.8, 125.9, 127.7, 128.4 (2C), 128.5, 129.1, 135.3, 136.6, 139.5, 149.6, 158.5; HRMS (FAB) calcd for $C_{26}H_{25}N_2O_3$ $[M+H]^+$: 413.1860, found 413.1861.

Compound **7gF**: white solid; mp 102–105 ºC; IR (neat) 3398, 1728; ^{1}H NMR (500 MHz, $CDCl_3$) δ: 1.72 (s, 9H), 3.89 (s, 3H), 6.83 (d, J = 3.4 Hz, 1H), 7.04 (dd, J = 6.3, 2.3 Hz, 2H), 7.26-7.29 (m, 1H), 7.35 (s, 1H), 7.40 (ddd, J = 7.4, 7.4, 1.1 Hz, 1H), 7.44 (d, J = 7.4 Hz, 1H), 7.55 (d, J = 4.0 Hz, 1H), 7.58 (dd, J = 6.6, 2.0 Hz, 2H), 7.97 (d, J = 7.4 Hz, 1H), 8.26 (s, 1H); ^{13}C NMR (125 MHz, $CDCl_3$) δ: 28.2 (3C), 55.4, 83.6, 107.3, 108.1, 110.0, 110.1, 114.0 (2C), 118.9, 122.1, 123.2, 124.6, 125.2, 125.8, 129.9, 130.2 (2C), 133.0, 133.3, 139.3, 139.5, 150.0, 158.9; HRMS (FAB) calcd for $C_{26}H_{25}N_2O_3$ $[M+H]^+$: 413.1860, found 413.1860.

tert-Butyl 9-Cyano-4-phenylpyrrolo[2,3-_c_]carbazole-3(6_H_)-carboxylate (6hF) and Its [3,2-_c_]-Isomer (7hF) (Table 5)

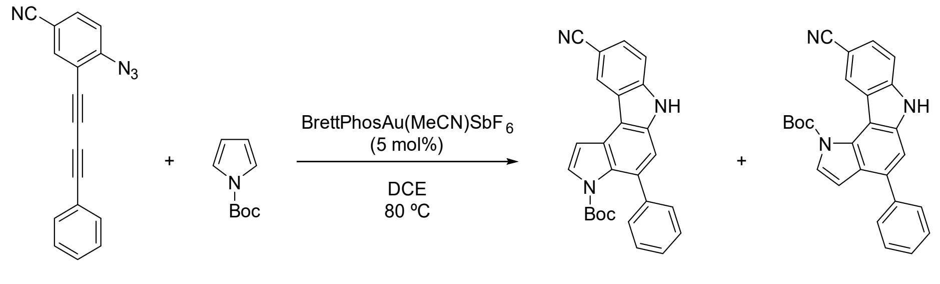

1h **9F** **6hF** **7hF**

To a solution of **1h** (26.8 mg, 0.100 mmol) and **9F** (83.6 mg, 0.500 mmol) in TCE (0.5 mL) was added [BrettPhosAu(MeCN)SbF_6] (5.05 mg, 5.00 μmol) at 110 ºC. The mixture was stirred at 110 ºC in pre-heated bath for 1 h and concentrated in vacuo. The residue was purified by column chromatography (hexane/EtOAc = 3/1) to give an inseparable mixture of **6hF** and **7hF** (24.9 mg, 61%; **6hF**:**7hF** = 81:19): brown solid; IR (neat) 3332, 2218, 1730;

^{1}H NMR (500 MHz, $CDCl_3$; mixture of isomers) δ: 1.33 (s, 9H), 6.84 (d, *J* = 4.0 Hz, 0.2H), 7.15 (d, *J* = 4.0 Hz, 0.8H), 7.27 (s, 0.8H), 7.32-7.36 (m, 1.2H), 7.39-7.53 (m, 4.2H), 7.59-7.62 (m, 1H), 7.65 (dd, *J* = 8.6, 1.7 Hz, 1H), 7.76 (d, *J* = 3.4 Hz, 0.8H), 8.32 (s, 0.2H), 8.51 (d, *J* = 1.1 Hz, 0.8H), 8.57 (s, 0.8H), 8.69 (s, 0.2H); ^{13}C NMR (125 MHz, $CDCl_3$; mixture of isomers) δ: 27.6, 28.4, 84.5, 105.0, 107.7, 108.2, 110.7, 111.3, 120.7, 121.0, 125.8, 126.1, 127.0, 127.4, 127.5, 127.6, 128.7, 129.2, 129.7, 131.5, 135.0, 139.5, 140.2, 141.2, 150.1; HRMS (ESI) calcd for $C_{26}H_{22}N_3O_2$ $[M+H]^+$: 408.1712, found 408.1709.

tert-Butyl 9-Methoxy-4-phenylpyrrolo[2,3-*c*]carbazole-3(6*H*)-carboxylate (6iF) and Its [3,2-*c*]-Isomer (7iF) (Table 5)

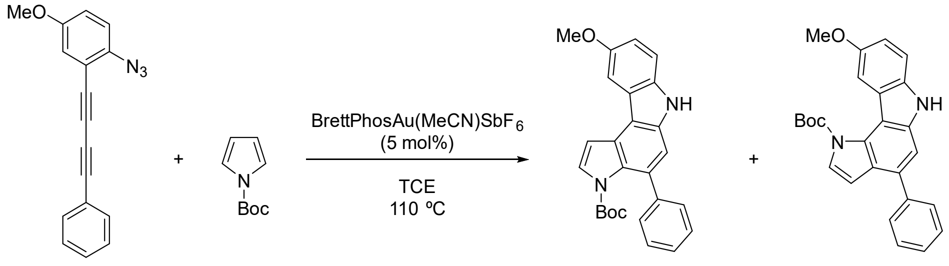

1i **9F** **6iF** **7iF**

To a solution of **1i** (27.3 mg, 0.100 mmol) and **9F** (83.6 mg, 0.500 mmol) in TCE (0.5 mL) was added [BrettPhosAu(MeCN)SbF_6] (5.05 mg, 5.00 μmol) at 110 ºC. The mixture was stirred at 110 ºC in pre-heated bath for 0.5 h and concentrated in vacuo. The residue was purified by column chromatography (hexane/EtOAc = 5/1) to give a mixture of **6iF** and **7iF** (21.5 mg, 52%; **6iF**:**7iF** = 91:9). These isomers were separated by column chromatography on amine silica gel (toluene/Et_2O = 6/1) to give, in the order of elution, **6iF** and **7iF**.

Compound **6iF**: light brown solid; mp 189–190 ºC; IR (neat) 3413, 1747; ^{1}H NMR (500 MHz, $CDCl_3$) δ: 1.30 (s, 9H), 3.98 (s, 3H), 7.07 (dd, *J* = 8.9, 2.6 Hz, 1H), 7.18 (d, *J* = 4.0 Hz, 1H), 7.28 (s, 1H), 7.32 (dd, *J* = 7.4, 7.4 Hz, 1H), 7.36 (d, *J* = 9.2 Hz, 1H), 7.42 (dd, *J* = 7.7, 7.7 Hz, 2H), 7.57 (d, *J* = 7.4 Hz, 2H), 7.70 (d, *J* = 2.3 Hz, 1H), 7.72 (d, *J* = 4.0 Hz, 1H), 8.03 (s, 1H); ^{13}C NMR (125 MHz, $CDCl_3$) δ: 27.6 (3C), 56.2, 83.5, 104.4, 105.3, 110.3, 111.3, 113.8, 113.9, 123.7, 125.8, 126.6, 127.3, 127.4 (2C), 128.5 (2C), 128.8, 129.0, 134.6, 137.3, 142.7, 149.6, 153.9; HRMS (FAB) calcd for $C_{26}H_{25}N_2O_3$ $[M+H]^+$: 413.1860, found 413.1869.

Compound **7iF**: white solid; mp 145–146 ºC; IR (neat) 3589, 1734; ^{1}H NMR (500 MHz, $CDCl_3$) δ: 1.74 (s, 9H), 3.95 (s, 3H), 6.83 (d, *J* = 4.0 Hz, 1H), 7.08 (dd, *J* = 8.6, 2.9 Hz, 1H), 7.36 (d, *J* = 8.6 Hz, 1H), 7.40 (s, 1H), 7.41-7.43 (m, 1H), 7.49-7.52 (m, 2H), 7.55-7.56 (m, 2H), 7.64-7.67 (m, 2H), 8.19 (s, 1H); ^{13}C NMR (125 MHz, $CDCl_3$) δ: 28.2 (3C), 55.9, 83.4, 107.8, 108.3, 108.9, 110.5, 110.6, 114.5, 122.6, 123.0, 125.2, 127.1, 128.5 (2C), 129.2 (2C), 129.9, 133.2, 134.6, 140.1, 140.8, 150.1, 153.3; HRMS (FAB) calcd for $C_{26}H_{25}N_2O_3$ $[M+H]^+$: 413.1860, found 413.1867.

tert-Butyl 6-Phenylindolo[2,3-*c*]carbazole-5(8*H*)-carboxylate (11A) (Table 6)

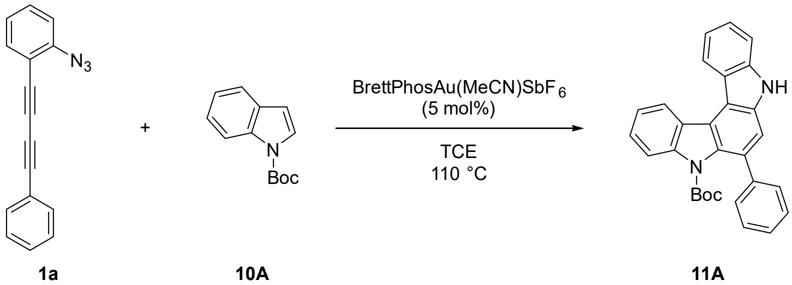

1a **10A** **11A**

To a solution of **1a** (24.3 mg, 0.100 mmol) and **10A** (109 mg, 0.500 mmol) in TCE (0.5 mL) was added [BrettPhosAu(MeCN)SbF_6] (5.05 mg, 5.00 μmol) at 110 ºC. The mixture was stirred at 110 ºC in pre-heated bath for 3 h and concentrated in vacuo. The residue was purified by column chromatography (hexane/toluene = 1/1) to give **11A** (15.1 mg, 35%): white powder; mp 194–195 ºC; IR (neat) 3412, 1750; ^{1}H NMR (500 MHz, $CDCl_3$) δ: 1.23 (s, 9H), 7.33 (dd, J = 7.4, 7.4 Hz, 1H), 7.36-7.39 (m, 1H), 7.44-7.46 (m, 5H), 7.51-7.57 (m, 2H), 7.67-7.68 (m, 2H), 8.25 (dd, J = 7.2, 2.0 Hz, 1H), 8.35 (s, 1H), 8.78 (d, J = 8.0 Hz, 1H), 8.82-8.83 (m, 1H); ^{13}C NMR (125 MHz, $CDCl_3$) δ: 27.5 (3C), 83.6, 110.9, 112.1, 114.6, 115.6, 119.4, 122.4, 122.6, 122.7, 122.9, 123.4, 125.4, 126.2, 126.5, 126.9, 127.2 (2C), 129.1 (2C), 129.4, 131.6, 137.4, 140.0, 140.5, 142.2, 151.1; HRMS (ESI) calcd for $C_{29}H_{25}N_2O_2$ [M+H]$^+$: 433.1916, found 433.1912.

2,2-Dimethyl-1-[6-phenylindolo[2,3-*c*]carbazol-5(8*H*)-yl]propan-1-one (11B) (Table 6)

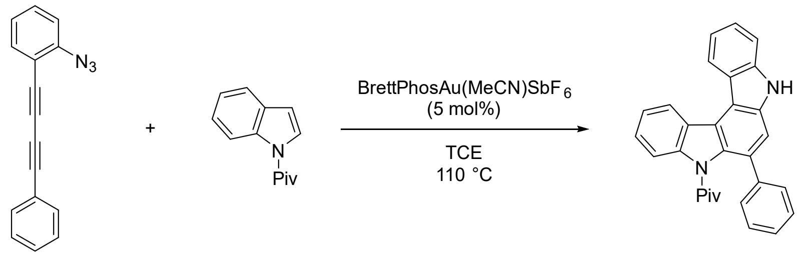

1a **10B** **11B**

To a solution of **1a** (24.3 mg, 0.100 mmol) and **10B** (101 mg, 0.500 mmol) in TCE (0.5 mL) was added [BrettPhosAu(MeCN)SbF_6] (5.05 mg, 5.00 μmol) at 110 ºC. The mixture was stirred at 110 ºC in pre-heated bath for 6.5 h and concentrated in vacuo. The residue was purified by column chromatography (hexane/toluene = 1/1) to give **11B** (4.90 mg, 12%): yellow solid; mp 227–230 ºC; IR (neat) 3412, 1692; ^{1}H NMR (500 MHz, $CDCl_3$) δ: 0.63 (s, 9H), 7.40-7.43 (m, 2H), 7.49-7.51 (m, 6H), 7.58 (dd, J = 8.0, 8.0 Hz, 2H), 7.74-7.75 (m, 2H), 8.38 (s, 1H), 8.86 (d, J = 8.6 Hz, 1H), 8.89-8.90 (m, 1H); ^{13}C NMR (125 MHz, $CDCl_3$) δ: 27.7 (3C), 46.4, 110.9, 111.4, 111.6, 116.1, 119.52, 119.55, 121.1, 122.9, 123.1, 123.6 (2C), 125.5, 126.1, 127.4, 128.0, 129.1 (2C), 130.5 (2C), 132.7, 136.4, 138.8, 139.5, 140.0, 186.7; HRMS (ESI) calcd for $C_{29}H_{25}N_2O$ [M+H]$^+$: 417.1967, found 417.1977

Ethyl 6-Phenylindolo[2,3-*c*]carbazole-5(8*H*)-carboxylate (11C) (Table 6)

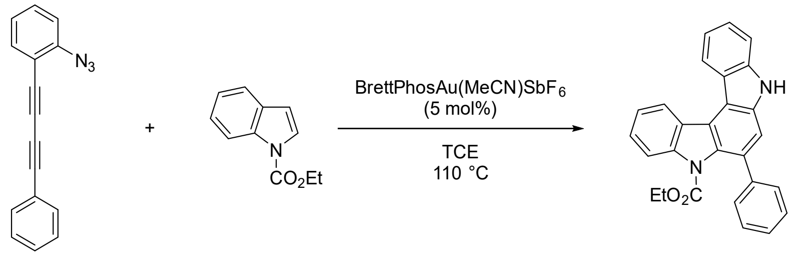

1a **10C** **11C**

To a solution of **1a** (24.3 mg, 0.100 mmol) and **10C** (94.6 mg, 0.500 mmol) in TCE (0.5 mL) was added [BrettPhosAu(MeCN)SbF_6] (5.05 mg, 5.00 μmol) at 110 ºC. The mixture was stirred at 110 ºC in pre-heated bath for 6.5 h and concentrated in vacuo. The residue was purified by column chromatography (hexane/toluene = 1/2) to give **11C** (17.9 mg, 44%): white powder; mp 226–228 ºC; IR (neat) 3410, 1725; ^{1}H NMR (500 MHz, $CDCl_3$) δ: 0.94 (t, J = 7.2 Hz, 3H), 3.59 (q, J = 7.3 Hz, 2H), 7.32-7.35 (m, 1H), 7.36-7.40 (m, 1H), 7.45-7.47 (m, 5H), 7.52-7.59 (m, 2H), 7.63 (dd, J = 8.0, 1.1 Hz, 2H), 8.32-8.34 (m, 2H), 8.78 (d, J = 7.4 Hz, 1H), 8.82-8.83 (m, 1H); ^{13}C NMR (125 MHz,

$CDCl_3$) δ: 13.9, 62.9, 110.9, 112.0, 114.6, 115.7, 119.5, 122.3, 122.6, 122.9 (2C), 123.5, 125.6, 125.9, 126.7, 126.9 (3C), 129.0 (3C), 131.1, 137.4, 140.05, 140.11, 142.3, 152.5; HRMS (ESI) calcd for $C_{27}H_{21}N_2O_2$ $[M+H]^+$: 405.1603, found 405.1594.

Ethyl 2-Bromo-6-phenylindolo[2,3-*c*]carbazole-5(8*H*)-carboxylate (11D) (Table 6)

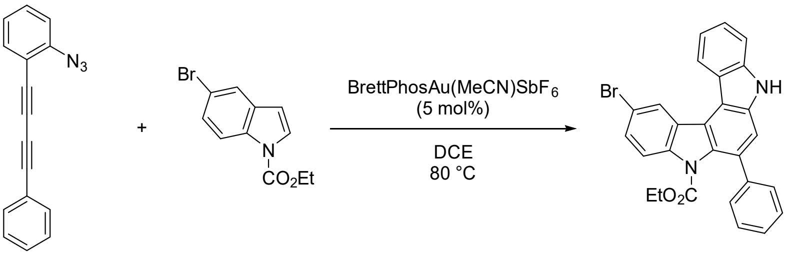

1a **10D** **11D**

To a solution of **1a** (24.3 mg, 0.100 mmol) and **10D** (134 mg, 0.500 mmol) in DCE (0.5 mL) was added [BrettPhosAu(MeCN)SbF_6] (5.05 mg, 5.00 μmol) at 80 ºC. The mixture was stirred at 80 ºC in pre-heated bath for 4.5 h and concentrated in vacuo. The residue was purified by column chromatography (hexane/EtOAc = 5/1) to give **11D** (24.0 mg, 50%): white powder; mp 246–248 ºC; IR (neat) 3420, 1729; ^{1}H NMR (500 MHz, $CDCl_3$) δ: 0.95 (t, *J* = 7.2 Hz, 3H), 3.59 (q, *J* = 7.3 Hz, 2H), 7.37 (dd, *J* = 7.4, 7.4 Hz, 1H), 7.42-7.45 (m, 1H), 7.49-7.54 (m, 4H), 7.55 (s, 1H), 7.64-7.66 (m, 3H), 8.19 (d, *J* = 8.6 Hz, 1H), 8.39 (s, 1H), 8.66 (d, *J* = 8.0 Hz, 1H), 8.90 (d, *J* = 1.7 Hz, 1H); ^{13}C NMR (125 MHz, $CDCl_3$) δ: 13.9, 63.1, 111.0, 112.8, 115.7, 115.8, 116.0, 119.9, 121.2, 122.4, 123.3, 125.4, 125.9, 126.9 (2C), 127.1, 127.7, 129.0, 129.1 (2C), 129.4, 131.6, 137.3, 138.8, 140.0, 142.0, 152.1; HRMS (ESI) calcd for $C_{27}H_{20}BrN_2O_2$ $[M+H]^+$: 483.0708, found 483.0695.

Ethyl 2-Chloro-6-phenylindolo[2,3-*c*]carbazole-5(8*H*)-carboxylate (11E) (Table 6)

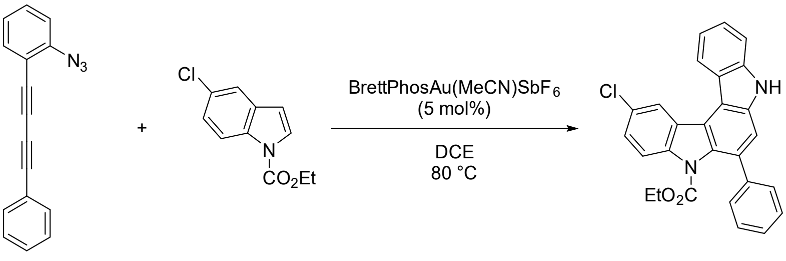

1a **10E** **11E**

To a solution of **1a** (24.3 mg, 0.100 mmol) and **10E** (112 mg, 0.500 mmol) in DCE (0.5 mL) was added [BrettPhosAu(MeCN)SbF_6] (5.05 mg, 5.00 μmol) at 80 ºC. The mixture was stirred at 80 ºC in pre-heated bath for 4.5 h and concentrated in vacuo. The residue was purified by column chromatography (hexane/EtOAc = 5/1) to give **11E** (23.0 mg, 52%): white powder; mp 241–243 ºC; IR (neat) 3420, 1728; ^{1}H NMR (500 MHz, $CDCl_3$) δ: 0.95 (t, *J* = 7.2 Hz, 3H), 3.59 (q, *J* = 7.1 Hz, 2H), 7.36-7.39 (m, 1H), 7.42-7.45 (m, 1H), 7.51-7.53 (m, 5H), 7.56 (s, 1H), 7.65 (dd, *J* = 6.6, 1.4 Hz, 2H), 8.24 (d, *J* = 9.2 Hz, 1H), 8.40 (s, 1H), 8.69 (d, *J* = 8.0 Hz, 1H), 8.76 (d, *J* = 2.3 Hz, 1H); ^{13}C NMR (125 MHz, $CDCl_3$) δ: 13.9, 63.1, 111.0, 112.8, 115.6, 115.7, 119.9, 121.7, 122.4, 122.5, 123.4, 125.9, 126.6, 126.9 (2C), 127.1, 128.3, 129.0, 129.1 (3C), 131.7, 137.3, 138.5, 140.1, 142.1, 152.2; HRMS (ESI) calcd for $C_{27}H_{20}ClN_2O_2$ $[M+H]^+$: 439.1213, found 439.1195.

Diethyl 6-Phenylindolo[2,3-*c*]carbazole-2,5(8*H*)-dicarboxylate (11F) (Table 6)

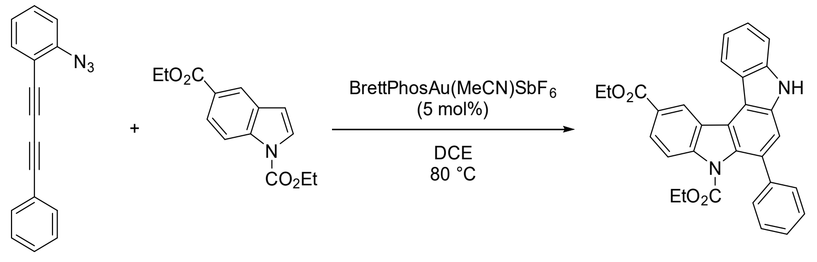

1a **10F** **11F**

To a solution of **1a** (24.3 mg, 0.100 mmol) and **10F** (131 mg, 0.500 mmol) in DCE (0.5 mL) was added [BrettPhosAu(MeCN)SbF_6] (5.05 mg, 5.00 μmol) at 80 ºC. The mixture was stirred at 80 ºC in pre-heated bath for 7.5 h and concentrated in vacuo. The residue was purified by column chromatography (hexane/EtOAc = 5/1) to give **11F** (31.8 mg, <67%): white powder; mp 292–294 ºC; IR (neat) 3342, 1698; ^{1}H NMR (500 MHz, $CDCl_3$) δ: 0.97 (t, *J* = 7.2 Hz, 3H), 1.54 (t, *J* = 7.2 Hz, 3H), 3.60 (q, *J* = 7.3 Hz, 2H), 4.53 (q, *J* = 7.1 Hz, 2H), 7.39 (dd, *J* = 7.4, 7.4 Hz, 1H), 7.46 (dd, *J* = 7.4, 7.4 Hz, 1H), 7.51-7.60 (m, 4H), 7.61 (s, 1H), 7.68 (d, *J* = 7.4 Hz, 2H), 8.27 (dd, *J* = 8.6, 1.1 Hz, 1H), 8.31 (d, *J* = 8.6 Hz, 1H), 8.44 (s, 1H), 8.89 (d, *J* = 8.0 Hz, 1H), 9.61 (s, 1H); ^{13}C NMR (125 MHz, $CDCl_3$) δ: 13.9, 14.5, 61.1, 63.3, 111.0, 112.6, 114.0, 115.9, 120.0, 121.9, 122.5, 123.6, 125.0 (2C), 125.7, 126.0, 127.0 (3C), 127.2, 128.0 ,128.9, 129.1 (2C), 131.6, 137.5, 140.2, 142.0, 152.1, 167.0; HRMS (ESI) calcd for $C_{30}H_{25}N_2O_4$ $[M+H]^+$: 477.1814, found 477.1824

***tert*-Butyl 3-[2-(Phenylethynyl)-1*H*-indol-3-yl]-1*H*-pyrrole-1-carboxylate (4aF) and Its 2-Indolyl Isomer (5aF) (Scheme 5)**

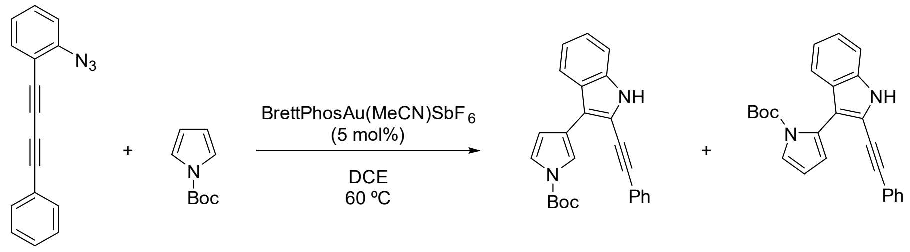

1a **9F** **4aF** **5aF**

To a solution of **1a** (24.3 mg, 0.100 mmol) and **9F** (83.6 mg, 0.500 mmol) in DCE (0.5 mL) was added [BrettPhosAu(MeCN)SbF_6] (5.05 mg, 5.00 μmol) at 60 ºC. The mixture was stirred at 60 ºC in pre-heated bath for 1.5 h and concentrated in vacuo. The residue was purified by column chromatography (hexane/EtOAc = 10/1) to give a mixture of **4aF** and **5aF** (9.40 mg, 25%; **4aF**:**5aF** = 69:31). These isomers were separated by column chromatography on silica gel (hexane/toluene = 1/2) to give, in the order of elution, **4aF** and **5aF**.

Compound **4aF**: brown viscous oil; IR (neat) 3404, 2978, 1738; ^{1}H NMR (500 MHz, $CDCl_3$) δ: 1.63 (s, 9H), 6.96 (dd, *J* = 3.2, 1.4 Hz, 1H), 7.20 (ddd, *J* = 4.0, 4.0, 1.5 Hz, 1H), 7.29 (ddd, *J* = 3.5, 3.5, 1.0 Hz, 1H), 7.34-7.40 (m, 5H), 7.55-7.57 (m, 2H), 7.80 (s, 1H), 7.88 (d, *J* = 8.0 Hz, 1H), 8.21 (s, 1H); ^{13}C NMR (125 MHz, $CDCl_3$) δ: 28.0 (3C), 82.4, 83.6, 95.7, 110.9, 112.1, 115.4, 116.2, 116.9, 120.2, 120.3, 120.4, 120.6, 122.8, 123.9, 125.9, 128.5, 128.5 (2C), 131.3 (2C), 136.2, 148.9; HRMS (FAB) calcd for $C_{25}H_{22}N_2O_2$ $[M^+]$: 382.1681, found 382.1677.

Compound **5aF**: brown viscous oil; IR (neat) 3370, 2979, 1734; ^{1}H NMR (500 MHz, $CDCl_3$) δ: 1.19 (s, 9H), 6.34 (dd, *J* = 3.2, 3.2 Hz, 1H), 6.44 (dd, *J* = 3.4, 1.7 Hz, 1H), 7.13 (dd, *J* = 7.4, 7.4 Hz, 1H), 7.24 (d, *J* = 8.0 Hz, 1H), 7.31-7.34 (m, 4H), 7.44-7.46 (m, 2H), 7.48-7.50 (m, 2H), 8.22 (s, 1H); ^{13}C NMR (125 MHz, $CDCl_3$) δ: 27.3 (3C),

81.7, 83.2, 94.8, 110.7, 110.8, 115.5, 116.3, 117.3, 120.4, 120.5, 122.6, 122.6, 123.7, 126.2, 127.8, 128.3 (2C), 128.4, 131.4 (2C), 135.4, 149.5; HRMS (FAB) calcd for $C_{25}H_{22}N_2O_2$ [M^+]: 382.1681, found 382.1678.

3,6-Dimethyl-4-phenyl-3,6-dihydropyrrolo[2,3-*c*]carbazole (6aF-Me₂) and Its [3,2-*c*]-Isomer (7aF-Me₂)

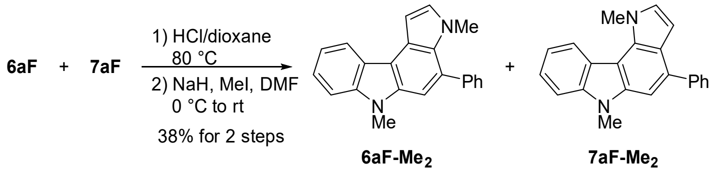

To a solution of **6aF** and **7aF** (50.0 mg, 0.130 mmol; **6aF**:**7aF** = 16:84) in 4 M HCl/dioxane (5.7 mL) was stirred at 80 °C for 16 min. The solvent was removed under vacuum. The crude was then poured into aqueous $NaHCO_3$, extracted with EtOAc, dried over Na_2SO_4, filtered, and concentrated in vacuo. The residue was purified by amine silica gel (hexane/$CHCl_3$ = 2/3) to give an isomeric mixture of **6aA** and **7aA** (22.0 mg, 60%). To a solution of this mixture (obtained by repeated reactions; 75.1 mg, 0.266 mmol) in DMF (0.912 mL) was added NaH (60% dispersion in paraffin liquid; 84.8 mg, 2.12 mmol) at 0 °C under Ar. The reaction mixture was stirred at room temperature for 30 min. MeI (0.132 ml, 2.12 mmol) was added to the reaction mixture. After being stirred for 14 h, the mixture was quenched with H_2O, extracted with $CHCl_3$, washed with brine, dried over Na_2SO_4, filtered, and concentrated in vacuo. The residue was purified by column chromatography (hexane/EtOAc = 30/1) to give **6aF-Me₂** and **7aF-Me₂** (52.8 mg, 64%; **6aF-Me₂**: **7aF-Me₂** = 12:88). These isomers were separated by careful column chromatography on silica gel (hexane/EtOAc = 30/1) to give, in the order of elution, **6aF-Me₂** and **7aF-Me₂.**

Compound **6aF-Me₂**: white powder; mp 140–142 ºC; ^{1}H NMR (500 MHz, $CDCl_3$) δ: 3.39 (s, 3H), 3.91 (s, 3H), 7.09-7.15 (m, 3H), 7.26-7.31 (m, 1H), 7.44-7.46 (m, 5H), 7.56 (d, J = 6.9 Hz, 2H), 8.30 (d, J = 7.4 Hz, 1H); ^{13}C NMR (125 MHz, $CDCl_3$) δ: 29.4, 37.2, 99.1, 106.3, 112.9, 118.4, 121.2, 122.9, 123.0, 123.9, 126.0, 127.2, 127.3, 127.6 (2C), 129.3, 130.3 (2C), 131.1, 135.5, 140.4, 141.2; HRMS (ESI) calcd for $C_{22}H_{19}N_2$ [M+H]$^+$: 311.1548, found 311.1556.

Compound **7aF-Me₂**: white powder; mp 111–113 ºC; ^{1}H NMR (500 MHz, $CDCl_3$) δ: 3.96 (s, 3H), 4.55 (s, 3H), 6.74 (d, J = 2.9 Hz, 1H), 7.03 (d, J = 3.4 Hz, 1H), 7.23 (ddd, J = 7.6, 7.6, 1.3 Hz, 1H), 7.27 (s, 1H), 7.40-7.53 (m, 5H), 7.77-7.78 (m, 2H), 8.56 (d, J = 8.0 Hz, 1H); ^{13}C NMR (125 MHz, $CDCl_3$) δ: 29.5, 39.0, 102.0, 102.7, 106.7, 108.6, 118.6, 120.7, 121.8, 122.3, 123.7, 126.9, 127.0, 127.5, 128.4 (2C), 129.1 (2C), 132.3, 133.8, 139.2, 141.7; HRMS (ESI) calcd for $C_{22}H_{19}N_2$ [M+H]$^+$: 311.1548, found 311.1545.

5,8-Dimethyl-6-phenyl-5,8-dihydroindolo[2,3-*c*]carbazole (11A-Me₂)

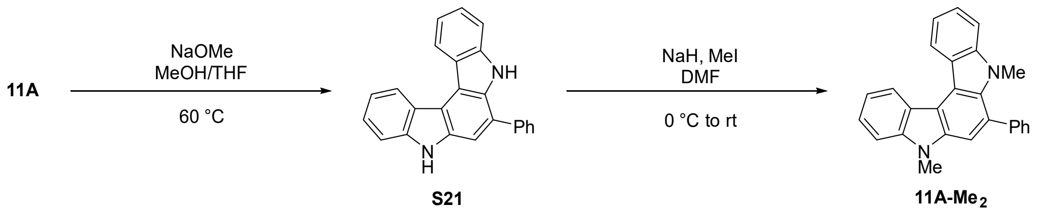

To a solution of **11A** (121 mg, 0.280 mmol) in THF (2.8 mL) was added NaOMe (5 M in MeOH) (1.68 mL, 8.39 mmol) at 60 °C. The mixture was stirred at 60 °C for 60 h. The mixture was quenched with sat. $NaHCO_3$ and the whole was extracted with Et_2O, dried over Na_2SO_4, filtered, and concentrated in vacuo. The residue was purified by column chromatography (hexane/EtOAc = 5/1) to give **S21** (52.2 mg, 56%) as a solid. To a solution of this solid (52.2 mg, 0.157 mmol) in DMF (0.502 mL) was added NaH (60% dispersion in paraffin liquid; 50.2 mg, 1.26 mmol)

at 0 °C under Ar. After being stirred at room temperature for 10 min, MeI (0.0391 ml, 0.628 mmol) was added to the mixture. After being stirred for 40 h, the mixture was quenched with H_2O, extracted with $CHCl_3$, washed with brine, dried over Na_2SO_4, filtered, and concentrated in vacuo. The residue was purified by column chromatography (hexane/EtOAc = 30/1) to give **11A-Me₂** (36.1 mg, 64%): yellow viscous oil; ^{1}H NMR (500 MHz, CD_2Cl_2) δ: 3.46 (s, 3H), 3.97 (s, 3H), 7.40-7.44 (m, 3H), 7.47-7.57 (m, 7H), 7.62 (dd, *J* = 8.3, 1.4 Hz, 2H), 8.93 (d, *J* = 8.0 Hz, 1H), 8.96 (d, *J* = 8.0 Hz, 1H); ^{13}C NMR (125 MHz, CD_2Cl_2) δ: 29.4, 33.7, 108.5, 109.0, 110.1, 115.5, 117.9, 118.4, 118.6, 122.3, 122.5, 123.4, 123.5, 124.8, 125.0, 125.1, 127.3, 127.9 (2C), 130.1 (2C), 134.1, 135.9, 141.1, 141.4, 142.3; HRMS (ESI) calcd for $C_{26}H_{21}N_2$ $[M+H]^+$: 361.1705, found 361.1690.

4. Preparation of the Catalyst

[BrettPhosAu·MeCN]SbF₆ [6]

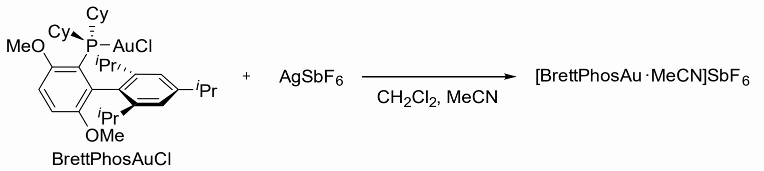

This catalyst was prepared according to the literature procedure for the synthesis of [**L**Au·MeCN]SbF_6 (**L** = {2-[2,4,6-(*i*-Pr)$_3$$C_6H_2$]$C_6H_4$}P(*t*-Bu)$_2$). $AgSbF_6$ (0.6 M solution in CH_2Cl_2; 1.98 mL, 1.19 mmol) was added to a stirred solution of chloro[2-(dicyclohexylphosphino)-3,6-dimethoxy-2',4',6'-triisopropyl-1,1'-biphenyl]gold(I) (Brett-PhosAuCl) (897 mg, 1.17 mmol) in MeCN (7.5 mL) and CH_2Cl_2 (7.5 mL), and the mixture was stirred at room temperature in the dark (using aluminium foil) for 8 h. The mixture was filtered through a pad of Celite and the solvent was removed in vacuo to afford a white powder (1.29 g, quant): ^{1}H NMR (500 MHz, $CDCl_3$) δ: 0.90 (d, *J* = 6.3 Hz, 6H), 1.07-1.09 (m, 2H), 1.17-1.24 (m, 4H), 1.27 (d, *J* = 6.9 Hz, 6H), 1.33 (d, *J* = 6.9 Hz, 6H), 1.37-1.40 (m, 4H), 1.49-1.50 (m, 2H), 1.67-1.98 (m, 8H), 2.25-2.30 (m, 2H), 2.37 (s, 3H), 2.55-2.59 (m, 2H), 2.92-2.98 (m, 1H), 3.56 (s, 3H), 3.94 (s, 3H), 6.95-7.12 (m, 4H); ^{13}C NMR (125 MHz, $CDCl_3$) δ: 2.52, 24.1 (d, *J* = 19.2 Hz, 4C), 24.8 (2C), 25.6 (2C), 26.5 (d, *J* = 16.8 Hz, 2C), 27.0 (d, *J* = 13.2 Hz, 2C), 30.0 (2C), 30.6 (2C), 33.7, 34.6, 34.7, 38.1 (d, *J* = 36.0 Hz, 2C), 54.9, 56.0, 110.8 (d, *J* = 7.2 Hz), 114.2, 114.7, 115.1, 118.9, 121.6 (2C), 131.5 (d, *J* = 8.4 Hz), 136.7 (d, *J* = 13.2 Hz), 147.3, 149.2, 153.1 (d, *J* = 10.8 Hz), 154.8; HRMS (FAB) calcd for $C_{35}H_{53}AuO_2P^+$ [M–MeCN–SbF_6]$^+$: 733.3443, found 733.3444.

5. References

(1) Matsuoka, J.; Matsuda, Y.; Kawada, Y.; Oishi, S.; Ohno, H. *Angew. Chem. Int. Ed.* **2017**, *56*, 7444–7448.
(2) Sakai, N.; Annaka, K.; Konakahara, T. *J. Org. Chem.* **2006**, *71*, 3653–3655.
(3) Nie, X.; Wang, G. *J. Org. Chem.* **2006**, *71*, 4734–4741.
(4) (a) Fiandanese, V.; Bottalico, D.; Marchese G.; Punzi, A, *Tetrahedron* **2008**, *64*, 7301–7306. (b) Padwa, A.; Austin, D. J.; Gareau, Y.; Kassir, J. M.; Xu, S. L. *J. Am. Chem. Soc.* **1993**, *115*, 2637–2647.
(5) Kueh, J. T. B.; Choi, K. W.; Brimble, M. A. *Org. Biomol. Chem.* **2012**, *10*, 5993–6002.
(6) Obradors, C.; Leboeuf, D.; Aydin, J.; Echavarren, A. M. *Org. Lett.* **2013**, *15*, 1576.

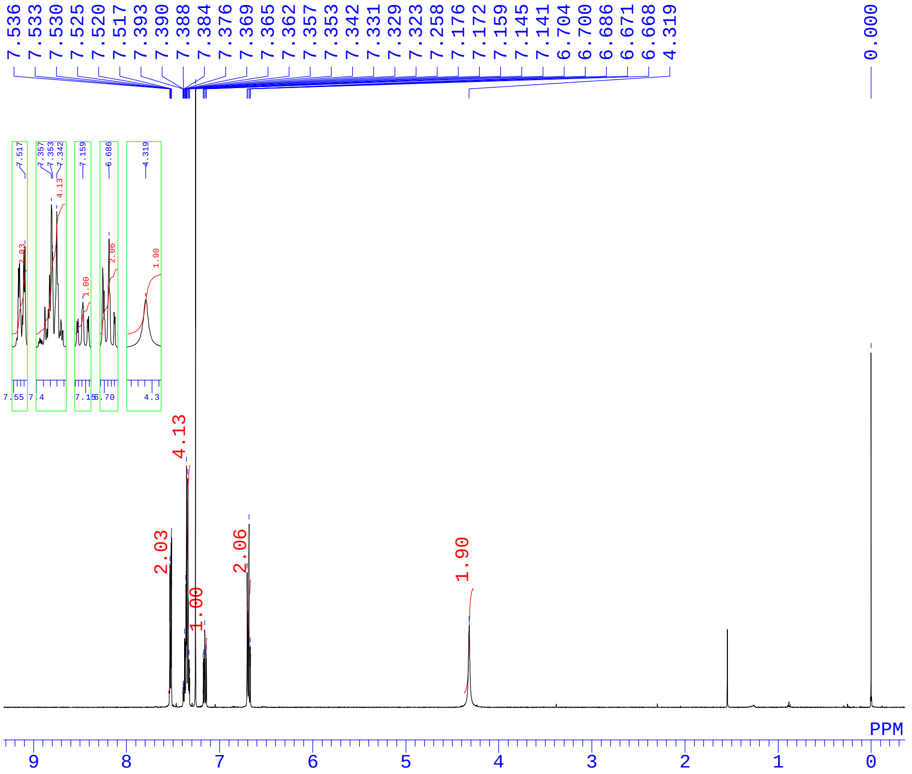

DFILE	aniline H-1.als
COMNT	single_pulse
DATIM	2016-04-13 10:35:
OBNUC	1H
EXMOD	single_pulse.ex2
OBFRQ	500.16 MHz
OBSET	2.41 KHz
OBFIN	6.01 Hz
POINT	13107
FREQU	7507.39 Hz
SCANS	8
ACQTM	1.7459 sec
PD	5.0000 sec
PW1	6.82 usec
IRNUC	1H
CTEMP	21.0 c
SLVNT	CDCL3
EXREF	0.00 ppm
BF	0.12 Hz
RGAIN	50

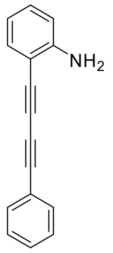

S3

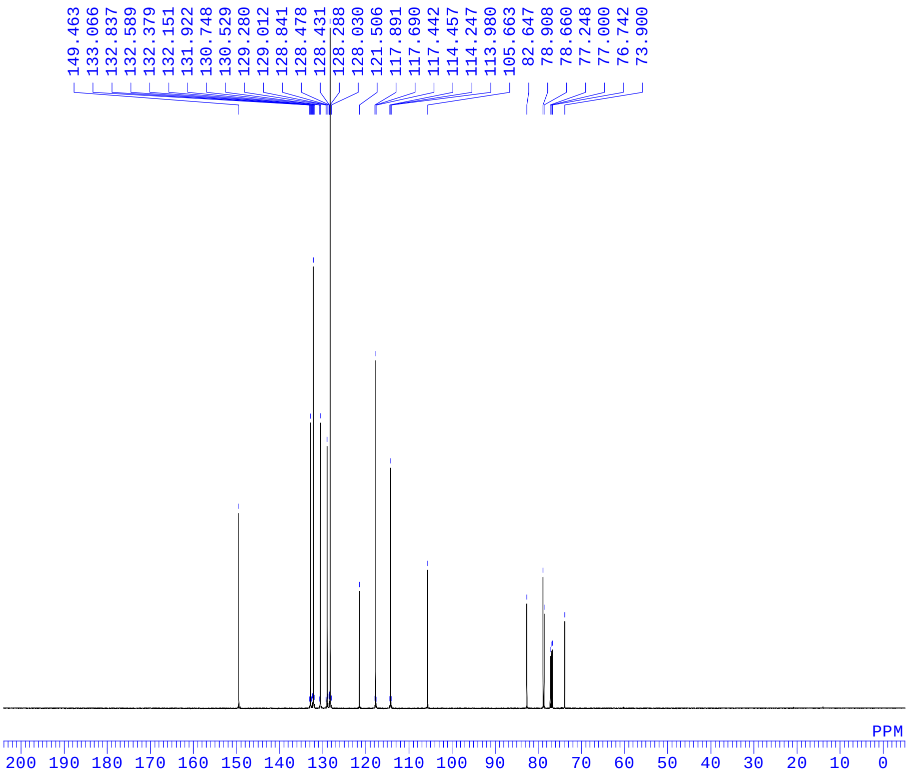

DFILE	aniline C-1.als
COMNT	
DATIM	2017-03-14 19:10:32
OBNUC	13C
EXMOD	single_pulse_dec
OBFRQ	125.77 MHz
OBSET	7.87 KHz
OBFIN	4.21 Hz
POINT	26214
FREQU	31446.06 Hz
SCANS	1024
ACQTM	0.8336 sec
PD	2.0000 sec
PW1	3.67 usec
IRNUC	1H
CTEMP	21.1 c
SLVNT	CDCL3
EXREF	77.00 ppm
BF	1.20 Hz
RGAIN	58

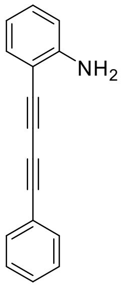

S3

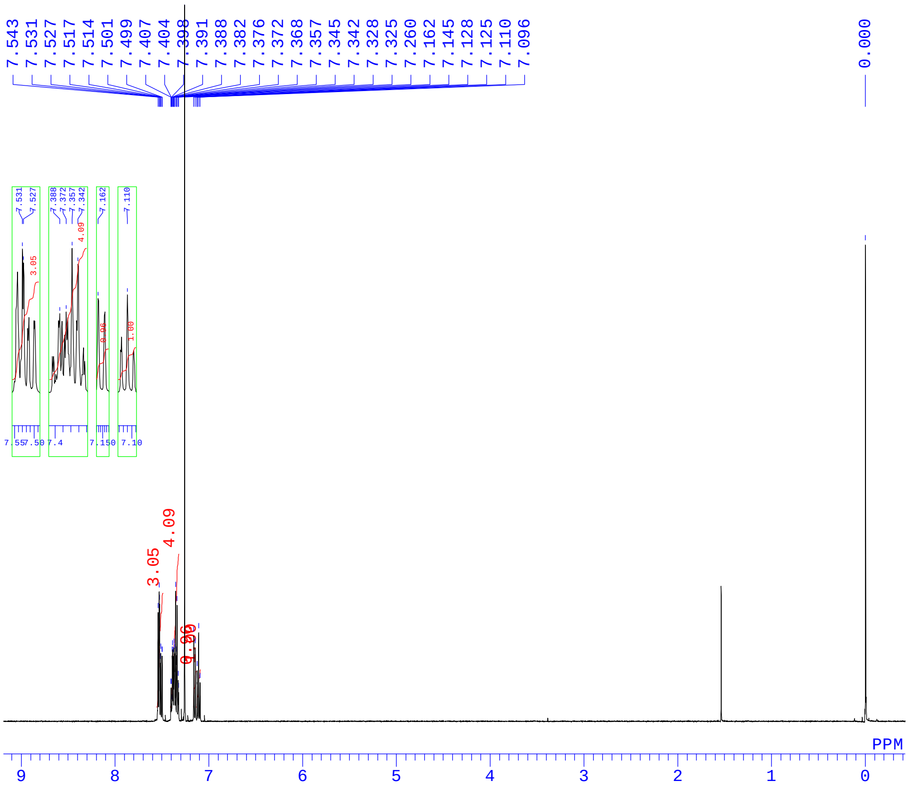

DFILE azide H-1.als
COMNT single_pulse
DATIM 2016-11-04 17:54:18
OBNUC 1H
EXMOD single_pulse.ex2
OBFRQ 500.16 MHz
OBSET 2.41 KHz
OBFIN 6.01 Hz
POINT 13107
FREQU 7507.39 Hz
SCANS 8
ACQTM 1.7459 sec
PD 5.0000 sec
PW1 7.15 usec
IRNUC 1H
CTEMP 21.7 c
SLVNT CDCL3
EXREF 0.00 ppm
BF 0.12 Hz
RGAIN 54

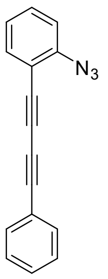

1a

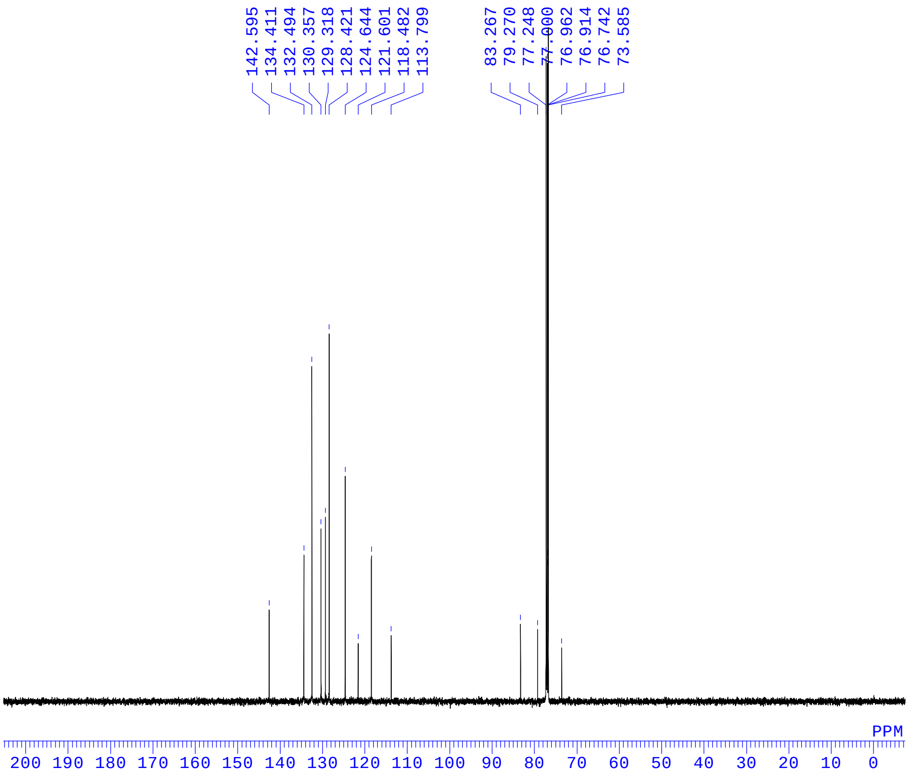

DFILE 10a-C.als
COMNT
DATIM 2017-03-15 12:29:01
OBNUC 13C
EXMOD single_pulse_dec
OBFRQ 125.77 MHz
OBSET 7.87 KHz
OBFIN 4.21 Hz
POINT 26214
FREQU 31446.06 Hz
SCANS 1058
ACQTM 0.8336 sec
PD 2.0000 sec
PW1 3.67 usec
IRNUC 1H
CTEMP 21.2 c
SLVNT CDCL3
EXREF 77.00 ppm
BF 1.20 Hz
RGAIN 58

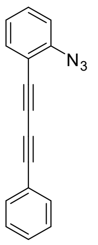

1a

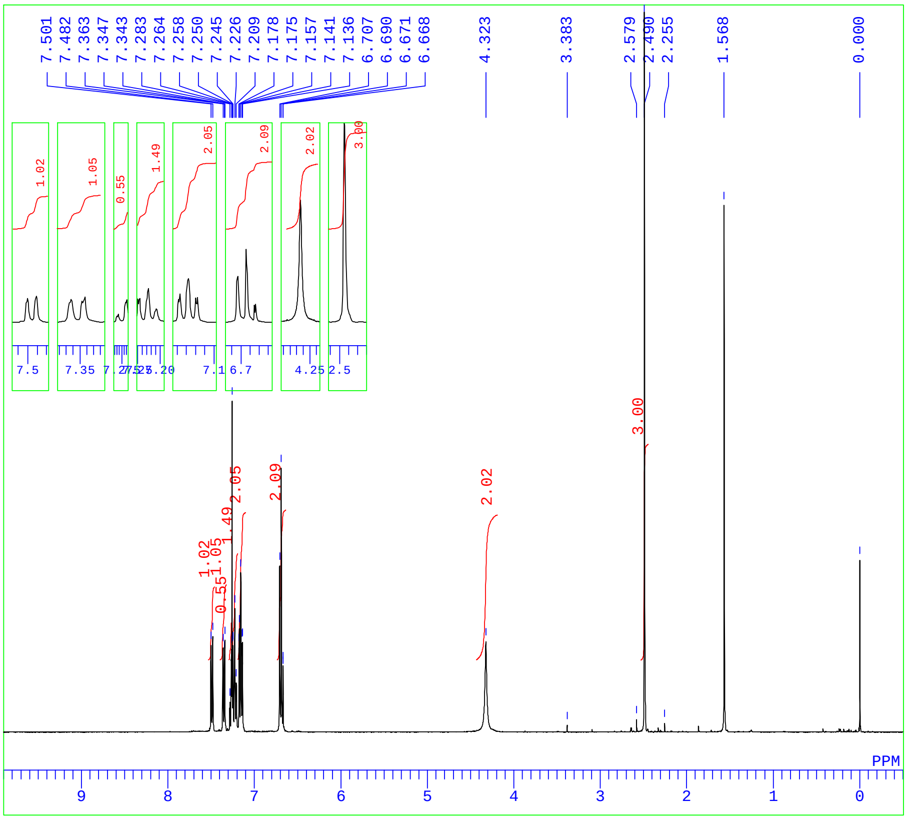

DFILE A118.als
COMNT
DATIM 2018-04-25 12:09:37
OBNUC 1H
EXMOD proton.jxp
OBFRQ 399.78 MHz
OBSET 4.19 KHz
OBFIN 7.29 Hz
POINT 13107
FREQU 7621.95 Hz
SCANS 8
ACQTM 1.7197 sec
PD 5.0000 sec
PW1 3.15 usec
IRNUC 1H
CTEMP 20.2 c
SLVNT CDCL3
EXREF 0.00 ppm
BF 0.12 Hz
RGAIN 40

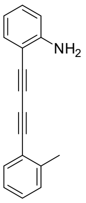

S5

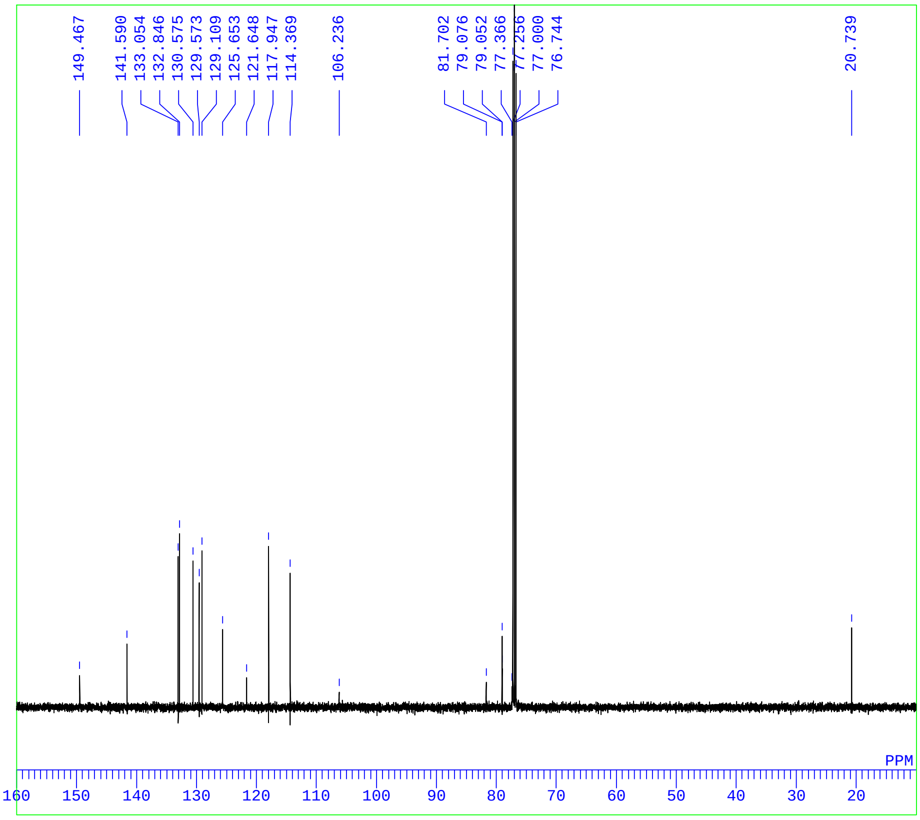

DFILE A118 C.als
COMNT
DATIM 2018-04-27 10:31:21
OBNUC 13C
EXMOD single_pulse_dec
OBFRQ 125.77 MHz
OBSET 7.87 KHz
OBFIN 4.21 Hz
POINT 16384
FREQU 25163.56 Hz
SCANS 2049
ACQTM 0.6511 sec
PD 1.0000 sec
PW1 3.67 usec
IRNUC 1H
CTEMP 21.7 c
SLVNT CDCL3
EXREF 77.00 ppm
BF 0.12 Hz
RGAIN 72

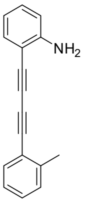

S5

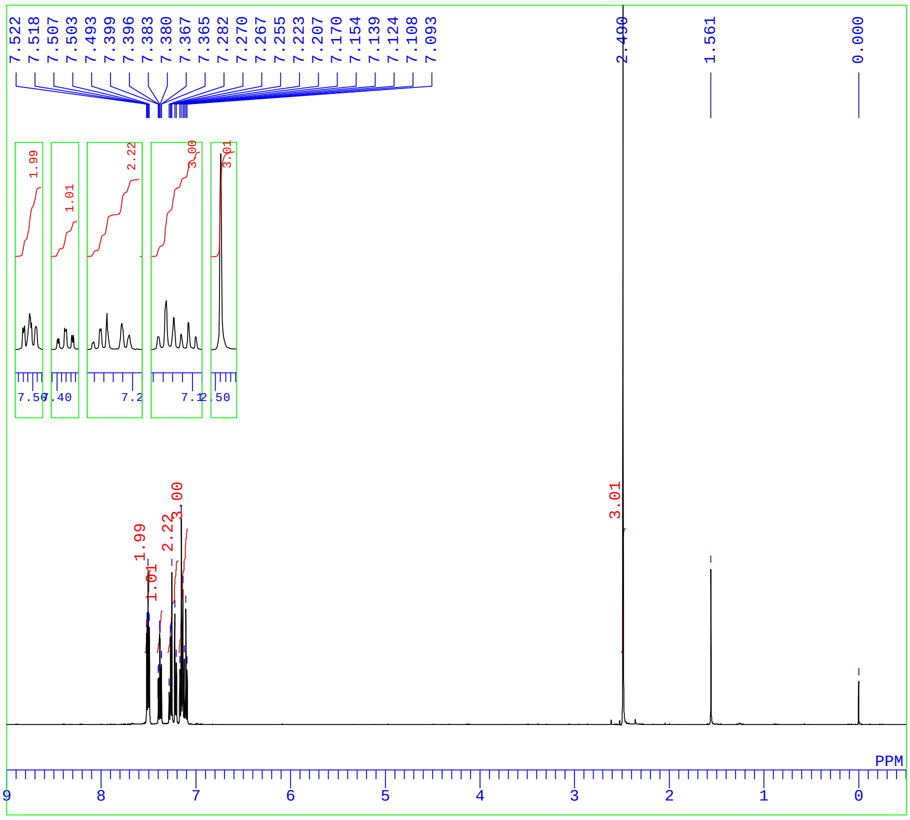

DFILE A140.als
COMNT single_pulse1
DATIM 2018-04-25 20:14:19
OBNUC 1H
EXMOD single_pulse.ex2
OBFRQ 500.16 MHz
OBSET 2.41 KHz
OBFIN 6.01 Hz
POINT 13107
FREQU 7507.39 Hz
SCANS 8
ACQTM 1.7459 sec
PD 5.0000 sec
PW1 7.15 usec
IRNUC 1H
CTEMP 21.1 c
SLVNT CDCL3
EXREF 0.00 ppm
BF 0.12 Hz
RGAIN 50

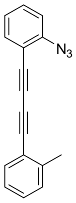

1b

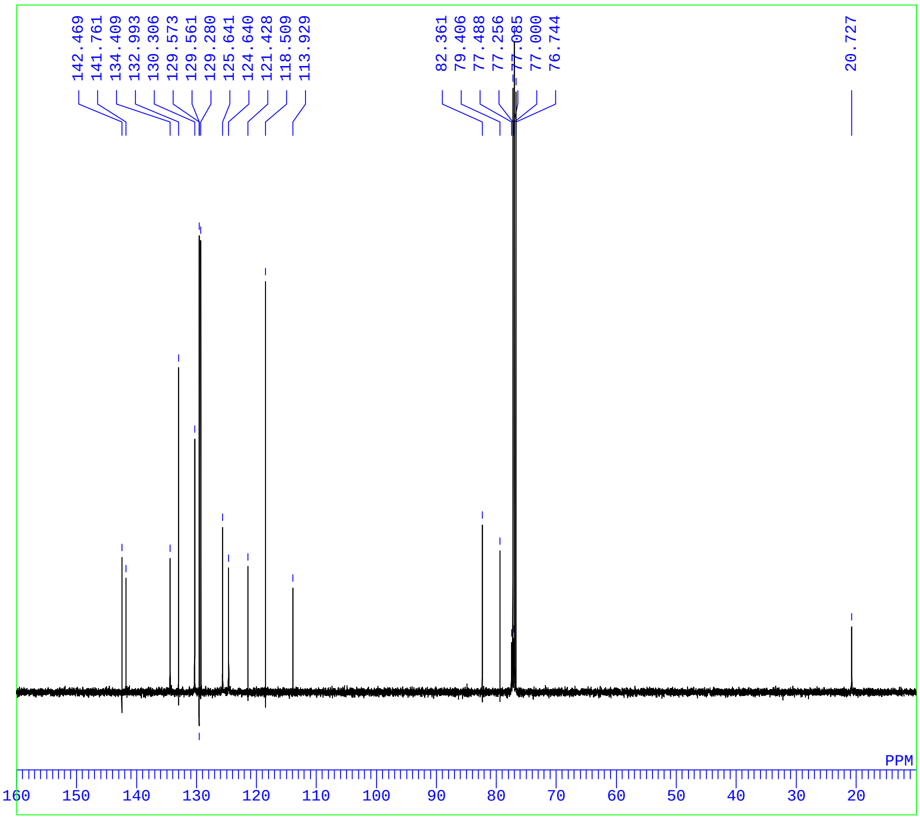

DFILE A140 C.als
COMNT
DATIM 2018-04-26 01:04:54
OBNUC 13C
EXMOD single_pulse_dec
OBFRQ 125.77 MHz
OBSET 7.87 KHz
OBFIN 4.21 Hz
POINT 16384
FREQU 25163.56 Hz
SCANS 2200
ACQTM 0.6511 sec
PD 1.0000 sec
PW1 3.67 usec
IRNUC 1H
CTEMP 22.2 c
SLVNT CDCL3
EXREF 77.00 ppm
BF 0.12 Hz
RGAIN 70

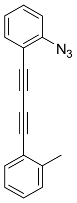

1b

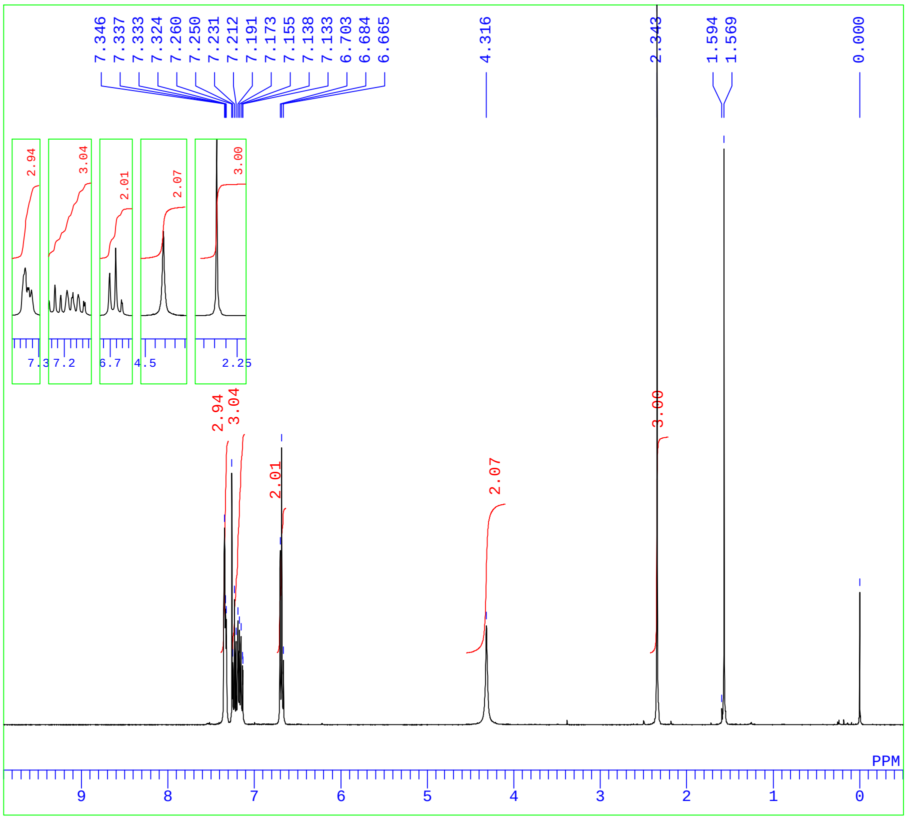

DFILE A124.als
COMNT single_pulse
DATIM 2018-04-25 12:28:09
OBNUC 1H
EXMOD proton.jxp
OBFRQ 399.78 MHz
OBSET 4.19 KHz
OBFIN 7.29 Hz
POINT 13107
FREQU 7621.95 Hz
SCANS 8
ACQTM 1.7197 sec
PD 5.0000 sec
PW1 3.15 usec
IRNUC 1H
CTEMP 19.8 c
SLVNT CDCL3
EXREF 0.00 ppm
BF 0.12 Hz
RGAIN 38

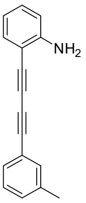

S7

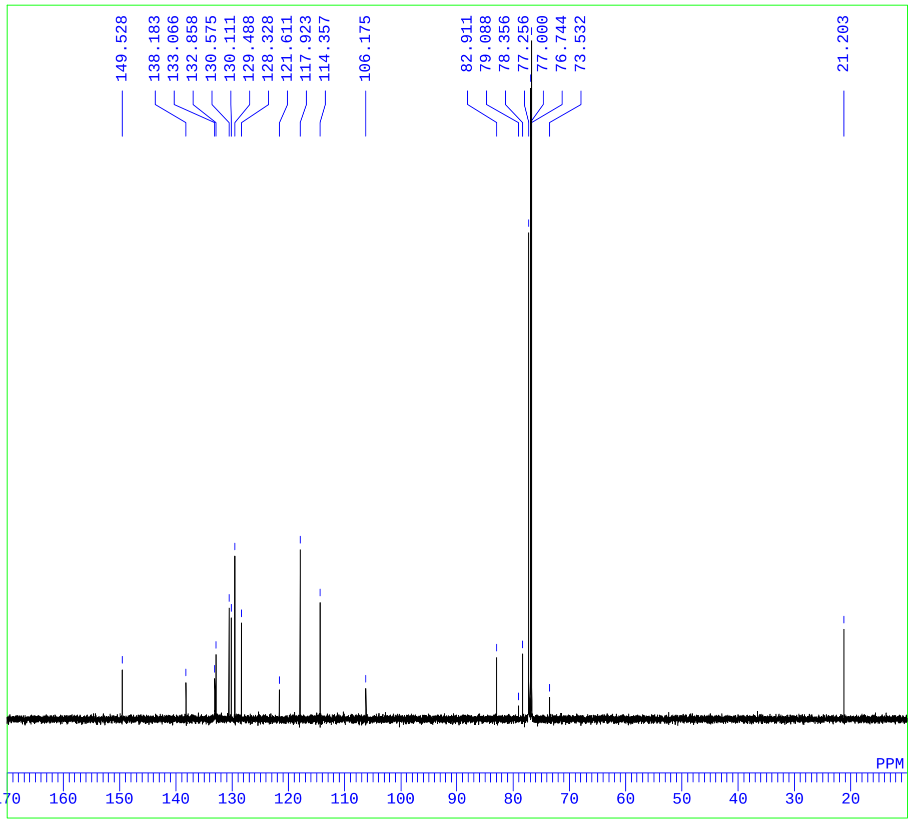

DFILE A124 C.als
COMNT
DATIM 2018-04-26 02:13:05
OBNUC 13C
EXMOD single_pulse_dec
OBFRQ 125.77 MHz
OBSET 7.87 KHz
OBFIN 4.21 Hz
POINT 16384
FREQU 25163.56 Hz
SCANS 2200
ACQTM 0.6511 sec
PD 1.0000 sec
PW1 3.67 usec
IRNUC 1H
CTEMP 22.2 c
SLVNT CDCL3
EXREF 77.00 ppm
BF 0.12 Hz
RGAIN 70

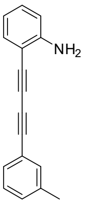

S7

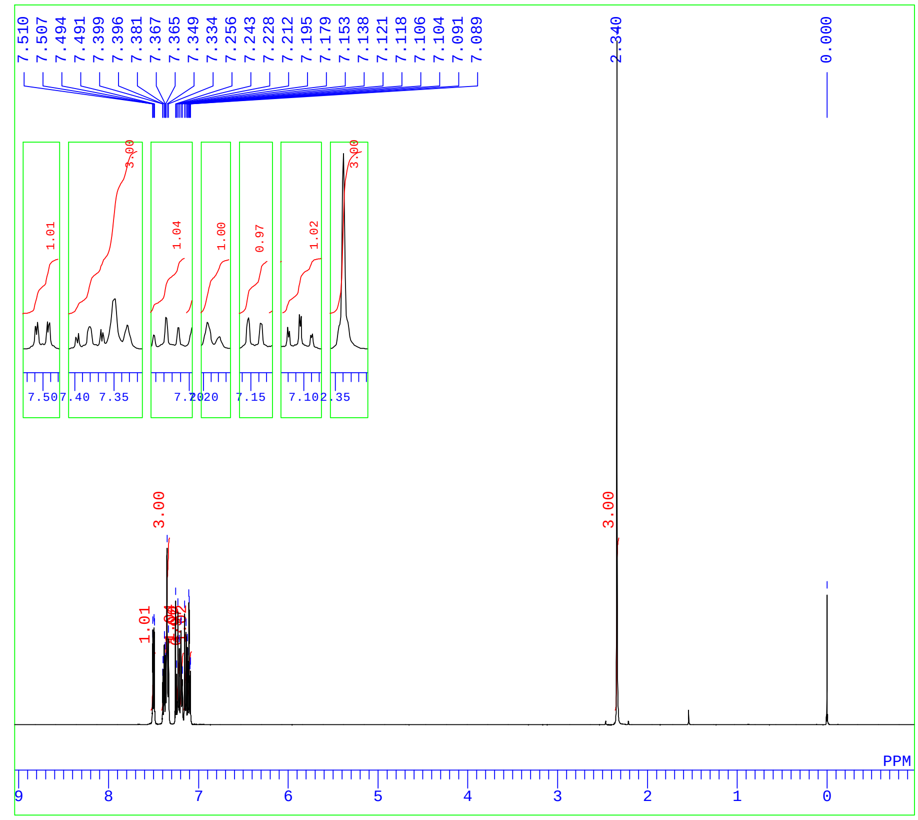

DFILE A-142 H-1.als
COMNT single_pulse
DATIM 2016-02-13 10:04:19
OBNUC 1H
EXMOD single_pulse.ex2
OBFRQ 500.16 MHz
OBSET 2.41 KHz
OBFIN 6.01 Hz
POINT 13107
FREQU 7507.39 Hz
SCANS 8
ACQTM 1.7459 sec
PD 5.0000 sec
PW1 6.82 usec
IRNUC 1H
CTEMP 20.3 c
SLVNT CDCL3
EXREF 0.00 ppm
BF 0.12 Hz
RGAIN 46

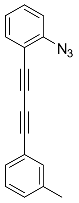

1c

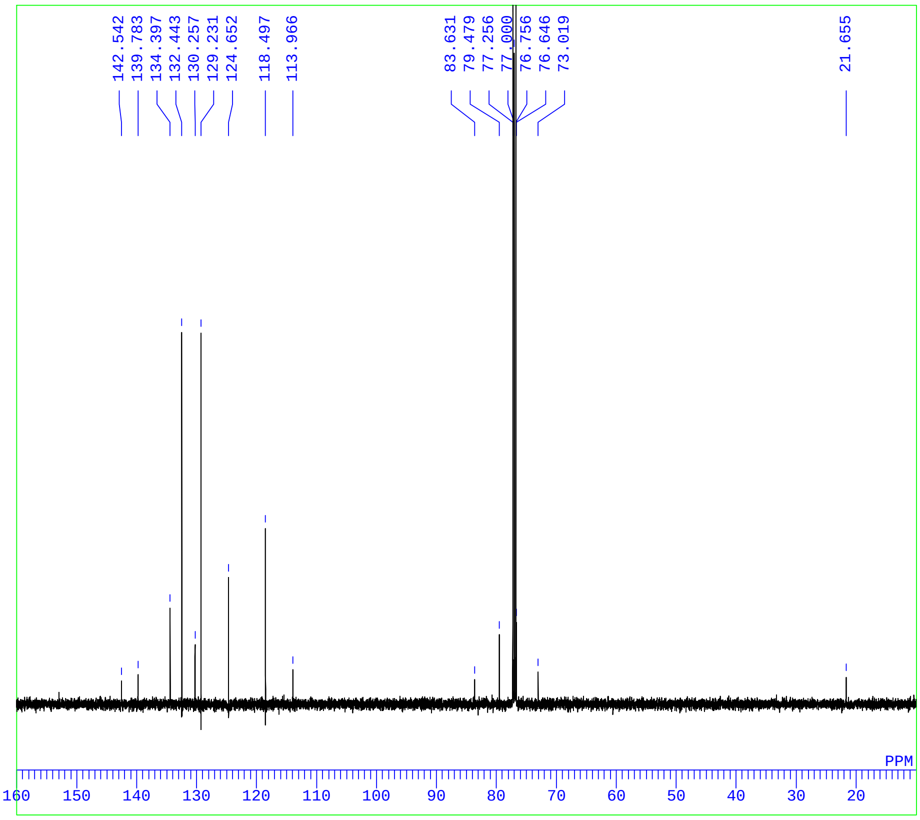

DFILE A142 C.als
COMNT
DATIM 2018-04-26 04:30:23
OBNUC 13C
EXMOD single_pulse_dec
OBFRQ 125.77 MHz
OBSET 7.87 KHz
OBFIN 4.21 Hz
POINT 16384
FREQU 25163.56 Hz
SCANS 2200
ACQTM 0.6511 sec
PD 1.0000 sec
PW1 3.67 usec
IRNUC 1H
CTEMP 22.2 c
SLVNT CDCL3
EXREF 77.00 ppm
BF 0.12 Hz
RGAIN 70

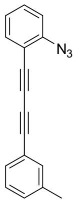

1c

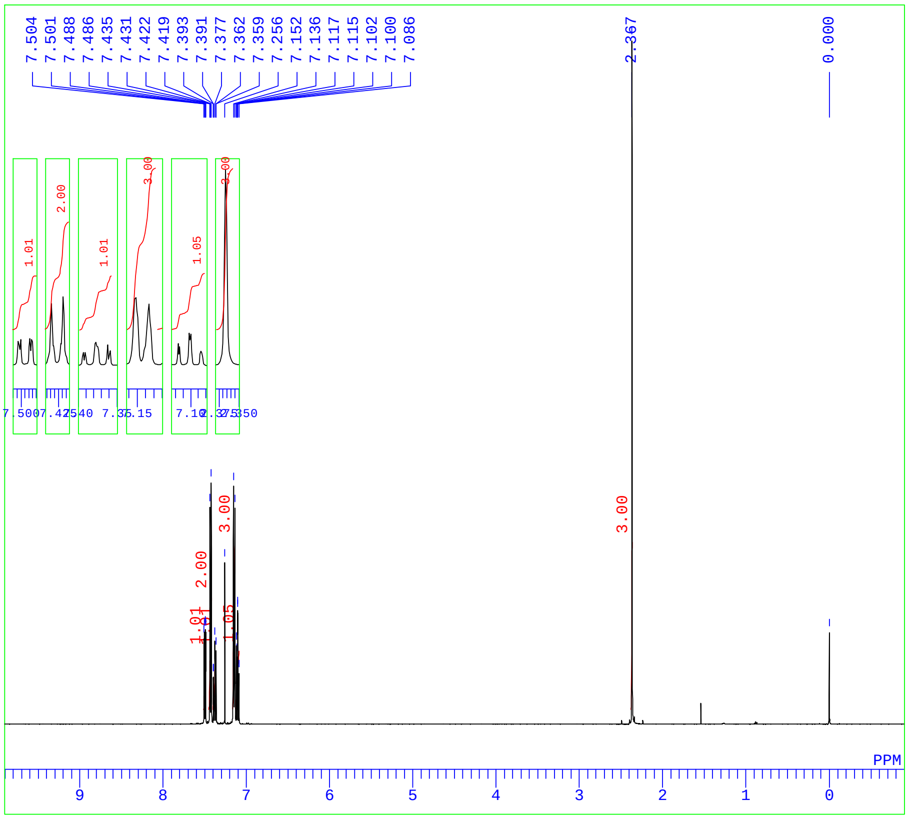

DFILE A-138 H-2.als
COMNT single_pulse
DATIM 2016-02-12 16:31:11
OBNUC 1H
EXMOD single_pulse.ex2
OBFRQ 500.16 MHz
OBSET 2.41 KHz
OBFIN 6.01 Hz
POINT 13107
FREQU 7507.39 Hz
SCANS 8
ACQTM 1.7459 sec
PD 5.0000 sec
PW1 6.82 usec
IRNUC 1H
CTEMP 20.3 c
SLVNT CDCL3
EXREF 0.00 ppm
BF 0.12 Hz
RGAIN 46

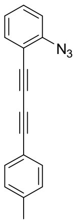

1d

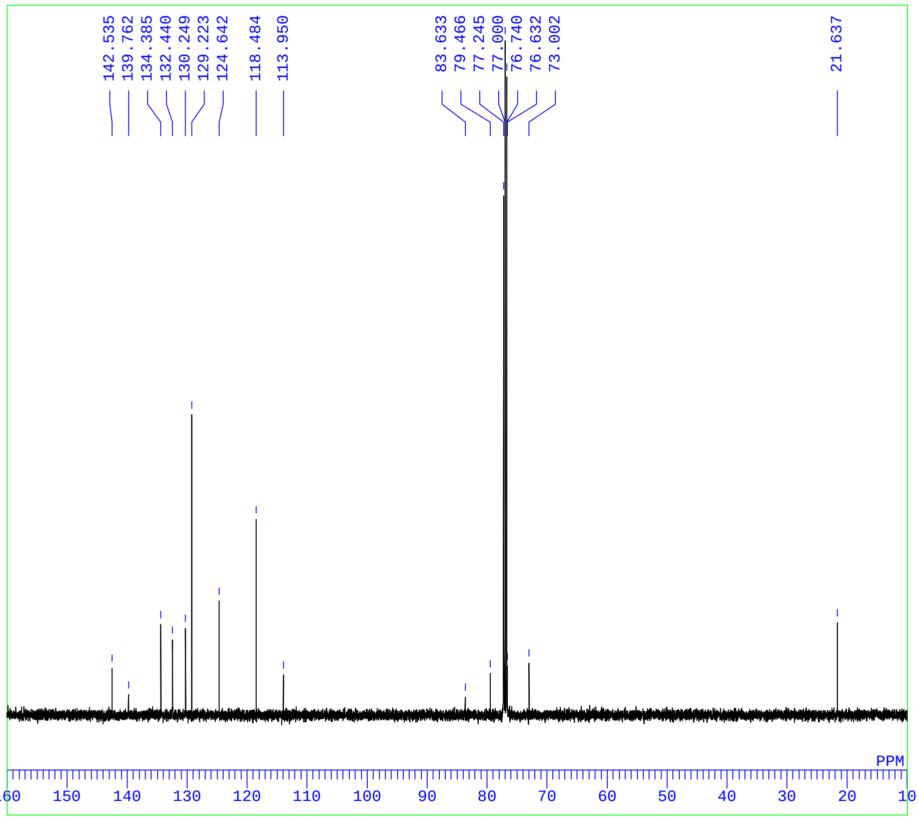

DFILE A138 C.als
COMNT
DATIM 2018-04-28 11:02:20
OBNUC 13C
EXMOD single_pulse_dec
OBFRQ 125.77 MHz
OBSET 7.87 KHz
OBFIN 4.21 Hz
POINT 13107
FREQU 25252.14 Hz
SCANS 2136
ACQTM 0.5190 sec
PD 1.0000 sec
PW1 3.67 usec
IRNUC 1H
CTEMP 21.4 c
SLVNT CDCL3
EXREF 77.00 ppm
BF 0.12 Hz
RGAIN 58

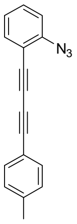

1d

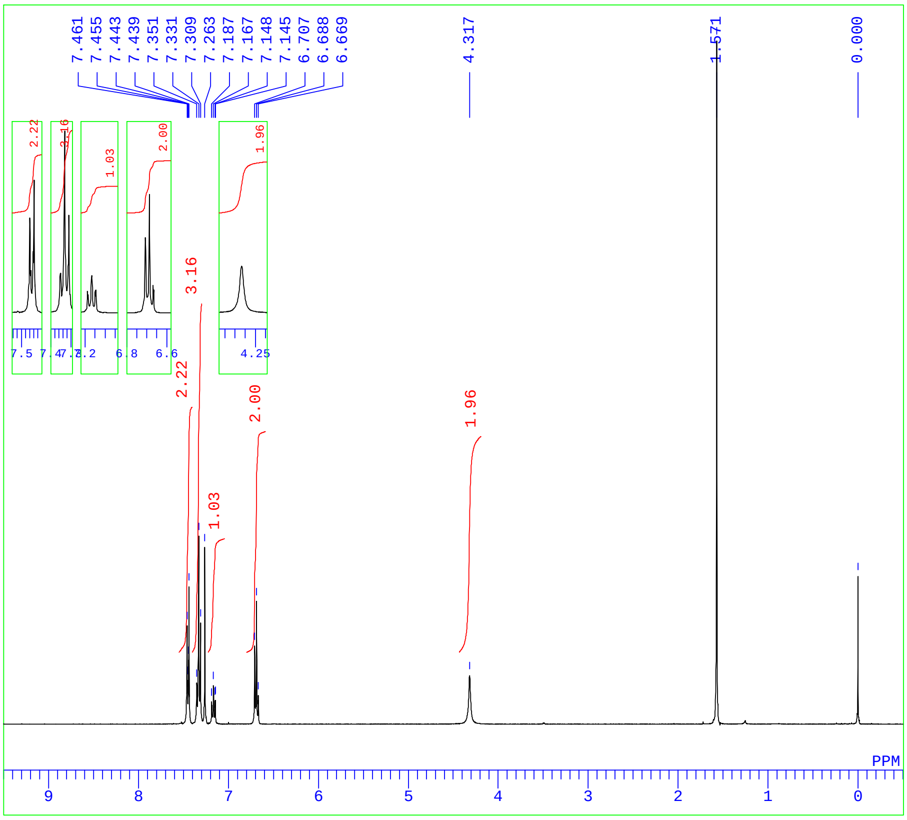


```
DFILE  A-123.als
COMNT  single_pulse
DATIM  2018-04-25 12:40:33
OBNUC  1H
EXMOD  proton.jxp
OBFRQ        399.78 MHz
OBSET          4.19 KHz
OBFIN          7.29 Hz
POINT         13107
FREQU       7621.95 Hz
SCANS             8
ACQTM        1.7197 sec
PD           5.0000 sec
PW1            3.15 usec
IRNUC  1H
CTEMP          19.8 c
SLVNT  CDCL3
EXREF          0.00 ppm
BF             0.12 Hz
RGAIN            44
```

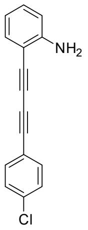

S11

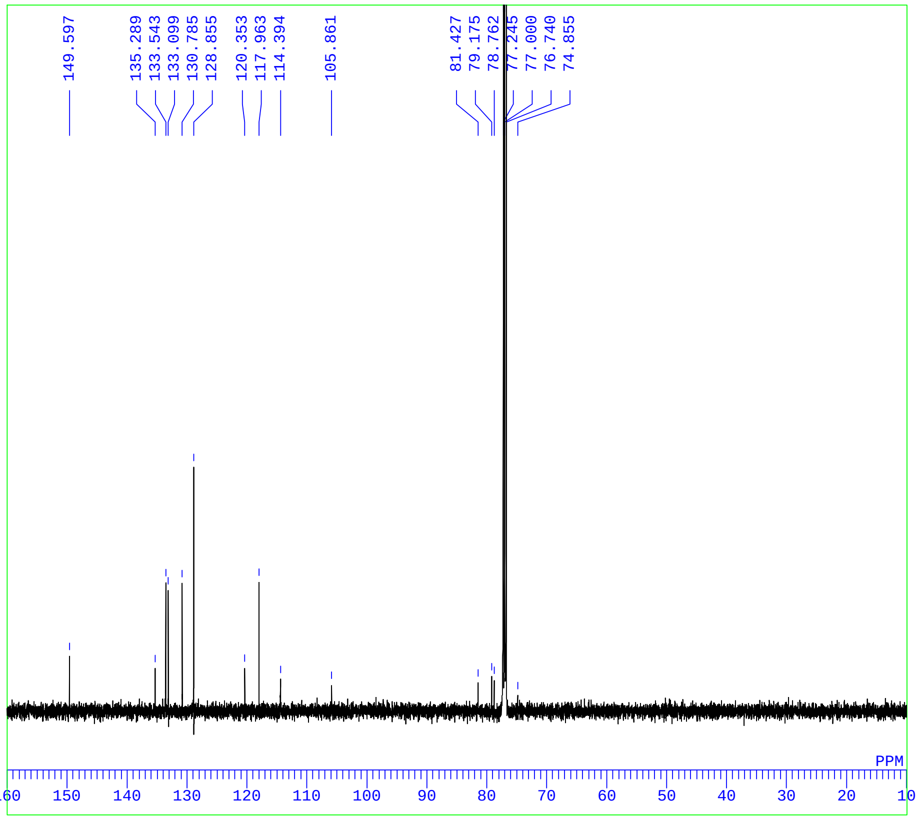


```
DFILE  A123 C.als
COMNT
DATIM  2018-04-28 19:07:07
OBNUC  13C
EXMOD  single_pulse_dec
OBFRQ        125.77 MHz
OBSET          7.87 KHz
OBFIN          4.21 Hz
POINT         16384
FREQU      31565.66 Hz
SCANS          3555
ACQTM        0.5190 sec
PD           1.0000 sec
PW1            3.67 usec
IRNUC  1H
CTEMP          21.6 c
SLVNT  CDCL3
EXREF         77.00 ppm
BF             0.12 Hz
RGAIN            58
```

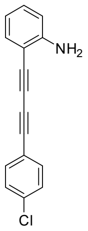

S11

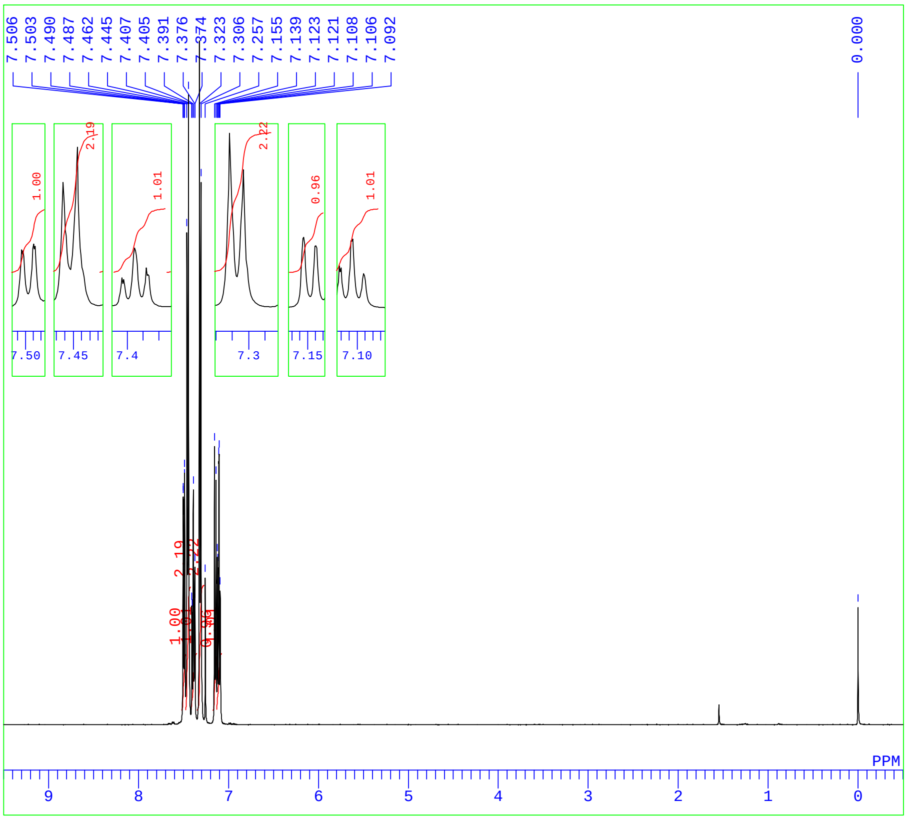

DFILE A-141 H-1.als
COMNT single_pulse
DATIM 2016-02-13 09:33:22
OBNUC 1H
EXMOD single_pulse.ex2
OBFRQ 500.16 MHz
OBSET 2.41 KHz
OBFIN 6.01 Hz
POINT 13107
FREQU 7507.39 Hz
SCANS 8
ACQTM 1.7459 sec
PD 5.0000 sec
PW1 6.82 usec
IRNUC 1H
CTEMP 20.0 c
SLVNT CDCL3
EXREF 0.00 ppm
BF 0.12 Hz
RGAIN 44

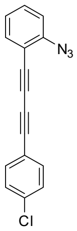

1e

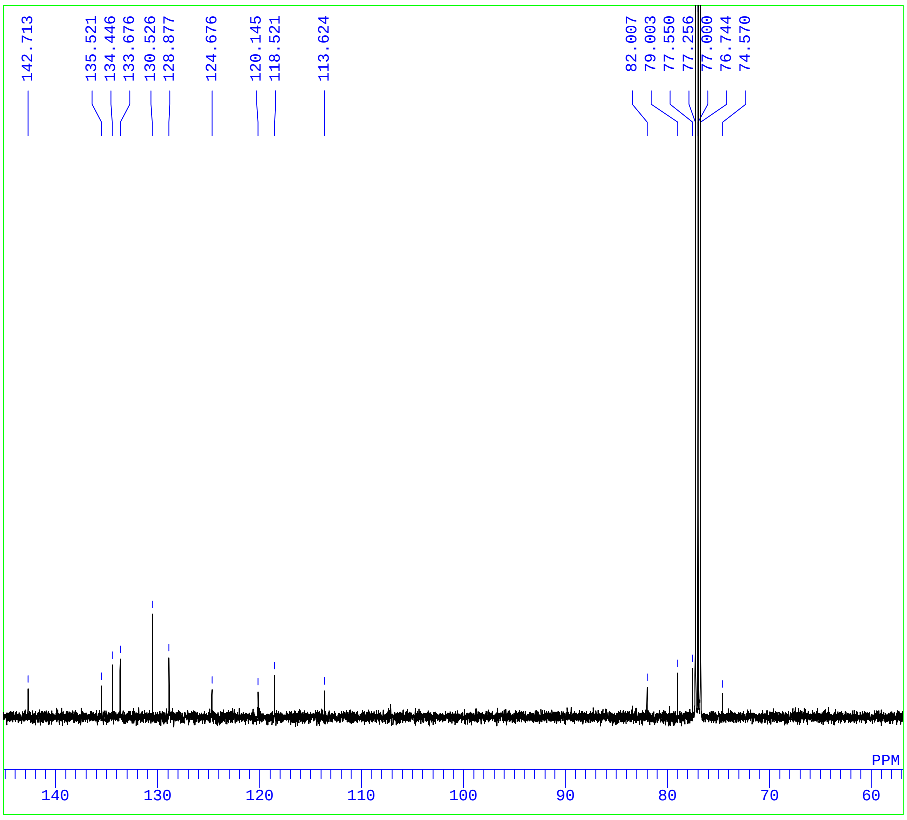

DFILE A141 C.als
COMNT
DATIM 2018-04-26 06:50:42
OBNUC 13C
EXMOD single_pulse_dec
OBFRQ 125.77 MHz
OBSET 7.87 KHz
OBFIN 4.21 Hz
POINT 16384
FREQU 25163.56 Hz
SCANS 2200
ACQTM 0.6511 sec
PD 1.0000 sec
PW1 3.67 usec
IRNUC 1H
CTEMP 22.2 c
SLVNT CDCL3
EXREF 77.00 ppm
BF 0.12 Hz
RGAIN 70

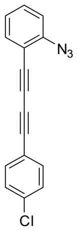

1e

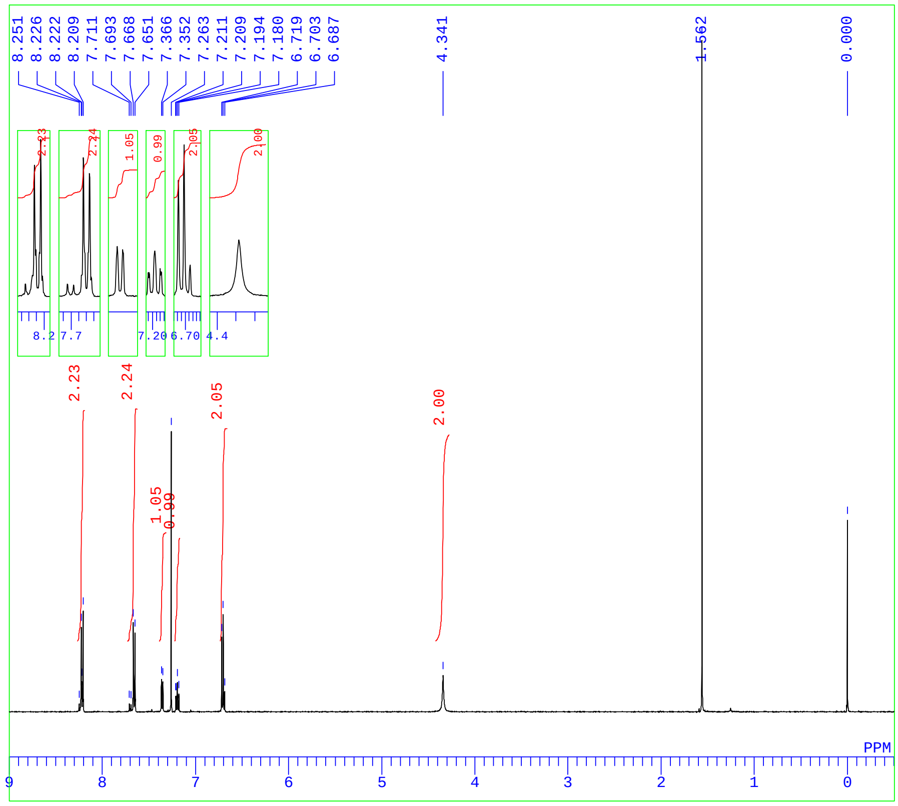

DFILE	A137.als
COMNT	single_pulse
DATIM	2018-04-25 19:54:36
OBNUC	1H
EXMOD	single_pulse.ex2
OBFRQ	500.16 MHz
OBSET	2.41 KHz
OBFIN	6.01 Hz
POINT	13107
FREQU	7507.39 Hz
SCANS	8
ACQTM	1.7459 sec
PD	5.0000 sec
PW1	7.15 usec
IRNUC	1H
CTEMP	21.2 c
SLVNT	CDCL3
EXREF	0.00 ppm
BF	0.12 Hz
RGAIN	52

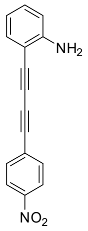

S13

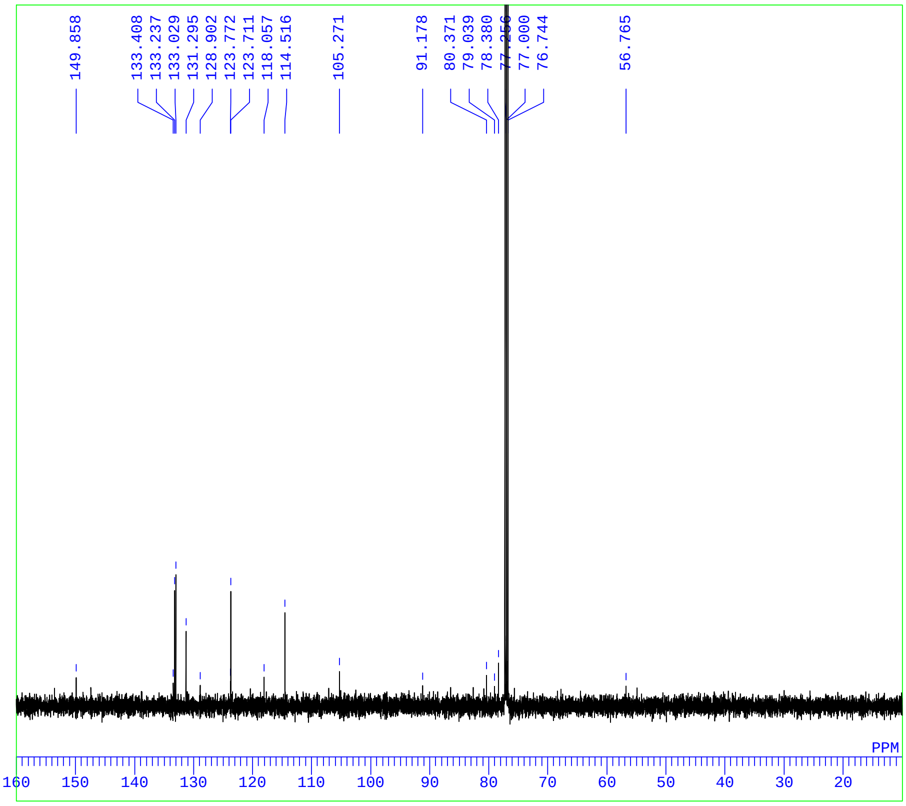

DFILE	A137 C.als
COMNT	
DATIM	2018-04-26 08:01:47
OBNUC	13C
EXMOD	single_pulse_dec
OBFRQ	125.77 MHz
OBSET	7.87 KHz
OBFIN	4.21 Hz
POINT	16384
FREQU	25163.56 Hz
SCANS	2200
ACQTM	0.6511 sec
PD	1.0000 sec
PW1	3.67 usec
IRNUC	1H
CTEMP	22.2 c
SLVNT	CDCL3
EXREF	77.00 ppm
BF	0.12 Hz
RGAIN	70

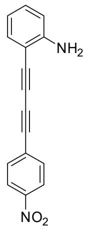

S13

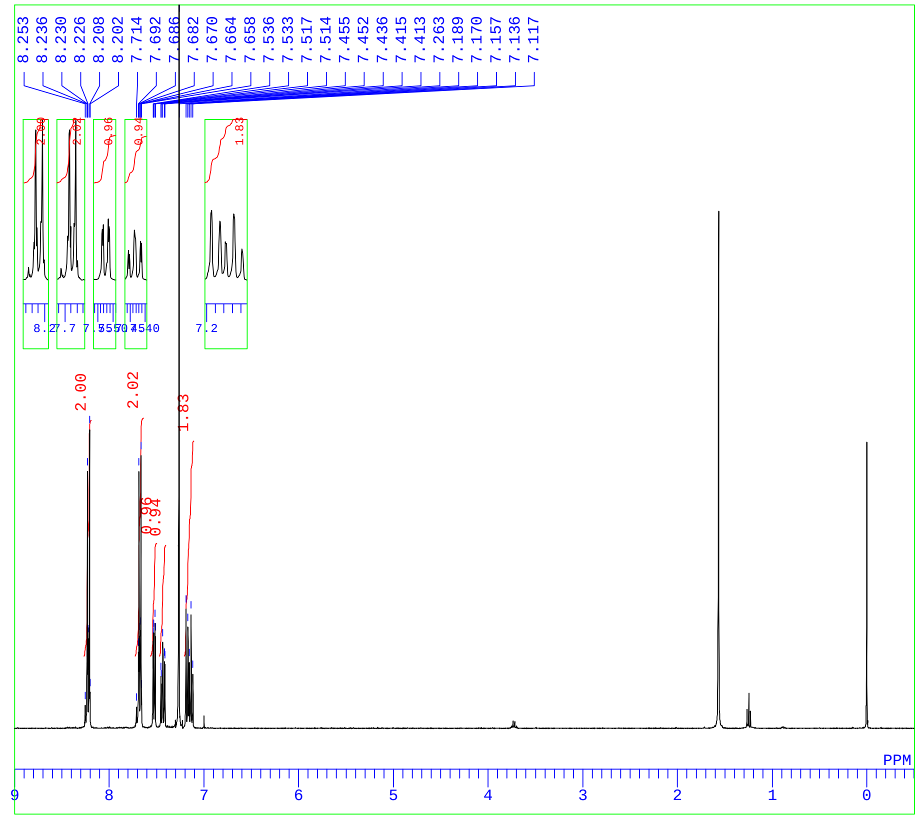

DFILE A143.als
COMNT single_pulse
DATIM 2018-04-26 12:15:14
OBNUC 1H
EXMOD proton.jxp
OBFRQ 399.78 MHz
OBSET 4.19 KHz
OBFIN 7.29 Hz
POINT 13107
FREQU 7621.95 Hz
SCANS 8
ACQTM 1.7197 sec
PD 5.0000 sec
PW1 3.15 usec
IRNUC 1H
CTEMP 20.1 c
SLVNT CDCL3
EXREF 0.00 ppm
BF 0.12 Hz
RGAIN 44

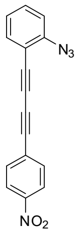

1f

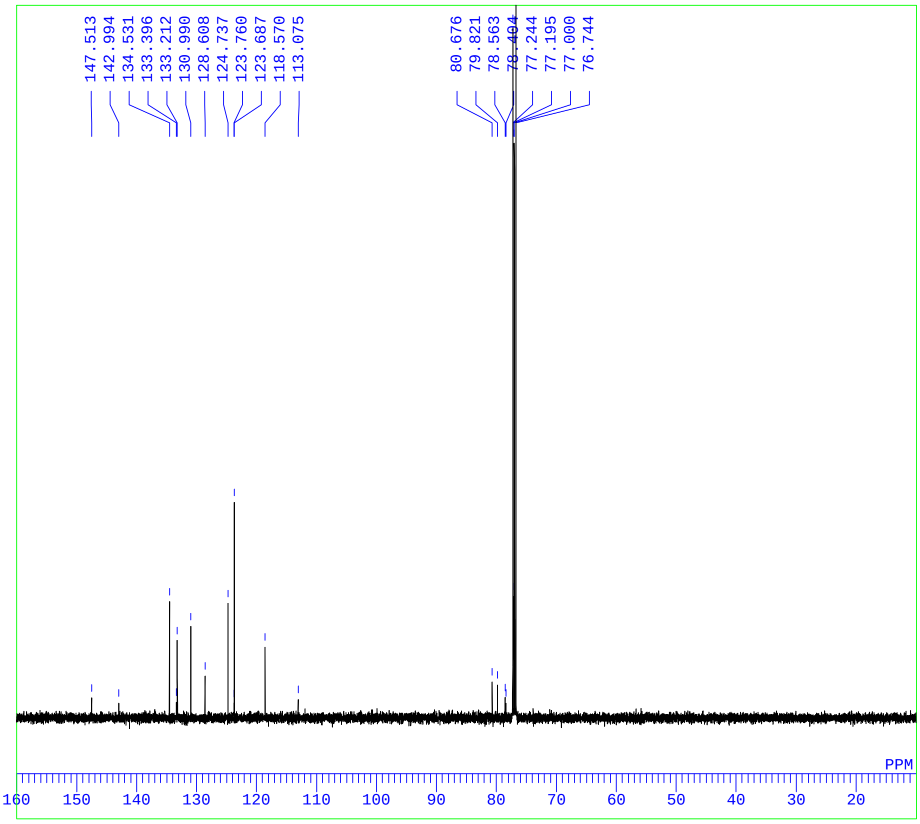

DFILE A143 c.als
COMNT
DATIM 2018-04-26 16:32:13
OBNUC 13C
EXMOD single_pulse_dec
OBFRQ 125.77 MHz
OBSET 7.87 KHz
OBFIN 4.21 Hz
POINT 16384
FREQU 25163.56 Hz
SCANS 2118
ACQTM 0.6511 sec
PD 1.0000 sec
PW1 3.67 usec
IRNUC 1H
CTEMP 21.7 c
SLVNT CDCL3
EXREF 77.00 ppm
BF 0.12 Hz
RGAIN 70

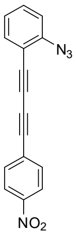

1f

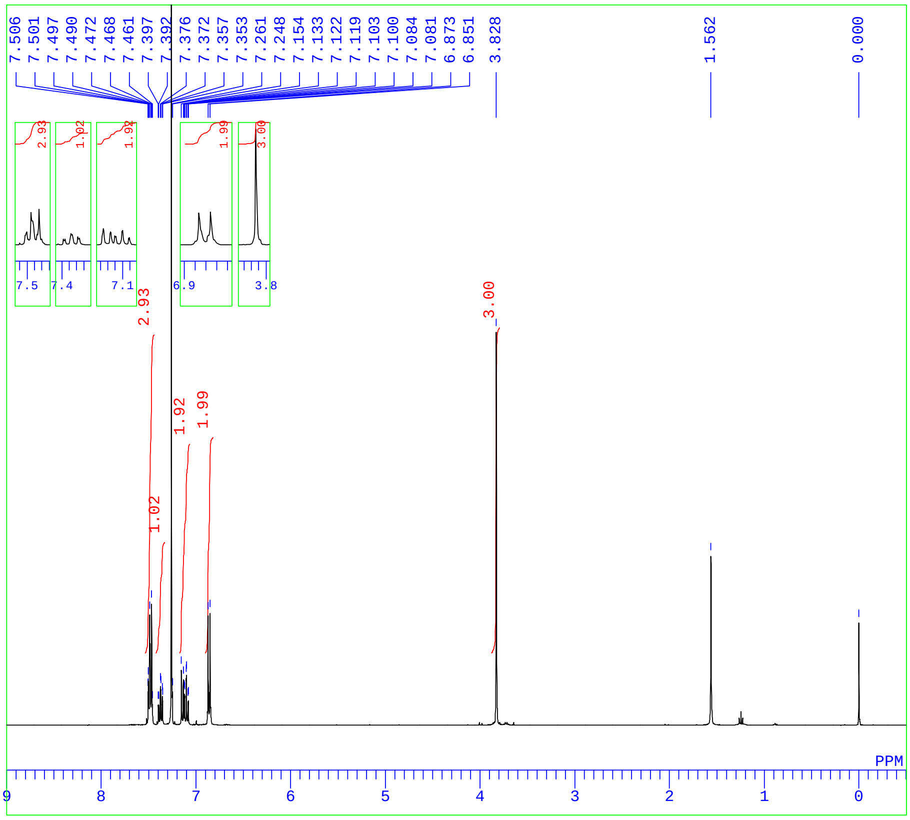

DFILE	A72.als
COMNT	single_pulse
DATIM	2018-04-26 12:19:50
OBNUC	1H
EXMOD	proton.jxp
OBFRQ	399.78 MHz
OBSET	4.19 KHz
OBFIN	7.29 Hz
POINT	13107
FREQU	7621.95 Hz
SCANS	8
ACQTM	1.7197 sec
PD	5.0000 sec
PW1	3.15 usec
IRNUC	1H
CTEMP	20.4 c
SLVNT	CDCL3
EXREF	0.00 ppm
BF	0.12 Hz
RGAIN	42

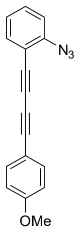

1g

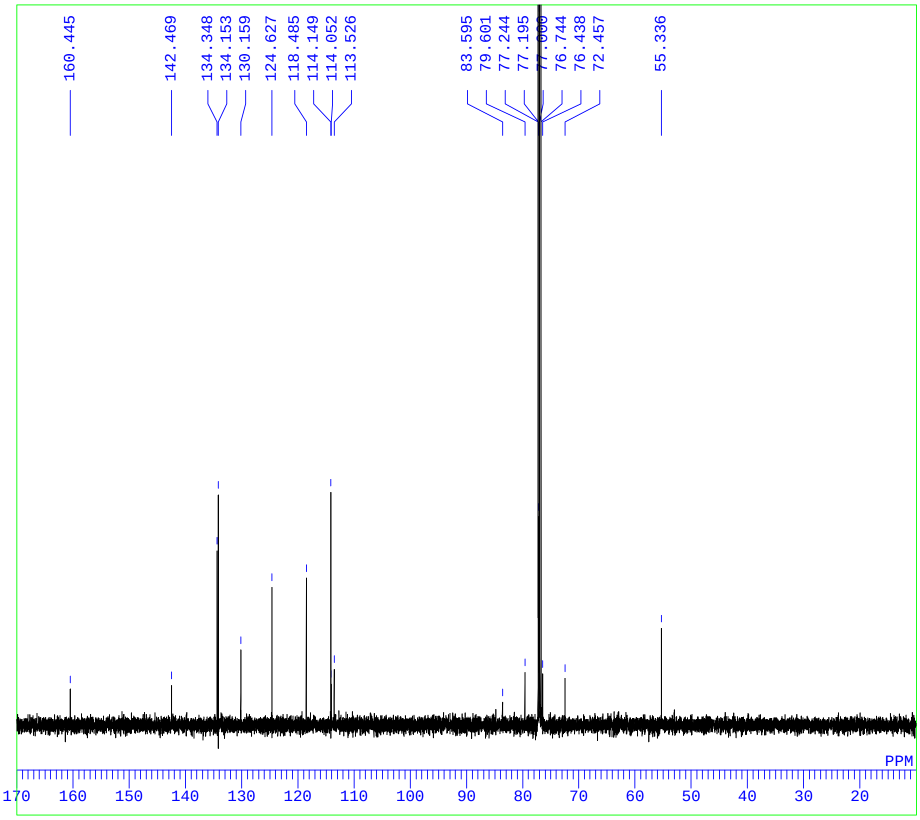

DFILE	A72 C.als
COMNT	
DATIM	2018-04-26 13:26:10
OBNUC	13C
EXMOD	single_pulse_dec
OBFRQ	125.77 MHz
OBSET	7.87 KHz
OBFIN	4.21 Hz
POINT	16384
FREQU	25163.56 Hz
SCANS	1380
ACQTM	0.6511 sec
PD	1.0000 sec
PW1	3.67 usec
IRNUC	1H
CTEMP	21.6 c
SLVNT	CDCL3
EXREF	77.00 ppm
BF	0.12 Hz
RGAIN	70

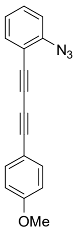

1g

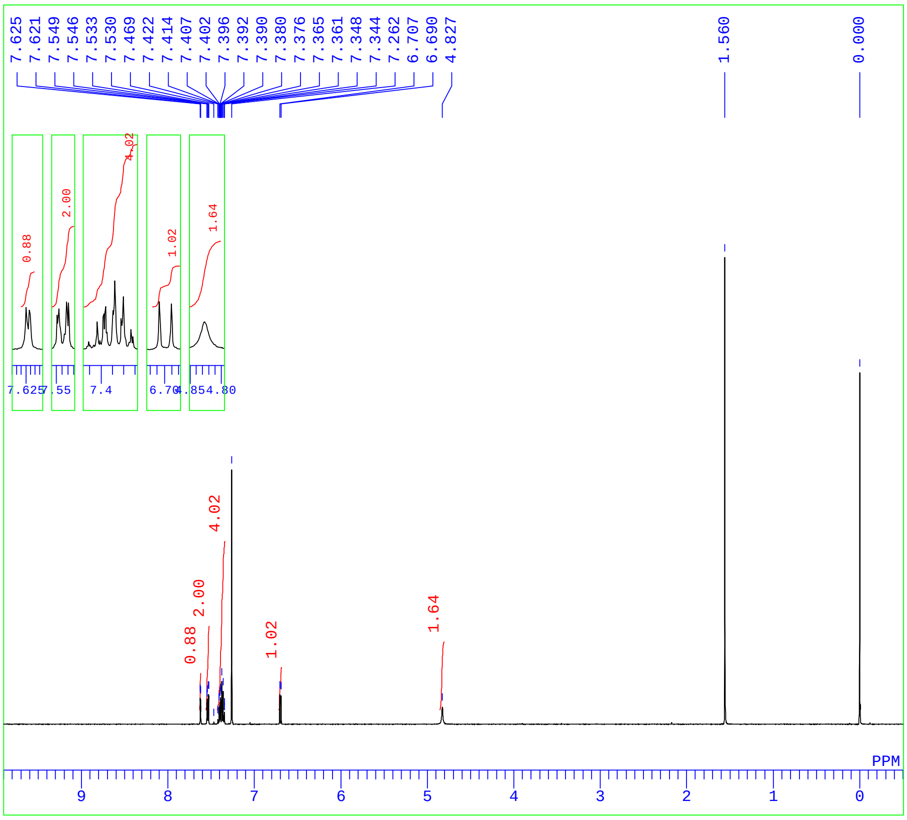

DFILE C-39 H-1.als
COMNT single_pulse
DATIM 2017-07-11 11:19:47
OBNUC 1H
EXMOD single_pulse.ex2
OBFRQ 500.16 MHz
OBSET 2.41 KHz
OBFIN 6.01 Hz
POINT 13107
FREQU 7507.39 Hz
SCANS 8
ACQTM 1.7459 sec
PD 5.0000 sec
PW1 7.15 usec
IRNUC 1H
CTEMP 20.7 c
SLVNT CDCL3
EXREF 0.00 ppm
BF 0.12 Hz
RGAIN 54

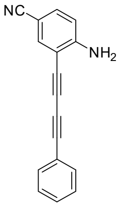

S17

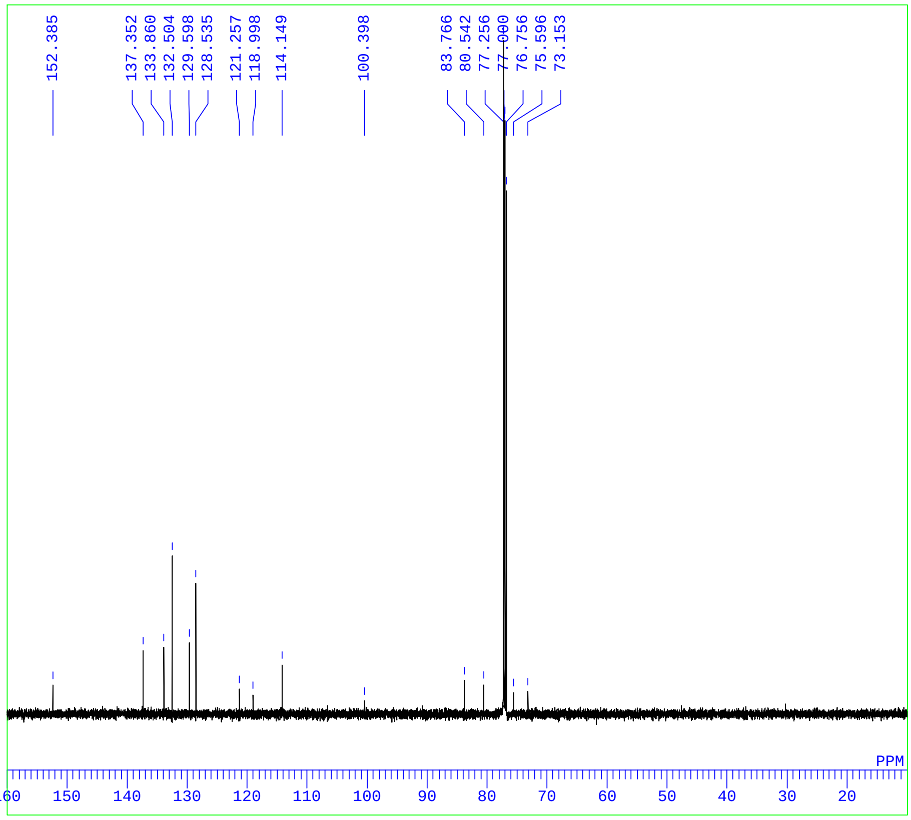

DFILE C39 C.als
COMNT
DATIM 2018-04-26 09:11:22
OBNUC 13C
EXMOD single_pulse_dec
OBFRQ 125.77 MHz
OBSET 7.87 KHz
OBFIN 4.21 Hz
POINT 16384
FREQU 25163.56 Hz
SCANS 2200
ACQTM 0.6511 sec
PD 1.0000 sec
PW1 3.67 usec
IRNUC 1H
CTEMP 22.5 c
SLVNT CDCL3
EXREF 77.00 ppm
BF 0.12 Hz
RGAIN 70

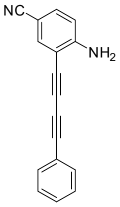

S17

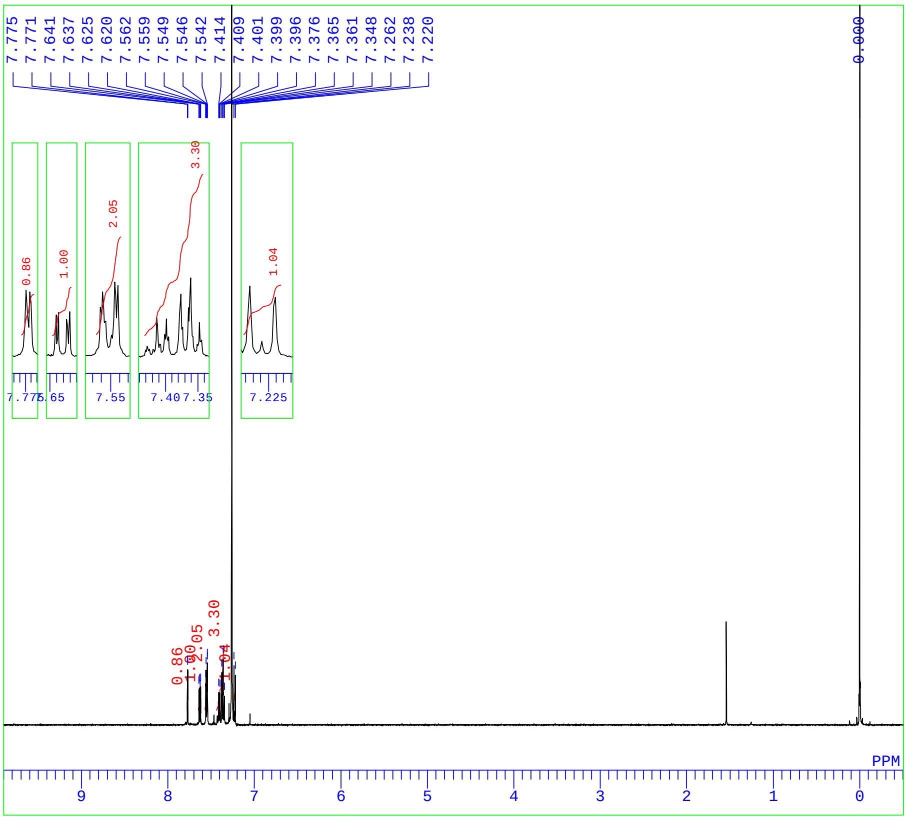

DFILE C-40 H 1 11-1.als
COMNT single_pulse
DATIM 2017-01-11 12:50:07
OBNUC 1H
EXMOD single_pulse.ex2
OBFRQ 500.16 MHz
OBSET 2.41 KHz
OBFIN 6.01 Hz
POINT 13107
FREQU 7507.39 Hz
SCANS 8
ACQTM 1.7459 sec
PD 5.0000 sec
PW1 7.15 usec
IRNUC 1H
CTEMP 20.6 c
SLVNT CDCL3
EXREF 0.00 ppm
BF 0.12 Hz
RGAIN 54

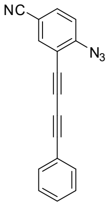

1h

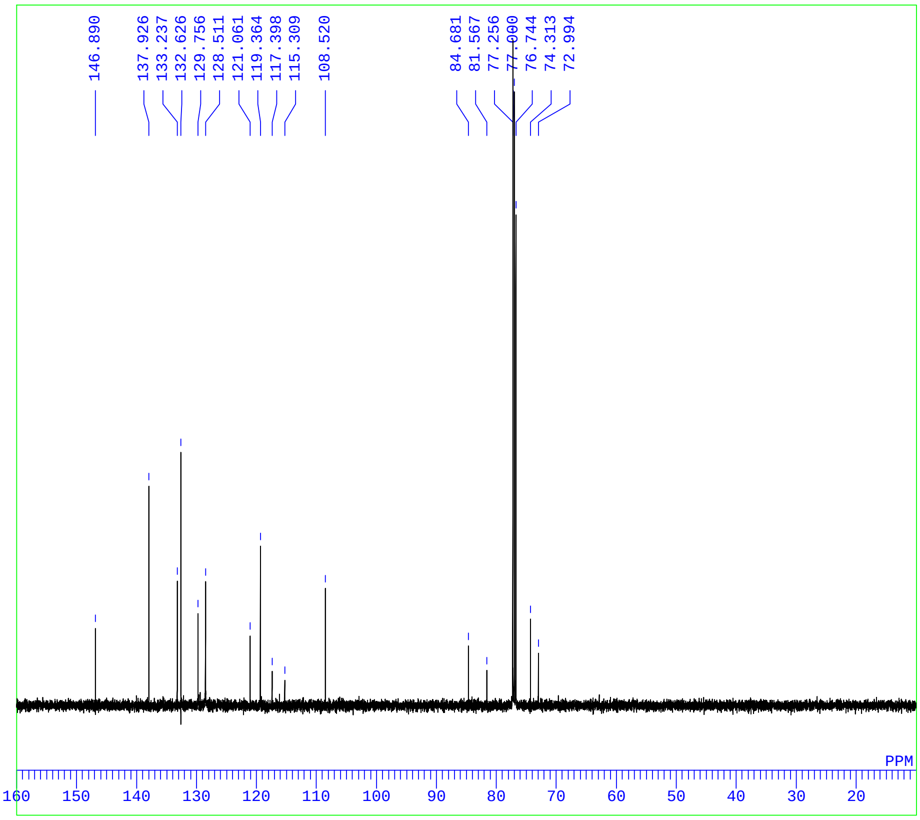

DFILE C40 C.als
COMNT
DATIM 2018-04-26 09:54:13
OBNUC 13C
EXMOD single_pulse_dec
OBFRQ 125.77 MHz
OBSET 7.87 KHz
OBFIN 4.21 Hz
POINT 16384
FREQU 25163.56 Hz
SCANS 1204
ACQTM 0.6511 sec
PD 1.0000 sec
PW1 3.67 usec
IRNUC 1H
CTEMP 21.6 c
SLVNT CDCL3
EXREF 77.00 ppm
BF 0.12 Hz
RGAIN 70

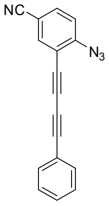

1h

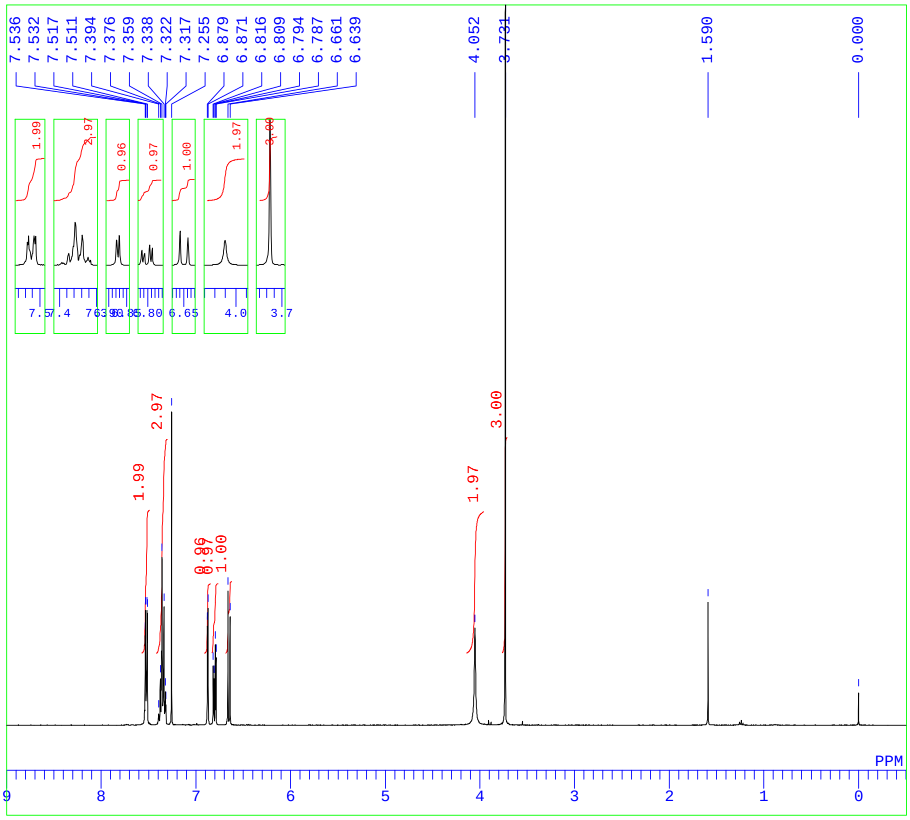

DFILE	A191.als
COMNT	single_pulse
DATIM	2018-04-26 12:23:27
OBNUC	1H
EXMOD	proton.jxp
OBFRQ	399.78 MHz
OBSET	4.19 KHz
OBFIN	7.29 Hz
POINT	13107
FREQU	7621.95 Hz
SCANS	8
ACQTM	1.7197 sec
PD	5.0000 sec
PW1	3.15 usec
IRNUC	1H
CTEMP	20.3 c
SLVNT	CDCL3
EXREF	0.00 ppm
BF	0.12 Hz
RGAIN	32

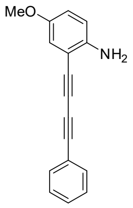

S19

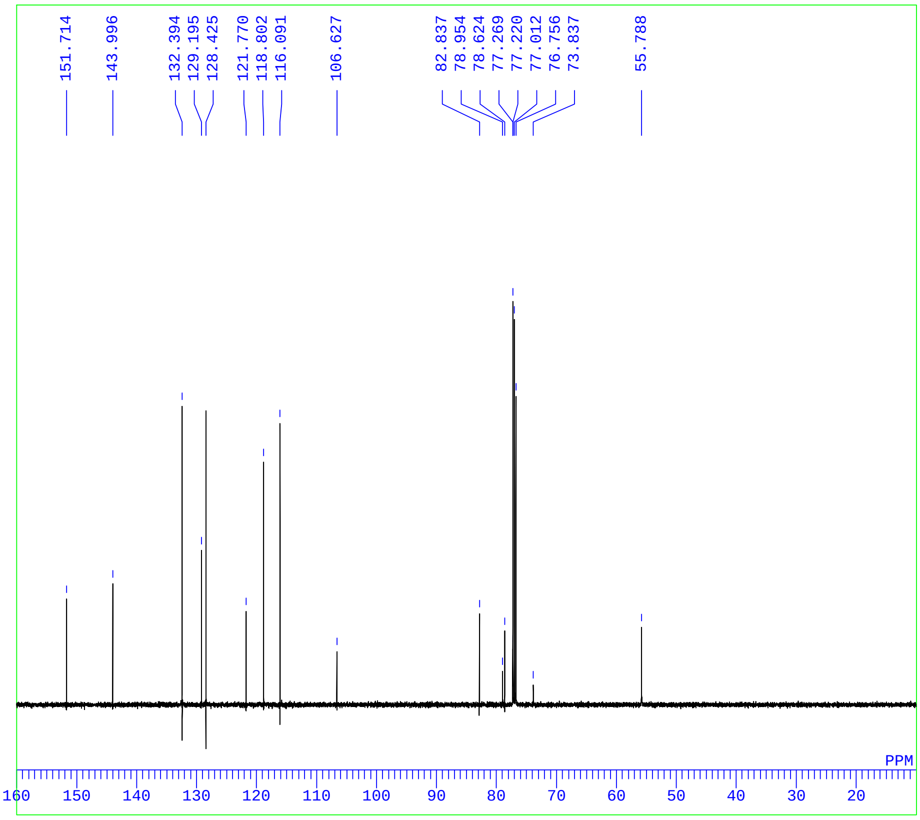

DFILE	A191 C.als
COMNT	
DATIM	2018-04-26 18:32:48
OBNUC	13C
EXMOD	single_pulse_dec
OBFRQ	125.77 MHz
OBSET	7.87 KHz
OBFIN	4.21 Hz
POINT	16384
FREQU	25163.56 Hz
SCANS	2021
ACQTM	0.6511 sec
PD	1.0000 sec
PW1	3.67 usec
IRNUC	1H
CTEMP	21.3 c
SLVNT	CDCL3
EXREF	77.00 ppm
BF	0.12 Hz
RGAIN	68

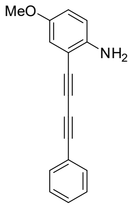

S19

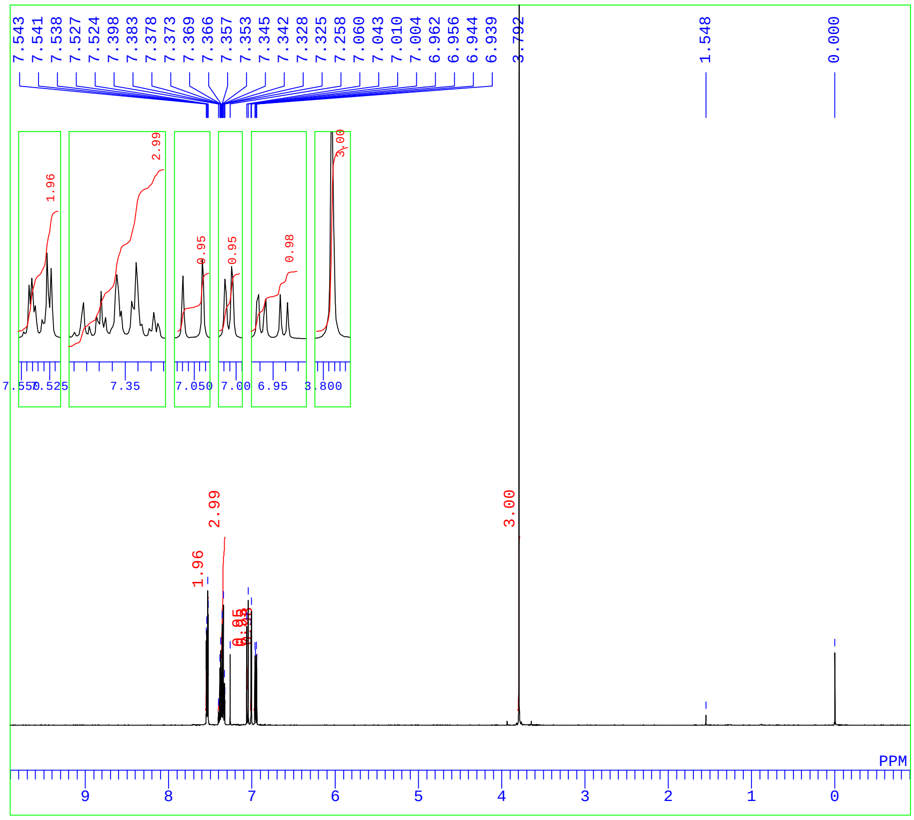

DFILE A-192 H.als
COMNT single_pulse
DATIM 2016-02-12 15:55:24
OBNUC 1H
EXMOD single_pulse.ex2
OBFRQ 500.16 MHz
OBSET 2.41 KHz
OBFIN 6.01 Hz
POINT 13107
FREQU 7507.39 Hz
SCANS 8
ACQTM 1.7459 sec
PD 5.0000 sec
PW1 6.82 usec
IRNUC 1H
CTEMP 20.0 c
SLVNT CDCL3
EXREF 0.00 ppm
BF 0.12 Hz
RGAIN 46

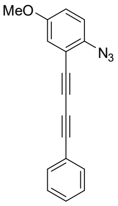

1i

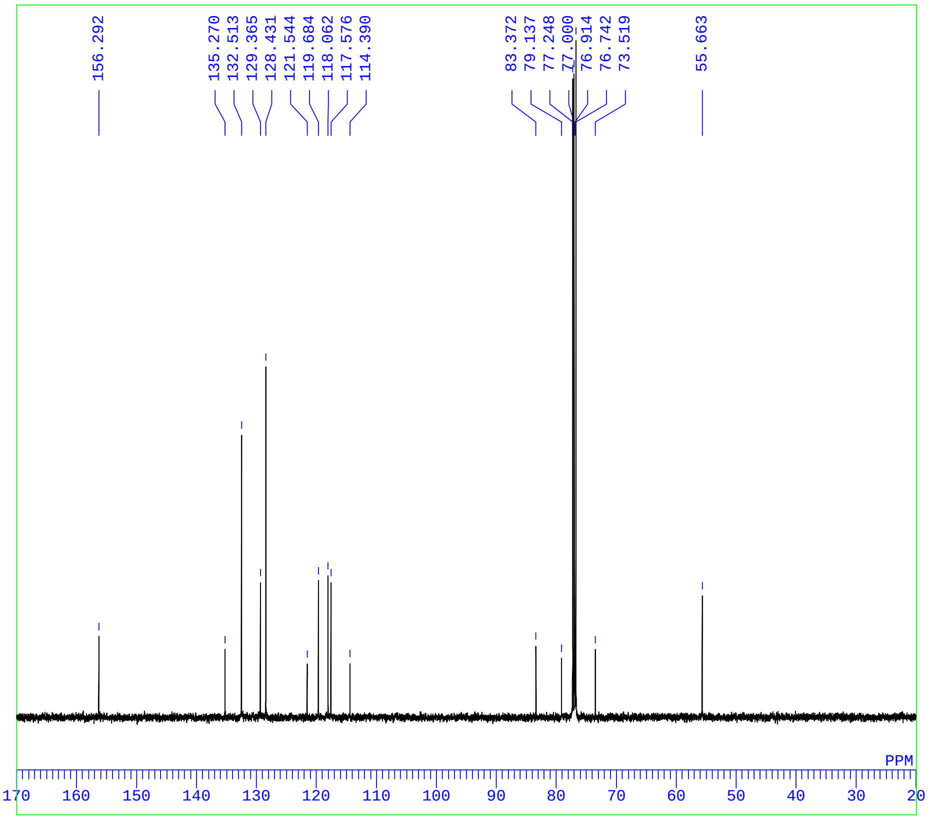

DFILE A-192 C-1.als
COMNT single pulse decoupled gat
DATIM 2016-02-12 16:25:56
OBNUC 13C
EXMOD single_pulse_dec
OBFRQ 125.77 MHz
OBSET 7.87 KHz
OBFIN 4.21 Hz
POINT 26214
FREQU 31446.06 Hz
SCANS 632
ACQTM 0.8336 sec
PD 2.0000 sec
PW1 3.50 usec
IRNUC 1H
CTEMP 20.6 c
SLVNT CDCL3
EXREF 77.00 ppm
BF 1.20 Hz
RGAIN 54

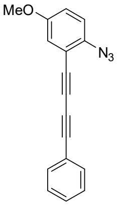

1i

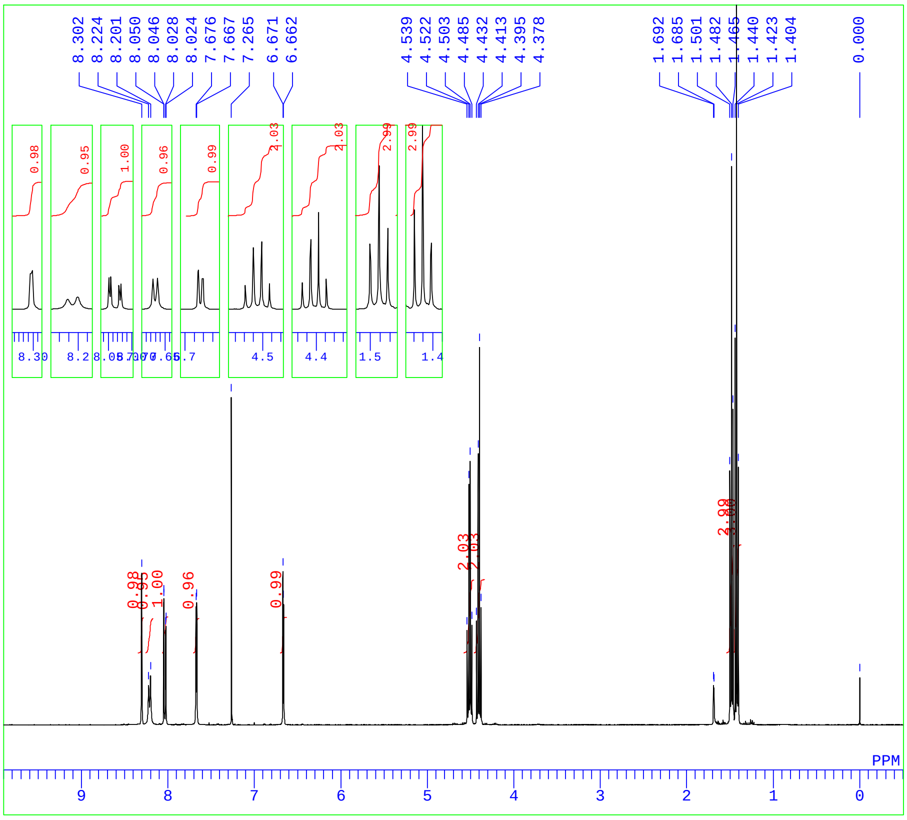

DFILE B176.als
COMNT single_pulse
DATIM 2018-04-28 11:36:26
OBNUC 1H
EXMOD proton.jxp
OBFRQ 399.78 MHz
OBSET 4.19 KHz
OBFIN 7.29 Hz
POINT 13107
FREQU 7598.78 Hz
SCANS 8
ACQTM 1.7249 sec
PD 5.0000 sec
PW1 3.15 usec
IRNUC 1H
CTEMP 20.3 c
SLVNT CDCL3
EXREF 0.00 ppm
BF 0.12 Hz
RGAIN 34

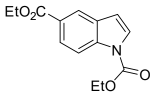

10F

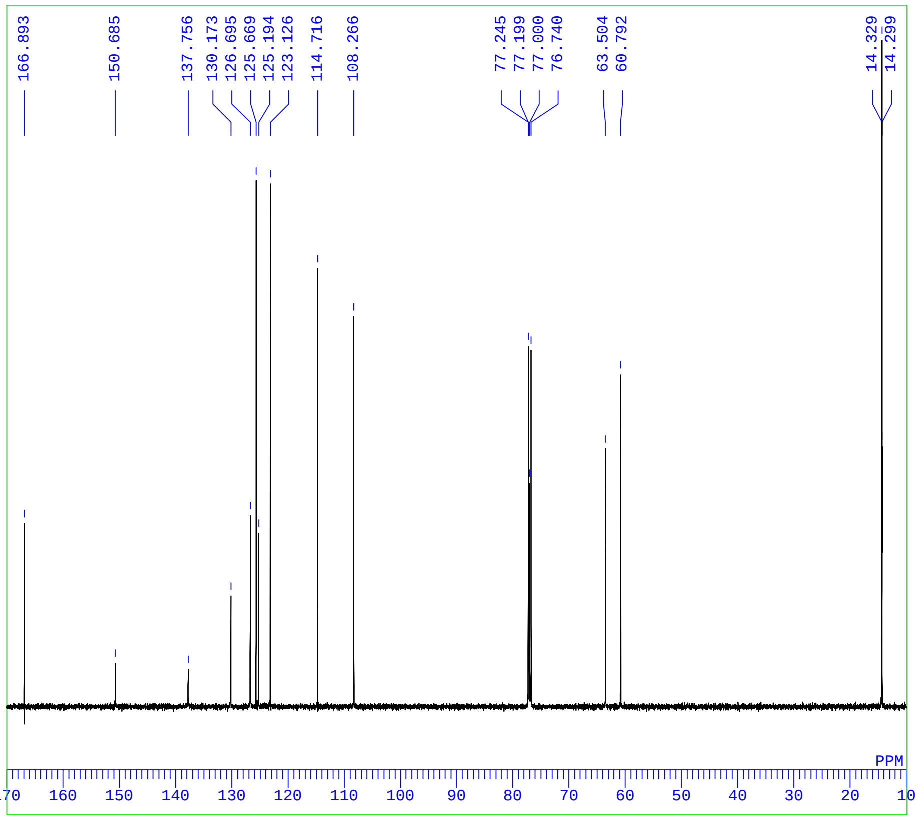

DFILE B176 C.als
COMNT
DATIM 2018-04-28 06:11:27
OBNUC 13C
EXMOD single_pulse_dec
OBFRQ 125.77 MHz
OBSET 7.87 KHz
OBFIN 4.21 Hz
POINT 13107
FREQU 25252.14 Hz
SCANS 2500
ACQTM 0.5190 sec
PD 1.0000 sec
PW1 3.67 usec
IRNUC 1H
CTEMP 21.6 c
SLVNT CDCL3
EXREF 77.00 ppm
BF 0.12 Hz
RGAIN 60

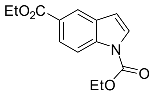

10F

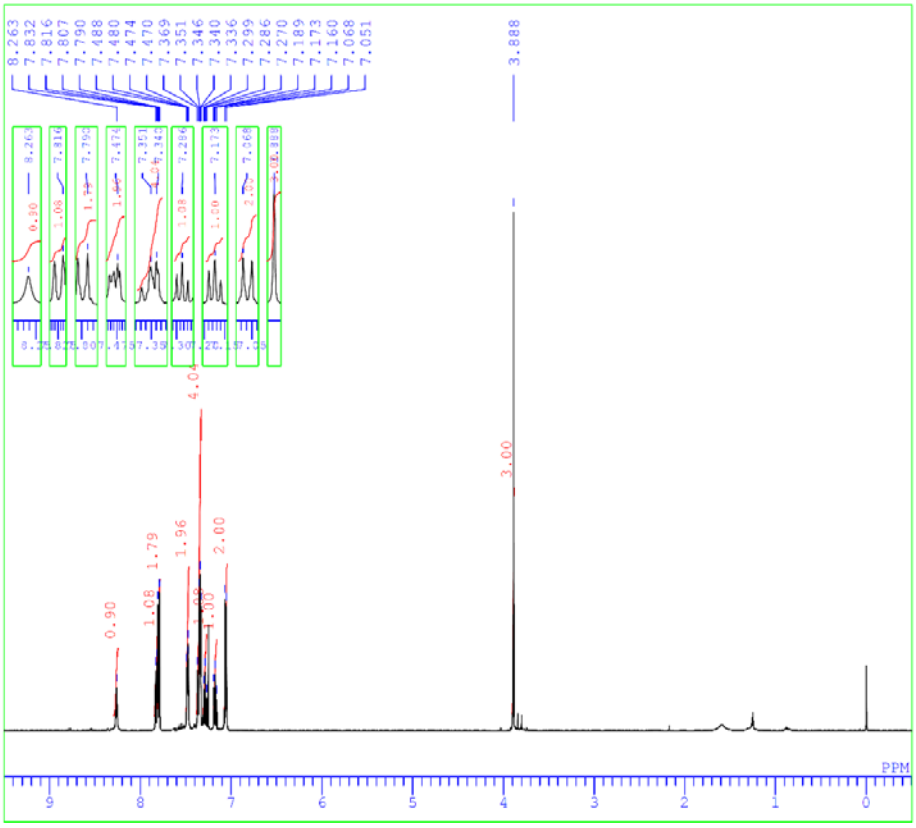

DFILE anisole intermediate h-1.als
COMNT single_pulse
DATIM 2018-01-22 19:27:10
OBNUC 1H
EXMOD single_pulse.ex2
OBFRQ 500.16 MHz
OBSET 2.41 KHz
OBFIN 6.01 Hz
POINT 13107
FREQU 7507.39 Hz
SCANS 8
ACQTM 1.7459 sec
PD 5.0000 sec
PW1 7.15 usec
IRNUC 1H
CTEMP 18.6 c
SLVNT CDCL3
EXREF 0.00 ppm
BF 0.12 Hz
RGAIN 46

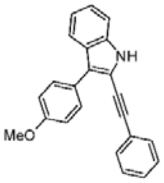

2aA

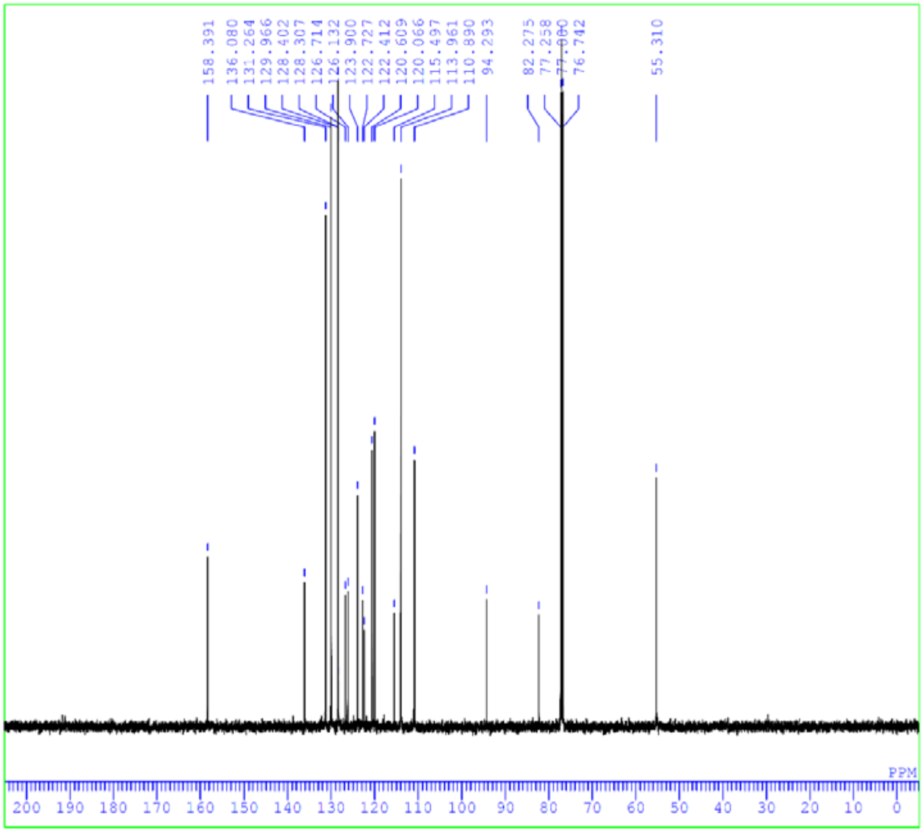

DFILE anisole intermediate c-1.als
COMNT
DATIM 2016-11-04 17:45:14
OBNUC 13C
EXMOD single_pulse_dec
OBFRQ 125.77 MHz
OBSET 7.87 KHz
OBFIN 4.21 Hz
POINT 26214
FREQU 31446.06 Hz
SCANS 464
ACQTM 0.8336 sec
PD 2.0000 sec
PW1 3.67 usec
IRNUC 1H
CTEMP 22.3 c
SLVNT CDCL3
EXREF 77.00 ppm
BF 1.20 Hz
RGAIN 60

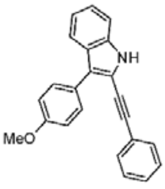

2aA

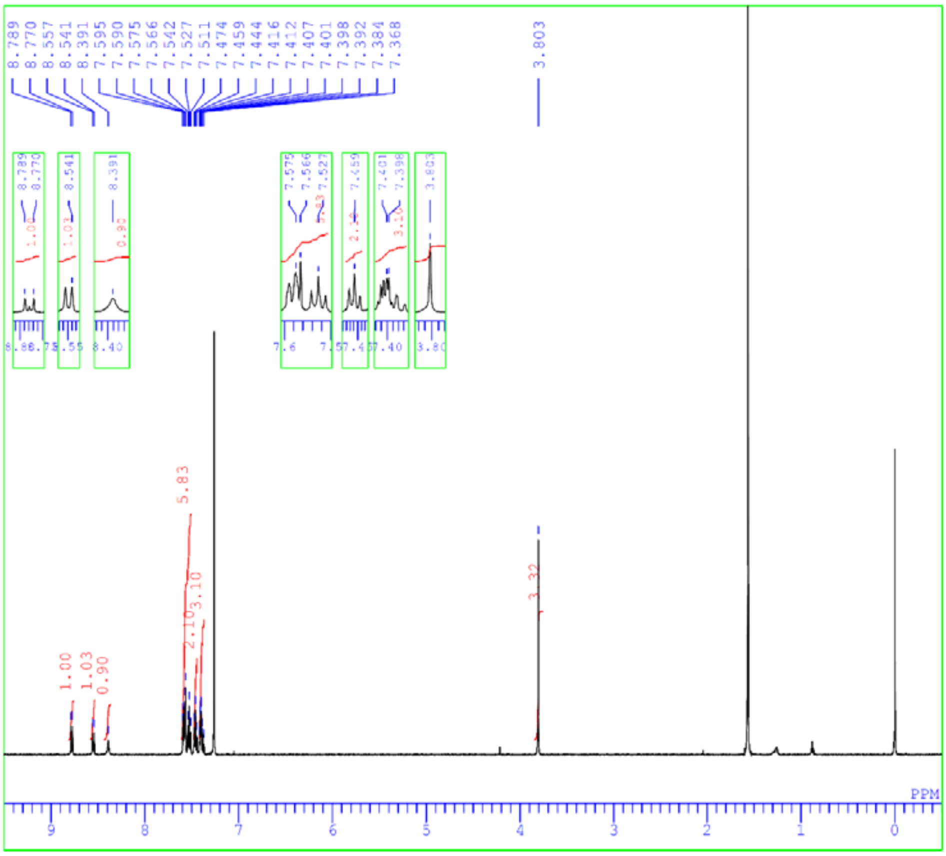

DFILE anisole product h-1.als
COMNT single_pulse
DATIM 2016-06-23 15:36:49
OBNUC 1H
EXMOD single_pulse.ex2
OBFRQ 500.16 MHz
OBSET 2.41 KHz
OBFIN 6.01 Hz
POINT 13107
FREQU 7507.39 Hz
SCANS 8
ACQTM 1.7459 sec
PD 5.0000 sec
PW1 6.50 usec
IRNUC 1H
CTEMP 21.6 c
SLVNT CDCL3
EXREF 0.00 ppm
BF 0.12 Hz
RGAIN 52

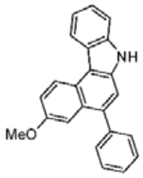

3aA

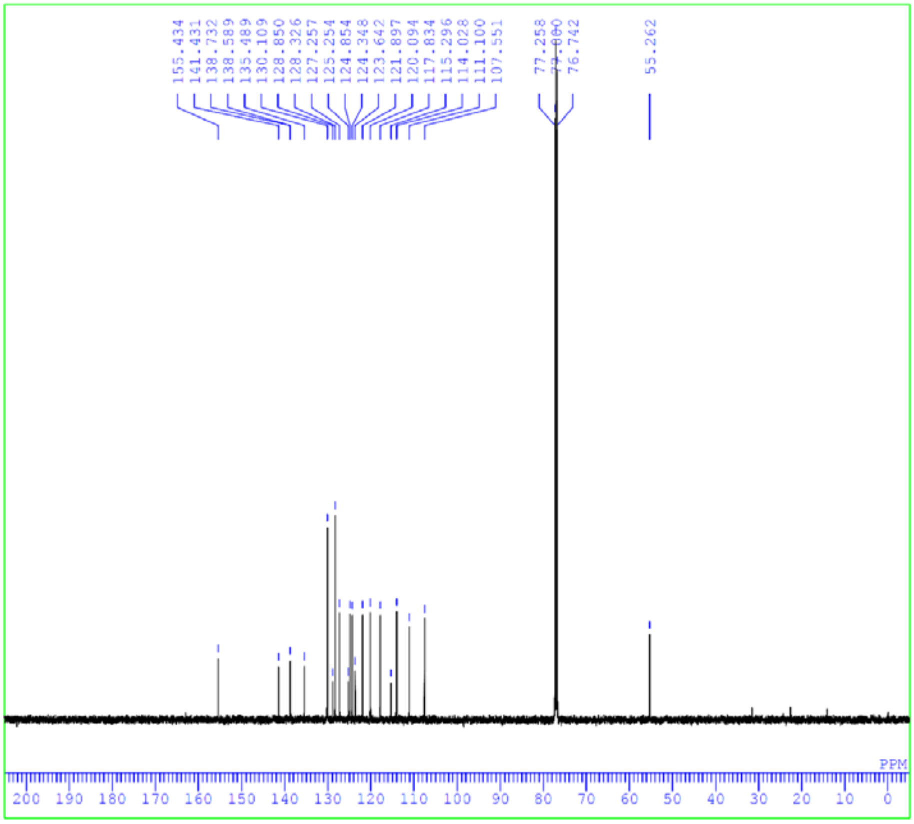

DFILE anisole product c-1.als
COMNT single pulse decoupled gated
DATIM 2016-06-23 17:40:33
OBNUC 13C
EXMOD single_pulse_dec
OBFRQ 125.77 MHz
OBSET 7.87 KHz
OBFIN 4.21 Hz
POINT 26214
FREQU 31446.06 Hz
SCANS 1024
ACQTM 0.8336 sec
PD 2.0000 sec
PW1 3.73 usec
IRNUC 1H
CTEMP 21.6 c
SLVNT CDCL3
EXREF 77.00 ppm
BF 1.20 Hz
RGAIN 54

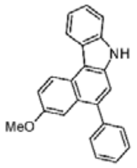

3aA

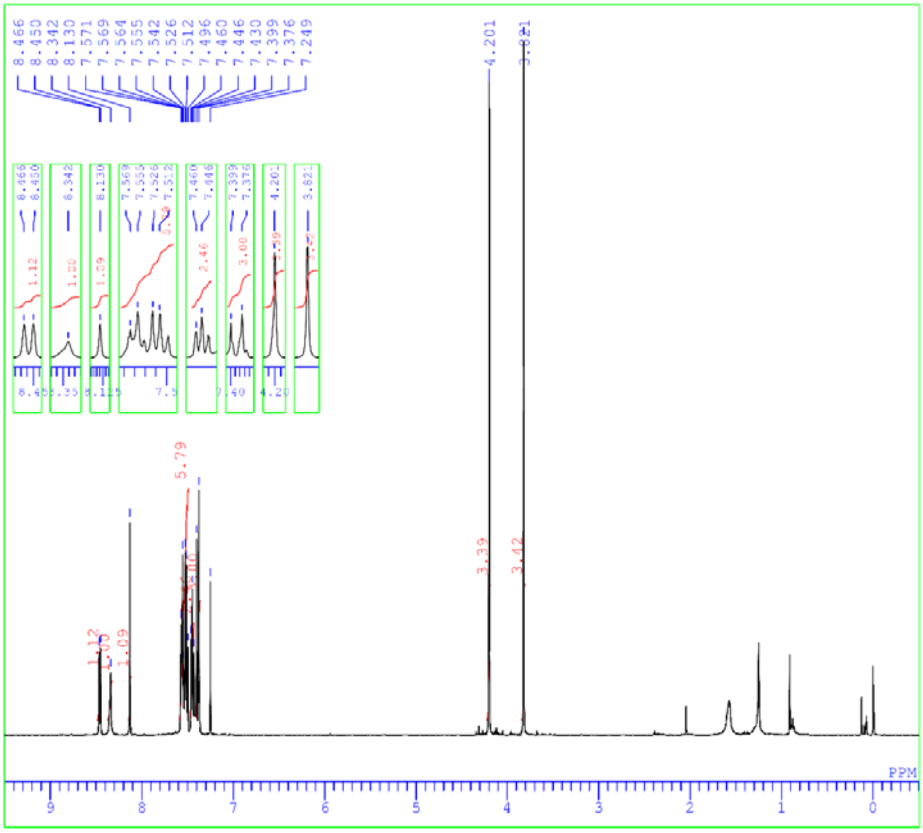

DFILE 1,2-dimethoxybenzene h-1.als
COMNT single_pulse
DATIM 2017-09-15 10:52:02
OBNUC 1H
EXMOD single_pulse.ex2
OBFRQ 500.16 MHz
OBSET 2.41 KHz
OBFIN 6.01 Hz
POINT 13107
FREQU 7507.39 Hz
SCANS 8
ACQTM 1.7459 sec
PD 5.0000 sec
PW1 7.15 usec
IRNUC 1H
CTEMP 21.1 c
SLVNT CDCL3
EXREF 0.00 ppm
BF 0.12 Hz
RGAIN 44

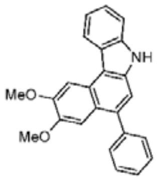

3aB

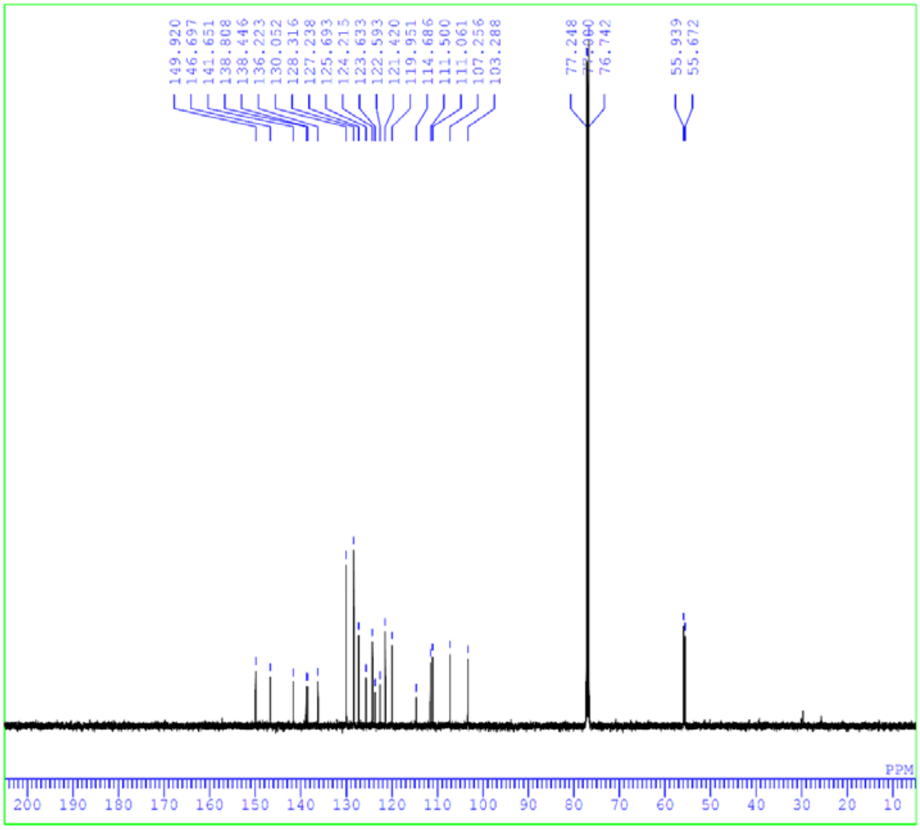

DFILE 1,2-dimethoxybenzene c-1.als
COMNT
DATIM 2017-09-15 11:41:46
OBNUC 13C
EXMOD single_pulse_dec
OBFRQ 125.77 MHz
OBSET 7.87 KHz
OBFIN 4.21 Hz
POINT 26214
FREQU 31446.06 Hz
SCANS 1024
ACQTM 0.8336 sec
PD 2.0000 sec
PW1 3.67 usec
IRNUC 1H
CTEMP 21.7 c
SLVNT CDCL3
EXREF 77.00 ppm
BF 1.20 Hz
RGAIN 60

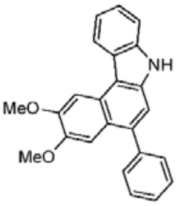

3aB

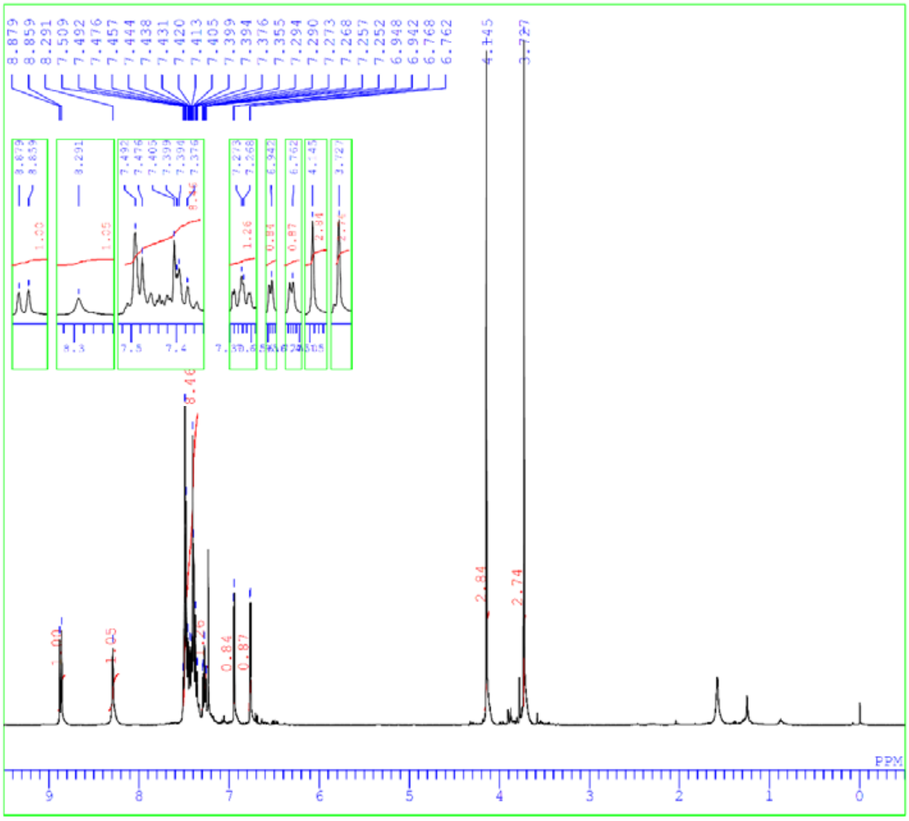
8.879
8.859
8.291
7.509
7.492
7.476
7.457
7.444
7.438
7.431
7.420
7.413
7.405
7.399
7.394
7.376
7.355
7.294
7.290
7.273
7.268
7.257
7.252
6.948
6.942
6.768
6.762
4.145
3.727
PPM
9
8
7
6
5
4
3
2
1
0
DFILE 1,3-dimethoxybenzene_Proton-1.
COMNT single_pulse
DATIM 2016-07-16 11:23:37
OBNUC 1H
EXMOD proton.jxp
OBFRQ 399.78 MHz
OBSET 4.19 KHz
OBFIN 7.29 Hz
POINT 13107
FREQU 7598.78 Hz
SCANS 8
ACQTM 1.7249 sec
PD 5.0000 sec
PW1 3.06 usec
IRNUC 1H
CTEMP 19.7 c
SLVNT CDCL3
EXREF 0.00 ppm
BF 0.12 Hz
RGAIN 36

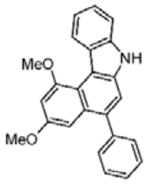

3aC

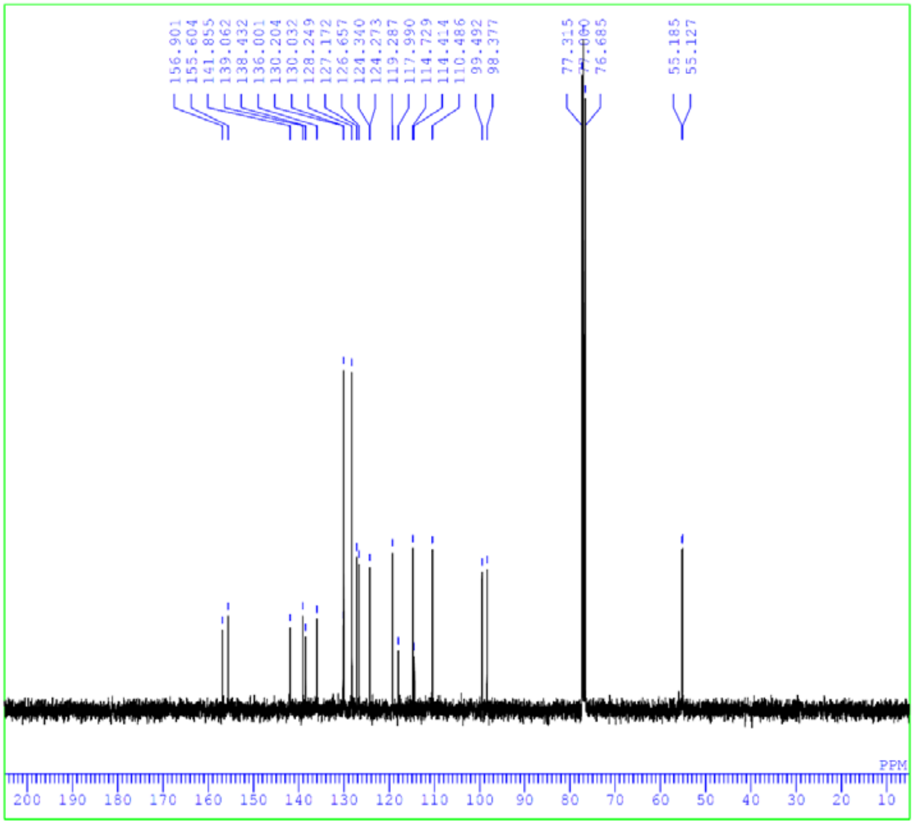
156.901
155.604
141.855
139.062
138.432
136.001
130.204
130.032
128.249
127.172
126.657
124.340
124.273
119.287
117.990
114.729
114.414
110.486
99.492
98.377
77.315
77.000
76.685
55.185
55.127
PPM
200
190
180
170
160
150
140
130
120
110
100
90
80
70
60
50
40
30
20
10
DFILE 1,3-dimethoxybenzene_Carbon-1.
COMNT single pulse decoupled gated
DATIM 2016-07-21 15:36:46
OBNUC 13C
EXMOD carbon.jxp
OBFRQ 100.53 MHz
OBSET 5.35 KHz
OBFIN 5.86 Hz
POINT 26214
FREQU 25125.63 Hz
SCANS 128
ACQTM 1.0433 sec
PD 2.0000 sec
PW1 3.77 usec
IRNUC 1H
CTEMP 19.7 c
SLVNT CDCL3
EXREF 77.00 ppm
BF 1.20 Hz
RGAIN 50

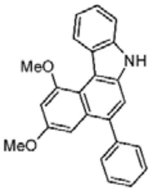

3aC

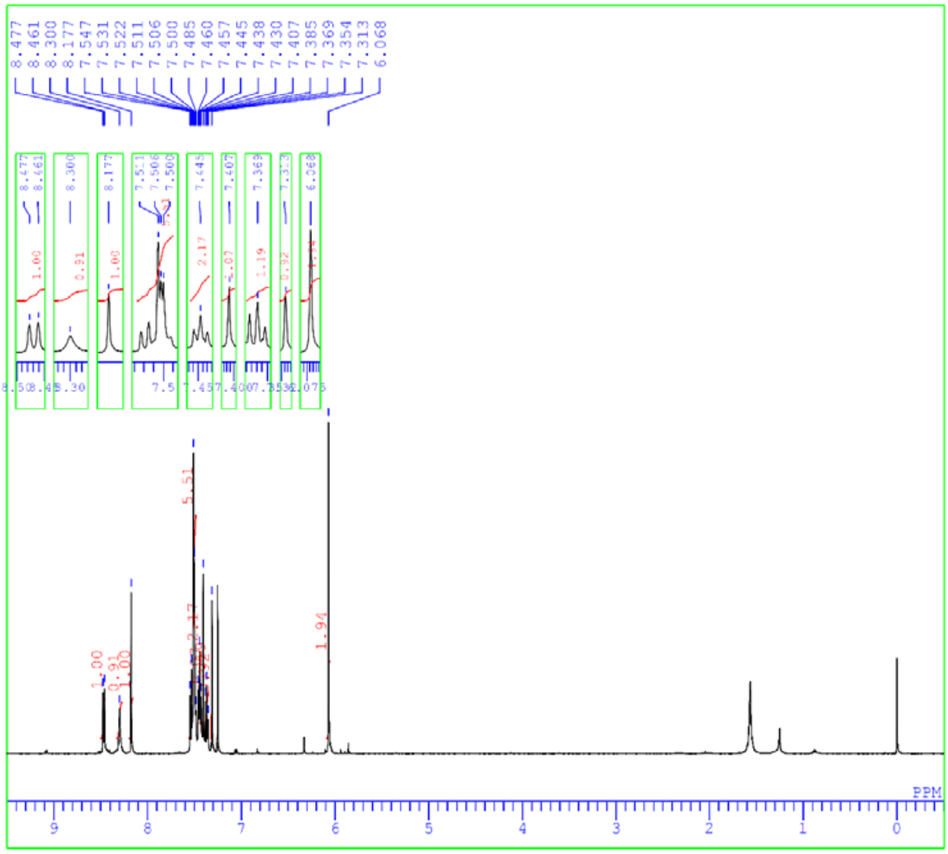


```
DFILE   1,3-benzodioxole h-1.als
COMNT   single_pulse
DATIM   2016-11-02 17:10:21
OBNUC   1H
EXMOD   single_pulse.ex2
OBFRQ        500.16 MHz
OBSET          2.41 KHz
OBFIN          6.01 Hz
POINT         13107
FREQU       7507.39 Hz
SCANS             8
ACQTM        1.7459 sec
PD           5.0000 sec
PW1            7.15 usec
IRNUC   1H
CTEMP          21.7 c
SLVNT   CDCL3
EXREF          0.00 ppm
BF             0.12 Hz
RGAIN            48
```

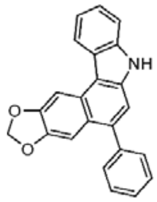

3aD

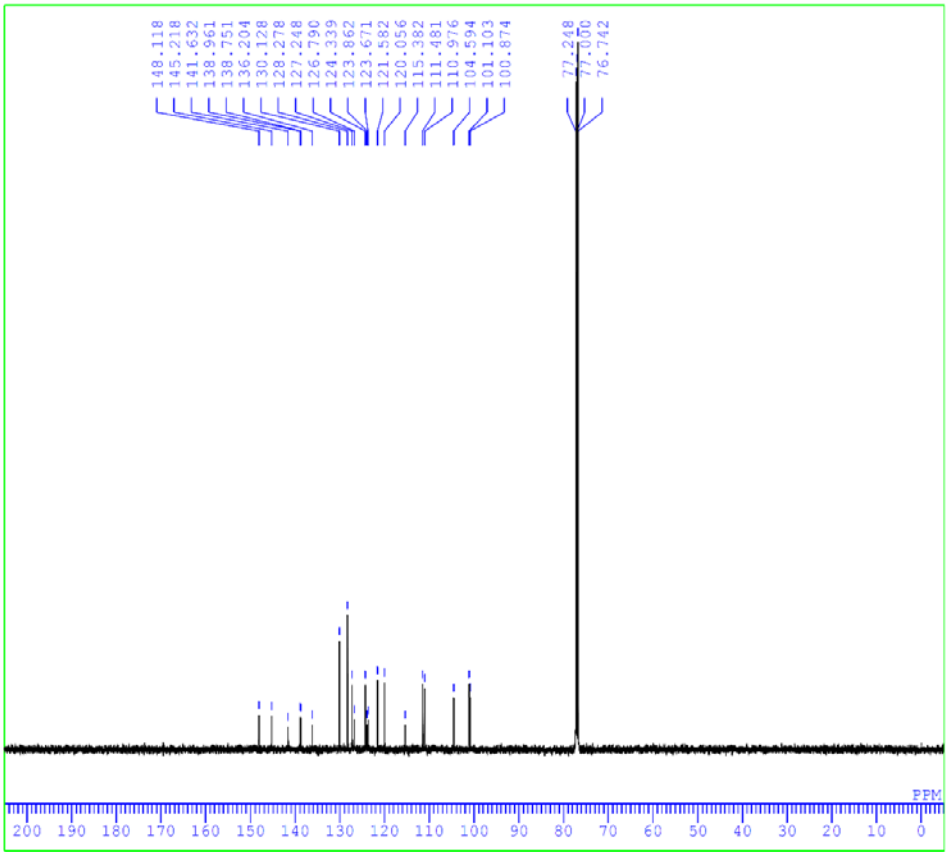


```
DFILE   1,3-benzodioxole c-1.als
COMNT
DATIM   2016-11-02 18:01:44
OBNUC   13C
EXMOD   single_pulse_dec
OBFRQ        125.77 MHz
OBSET          7.87 KHz
OBFIN          4.21 Hz
POINT         26214
FREQU      31446.06 Hz
SCANS          1024
ACQTM        0.8336 sec
PD           2.0000 sec
PW1            3.67 usec
IRNUC   1H
CTEMP          22.2 c
SLVNT   CDCL3
EXREF         77.00 ppm
BF             1.20 Hz
RGAIN            58
```

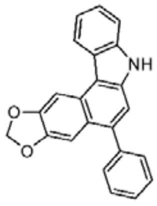

3aD

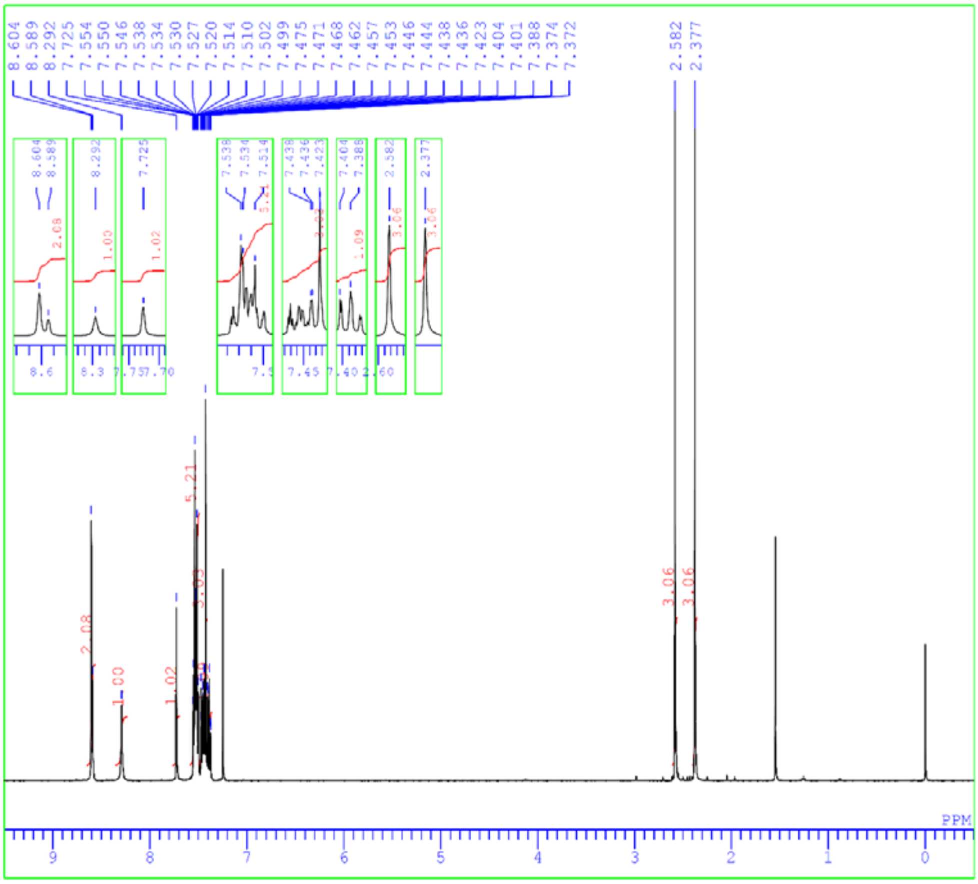


```
DFILE  o-xylene h-1.als
COMNT  single_pulse
DATIM  2018-03-08 16:12:04
OBNUC  1H
EXMOD  single_pulse.ex2
OBFRQ       500.16 MHz
OBSET         2.41 KHz
OBFIN         6.01 Hz
POINT        13107
FREQU      7507.39 Hz
SCANS            8
ACQTM       1.7459 sec
PD          5.0000 sec
PW1           7.15 usec
IRNUC  1H
CTEMP         20.2 c
SLVNT  CDCL3
EXREF         0.00 ppm
BF            0.12 Hz
RGAIN           46
```

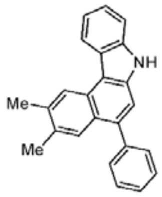

3aE

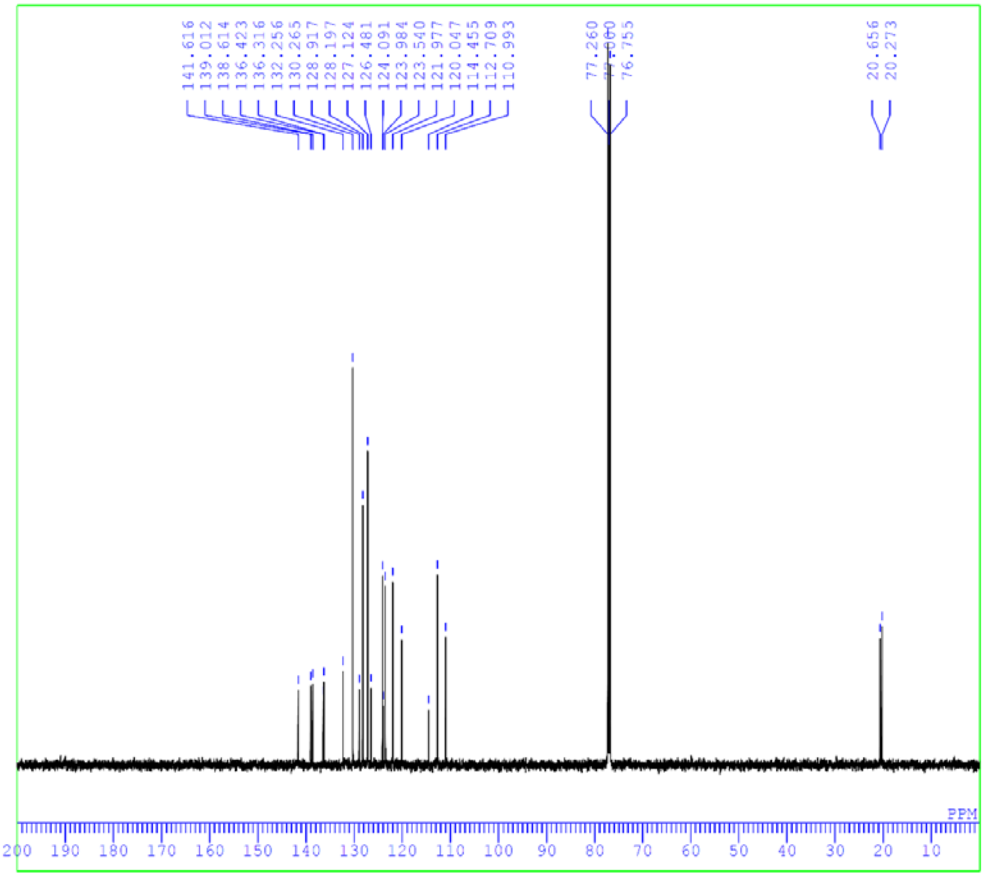


```
DFILE  o-xylene c-1.als
COMNT
DATIM  2018-03-08 17:05:18
OBNUC  13C
EXMOD  single_pulse_dec
OBFRQ       125.77 MHz
OBSET         7.87 KHz
OBFIN         4.21 Hz
POINT        13107
FREQU     25252.14 Hz
SCANS         2077
ACQTM       0.5190 sec
PD          1.0000 sec
PW1           3.67 usec
IRNUC  1H
CTEMP         20.6 c
SLVNT  CDCL3
EXREF        77.00 ppm
BF            1.20 Hz
RGAIN           54
```

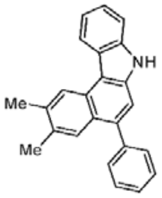

3aE

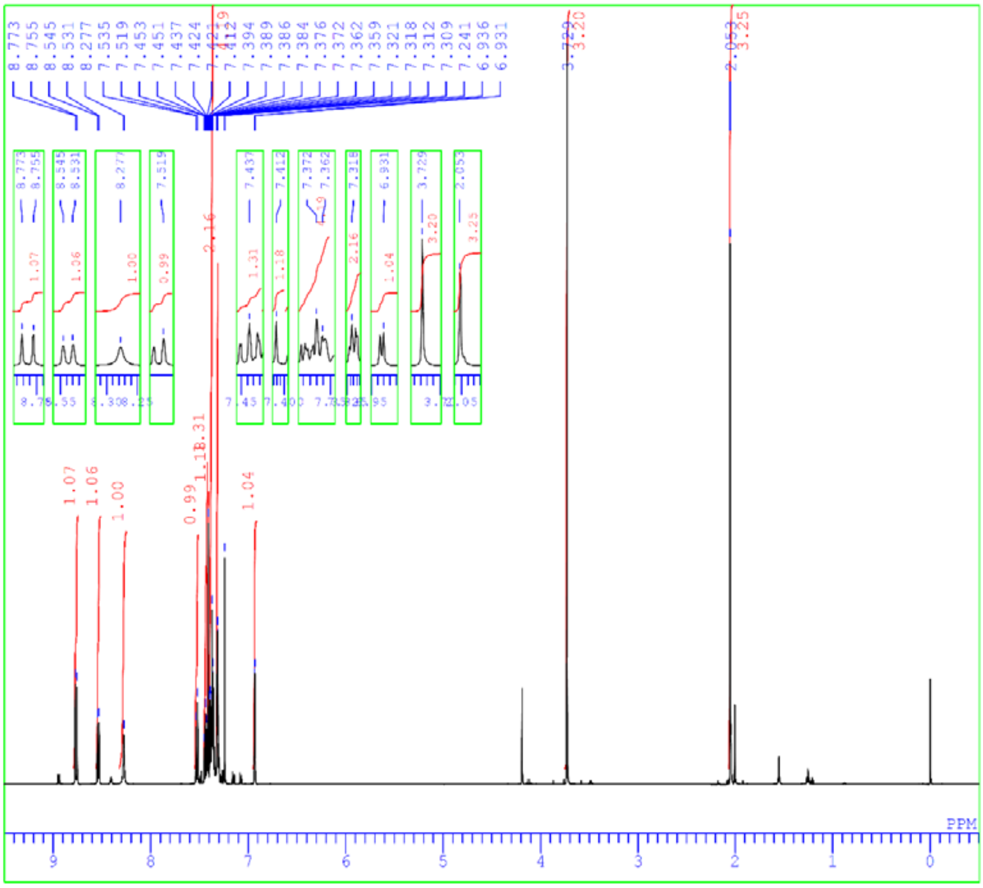

DFILE o-methyl h-1.als
COMNT single_pulse
DATIM 2018-03-06 14:45:12
OBNUC 1H
EXMOD single_pulse.ex2
OBFRQ 500.16 MHz
OBSET 2.41 KHz
OBFIN 6.01 Hz
POINT 13107
FREQU 7507.39 Hz
SCANS 8
ACQTM 1.7459 sec
PD 5.0000 sec
PW1 7.15 usec
IRNUC 1H
CTEMP 19.6 c
SLVNT CDCL3
EXREF 0.00 ppm
BF 0.12 Hz
RGAIN 44

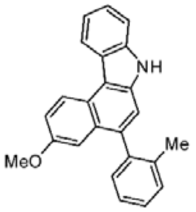

3ba

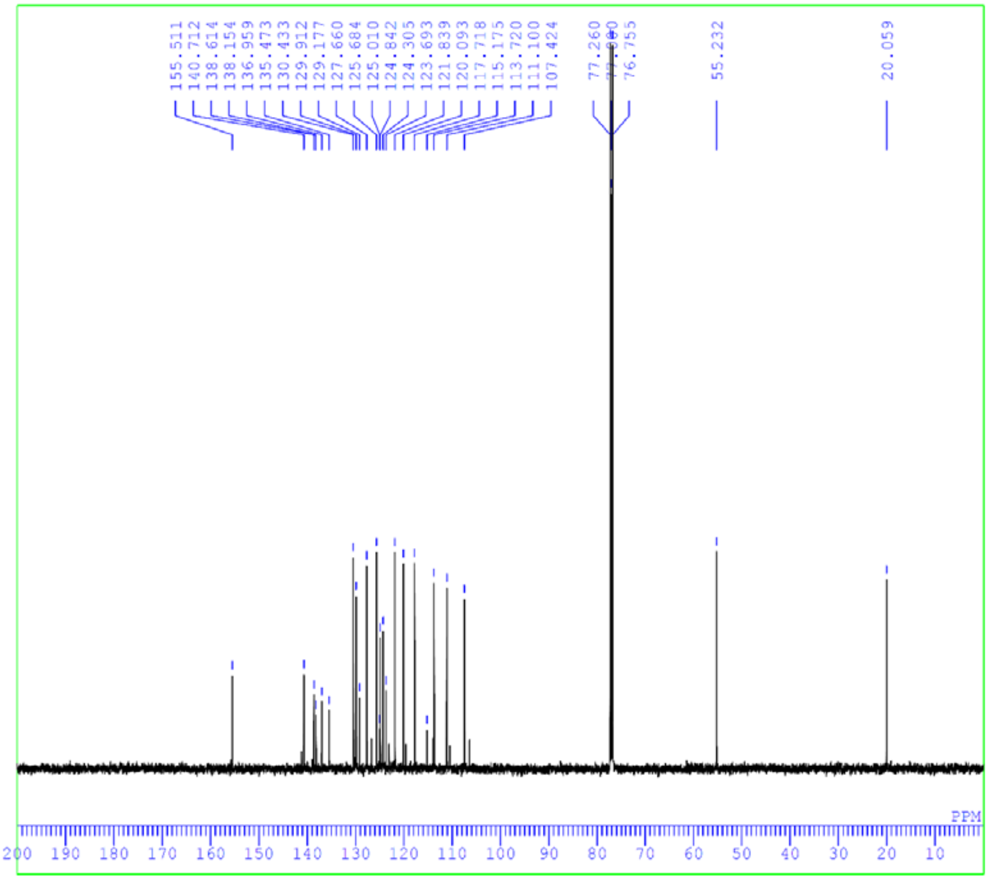

DFILE o-methyl c-1.als
COMNT
DATIM 2018-03-06 15:22:28
OBNUC 13C
EXMOD single_pulse_dec
OBFRQ 125.77 MHz
OBSET 7.87 KHz
OBFIN 4.21 Hz
POINT 13107
FREQU 25252.14 Hz
SCANS 1425
ACQTM 0.5190 sec
PD 1.0000 sec
PW1 3.67 usec
IRNUC 1H
CTEMP 20.2 c
SLVNT CDCL3
EXREF 77.00 ppm
BF 1.20 Hz
RGAIN 56

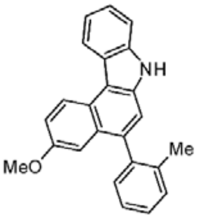

3ba

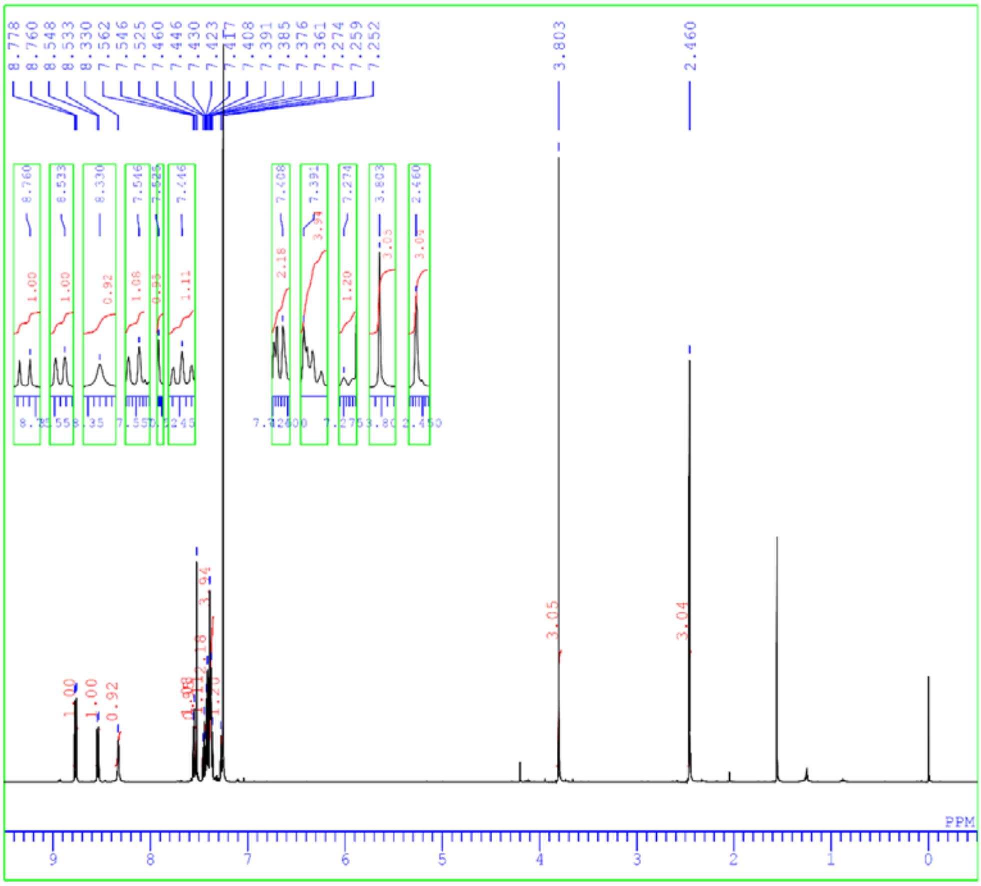

DFILE m-methyl h-1.als
COMNT single_pulse
DATIM 2018-03-05 14:40:30
OBNUC 1H
EXMOD single_pulse.ex2
OBFRQ 500.16 MHz
OBSET 2.41 KHz
OBFIN 6.01 Hz
POINT 13107
FREQU 7507.39 Hz
SCANS 8
ACQTM 1.7459 sec
PD 5.0000 sec
PW1 7.15 usec
IRNUC 1H
CTEMP 19.8 c
SLVNT CDCL3
EXREF 0.00 ppm
BF 0.12 Hz
RGAIN 48

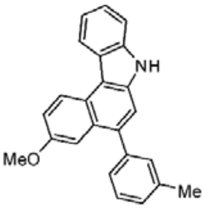

3cA

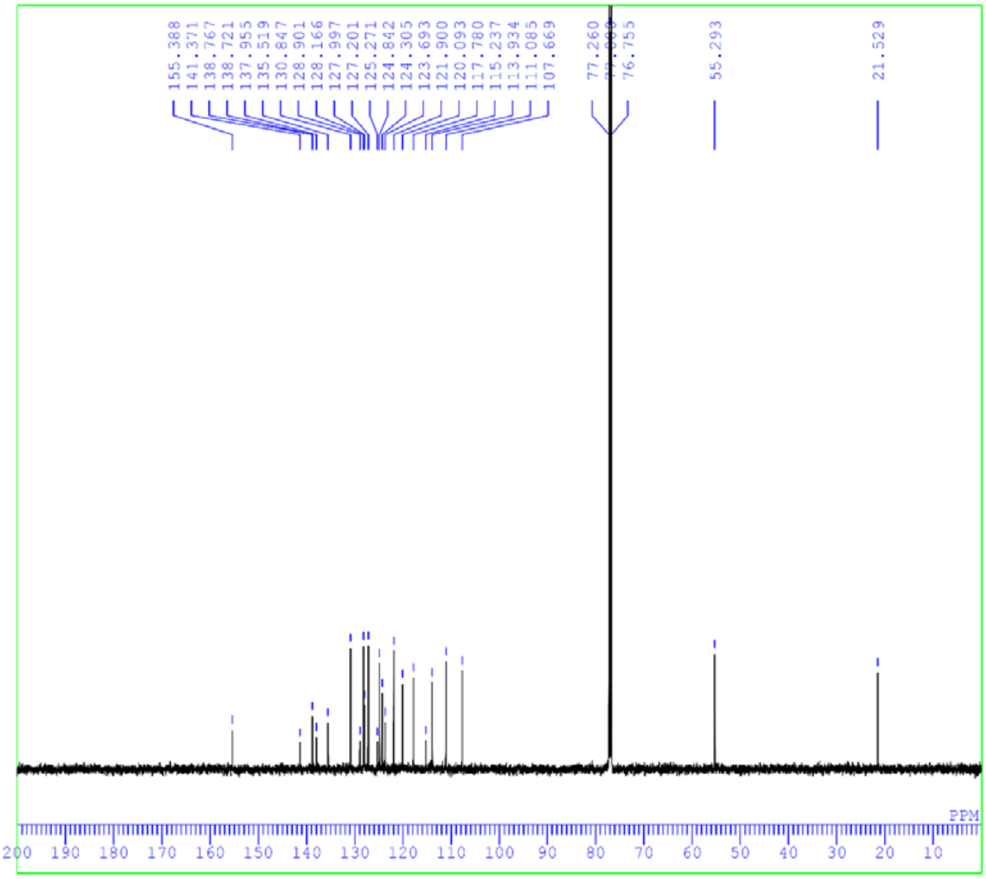

DFILE m-methyl c-1.als
COMNT
DATIM 2018-03-05 15:37:39
OBNUC 13C
EXMOD single_pulse_dec
OBFRQ 125.77 MHz
OBSET 7.87 KHz
OBFIN 4.21 Hz
POINT 13107
FREQU 25252.14 Hz
SCANS 2216
ACQTM 0.5190 sec
PD 1.0000 sec
PW1 3.67 usec
IRNUC 1H
CTEMP 20.5 c
SLVNT CDCL3
EXREF 77.00 ppm
BF 1.20 Hz
RGAIN 56

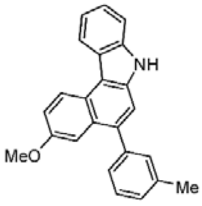

3cA

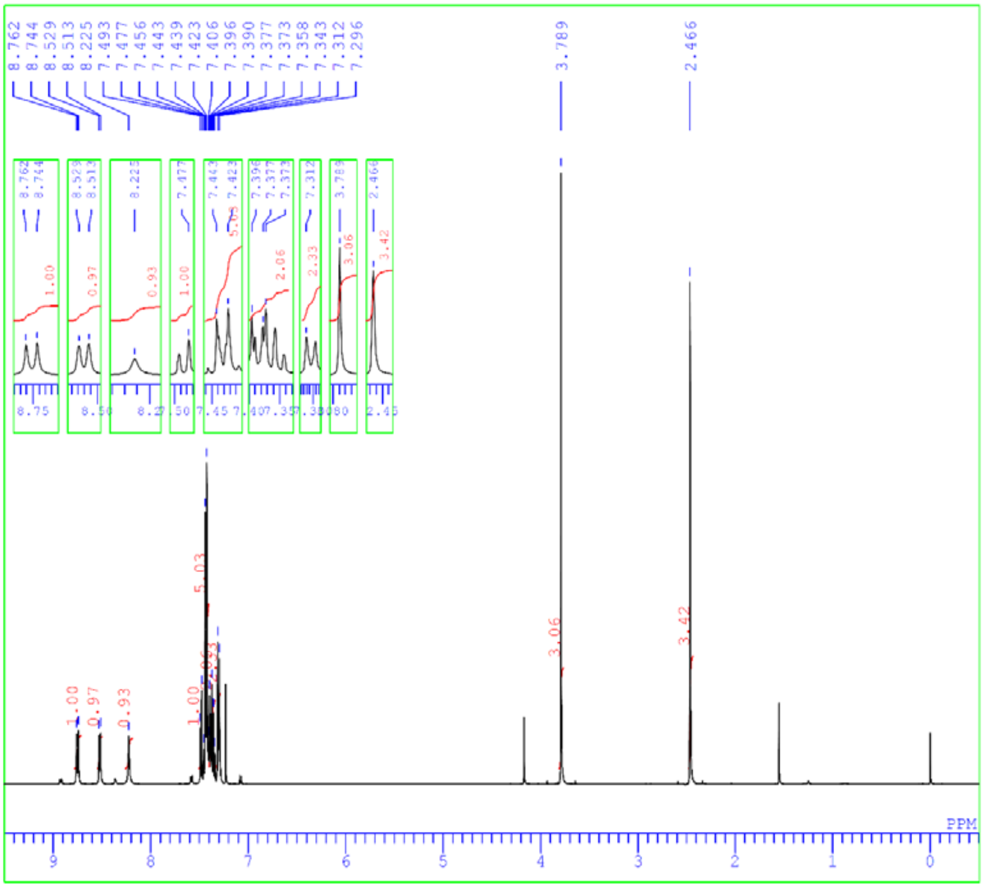

DFILE p-methyl h-1.als
COMNT single_pulse
DATIM 2018-03-05 16:55:01
OBNUC 1H
EXMOD single_pulse.ex2
OBFRQ 500.16 MHz
OBSET 2.41 KHz
OBFIN 6.01 Hz
POINT 13107
FREQU 7507.39 Hz
SCANS 8
ACQTM 1.7459 sec
PD 5.0000 sec
PW1 7.15 usec
IRNUC 1H
CTEMP 19.8 c
SLVNT CDCL3
EXREF 0.00 ppm
BF 0.12 Hz
RGAIN 42

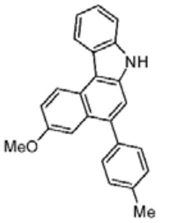

3dA

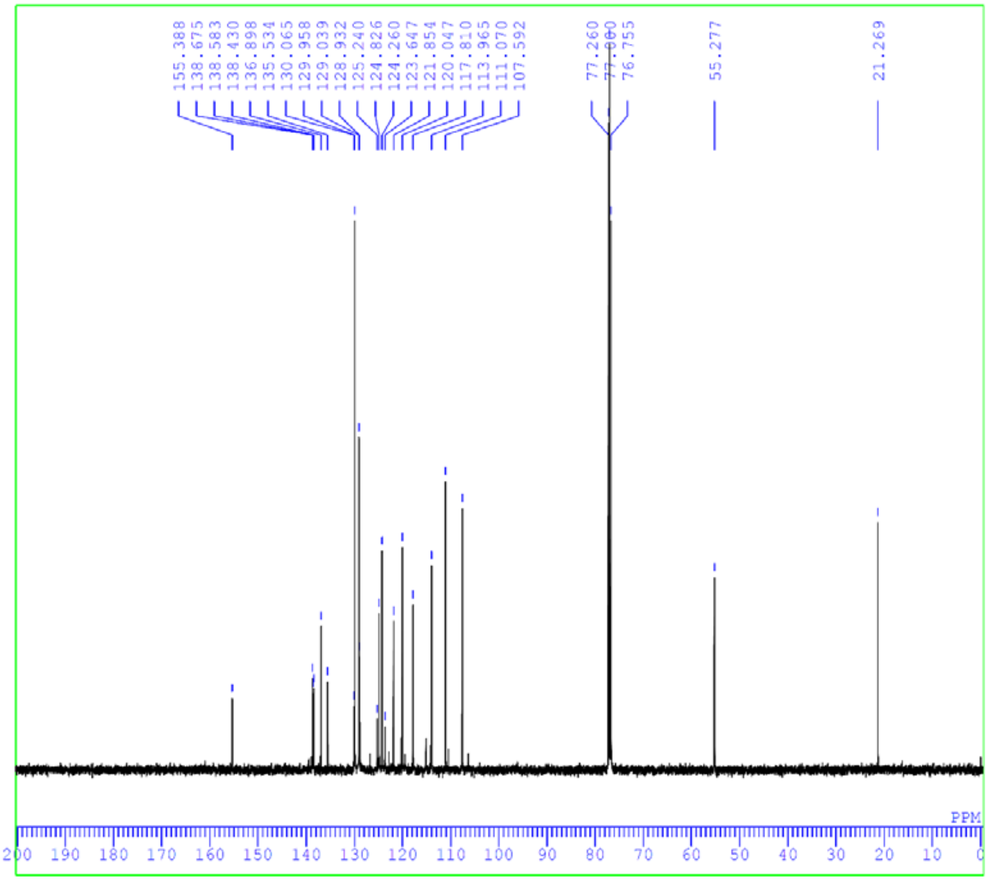

DFILE p-methyl c-1.als
COMNT
DATIM 2018-03-05 17:35:48
OBNUC 13C
EXMOD single_pulse_dec
OBFRQ 125.77 MHz
OBSET 7.87 KHz
OBFIN 4.21 Hz
POINT 13107
FREQU 25252.14 Hz
SCANS 1567
ACQTM 0.5190 sec
PD 1.0000 sec
PW1 3.67 usec
IRNUC 1H
CTEMP 20.3 c
SLVNT CDCL3
EXREF 77.00 ppm
BF 1.20 Hz
RGAIN 58

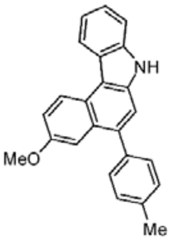

3dA

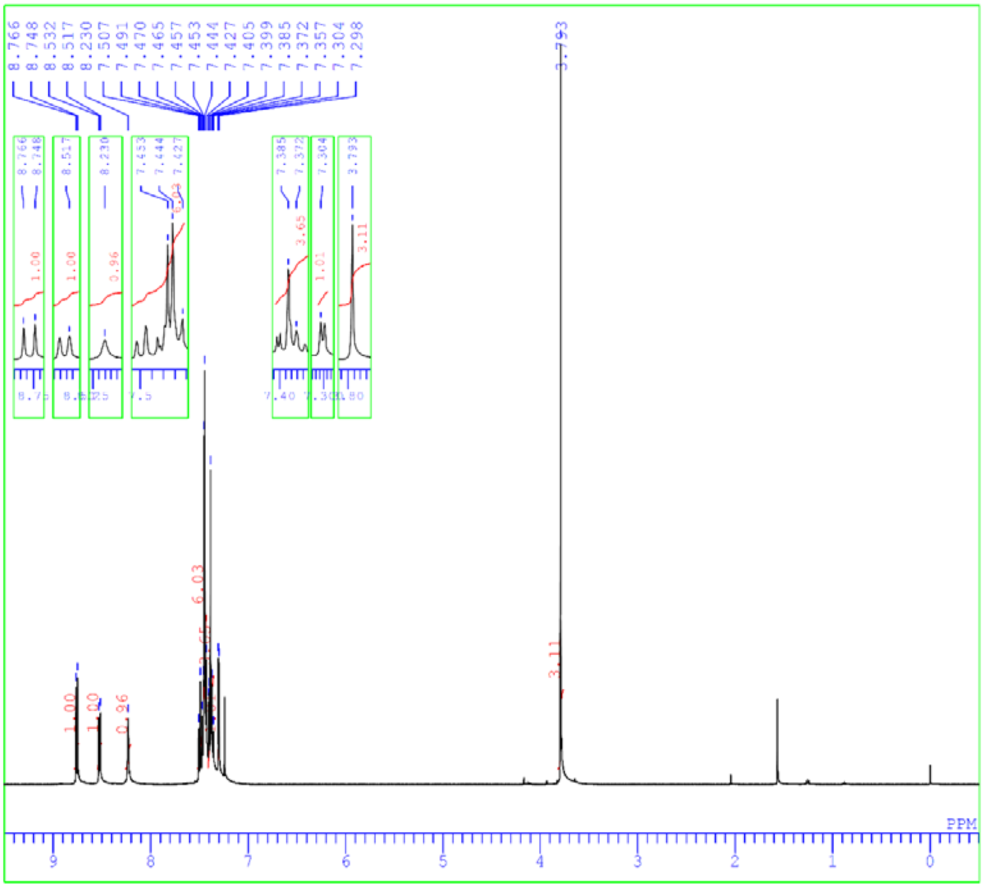


```
DFILE   chloro h-1.als
COMNT   single_pulse
DATIM   2018-02-21 14:53:57
OBNUC   1H
EXMOD   single_pulse.ex2
OBFRQ        500.16 MHz
OBSET          2.41 KHz
OBFIN          6.01 Hz
POINT         13107
FREQU       7507.39 Hz
SCANS             8
ACQTM        1.7459 sec
PD           5.0000 sec
PW1            7.15 usec
IRNUC   1H
CTEMP          18.6 c
SLVNT   CDCL3
EXREF          0.00 ppm
BF             0.12 Hz
RGAIN            44
```

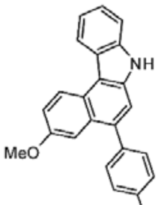

3eA

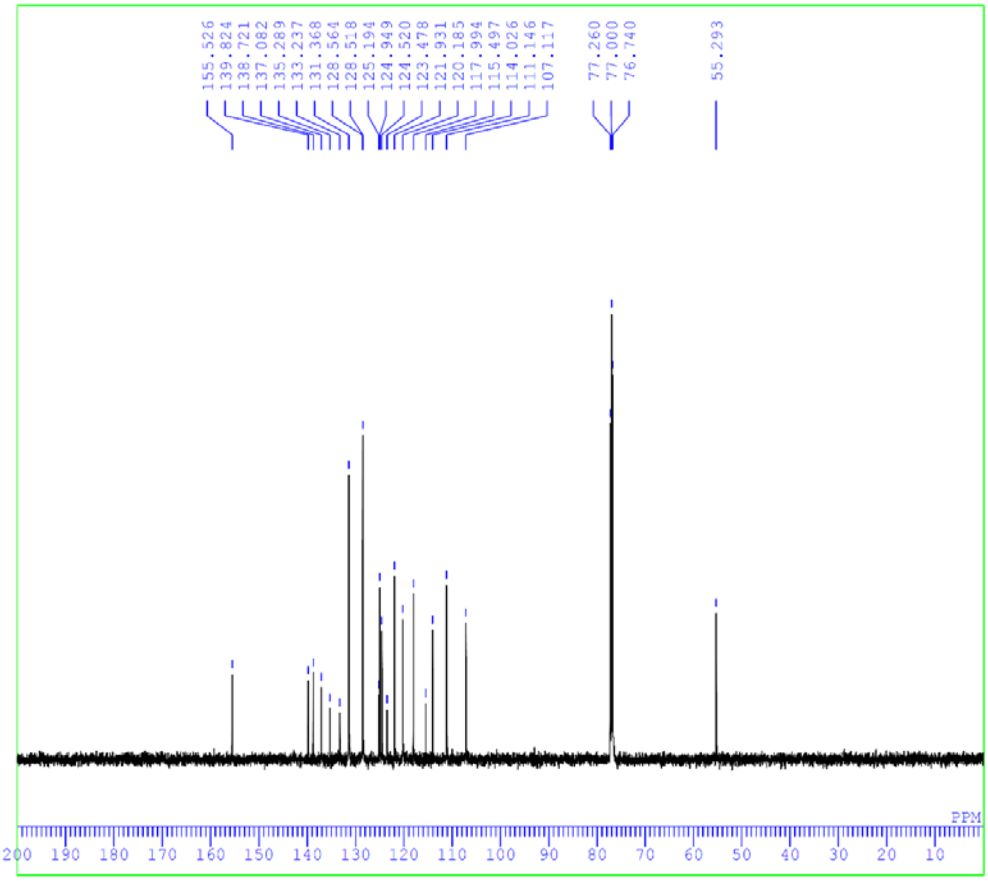


```
DFILE   chloro c-1.als
COMNT
DATIM   2018-02-21 15:10:38
OBNUC   13C
EXMOD   single_pulse_dec
OBFRQ        125.77 MHz
OBSET          7.87 KHz
OBFIN          4.21 Hz
POINT         13107
FREQU      25252.14 Hz
SCANS           616
ACQTM        0.5190 sec
PD           1.0000 sec
PW1            3.67 usec
IRNUC   1H
CTEMP          19.1 c
SLVNT   CDCL3
EXREF         77.00 ppm
BF             1.20 Hz
RGAIN            56
```

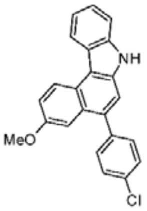

3eA

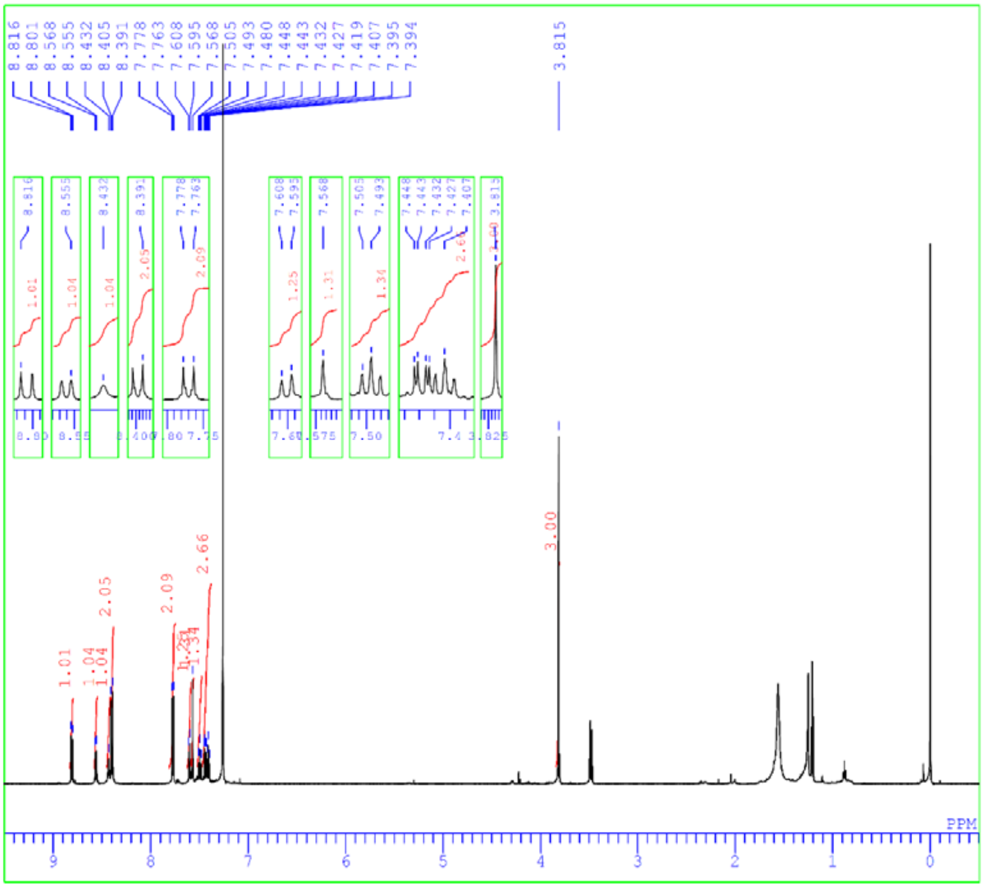

DFILE	nitro h-1.als
COMNT	
DATIM	2018-03-08 14:09:33
OBNUC	1H
EXMOD	zg30
OBFRQ	600.13 MHz
OBSET	3.00 KHz
OBFIN	0.65 Hz
POINT	16384
FREQU	8992.81 Hz
SCANS	128
ACQTM	0.0000 sec
PD	0.0000 sec
PW1	10.00 usec
IRNUC	
CTEMP	24.9 c
SLVNT	CDCl3
EXREF	0.00 ppm
BF	0.12 Hz
RGAIN	574

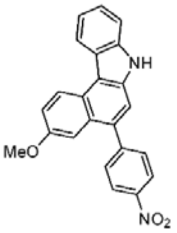

3fA

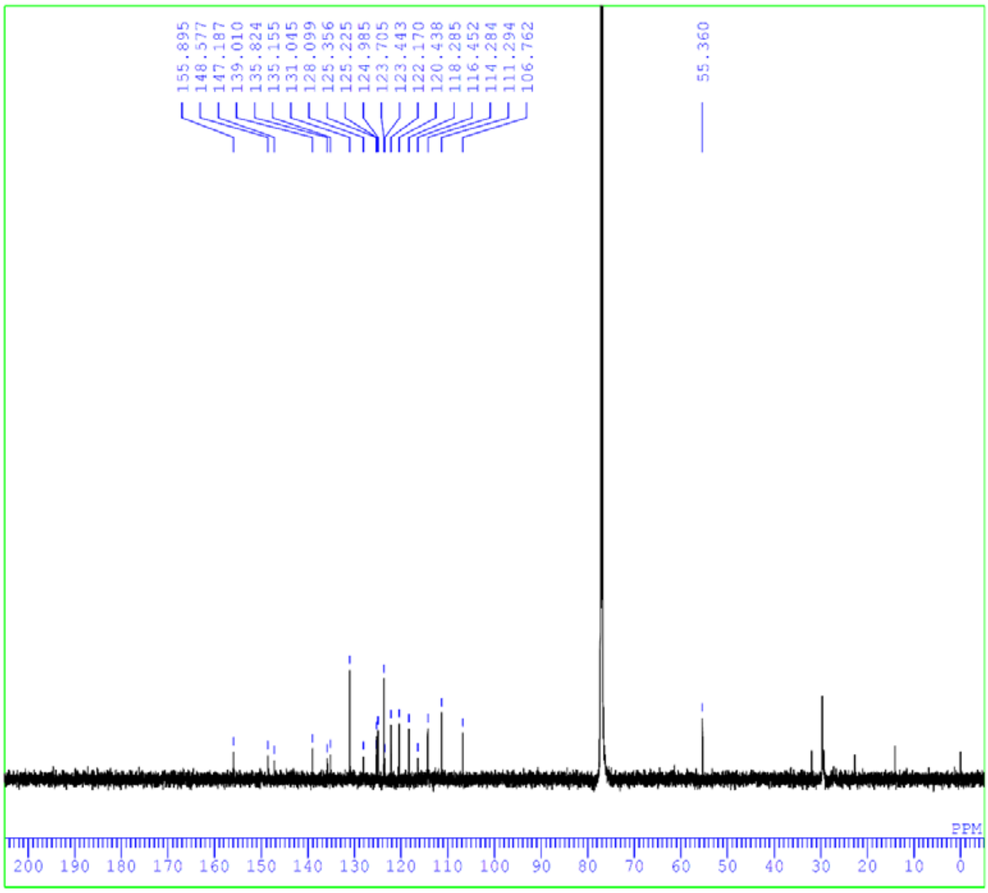

DFILE	nitro c-1.als
COMNT	
DATIM	2018-03-23 06:58:45
OBNUC	13C
EXMOD	zgpg30
OBFRQ	150.91 MHz
OBSET	7.89 KHz
OBFIN	8.81 Hz
POINT	32768
FREQU	35971.22 Hz
SCANS	80000
ACQTM	0.0000 sec
PD	0.0000 sec
PW1	10.00 usec
IRNUC	
CTEMP	24.9 c
SLVNT	CDCl3
EXREF	219.18 ppm
BF	1.20 Hz
RGAIN	4597

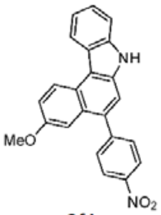

3fA

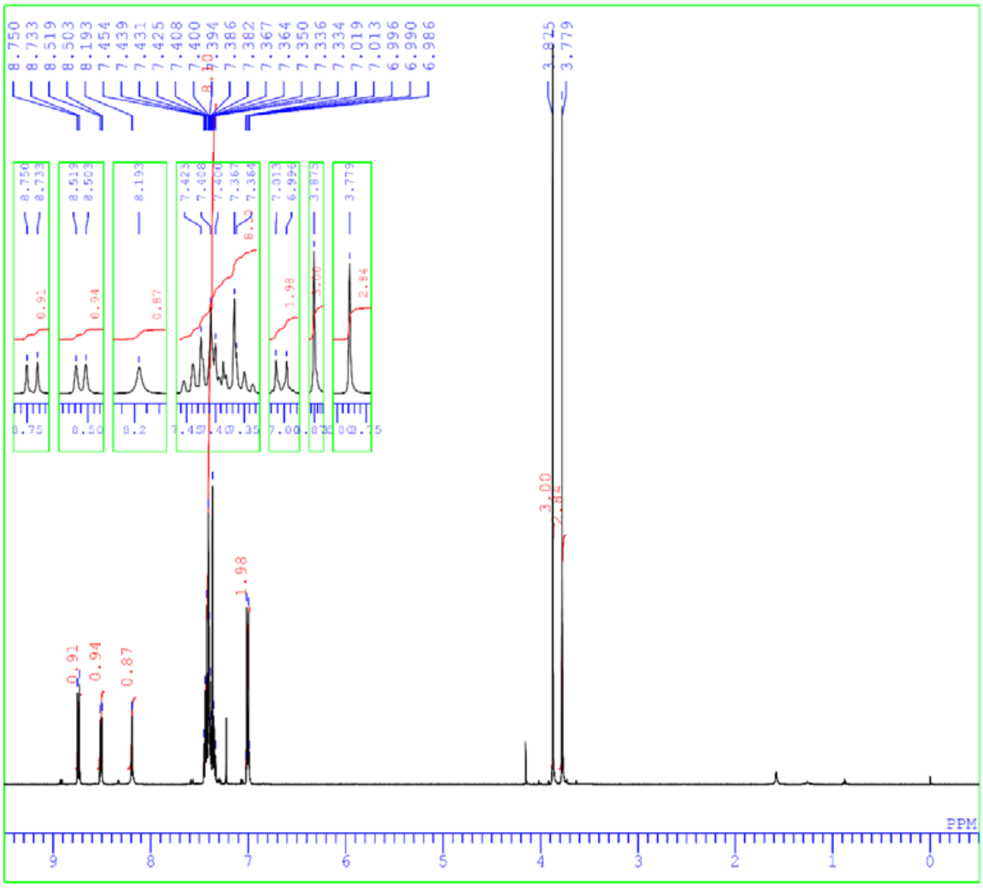

DFILE methoxy h-1.als
COMNT single_pulse
DATIM 2018-03-01 16:39:44
OBNUC 1H
EXMOD single_pulse.ex2
OBFRQ 500.16 MHz
OBSET 2.41 KHz
OBFIN 6.01 Hz
POINT 13107
FREQU 7507.39 Hz
SCANS 8
ACQTM 1.7459 sec
PD 5.0000 sec
PW1 7.15 usec
IRNUC 1H
CTEMP 19.6 c
SLVNT CDCL3
EXREF 0.00 ppm
BF 0.12 Hz
RGAIN 40

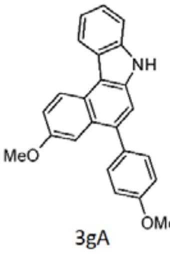

3gA

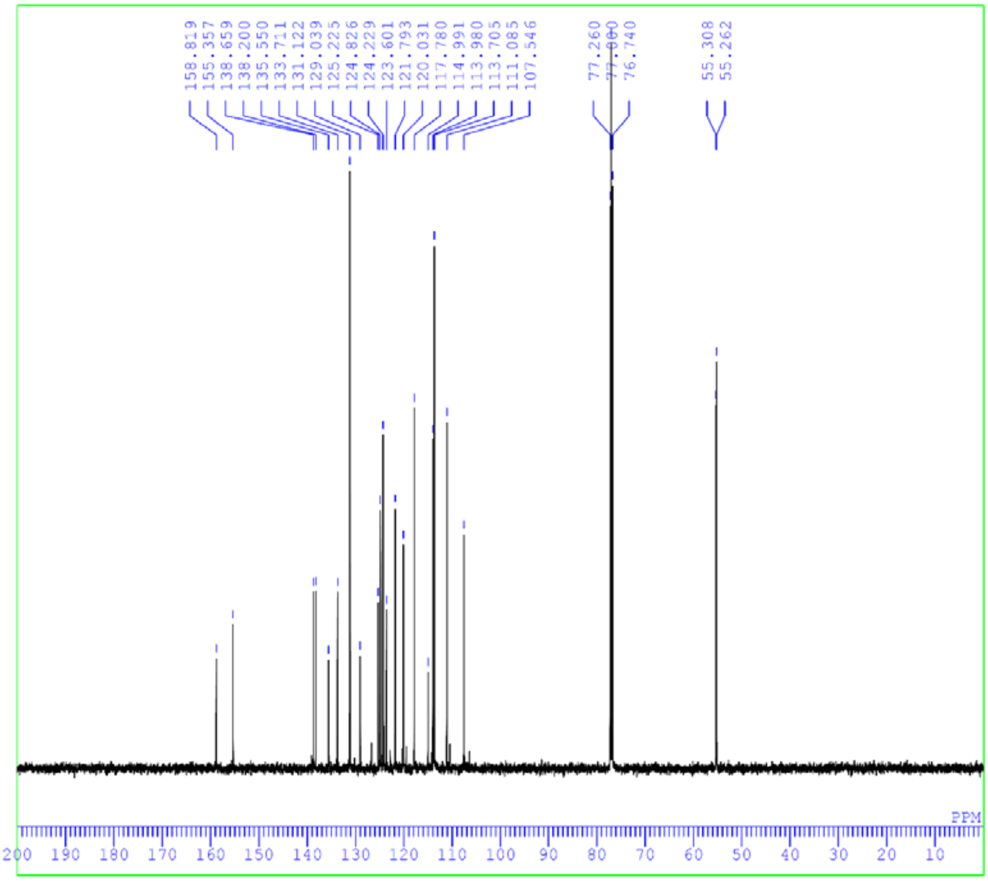

DFILE methoxy c-1.als
COMNT
DATIM 2018-03-01 17:13:56
OBNUC 13C
EXMOD single_pulse_dec
OBFRQ 125.77 MHz
OBSET 7.87 KHz
OBFIN 4.21 Hz
POINT 13107
FREQU 25252.14 Hz
SCANS 1332
ACQTM 0.5190 sec
PD 1.0000 sec
PW1 3.67 usec
IRNUC 1H
CTEMP 20.0 c
SLVNT CDCL3
EXREF 77.00 ppm
BF 1.20 Hz
RGAIN 60

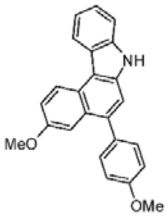

3gA

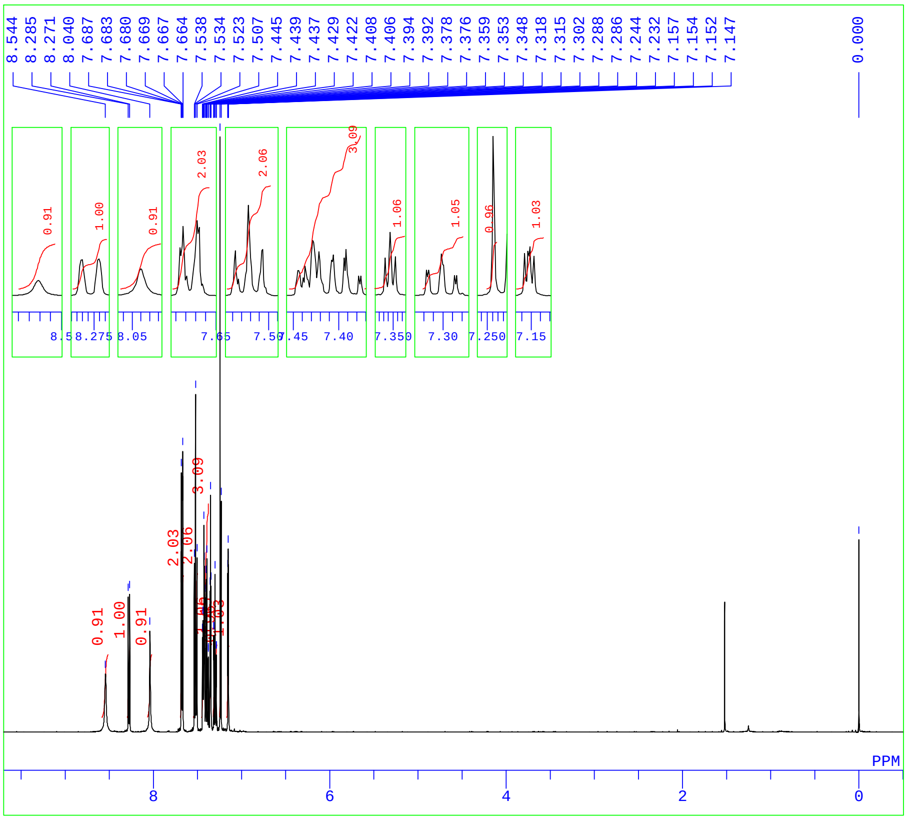

DFILE	6aA H-1.als
COMNT	single_pulse
DATIM	2017-02-23 21:40:53
OBNUC	1H
EXMOD	single_pulse.ex2
OBFRQ	500.16 MHz
OBSET	2.41 KHz
OBFIN	6.01 Hz
POINT	13107
FREQU	7507.39 Hz
SCANS	8
ACQTM	1.7459 sec
PD	5.0000 sec
PW1	7.15 usec
IRNUC	1H
CTEMP	19.7 c
SLVNT	CDCL3
EXREF	0.00 ppm
BF	0.12 Hz
RGAIN	44

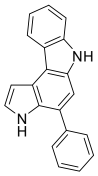

6aA

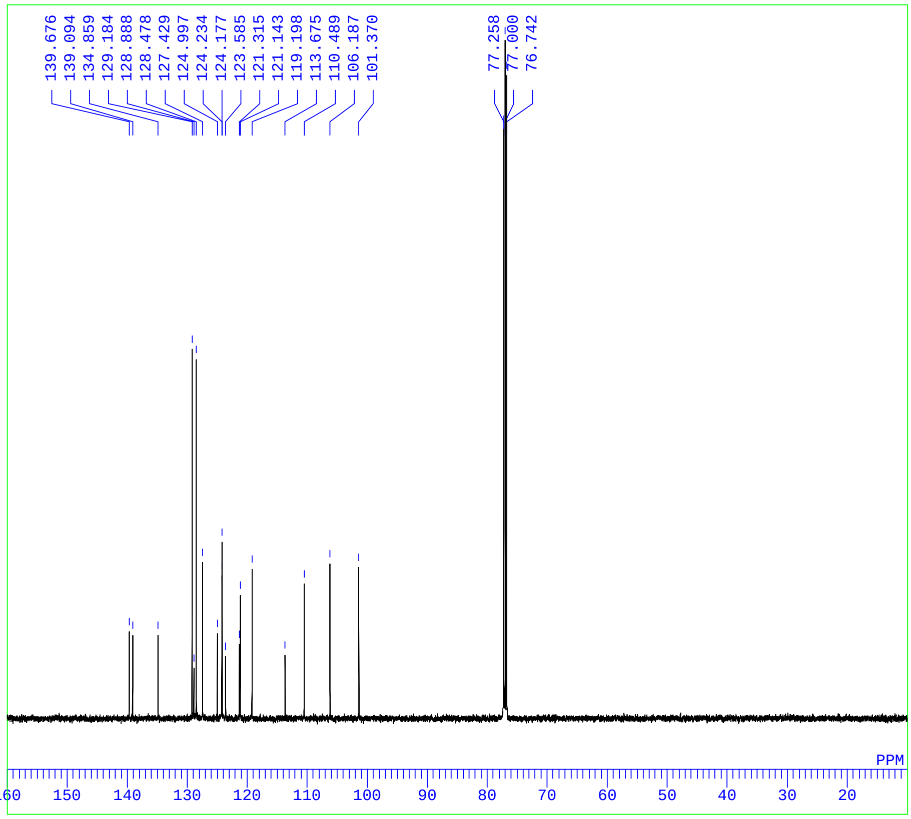

DFILE	6aA C-1.als
COMNT	
DATIM	2017-02-24 01:01:33
OBNUC	13C
EXMOD	single_pulse_dec
OBFRQ	125.77 MHz
OBSET	7.87 KHz
OBFIN	4.21 Hz
POINT	26214
FREQU	31446.06 Hz
SCANS	1024
ACQTM	0.8336 sec
PD	2.0000 sec
PW1	3.67 usec
IRNUC	1H
CTEMP	20.4 c
SLVNT	CDCL3
EXREF	77.00 ppm
BF	1.20 Hz
RGAIN	60

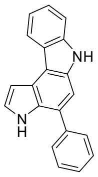

6aA

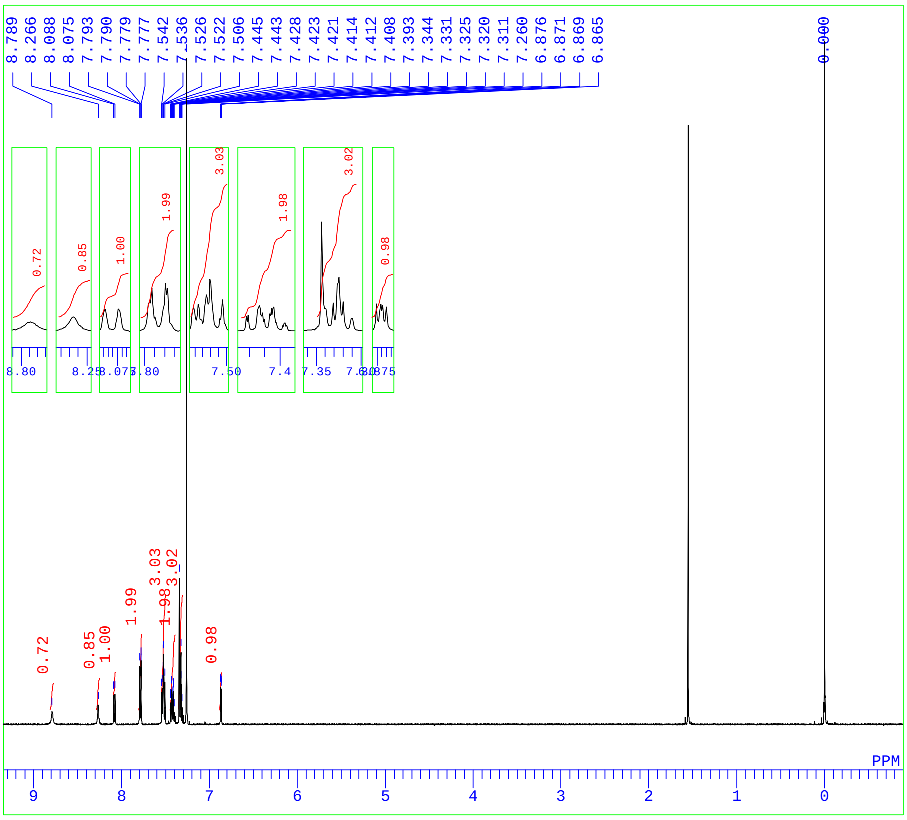

```
DFILE  7aA H-1.als
COMNT  single_pulse
DATIM  2017-02-24 16:25:33
OBNUC  1H
EXMOD  single_pulse.ex2
OBFRQ       500.16 MHz
OBSET         2.41 KHz
OBFIN         6.01 Hz
POINT        13107
FREQU      7507.39 Hz
SCANS            8
ACQTM       1.7459 sec
PD          5.0000 sec
PW1           7.15 usec
IRNUC  1H
CTEMP         19.5 c
SLVNT  CDCL3
EXREF         0.00 ppm
BF            0.12 Hz
RGAIN           54
```

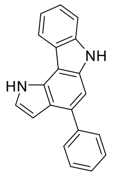

7aA

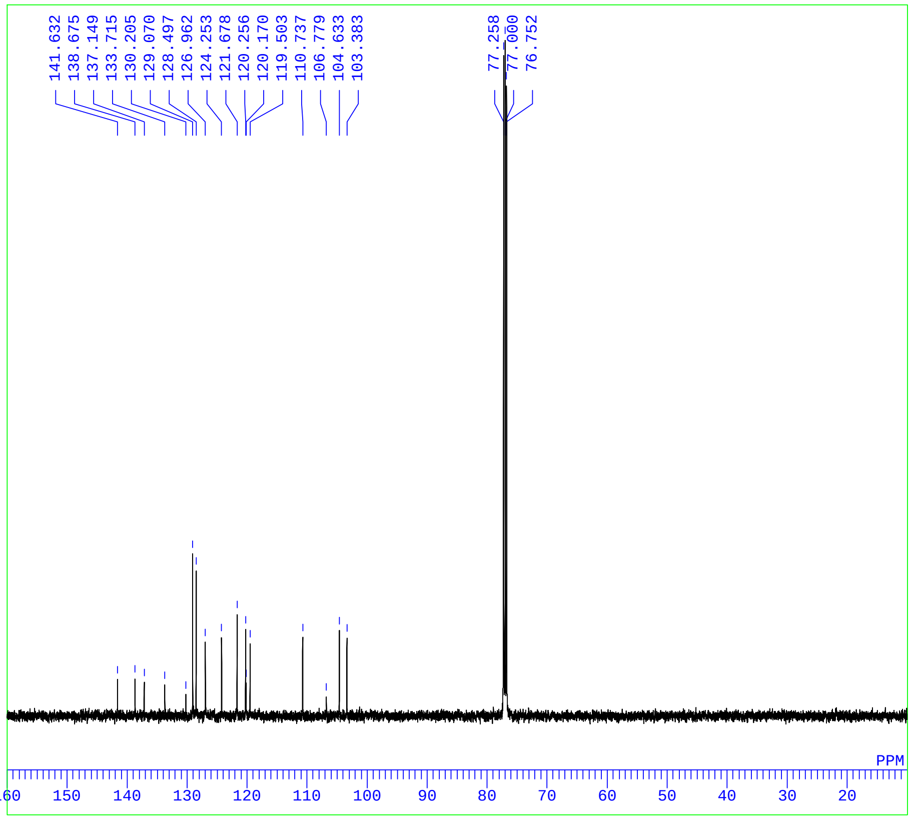

```
DFILE  7aA C-1.als
COMNT
DATIM  2017-02-24 00:05:10
OBNUC  13C
EXMOD  single_pulse_dec
OBFRQ       125.77 MHz
OBSET         7.87 KHz
OBFIN         4.21 Hz
POINT        26214
FREQU     31446.06 Hz
SCANS         1024
ACQTM       0.8336 sec
PD          2.0000 sec
PW1           3.67 usec
IRNUC  1H
CTEMP         20.4 c
SLVNT  CDCL3
EXREF        77.00 ppm
BF            1.20 Hz
RGAIN           54
```

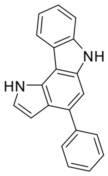

7aA

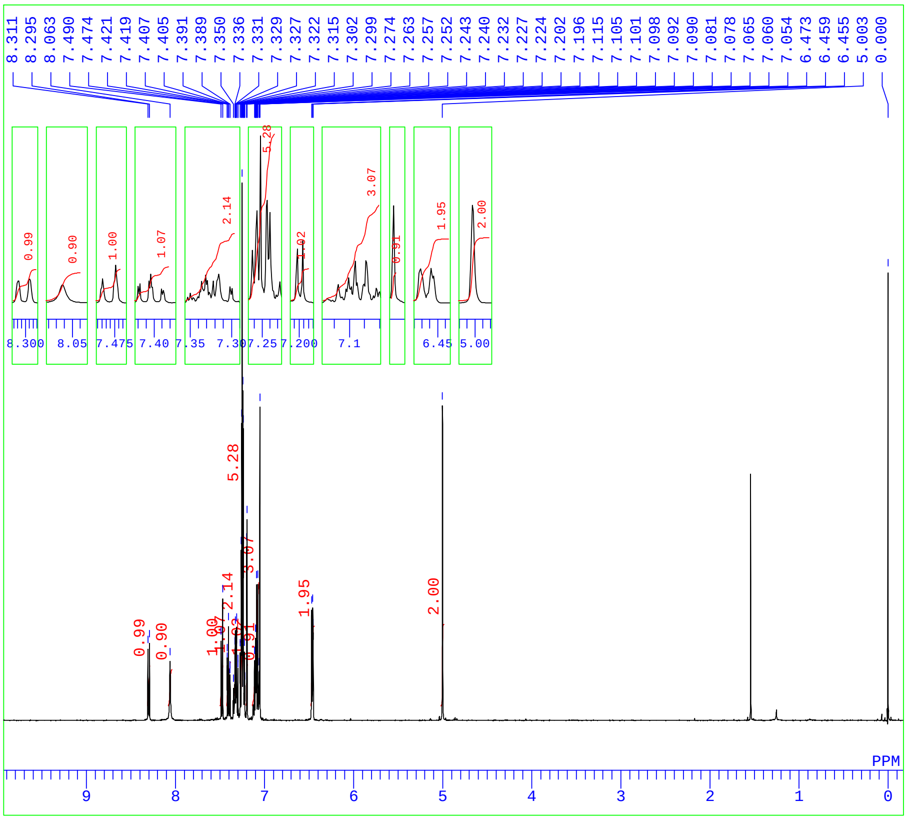

DFILE 6aB H-1.als
COMNT single_pulse
DATIM 2017-02-22 17:44:13
OBNUC 1H
EXMOD single_pulse.ex2
OBFRQ 500.16 MHz
OBSET 2.41 KHz
OBFIN 6.01 Hz
POINT 13107
FREQU 7507.39 Hz
SCANS 8
ACQTM 1.7459 sec
PD 5.0000 sec
PW1 7.15 usec
IRNUC 1H
CTEMP 19.8 c
SLVNT CDCL3
EXREF 0.00 ppm
BF 0.12 Hz
RGAIN 48

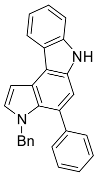

6aB

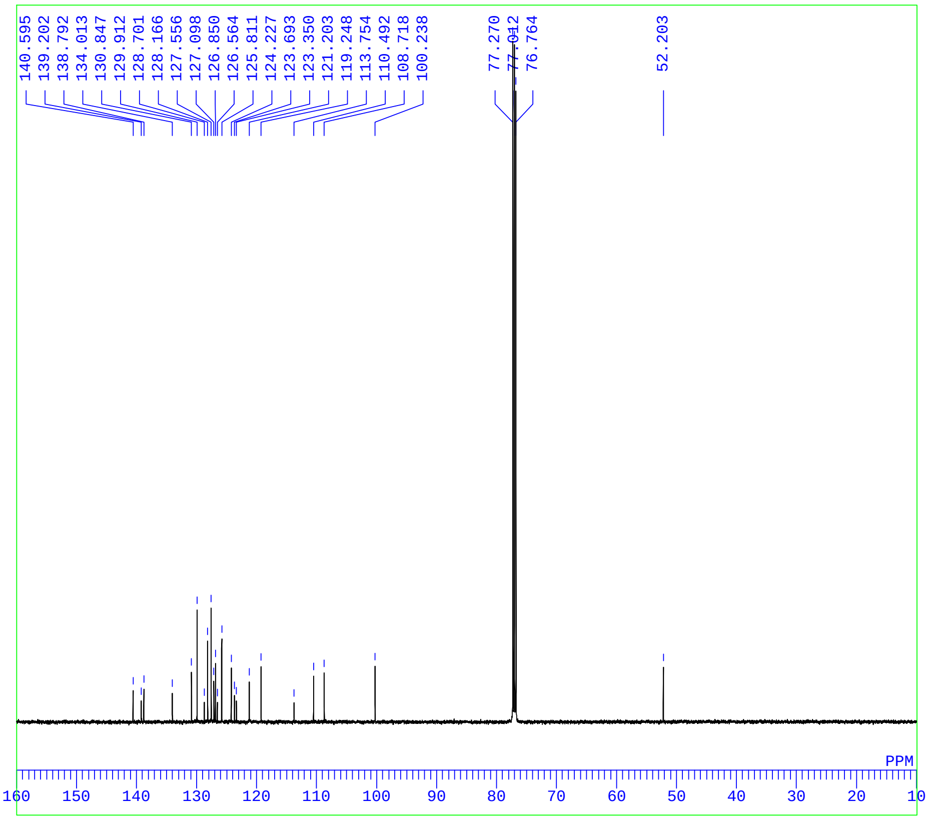

DFILE 6aB C-1.als
COMNT
DATIM 2017-02-24 04:12:56
OBNUC 13C
EXMOD single_pulse_dec
OBFRQ 125.77 MHz
OBSET 7.87 KHz
OBFIN 4.21 Hz
POINT 26214
FREQU 31446.06 Hz
SCANS 3900
ACQTM 0.8336 sec
PD 2.0000 sec
PW1 3.67 usec
IRNUC 1H
CTEMP 20.3 c
SLVNT CDCL3
EXREF 0.00 ppm
BF 1.20 Hz
RGAIN 60

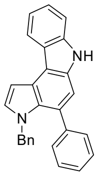

6aB

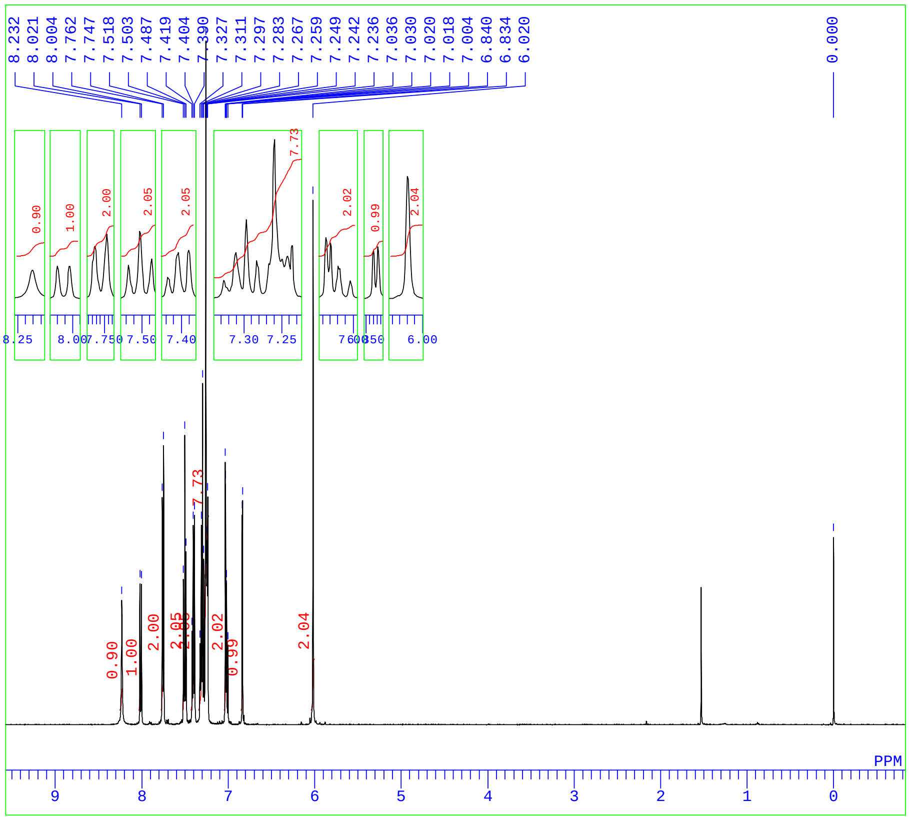

DFILE 7aB H-1.als
COMNT single_pulse
DATIM 2017-02-22 17:49:26
OBNUC 1H
EXMOD single_pulse.ex2
OBFRQ 500.16 MHz
OBSET 2.41 KHz
OBFIN 6.01 Hz
POINT 13107
FREQU 7507.39 Hz
SCANS 8
ACQTM 1.7459 sec
PD 5.0000 sec
PW1 7.15 usec
IRNUC 1H
CTEMP 19.8 c
SLVNT CDCL3
EXREF 0.00 ppm
BF 0.12 Hz
RGAIN 44

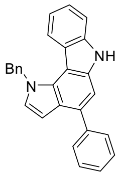

7aB

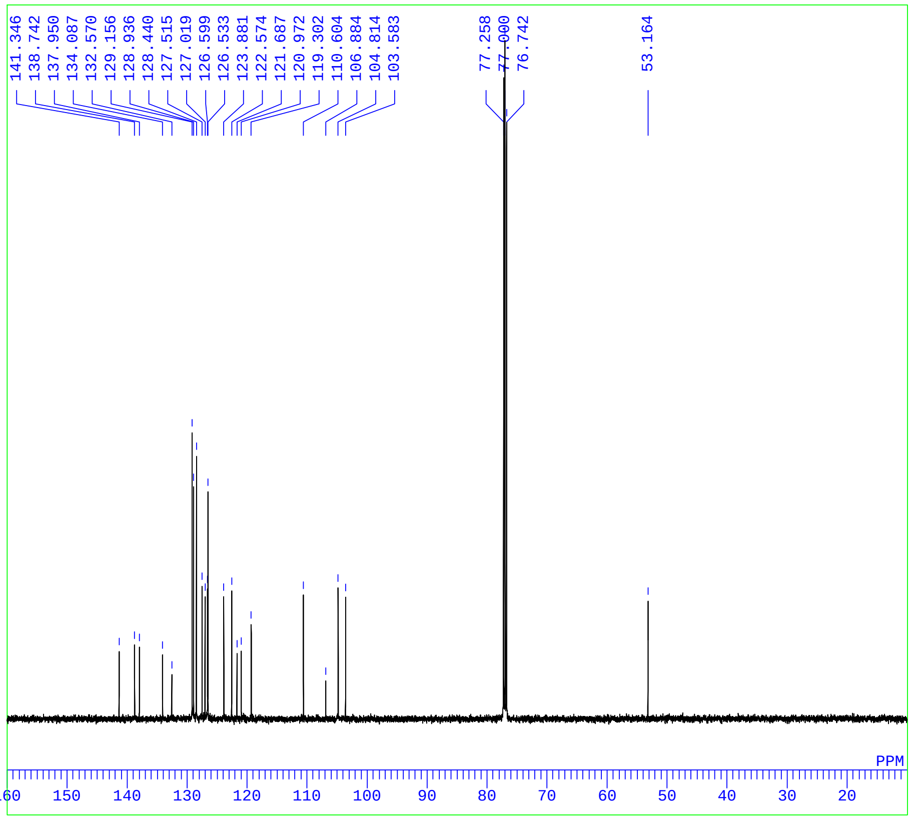

DFILE 7aB C-1.als
COMNT
DATIM 2017-02-24 05:06:42
OBNUC 13C
EXMOD single_pulse_dec
OBFRQ 125.77 MHz
OBSET 7.87 KHz
OBFIN 4.21 Hz
POINT 26214
FREQU 31446.06 Hz
SCANS 1024
ACQTM 0.8336 sec
PD 2.0000 sec
PW1 3.67 usec
IRNUC 1H
CTEMP 20.2 c
SLVNT CDCL3
EXREF 77.00 ppm
BF 1.20 Hz
RGAIN 60

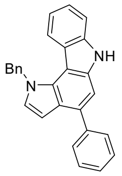

7aB

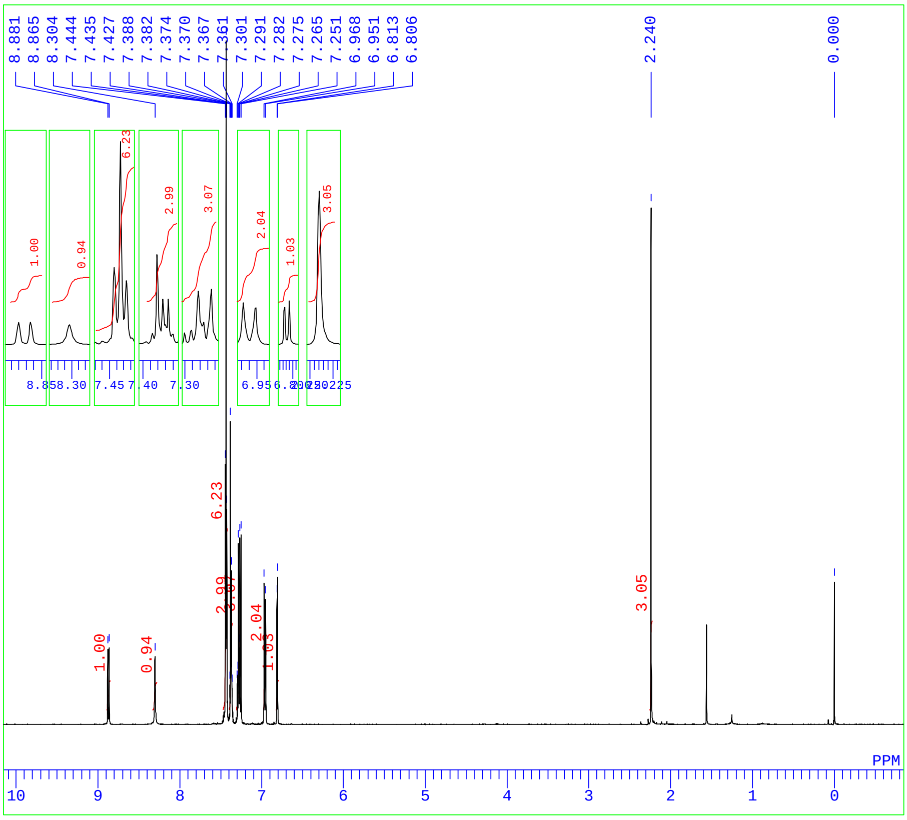

DFILE 6aC H-1.als
COMNT single_pulse
DATIM 2017-02-22 17:53:52
OBNUC 1H
EXMOD single_pulse.ex2
OBFRQ 500.16 MHz
OBSET 2.41 KHz
OBFIN 6.01 Hz
POINT 13107
FREQU 7507.39 Hz
SCANS 8
ACQTM 1.7459 sec
PD 5.0000 sec
PW1 7.15 usec
IRNUC 1H
CTEMP 19.8 c
SLVNT CDCL3
EXREF 0.00 ppm
BF 0.12 Hz
RGAIN 44

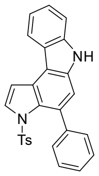

6aC

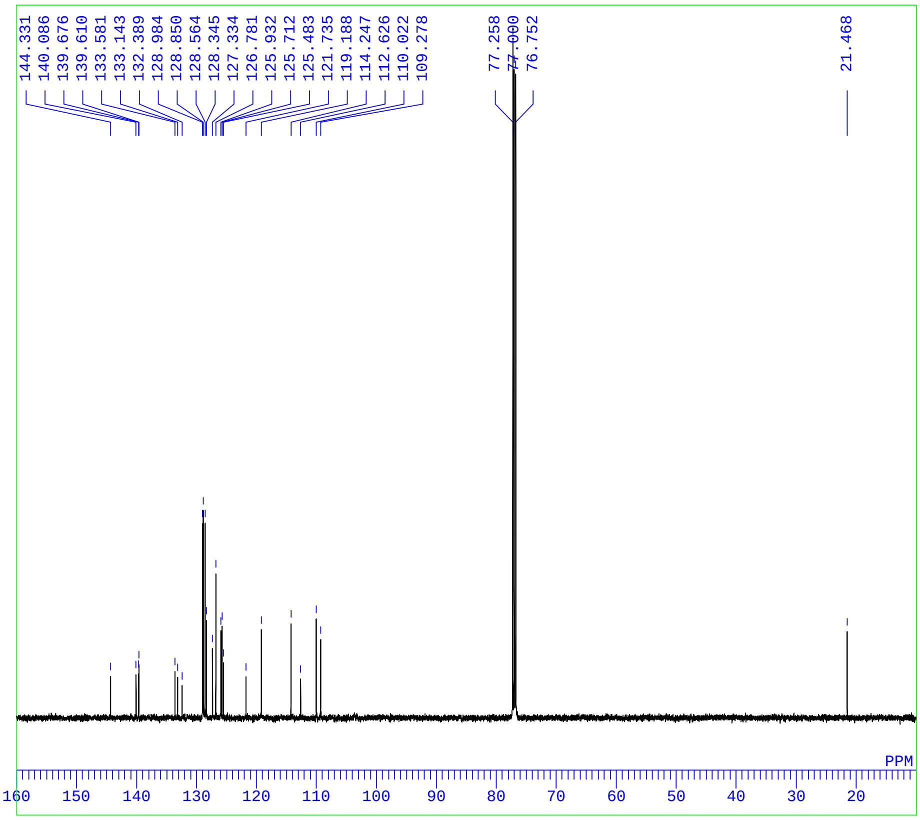

DFILE 6aC C-1.als
COMNT
DATIM 2017-02-24 07:51:52
OBNUC 13C
EXMOD single_pulse_dec
OBFRQ 125.77 MHz
OBSET 7.87 KHz
OBFIN 4.21 Hz
POINT 26214
FREQU 31446.06 Hz
SCANS 1024
ACQTM 0.8336 sec
PD 2.0000 sec
PW1 3.67 usec
IRNUC 1H
CTEMP 20.0 c
SLVNT CDCL3
EXREF 77.00 ppm
BF 1.20 Hz
RGAIN 60

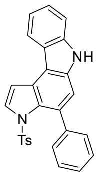

6aC

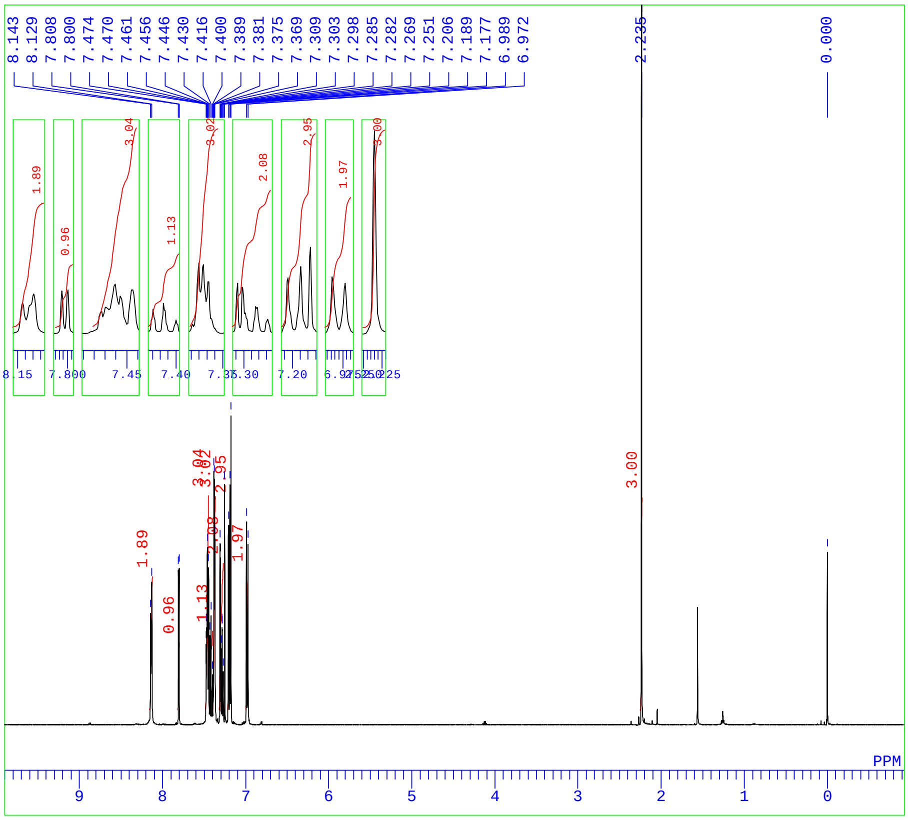

DFILE 7aC H-1.als
COMNT single_pulse
DATIM 2017-02-22 17:58:39
OBNUC 1H
EXMOD single_pulse.ex2
OBFRQ 500.16 MHz
OBSET 2.41 KHz
OBFIN 6.01 Hz
POINT 13107
FREQU 7507.39 Hz
SCANS 8
ACQTM 1.7459 sec
PD 5.0000 sec
PW1 7.15 usec
IRNUC 1H
CTEMP 19.8 c
SLVNT CDCL3
EXREF 0.00 ppm
BF 0.12 Hz
RGAIN 44

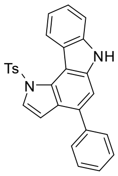

7aC

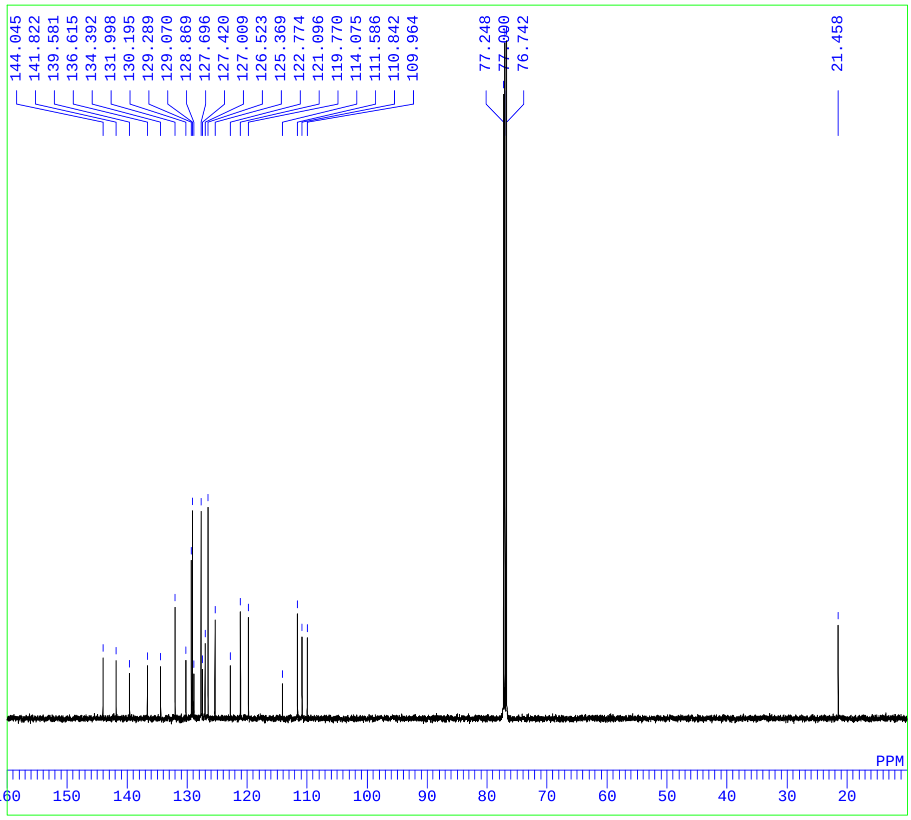

DFILE 7aC C-1.als
COMNT
DATIM 2017-02-24 08:46:53
OBNUC 13C
EXMOD single_pulse_dec
OBFRQ 125.77 MHz
OBSET 7.87 KHz
OBFIN 4.21 Hz
POINT 26214
FREQU 31446.06 Hz
SCANS 1024
ACQTM 0.8336 sec
PD 2.0000 sec
PW1 3.67 usec
IRNUC 1H
CTEMP 20.0 c
SLVNT CDCL3
EXREF 77.00 ppm
BF 1.20 Hz
RGAIN 60

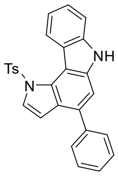

7aC

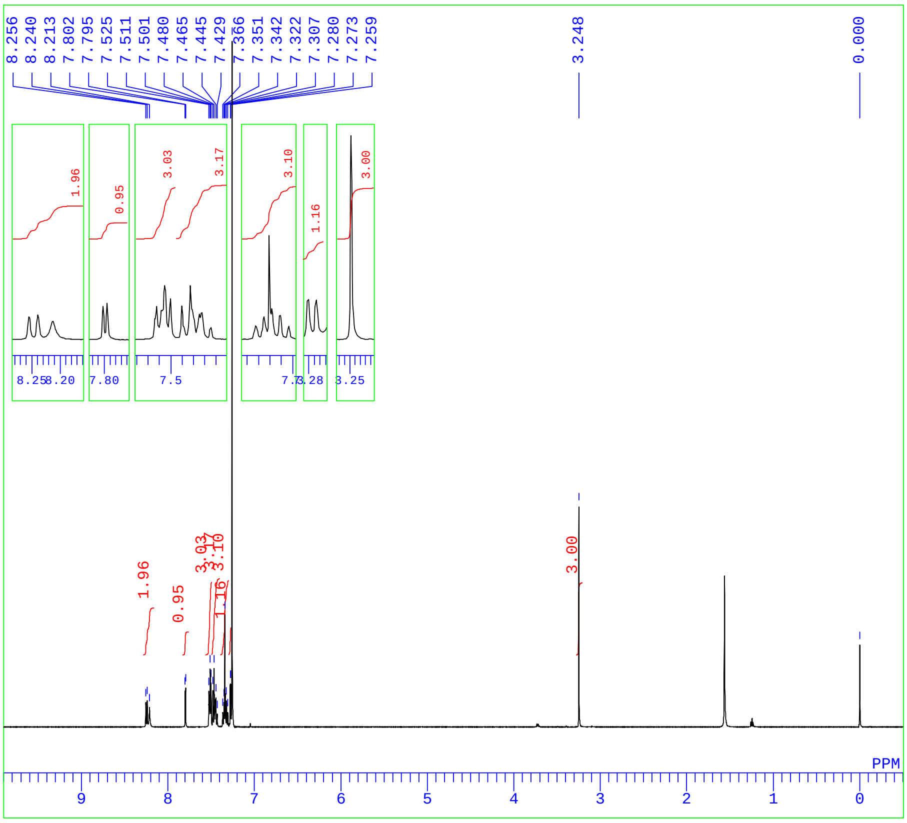

DFILE 6aD.als
COMNT single_pulse
DATIM 2018-04-27 19:42:40
OBNUC 1H
EXMOD single_pulse.ex2
OBFRQ 500.16 MHz
OBSET 2.41 KHz
OBFIN 6.01 Hz
POINT 13107
FREQU 7507.39 Hz
SCANS 8
ACQTM 1.7459 sec
PD 5.0000 sec
PW1 7.15 usec
IRNUC 1H
CTEMP 21.2 c
SLVNT CDCL3
EXREF 0.00 ppm
BF 0.12 Hz
RGAIN 50

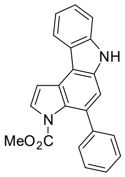

6aD

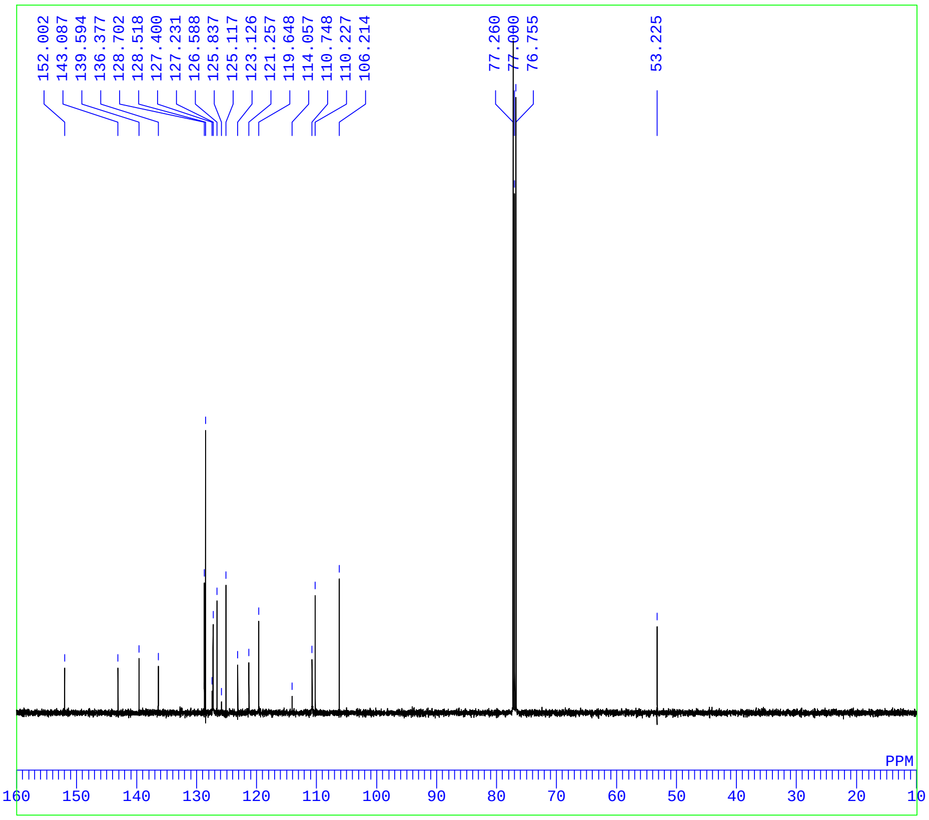

DFILE 6aD Cc-1.als
COMNT
DATIM 2018-04-29 18:34:57
OBNUC 13C
EXMOD single_pulse_dec
OBFRQ 125.77 MHz
OBSET 7.87 KHz
OBFIN 4.21 Hz
POINT 13107
FREQU 25252.14 Hz
SCANS 3507
ACQTM 0.5190 sec
PD 1.0000 sec
PW1 3.67 usec
IRNUC 1H
CTEMP 21.4 c
SLVNT CDCL3
EXREF 77.00 ppm
BF 0.12 Hz
RGAIN 60

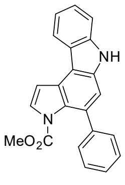

6aD

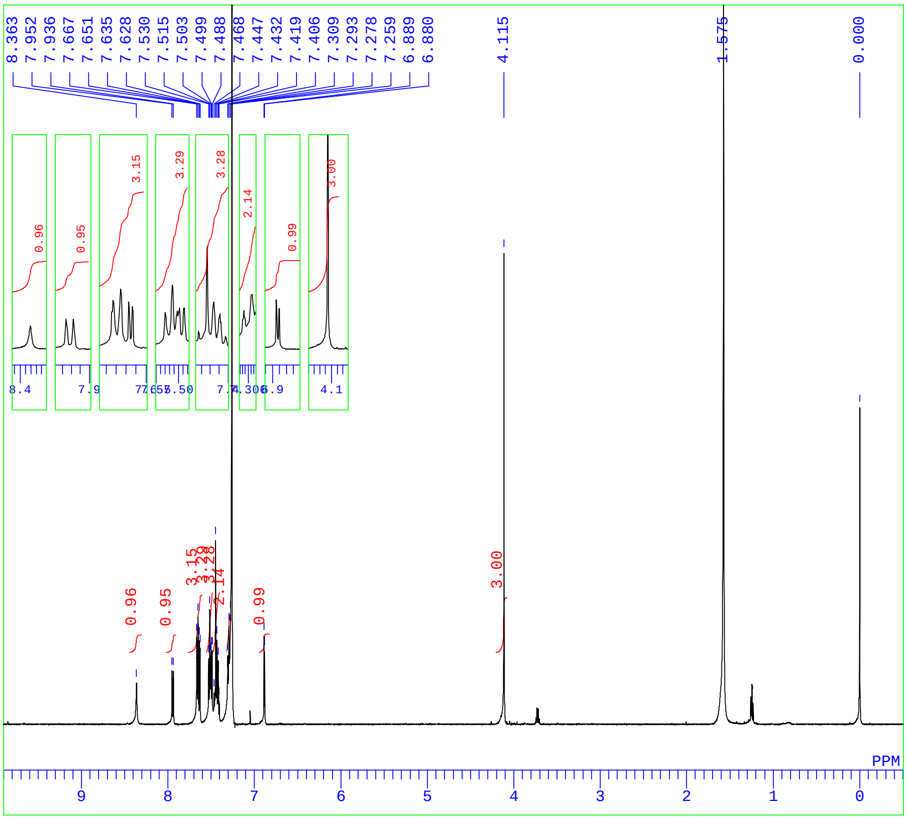

DFILE	7aD.als
COMNT	single_pulse
DATIM	2018-04-28 15:40:00
OBNUC	1H
EXMOD	single_pulse.ex2
OBFRQ	500.16 MHz
OBSET	2.41 KHz
OBFIN	6.01 Hz
POINT	13107
FREQU	7507.39 Hz
SCANS	16
ACQTM	1.7459 sec
PD	5.0000 sec
PW1	7.15 usec
IRNUC	1H
CTEMP	21.5 c
SLVNT	CDCL3
EXREF	0.00 ppm
BF	0.12 Hz
RGAIN	50

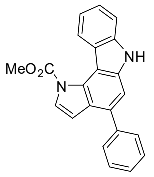

7aD

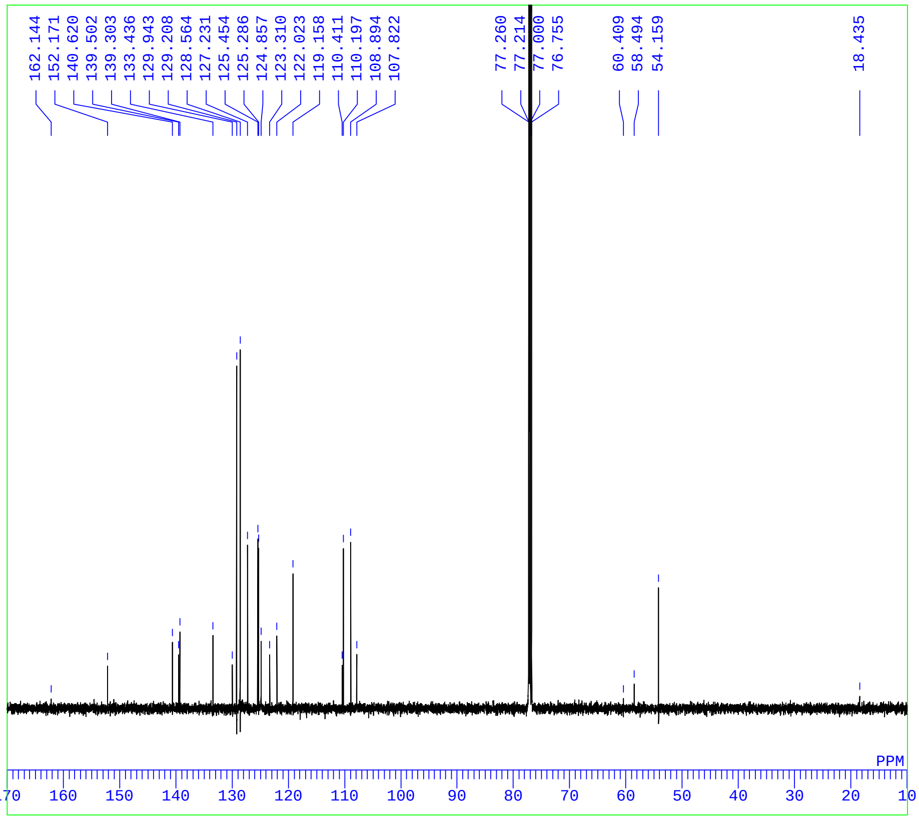

DFILE	7aD C.als
COMNT	
DATIM	2018-04-28 21:43:57
OBNUC	13C
EXMOD	single_pulse_dec
OBFRQ	125.77 MHz
OBSET	7.87 KHz
OBFIN	4.21 Hz
POINT	16384
FREQU	31565.66 Hz
SCANS	5000
ACQTM	0.5190 sec
PD	1.0000 sec
PW1	3.67 usec
IRNUC	1H
CTEMP	21.5 c
SLVNT	CDCL3
EXREF	77.00 ppm
BF	0.12 Hz
RGAIN	60

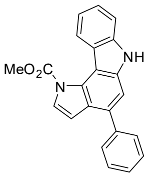

7aD

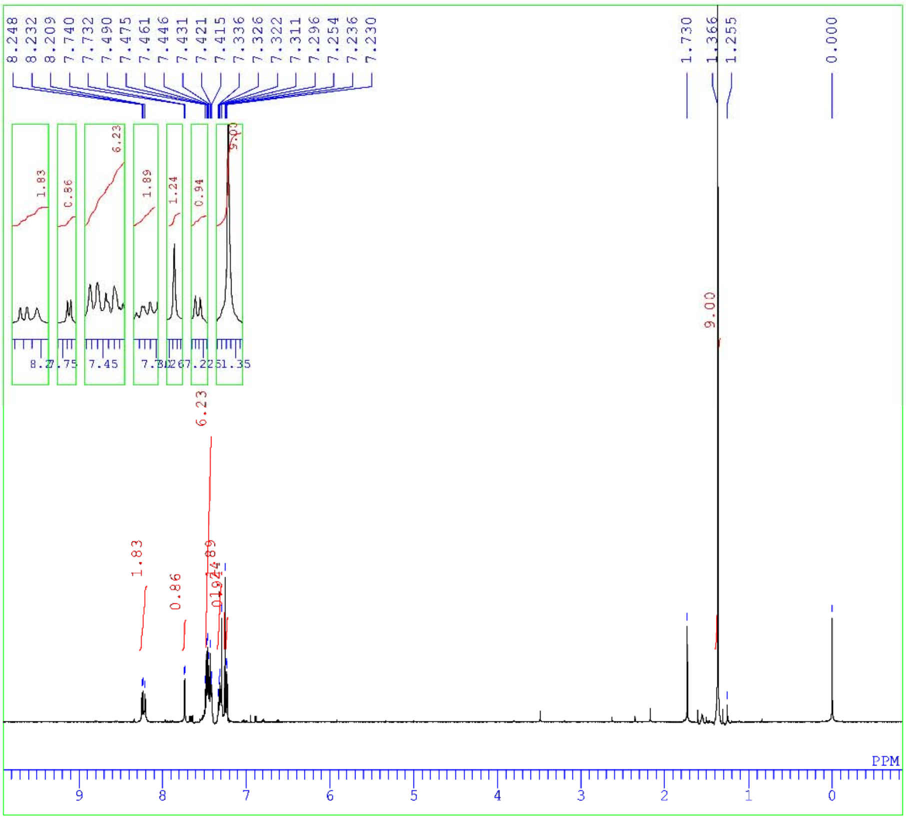

DFILE D-22.als
COMNT single_pulse
DATIM 2018-03-02 15:21:53
OBNUC 1H
EXMOD single_pulse.ex2
OBFRQ 500.16 MHz
OBSET 2.41 KHz
OBFIN 6.01 Hz
POINT 13107
FREQU 7507.39 Hz
SCANS 8
ACQTM 1.7459 sec
PD 5.0000 sec
PW1 7.15 usec
IRNUC 1H
CTEMP 19.3 c
SLVNT CDCL3
EXREF 0.00 ppm
BF 0.12 Hz
RGAIN 48

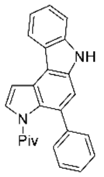

+ minor isomer

6aE

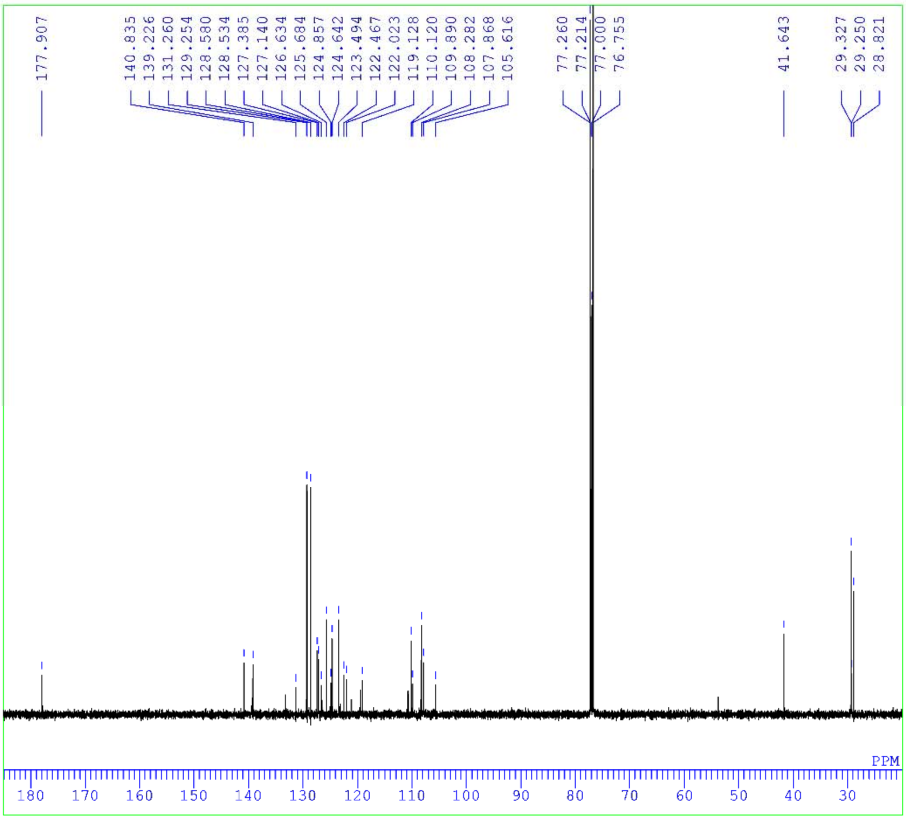

DFILE D22 C.als
COMNT
DATIM 2018-05-23 17:33:15
OBNUC 13C
EXMOD single_pulse_dec
OBFRQ 125.77 MHz
OBSET 7.87 KHz
OBFIN 4.21 Hz
POINT 13107
FREQU 25252.14 Hz
SCANS 2200
ACQTM 0.5190 sec
PD 1.0000 sec
PW1 3.67 usec
IRNUC 1H
CTEMP 21.8 c
SLVNT CDCL3
EXREF 77.00 ppm
BF 0.12 Hz
RGAIN 60

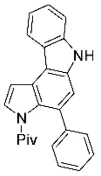

+ minor isomer

6aE

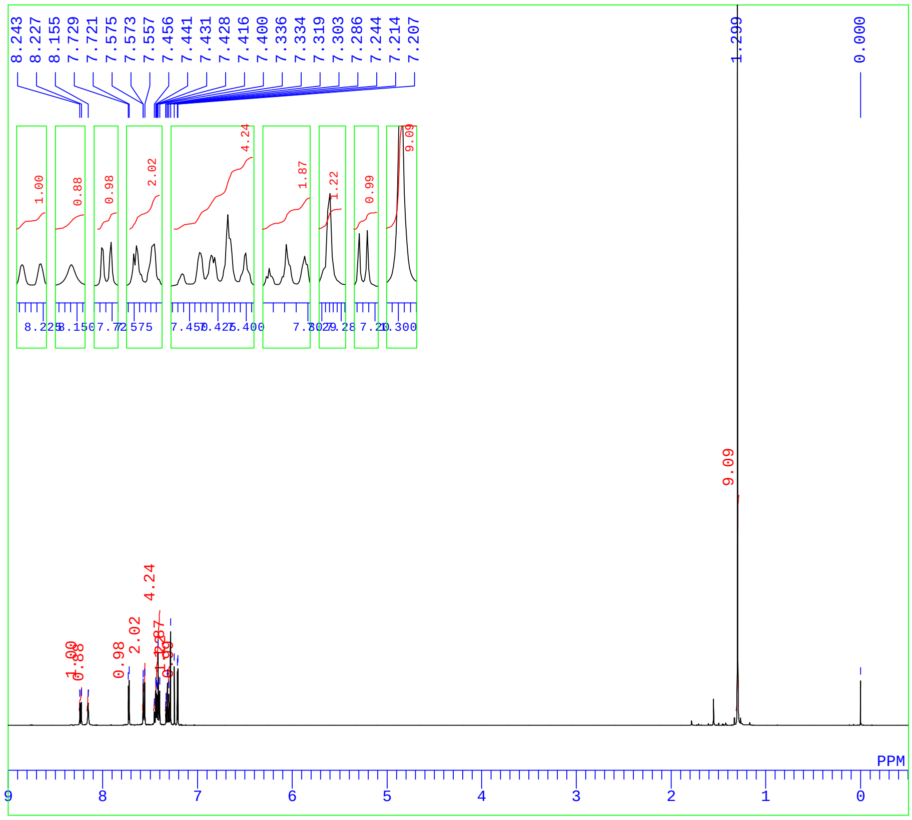


```
DFILE  6aF H-1.als
COMNT  single_pulse
DATIM  2017-02-24 16:33:32
OBNUC  1H
EXMOD  single_pulse.ex2
OBFRQ        500.16 MHz
OBSET          2.41 KHz
OBFIN          6.01 Hz
POINT         13107
FREQU       7507.39 Hz
SCANS             8
ACQTM        1.7459 sec
PD           5.0000 sec
PW1            7.15 usec
IRNUC  1H
CTEMP          19.6 c
SLVNT  CDCL3
EXREF          0.00 ppm
BF             0.12 Hz
RGAIN            44
```

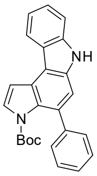

6aF

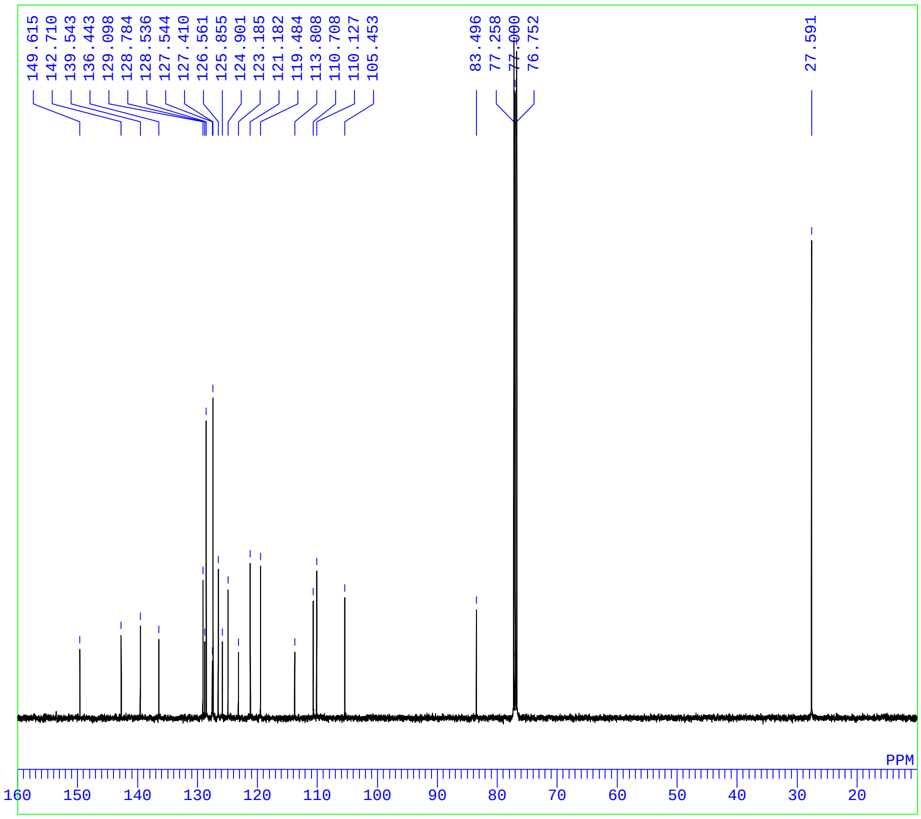


```
DFILE  6aF C-1.als
COMNT
DATIM  2017-02-24 06:01:34
OBNUC  13C
EXMOD  single_pulse_dec
OBFRQ        125.77 MHz
OBSET          7.87 KHz
OBFIN          4.21 Hz
POINT         26214
FREQU      31446.06 Hz
SCANS          1024
ACQTM        0.8336 sec
PD           2.0000 sec
PW1            3.67 usec
IRNUC  1H
CTEMP          20.1 c
SLVNT  CDCL3
EXREF         77.00 ppm
BF             1.20 Hz
RGAIN            60
```

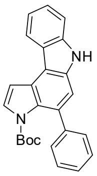

6aF

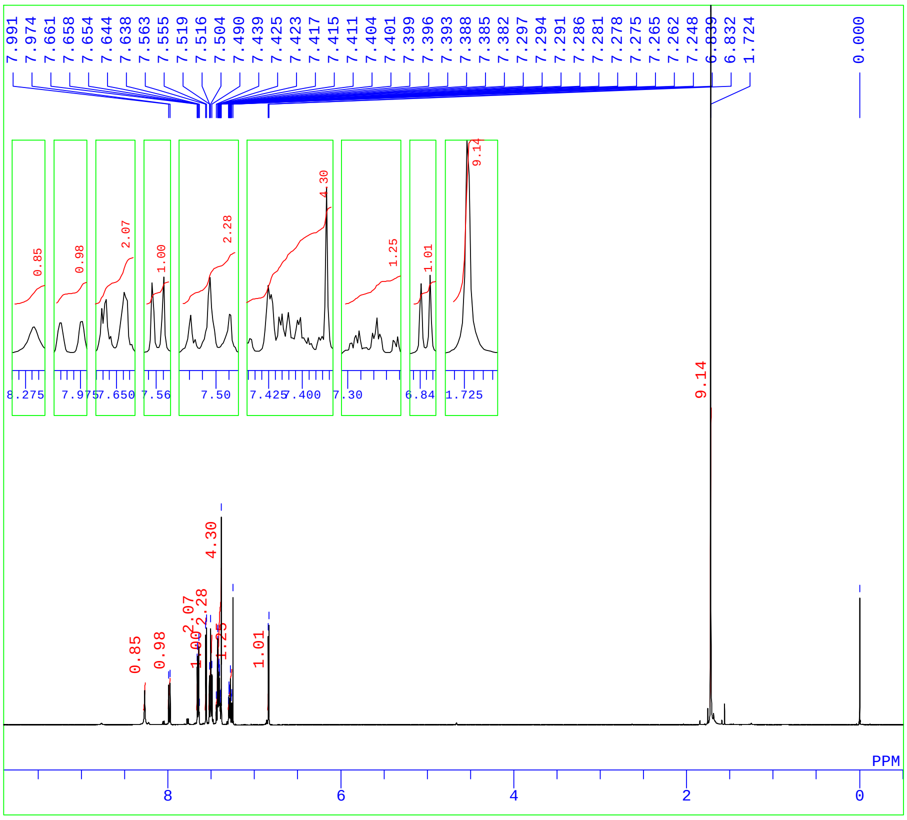

DFILE 7aF H-1.als
COMNT single_pulse
DATIM 2017-02-23 21:44:59
OBNUC 1H
EXMOD single_pulse.ex2
OBFRQ 500.16 MHz
OBSET 2.41 KHz
OBFIN 6.01 Hz
POINT 13107
FREQU 7507.39 Hz
SCANS 8
ACQTM 1.7459 sec
PD 5.0000 sec
PW1 7.15 usec
IRNUC 1H
CTEMP 19.6 c
SLVNT CDCL3
EXREF 0.00 ppm
BF 0.12 Hz
RGAIN 44

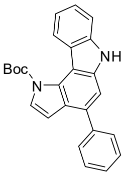

7aF

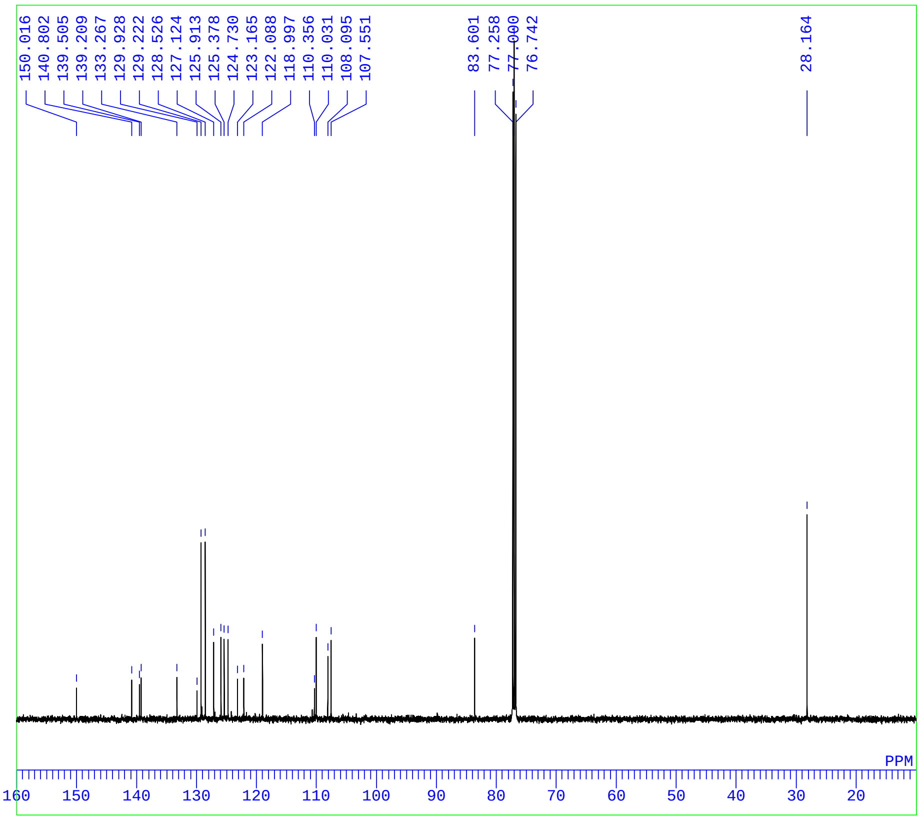

DFILE 7aF C-1.als
COMNT
DATIM 2017-02-24 06:56:18
OBNUC 13C
EXMOD single_pulse_dec
OBFRQ 125.77 MHz
OBSET 7.87 KHz
OBFIN 4.21 Hz
POINT 26214
FREQU 31446.06 Hz
SCANS 1024
ACQTM 0.8336 sec
PD 2.0000 sec
PW1 3.67 usec
IRNUC 1H
CTEMP 20.0 c
SLVNT CDCL3
EXREF 77.00 ppm
BF 1.20 Hz
RGAIN 60

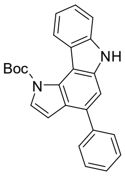

7aF

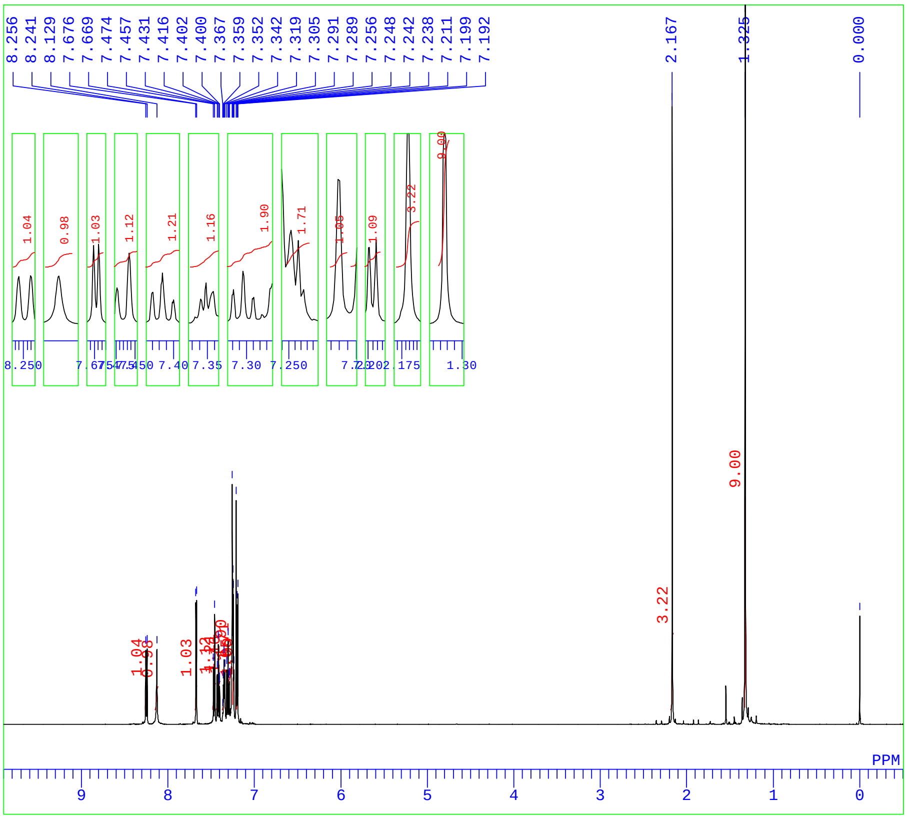

DFILE C-34 ue H 1 26-1.als
COMNT single_pulse
DATIM 2017-01-26 21:24:44
OBNUC 1H
EXMOD single_pulse.ex2
OBFRQ 500.16 MHz
OBSET 2.41 KHz
OBFIN 6.01 Hz
POINT 13107
FREQU 7507.39 Hz
SCANS 8
ACQTM 1.7459 sec
PD 5.0000 sec
PW1 7.15 usec
IRNUC 1H
CTEMP 18.9 c
SLVNT CDCL3
EXREF 0.00 ppm
BF 0.12 Hz
RGAIN 42

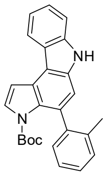

6bF

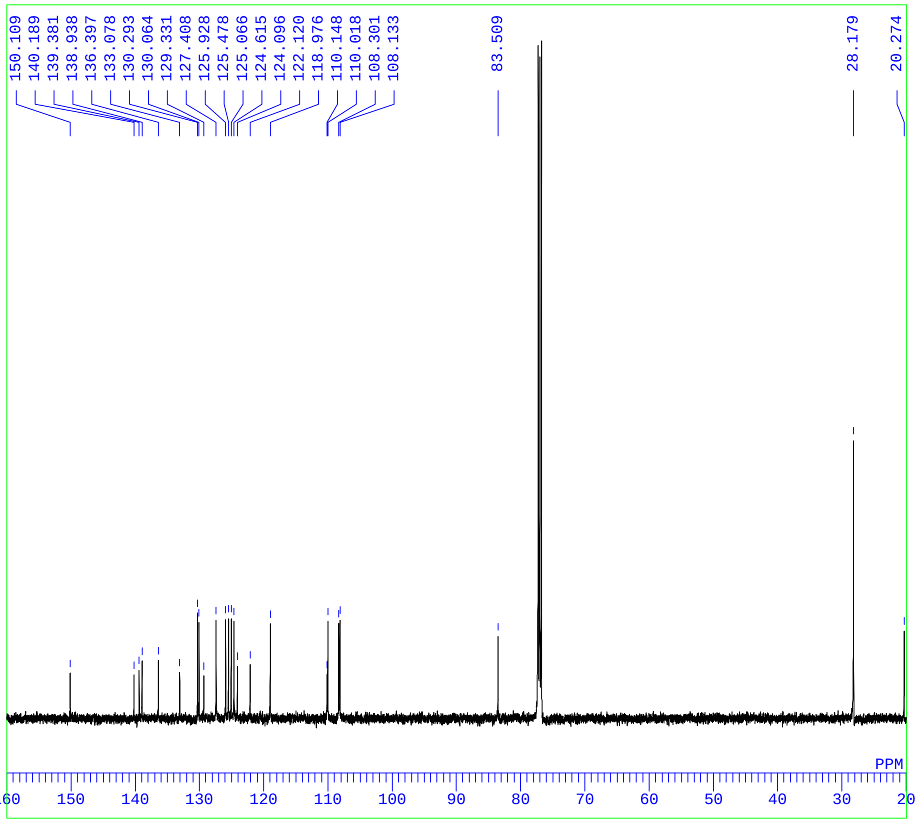

DFILE C-34 sita C 2 3-1.als
COMNT
DATIM 2017-02-03 14:26:05
OBNUC 13C
EXMOD single_pulse_dec
OBFRQ 125.77 MHz
OBSET 7.87 KHz
OBFIN 4.21 Hz
POINT 32768
FREQU 31446.54 Hz
SCANS 1024
ACQTM 1.0420 sec
PD 2.0000 sec
PW1 3.67 usec
IRNUC 1H
CTEMP 20.3 c
SLVNT CDCL3
EXREF 77.00 ppm
BF 1.20 Hz
RGAIN 70

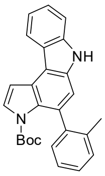

6bF

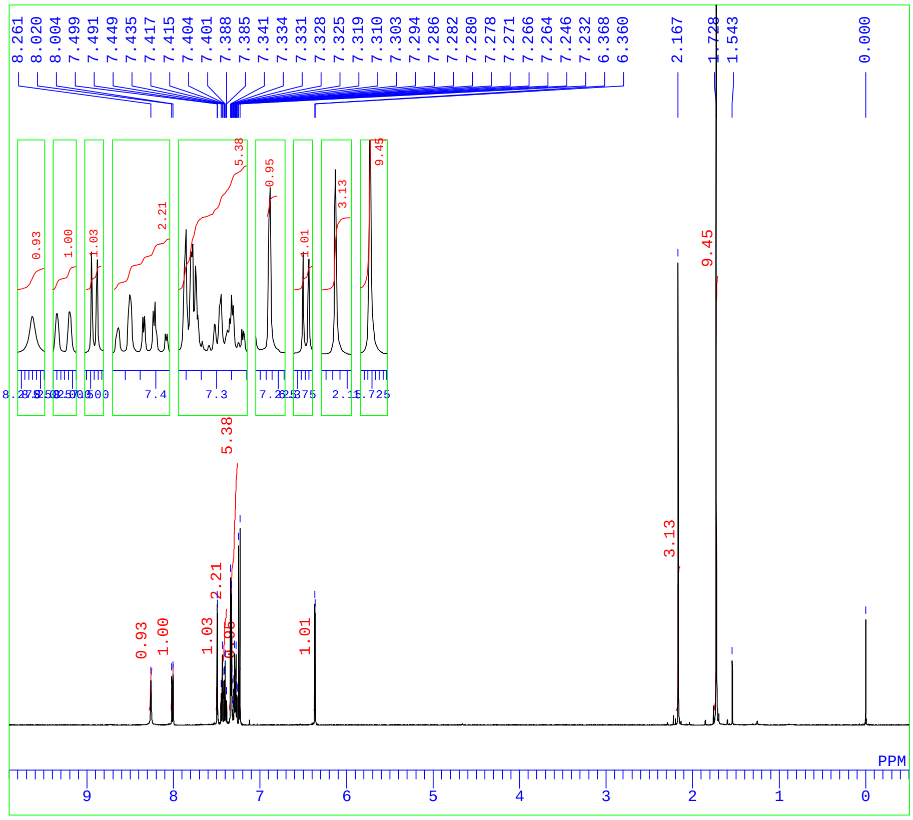

DFILE C-34 d H.als
COMNT single_pulse
DATIM 2017-02-08 13:25:11
OBNUC 1H
EXMOD single_pulse.ex2
OBFRQ 500.16 MHz
OBSET 2.41 KHz
OBFIN 6.01 Hz
POINT 13107
FREQU 7507.39 Hz
SCANS 8
ACQTM 1.7459 sec
PD 5.0000 sec
PW1 7.15 usec
IRNUC 1H
CTEMP 20.4 c
SLVNT CDCL3
EXREF 0.00 ppm
BF 0.12 Hz
RGAIN 44

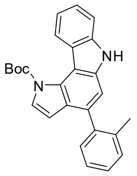

7bF

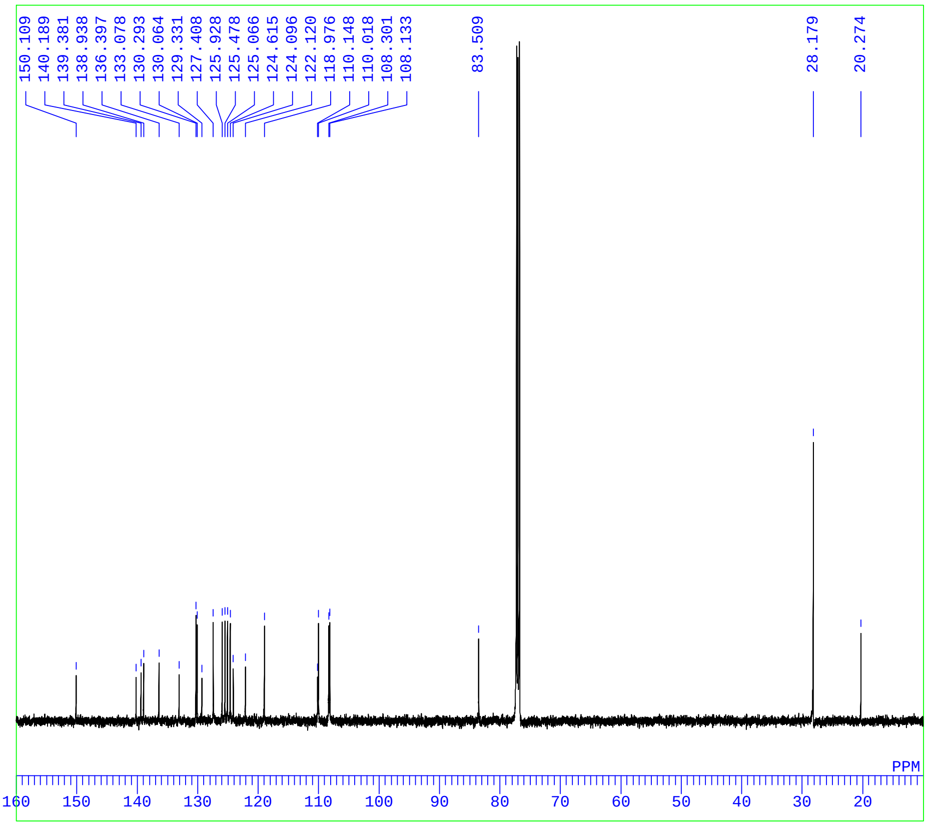

DFILE C-34 d C.als
COMNT
DATIM 2017-02-03 14:26:05
OBNUC 13C
EXMOD single_pulse_dec
OBFRQ 125.77 MHz
OBSET 7.87 KHz
OBFIN 4.21 Hz
POINT 32768
FREQU 31446.54 Hz
SCANS 1024
ACQTM 1.0420 sec
PD 2.0000 sec
PW1 3.67 usec
IRNUC 1H
CTEMP 20.3 c
SLVNT CDCL3
EXREF 77.00 ppm
BF 1.20 Hz
RGAIN 70

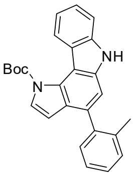

7bF

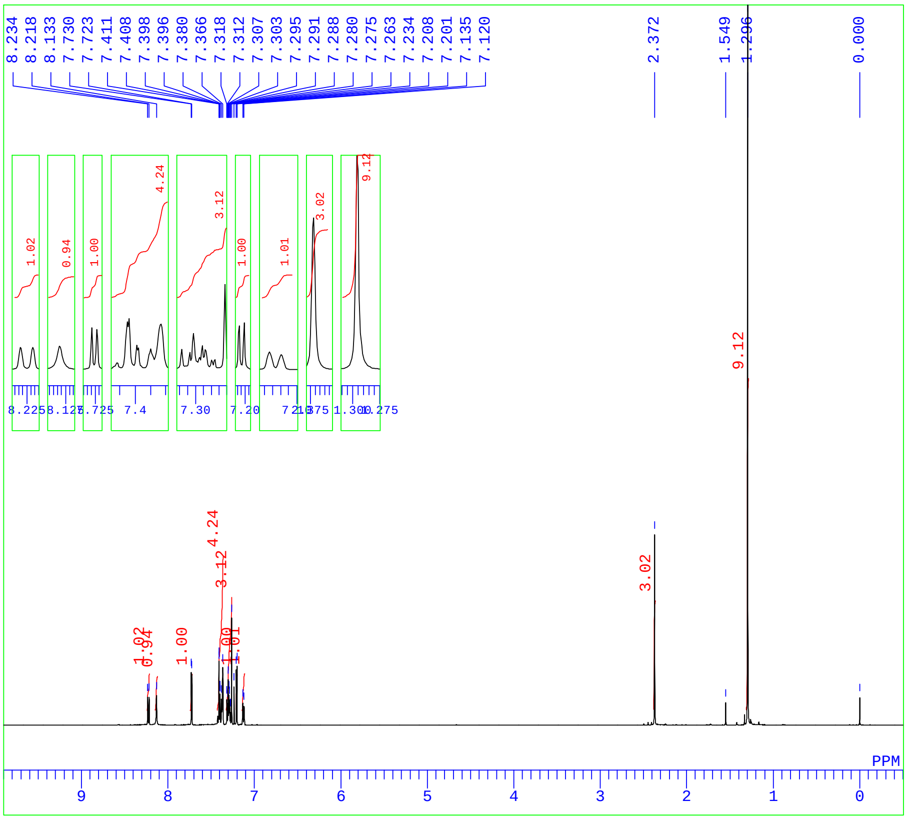

DFILE C-30 u H.als
COMNT single_pulse
DATIM 2017-01-27 20:56:05
OBNUC 1H
EXMOD single_pulse.ex2
OBFRQ 500.16 MHz
OBSET 2.41 KHz
OBFIN 6.01 Hz
POINT 13107
FREQU 7507.39 Hz
SCANS 8
ACQTM 1.7459 sec
PD 5.0000 sec
PW1 7.15 usec
IRNUC 1H
CTEMP 19.6 c
SLVNT CDCL3
EXREF 0.00 ppm
BF 0.12 Hz
RGAIN 40

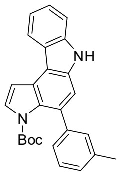

6cF

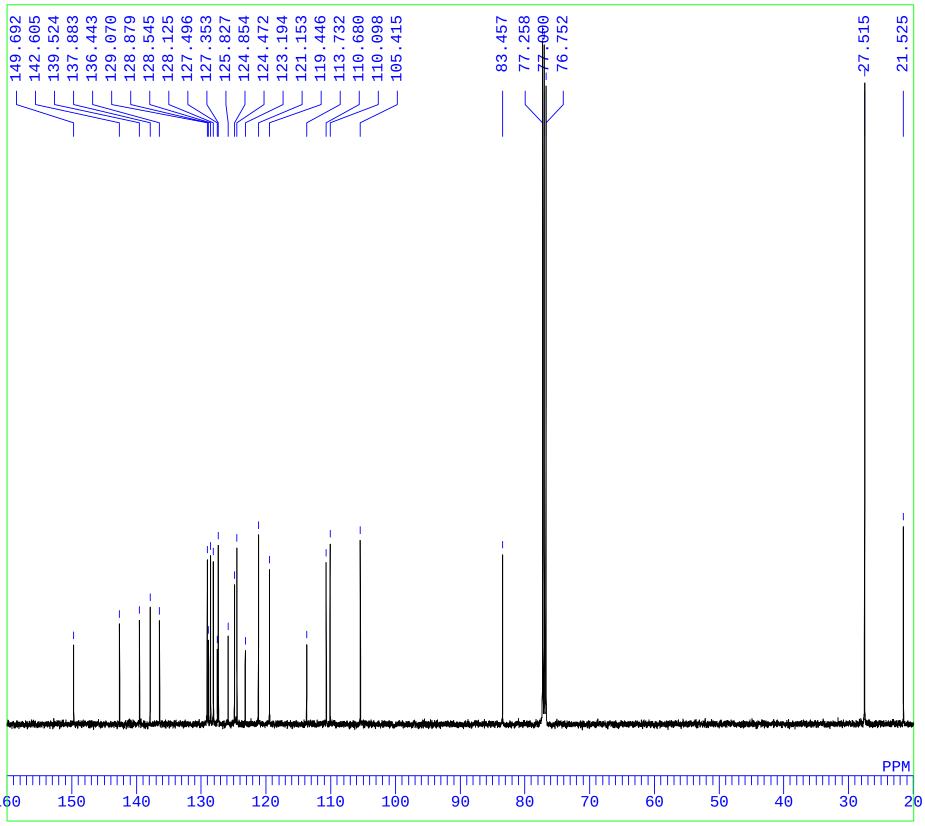

DFILE C-30 u C.als
COMNT
DATIM 2017-01-26 23:51:11
OBNUC 13C
EXMOD single_pulse_dec
OBFRQ 125.77 MHz
OBSET 7.87 KHz
OBFIN 4.21 Hz
POINT 26214
FREQU 31446.06 Hz
SCANS 1024
ACQTM 0.8336 sec
PD 2.0000 sec
PW1 3.67 usec
IRNUC 1H
CTEMP 20.0 c
SLVNT CDCL3
EXREF 77.00 ppm
BF 1.20 Hz
RGAIN 54

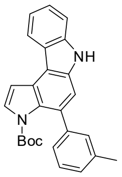

6cF

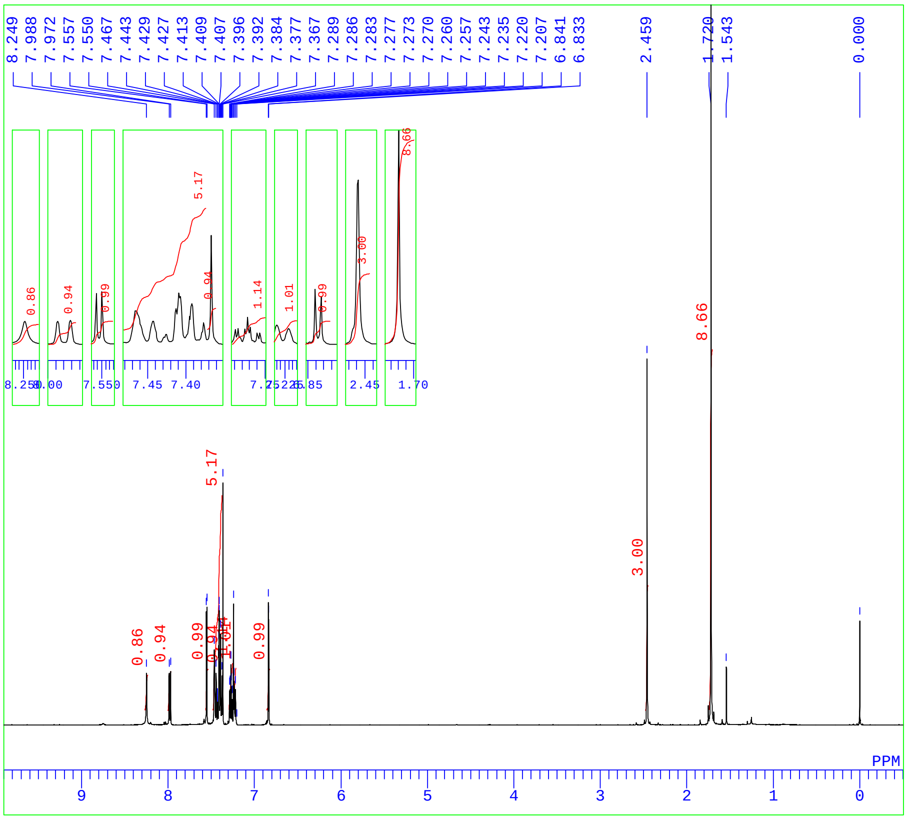

DFILE C-30 d H.als
COMNT single_pulse
DATIM 2017-01-27 21:01:29
OBNUC 1H
EXMOD single_pulse.ex2
OBFRQ 500.16 MHz
OBSET 2.41 KHz
OBFIN 6.01 Hz
POINT 13107
FREQU 7507.39 Hz
SCANS 8
ACQTM 1.7459 sec
PD 5.0000 sec
PW1 7.15 usec
IRNUC 1H
CTEMP 19.5 c
SLVNT CDCL3
EXREF 0.00 ppm
BF 0.12 Hz
RGAIN 44

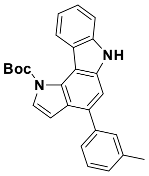

7cF

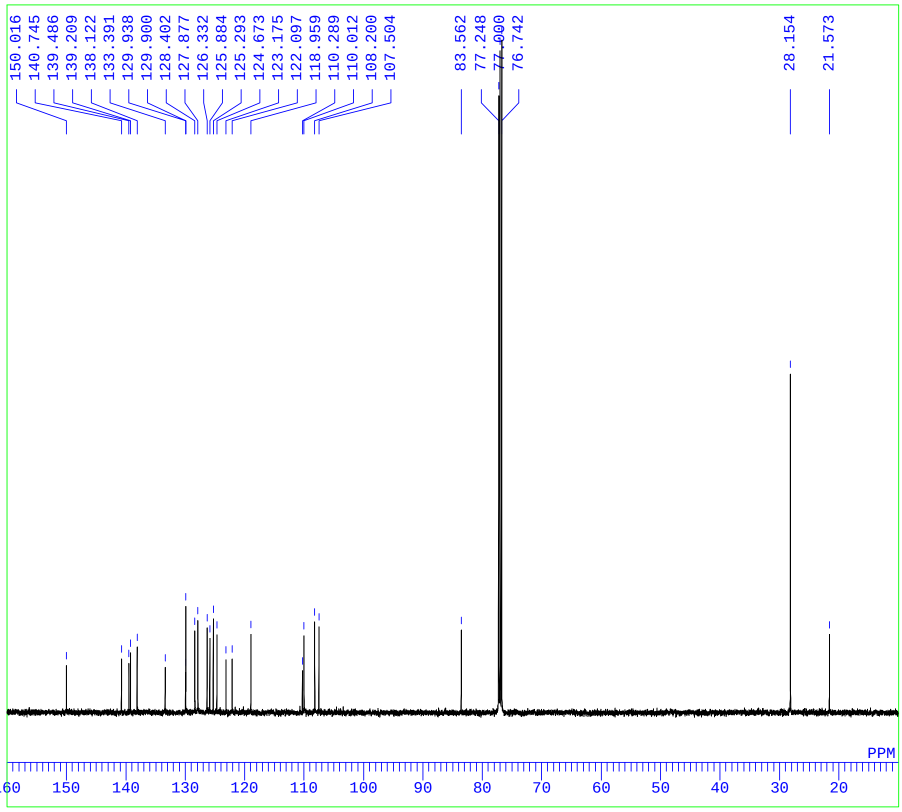

DFILE C-30 d C.als
COMNT
DATIM 2017-01-27 00:53:29
OBNUC 13C
EXMOD single_pulse_dec
OBFRQ 125.77 MHz
OBSET 7.87 KHz
OBFIN 4.21 Hz
POINT 26214
FREQU 31446.06 Hz
SCANS 1024
ACQTM 0.8336 sec
PD 2.0000 sec
PW1 3.67 usec
IRNUC 1H
CTEMP 19.8 c
SLVNT CDCL3
EXREF 77.00 ppm
BF 1.20 Hz
RGAIN 58

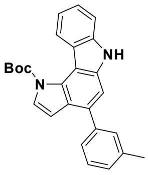

7cF

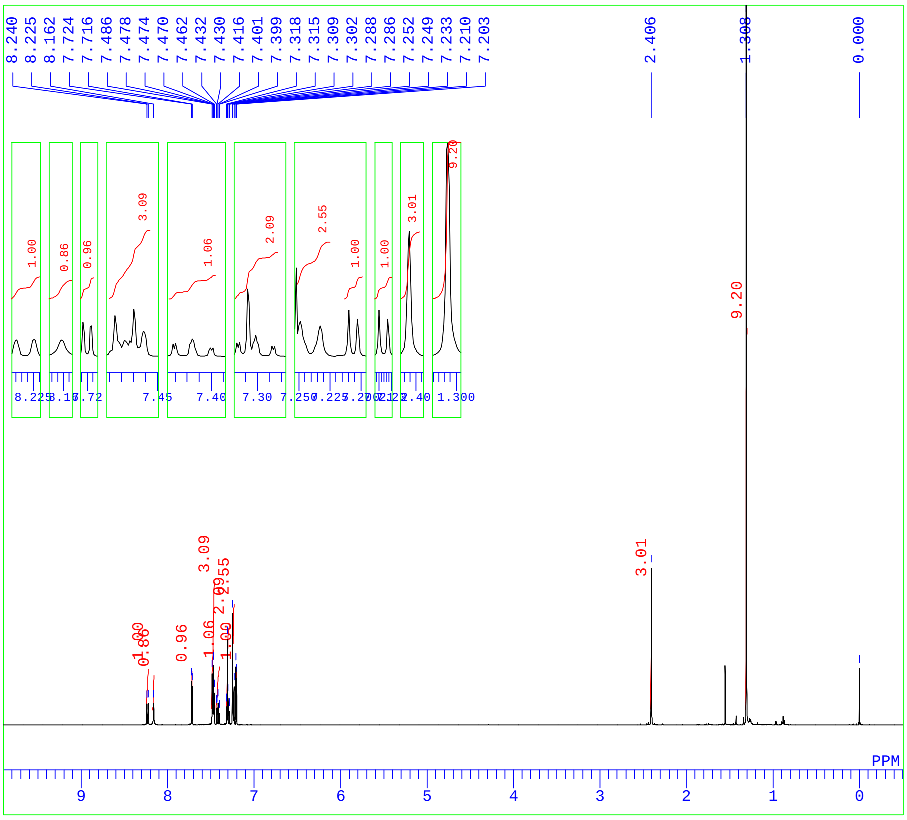

DFILE C-35 u H.als
COMNT single_pulse
DATIM 2017-02-24 16:39:36
OBNUC 1H
EXMOD single_pulse.ex2
OBFRQ 500.16 MHz
OBSET 2.41 KHz
OBFIN 6.01 Hz
POINT 13107
FREQU 7507.39 Hz
SCANS 8
ACQTM 1.7459 sec
PD 5.0000 sec
PW1 7.15 usec
IRNUC 1H
CTEMP 19.7 c
SLVNT CDCL3
EXREF 0.00 ppm
BF 0.12 Hz
RGAIN 46

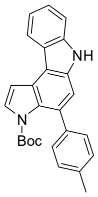

6dF

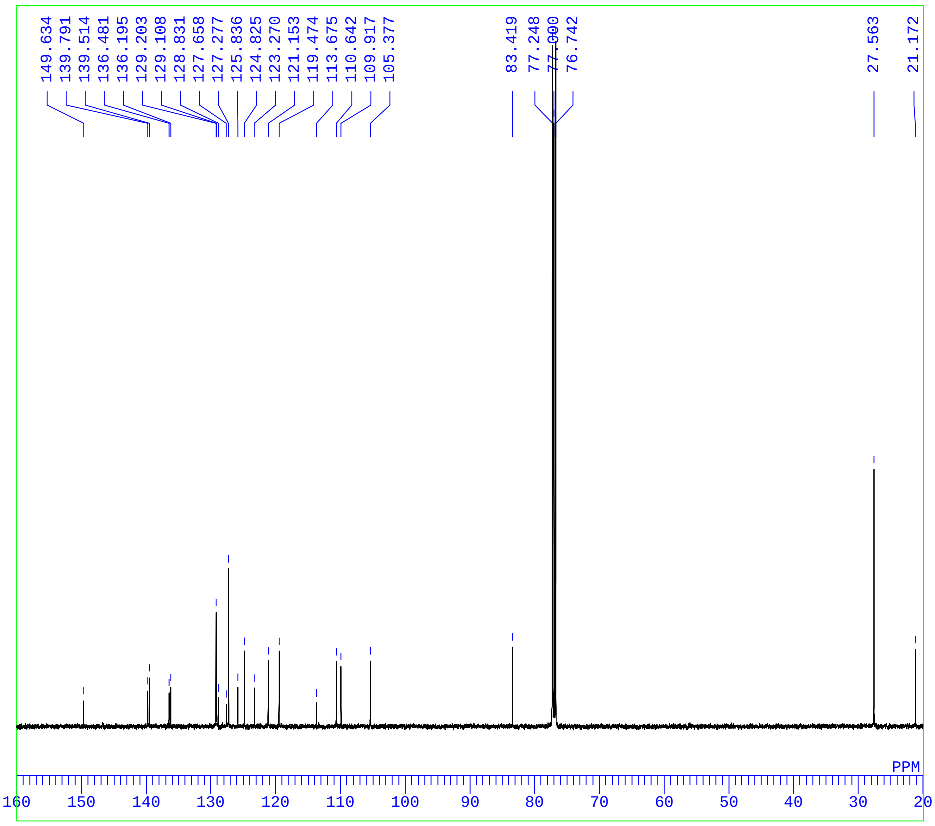

DFILE C-35 u C.als
COMNT
DATIM 2017-01-28 06:21:27
OBNUC 13C
EXMOD single_pulse_dec
OBFRQ 125.77 MHz
OBSET 7.87 KHz
OBFIN 4.21 Hz
POINT 26214
FREQU 31446.06 Hz
SCANS 2048
ACQTM 0.8336 sec
PD 2.0000 sec
PW1 3.67 usec
IRNUC 1H
CTEMP 20.8 c
SLVNT CDCL3
EXREF 77.00 ppm
BF 1.20 Hz
RGAIN 60

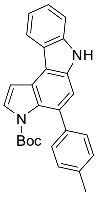

6dF

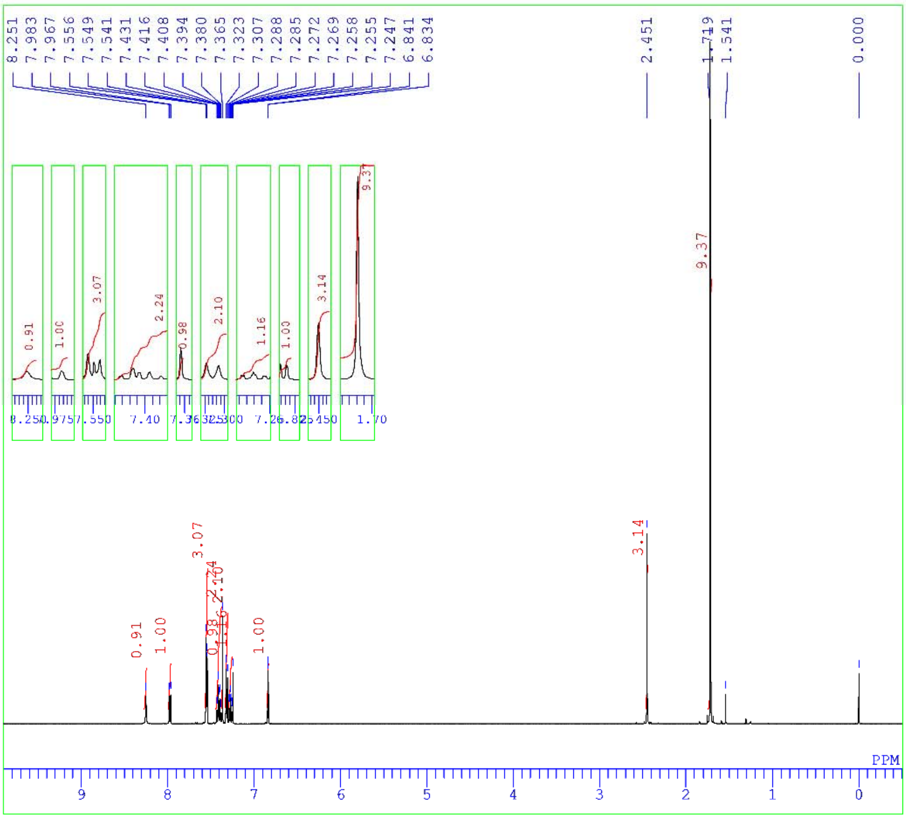

DFILE C-35 d H.als
COMNT single_pulse
DATIM 2017-01-27 21:23:20
OBNUC 1H
EXMOD single_pulse.ex2
OBFRQ 500.16 MHz
OBSET 2.41 KHz
OBFIN 6.01 Hz
POINT 13107
FREQU 7507.39 Hz
SCANS 8
ACQTM 1.7459 sec
PD 5.0000 sec
PW1 7.15 usec
IRNUC 1H
CTEMP 19.6 c
SLVNT CDCL3
EXREF 0.00 ppm
BF 0.12 Hz
RGAIN 44

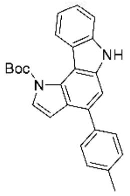

7dF

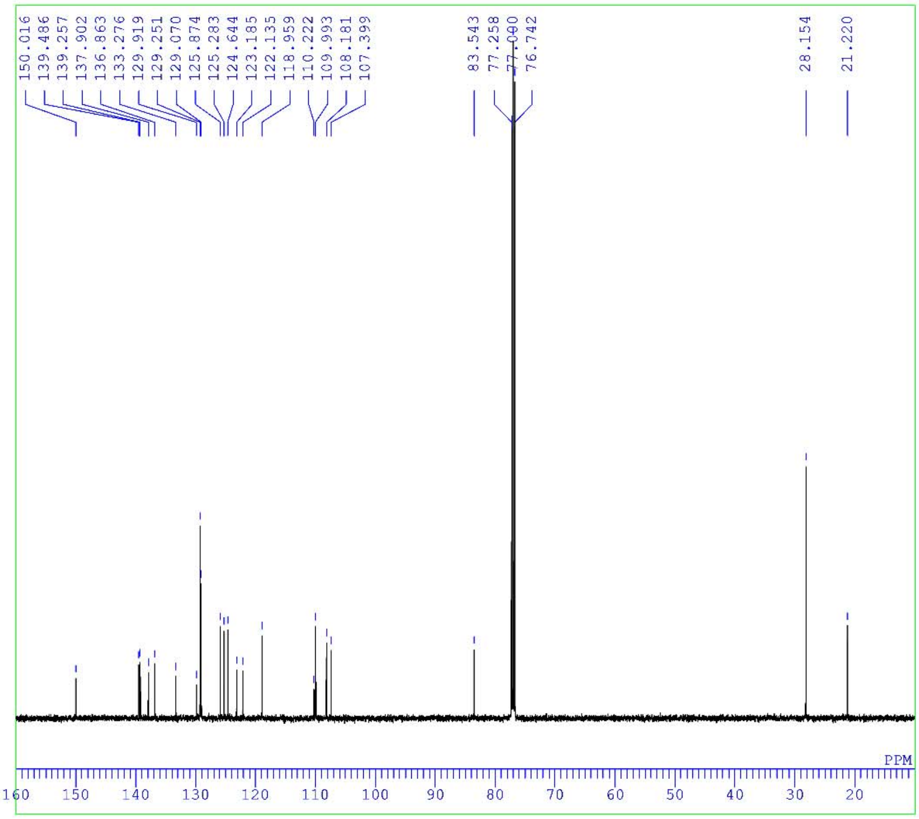

DFILE C-35 d C.als
COMNT
DATIM 2017-01-28 07:16:31
OBNUC 13C
EXMOD single_pulse_dec
OBFRQ 125.77 MHz
OBSET 7.87 KHz
OBFIN 4.21 Hz
POINT 26214
FREQU 31446.06 Hz
SCANS 1024
ACQTM 0.8336 sec
PD 2.0000 sec
PW1 3.67 usec
IRNUC 1H
CTEMP 20.8 c
SLVNT CDCL3
EXREF 77.00 ppm
BF 1.20 Hz
RGAIN 60

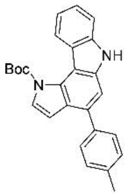

7dF

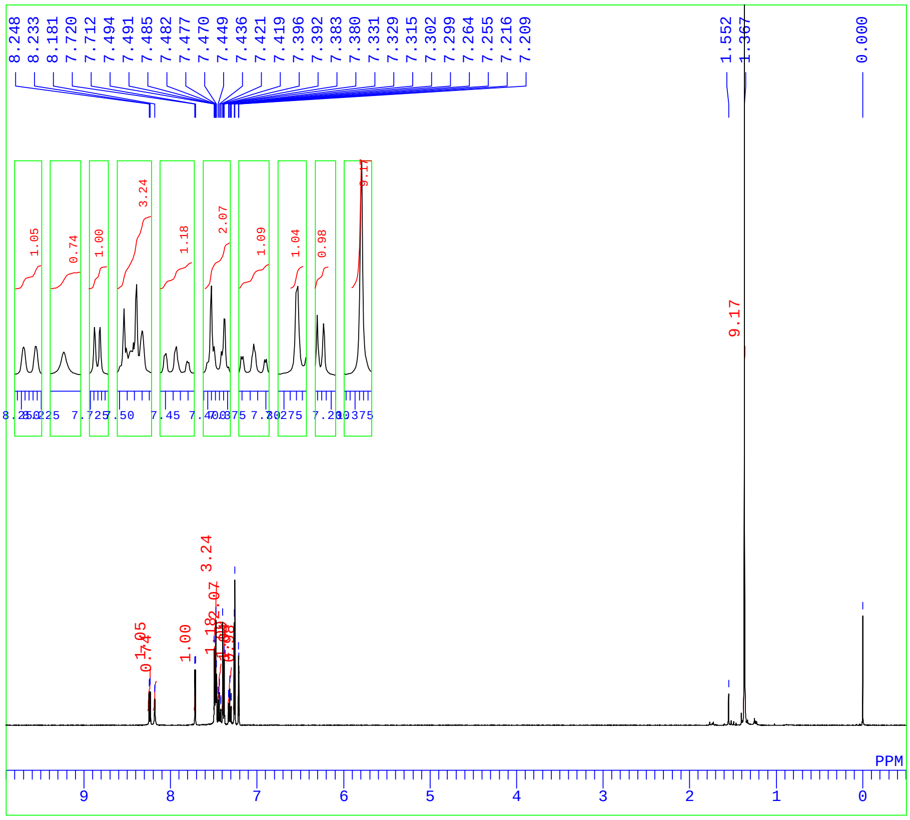

DFILE C-33 u H.als
COMNT single_pulse
DATIM 2017-02-09 13:23:17
OBNUC 1H
EXMOD single_pulse.ex2
OBFRQ 500.16 MHz
OBSET 2.41 KHz
OBFIN 6.01 Hz
POINT 13107
FREQU 7507.39 Hz
SCANS 8
ACQTM 1.7459 sec
PD 5.0000 sec
PW1 7.15 usec
IRNUC 1H
CTEMP 20.1 c
SLVNT CDCL3
EXREF 0.00 ppm
BF 0.12 Hz
RGAIN 46

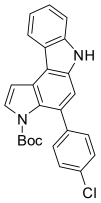

6eF

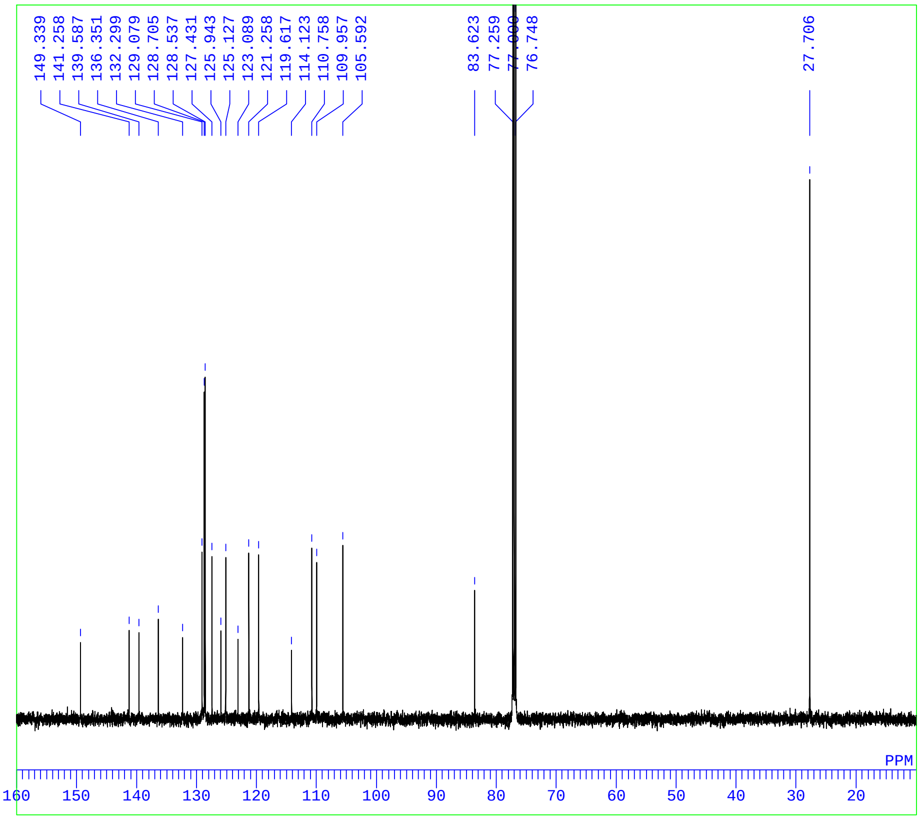

DFILE C33 u C.als
COMNT
DATIM 2017-02-03 12:29:04
OBNUC 13C
EXMOD single_pulse_dec
OBFRQ 125.77 MHz
OBSET 7.87 KHz
OBFIN 4.21 Hz
POINT 32768
FREQU 31446.54 Hz
SCANS 1024
ACQTM 1.0420 sec
PD 2.0000 sec
PW1 3.67 usec
IRNUC 1H
CTEMP 20.1 c
SLVNT CDCL3
EXREF 77.00 ppm
BF 1.20 Hz
RGAIN 70

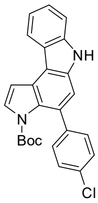

6eF

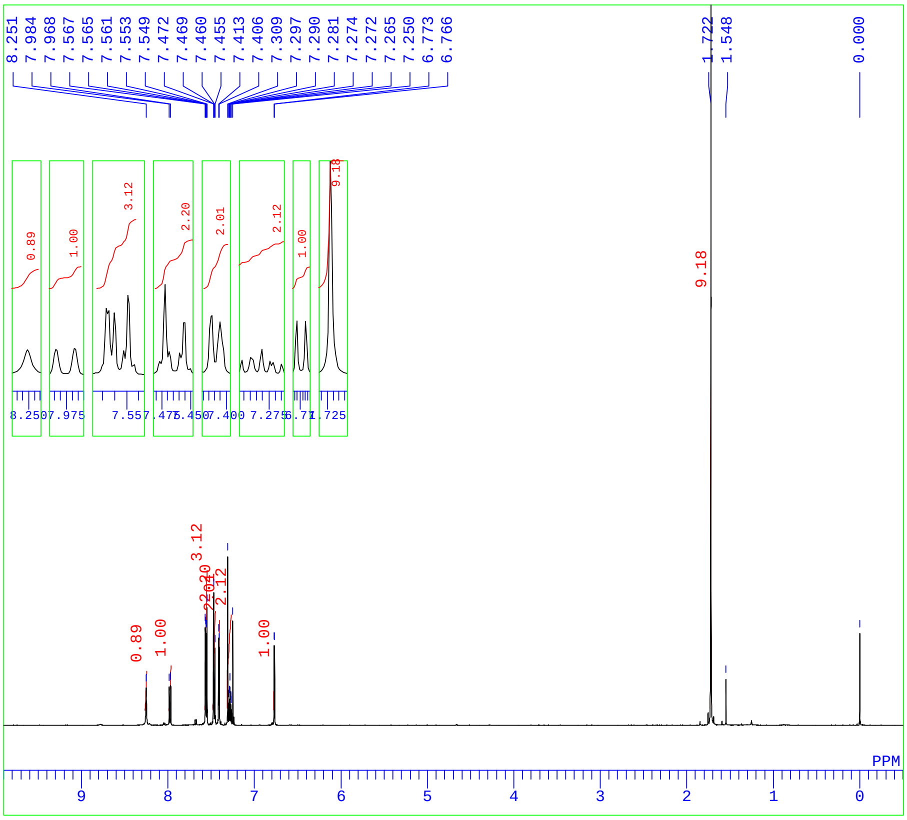

DFILE C-33 d H.als
COMNT single_pulse
DATIM 2017-01-27 21:12:06
OBNUC 1H
EXMOD single_pulse.ex2
OBFRQ 500.16 MHz
OBSET 2.41 KHz
OBFIN 6.01 Hz
POINT 13107
FREQU 7507.39 Hz
SCANS 8
ACQTM 1.7459 sec
PD 5.0000 sec
PW1 7.15 usec
IRNUC 1H
CTEMP 19.5 c
SLVNT CDCL3
EXREF 0.00 ppm
BF 0.12 Hz
RGAIN 46

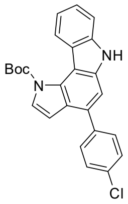

7eF

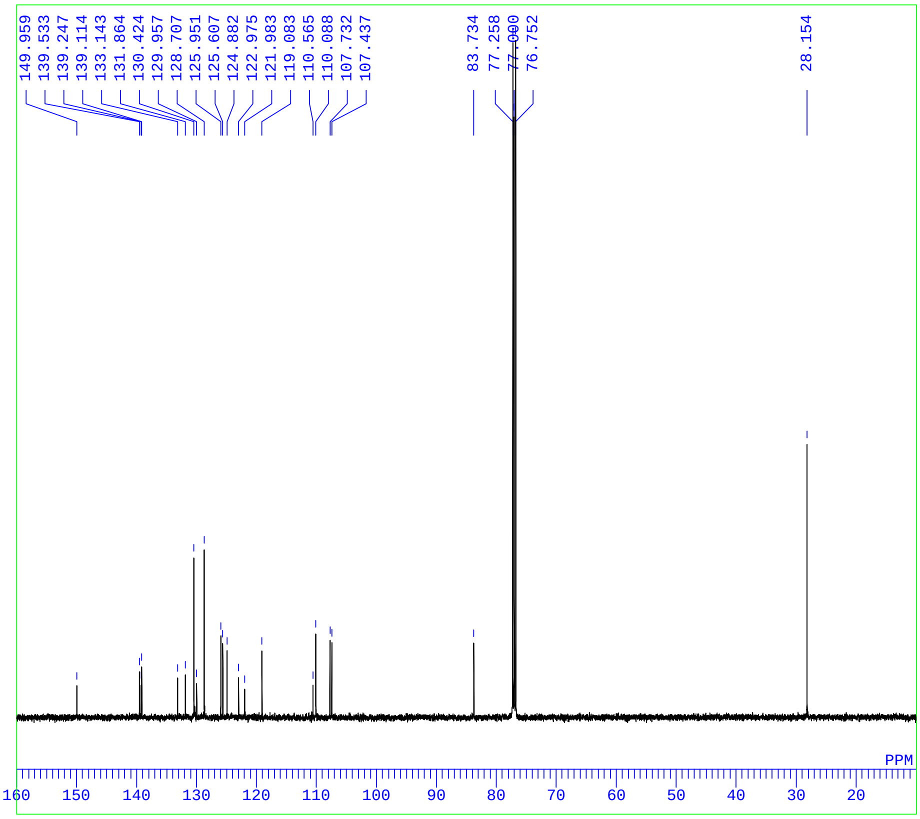

DFILE C-33 d C.als
COMNT
DATIM 2017-01-28 04:36:45
OBNUC 13C
EXMOD single_pulse_dec
OBFRQ 125.77 MHz
OBSET 7.87 KHz
OBFIN 4.21 Hz
POINT 26214
FREQU 31446.06 Hz
SCANS 1024
ACQTM 0.8336 sec
PD 2.0000 sec
PW1 3.67 usec
IRNUC 1H
CTEMP 20.9 c
SLVNT CDCL3
EXREF 77.00 ppm
BF 1.20 Hz
RGAIN 60

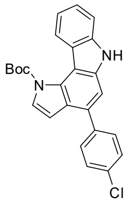

7eF

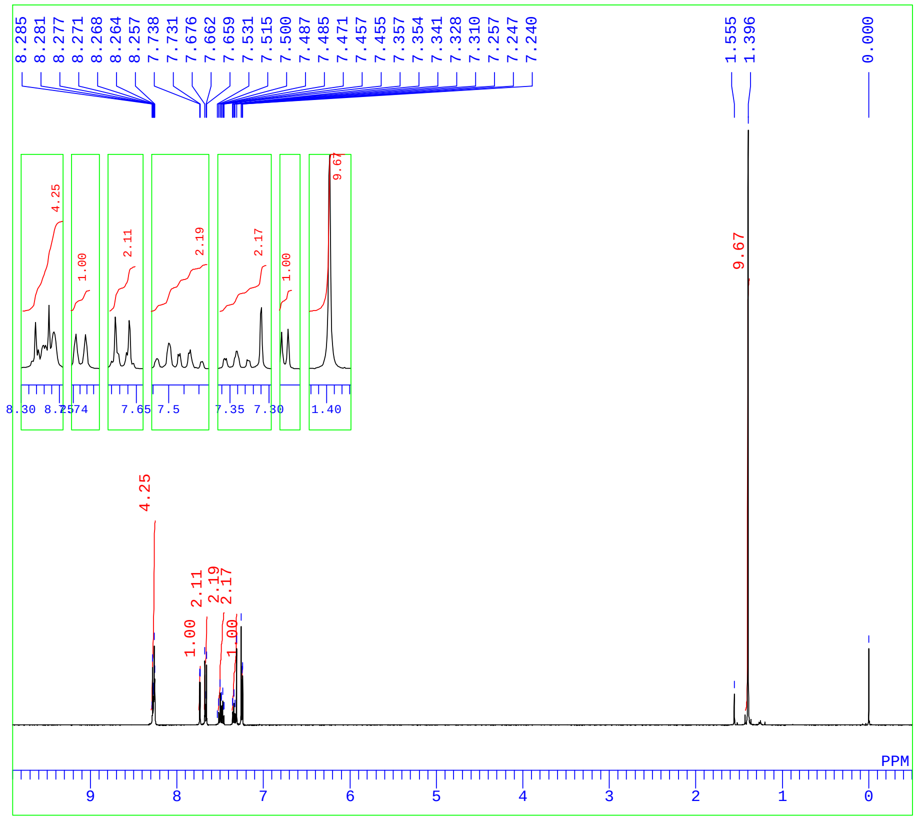

DFILE C-29 u H.als
COMNT single_pulse
DATIM 2017-02-08 13:05:22
OBNUC 1H
EXMOD single_pulse.ex2
OBFRQ 500.16 MHz
OBSET 2.41 KHz
OBFIN 6.01 Hz
POINT 13107
FREQU 7507.39 Hz
SCANS 8
ACQTM 1.7459 sec
PD 5.0000 sec
PW1 7.15 usec
IRNUC 1H
CTEMP 20.3 c
SLVNT CDCL3
EXREF 0.00 ppm
BF 0.12 Hz
RGAIN 48

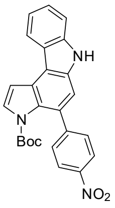

6fF

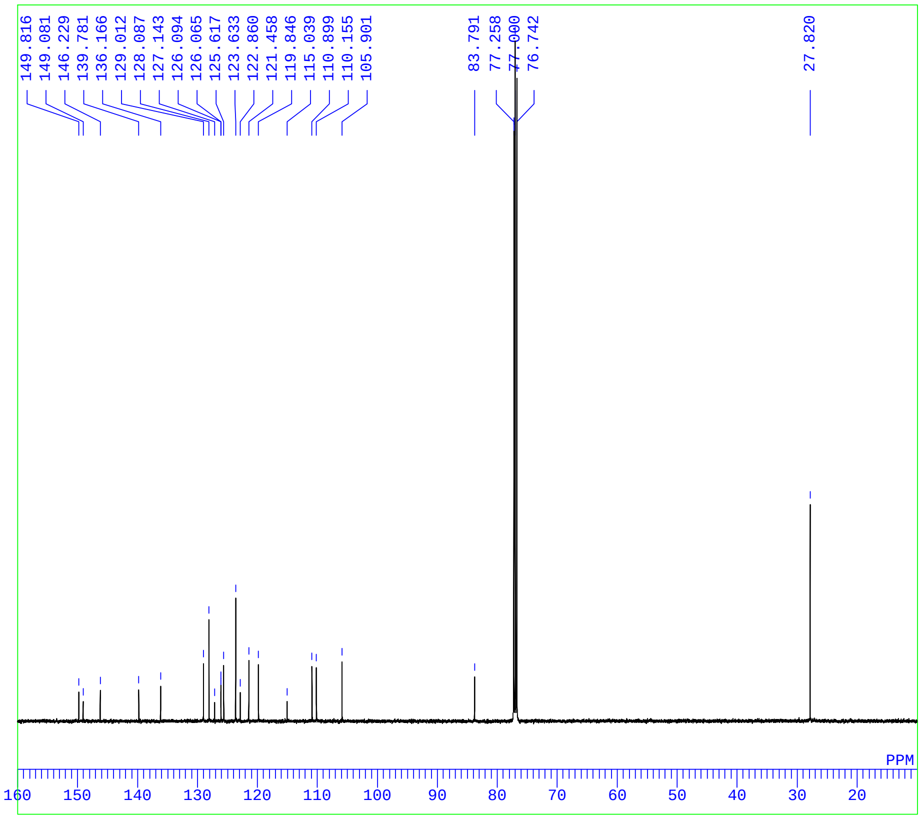

DFILE C-29 u C.als
COMNT
DATIM 2017-02-02 05:16:08
OBNUC 13C
EXMOD single_pulse_dec
OBFRQ 125.77 MHz
OBSET 7.87 KHz
OBFIN 4.21 Hz
POINT 26214
FREQU 31446.06 Hz
SCANS 3800
ACQTM 0.8336 sec
PD 2.0000 sec
PW1 3.67 usec
IRNUC 1H
CTEMP 20.2 c
SLVNT CDCL3
EXREF 77.00 ppm
BF 1.20 Hz
RGAIN 60

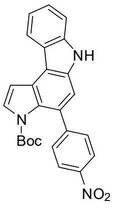

6fF

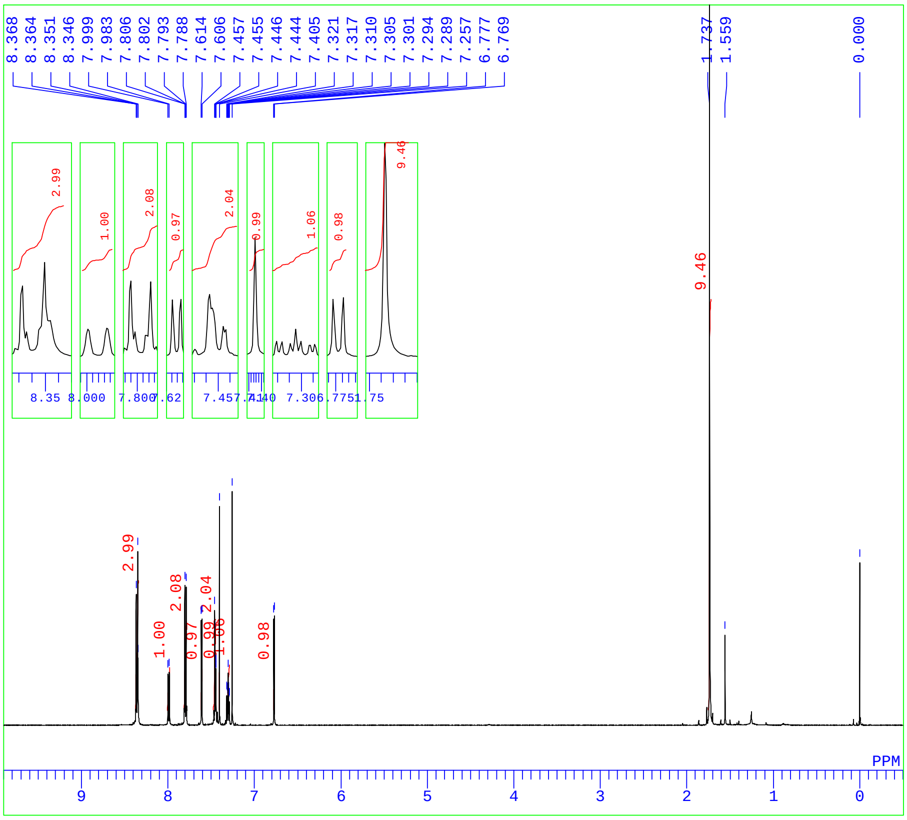

DFILE C-29 d H.als
COMNT single_pulse
DATIM 2017-02-08 13:11:43
OBNUC 1H
EXMOD single_pulse.ex2
OBFRQ 500.16 MHz
OBSET 2.41 KHz
OBFIN 6.01 Hz
POINT 13107
FREQU 7507.39 Hz
SCANS 8
ACQTM 1.7459 sec
PD 5.0000 sec
PW1 7.15 usec
IRNUC 1H
CTEMP 20.4 c
SLVNT CDCL3
EXREF 0.00 ppm
BF 0.12 Hz
RGAIN 46

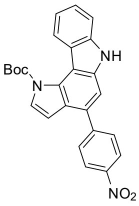

7fF

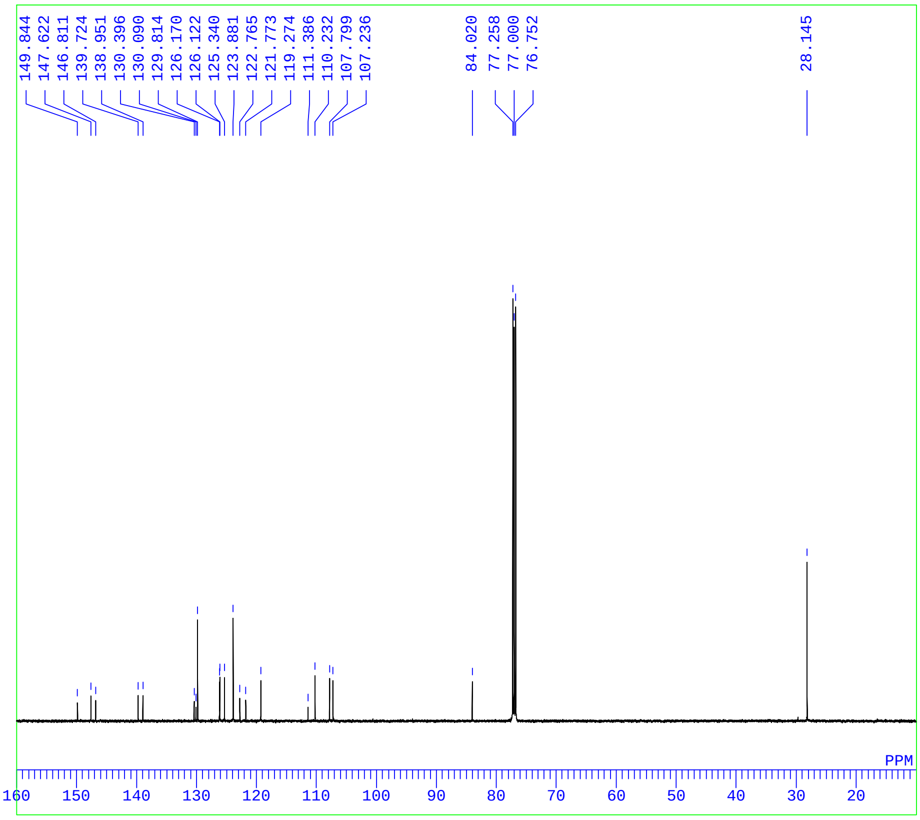

DFILE C-29 d C.als
COMNT
DATIM 2017-02-02 08:27:49
OBNUC 13C
EXMOD single_pulse_dec
OBFRQ 125.77 MHz
OBSET 7.87 KHz
OBFIN 4.21 Hz
POINT 26214
FREQU 31446.06 Hz
SCANS 3800
ACQTM 0.8336 sec
PD 2.0000 sec
PW1 3.67 usec
IRNUC 1H
CTEMP 19.9 c
SLVNT CDCL3
EXREF 77.00 ppm
BF 1.20 Hz
RGAIN 60

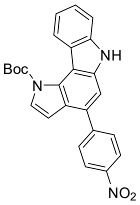

7fF

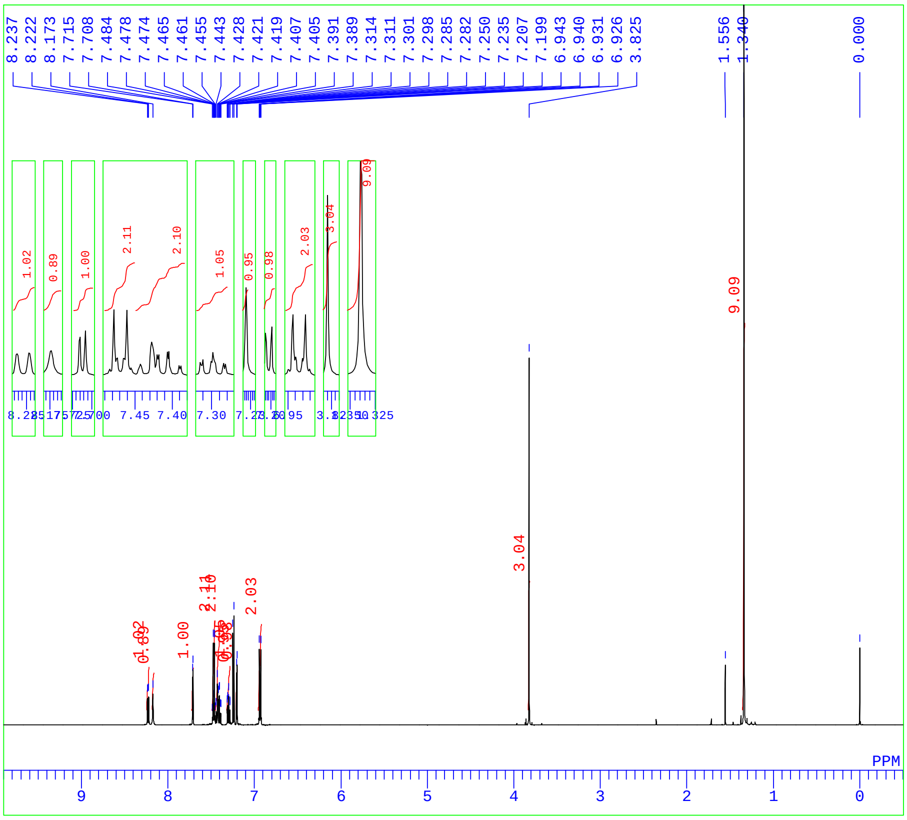

DFILE C-32 u H.als
COMNT single_pulse
DATIM 2017-01-27 21:07:15
OBNUC 1H
EXMOD single_pulse.ex2
OBFRQ 500.16 MHz
OBSET 2.41 KHz
OBFIN 6.01 Hz
POINT 13107
FREQU 7507.39 Hz
SCANS 8
ACQTM 1.7459 sec
PD 5.0000 sec
PW1 7.15 usec
IRNUC 1H
CTEMP 19.6 c
SLVNT CDCL3
EXREF 0.00 ppm
BF 0.12 Hz
RGAIN 44

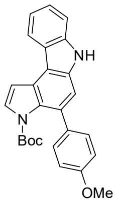

6gF

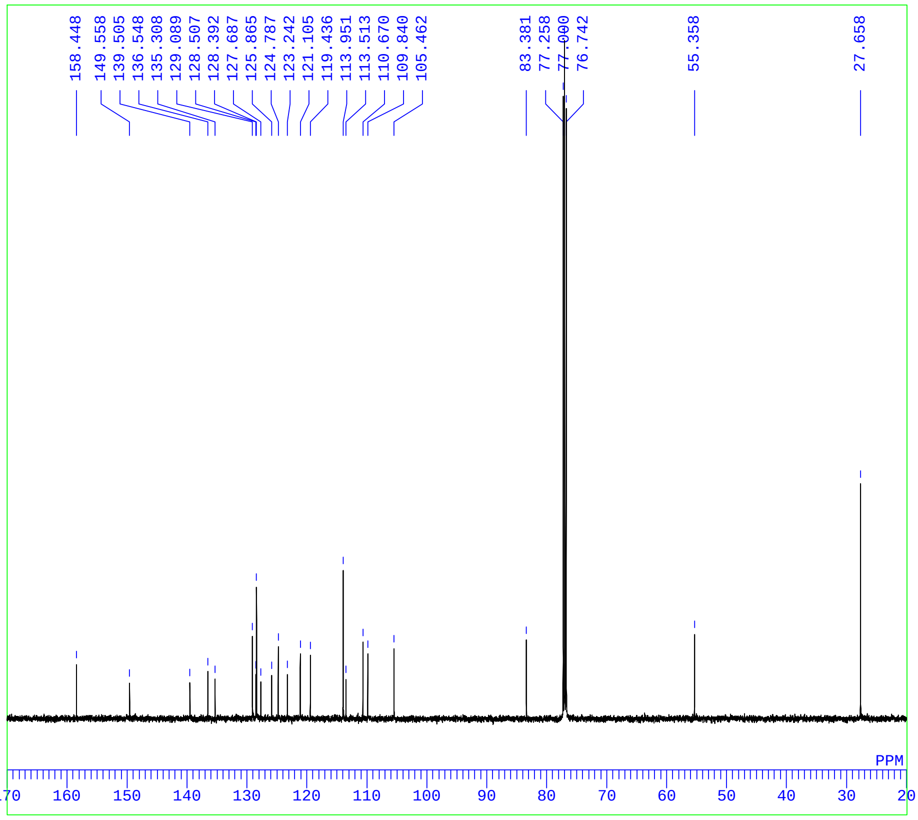

DFILE C-32 u C.als
COMNT
DATIM 2017-01-27 01:50:51
OBNUC 13C
EXMOD single_pulse_dec
OBFRQ 125.77 MHz
OBSET 7.87 KHz
OBFIN 4.21 Hz
POINT 26214
FREQU 31446.06 Hz
SCANS 1024
ACQTM 0.8336 sec
PD 2.0000 sec
PW1 3.67 usec
IRNUC 1H
CTEMP 19.6 c
SLVNT CDCL3
EXREF 77.00 ppm
BF 1.20 Hz
RGAIN 58

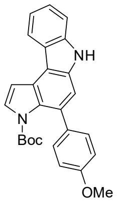

6gF

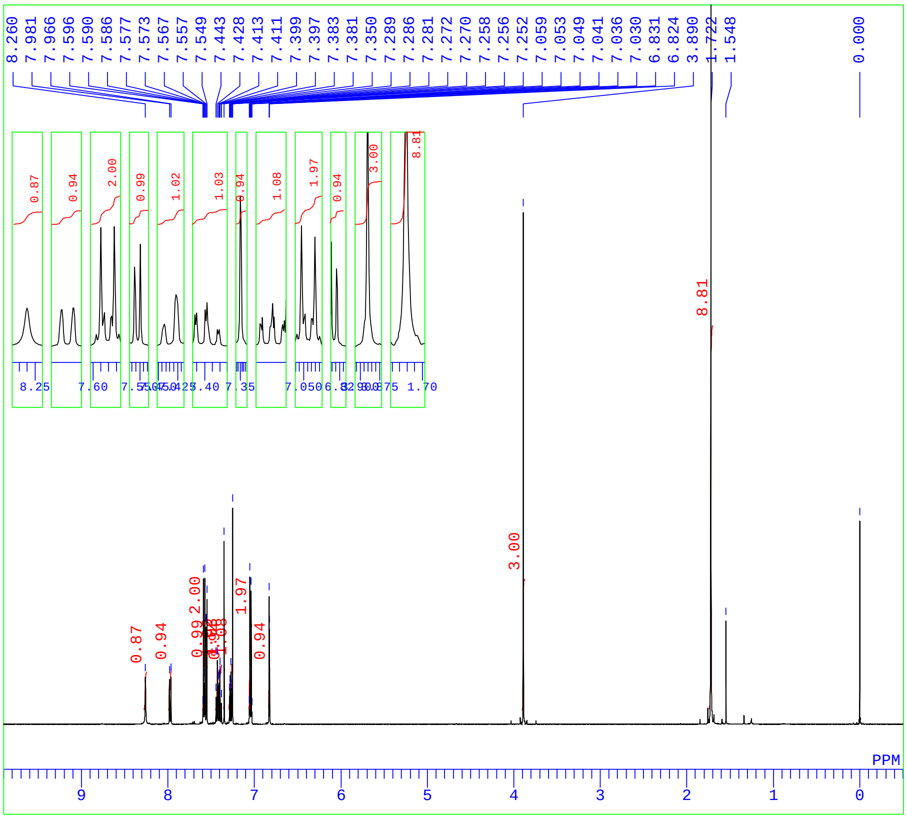

DFILE C-32 d H.als
COMNT single_pulse
DATIM 2017-02-08 13:18:54
OBNUC 1H
EXMOD single_pulse.ex2
OBFRQ 500.16 MHz
OBSET 2.41 KHz
OBFIN 6.01 Hz
POINT 13107
FREQU 7507.39 Hz
SCANS 8
ACQTM 1.7459 sec
PD 5.0000 sec
PW1 7.15 usec
IRNUC 1H
CTEMP 20.4 c
SLVNT CDCL3
EXREF 0.00 ppm
BF 0.12 Hz
RGAIN 46

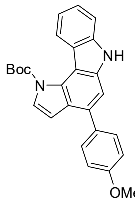

7gF

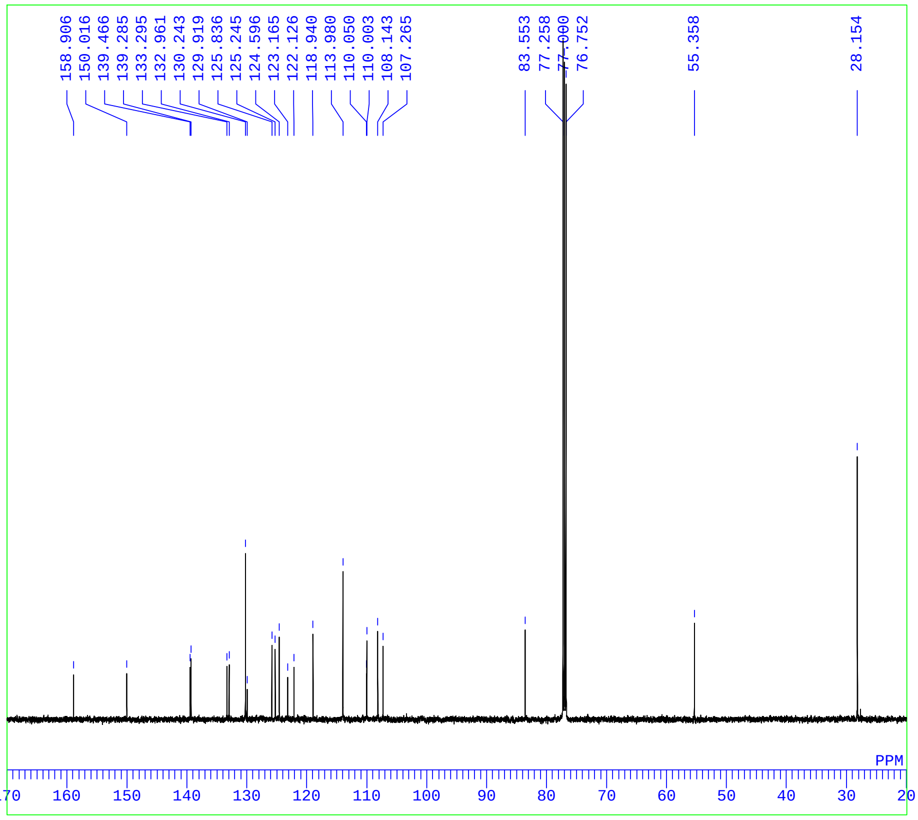

DFILE C-32 d C.als
COMNT
DATIM 2017-01-28 02:43:08
OBNUC 13C
EXMOD single_pulse_dec
OBFRQ 125.77 MHz
OBSET 7.87 KHz
OBFIN 4.21 Hz
POINT 26214
FREQU 31446.06 Hz
SCANS 1024
ACQTM 0.8336 sec
PD 2.0000 sec
PW1 3.67 usec
IRNUC 1H
CTEMP 20.9 c
SLVNT CDCL3
EXREF 77.00 ppm
BF 1.20 Hz
RGAIN 58

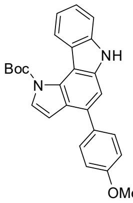

7gF

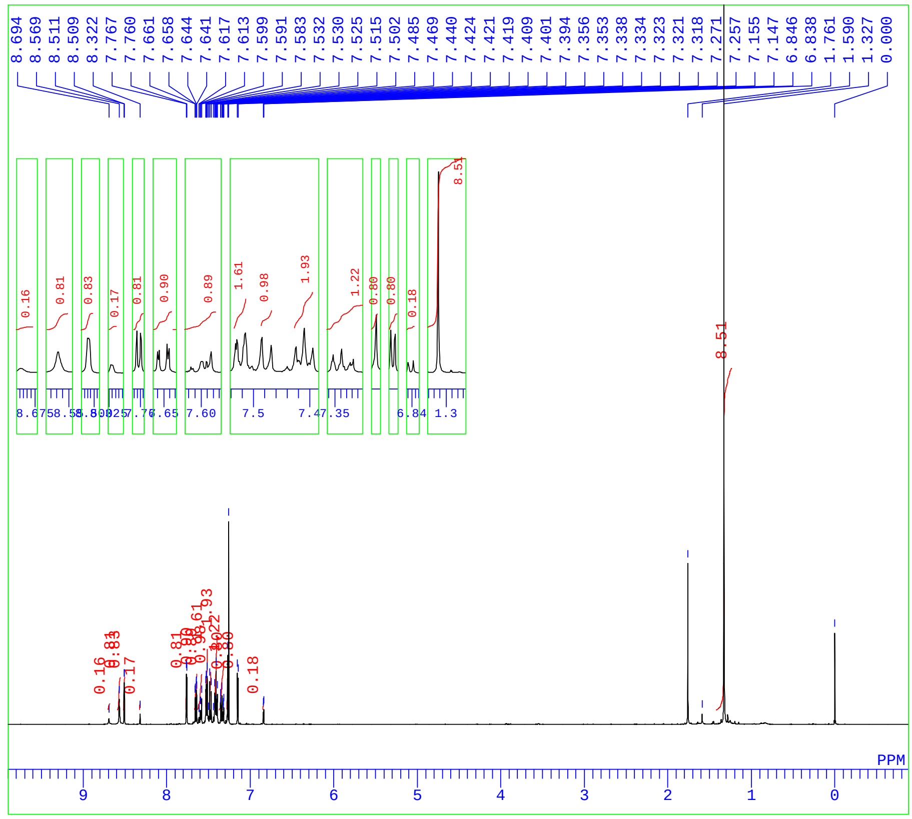

DFILE B-41 H.als
COMNT single_pulse
DATIM 2016-03-11 19:56:47
OBNUC 1H
EXMOD single_pulse.ex2
OBFRQ 500.16 MHz
OBSET 2.41 KHz
OBFIN 6.01 Hz
POINT 13107
FREQU 7507.39 Hz
SCANS 8
ACQTM 1.7459 sec
PD 5.0000 sec
PW1 6.82 usec
IRNUC 1H
CTEMP 20.2 c
SLVNT CDCL3
EXREF 0.00 ppm
BF 0.12 Hz
RGAIN 46

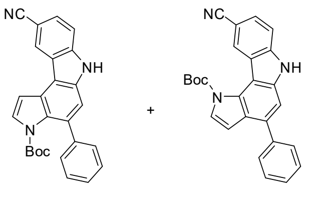

6hF **7hF**

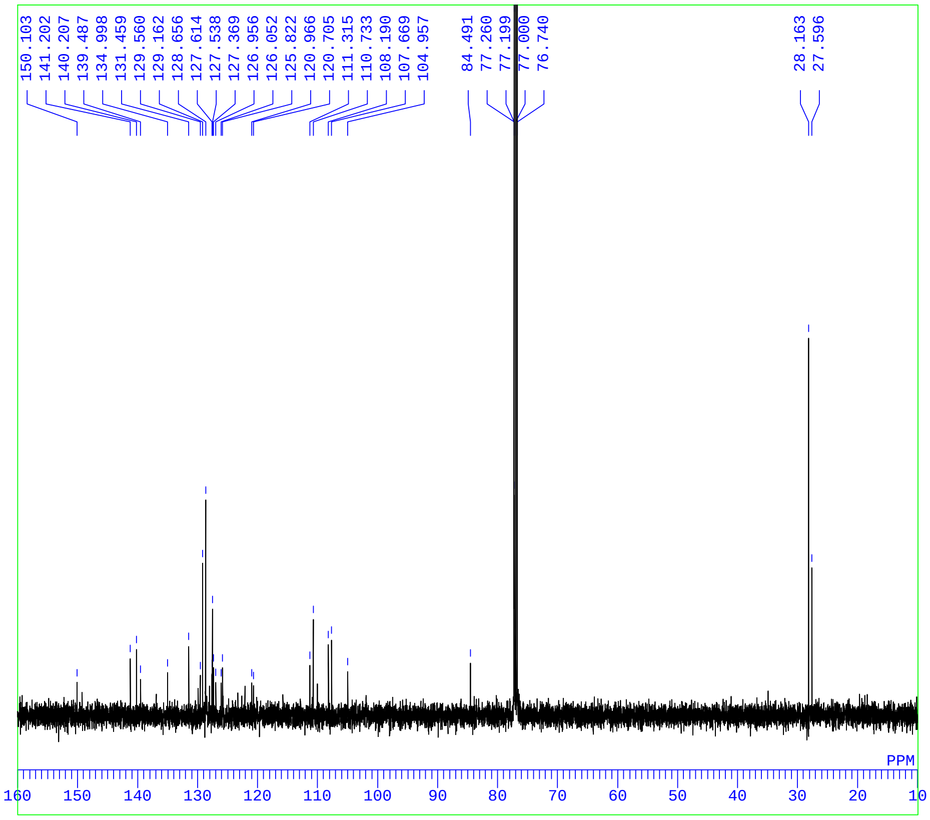

DFILE B41 C.als
COMNT
DATIM 2018-05-23 15:23:31
OBNUC 13C
EXMOD single_pulse_dec
OBFRQ 125.77 MHz
OBSET 7.87 KHz
OBFIN 4.21 Hz
POINT 16384
FREQU 31565.66 Hz
SCANS 1926
ACQTM 0.5190 sec
PD 1.0000 sec
PW1 3.67 usec
IRNUC 1H
CTEMP 21.7 c
SLVNT CDCL3
EXREF 77.00 ppm
BF 0.12 Hz
RGAIN 60

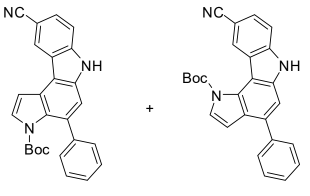

6hF **7hF**

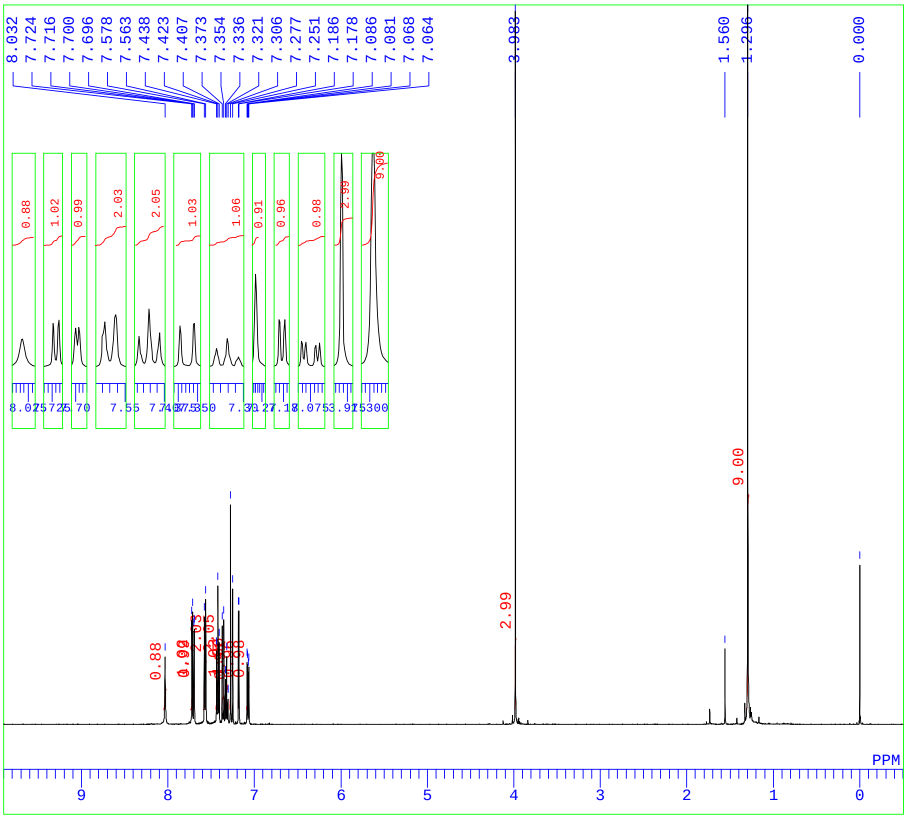

DFILE C-42 u H.als
COMNT single_pulse
DATIM 2017-01-27 21:27:46
OBNUC 1H
EXMOD single_pulse.ex2
OBFRQ 500.16 MHz
OBSET 2.41 KHz
OBFIN 6.01 Hz
POINT 13107
FREQU 7507.39 Hz
SCANS 8
ACQTM 1.7459 sec
PD 5.0000 sec
PW1 7.15 usec
IRNUC 1H
CTEMP 19.6 c
SLVNT CDCL3
EXREF 0.00 ppm
BF 0.12 Hz
RGAIN 44

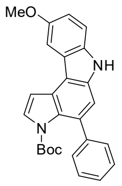

6iF

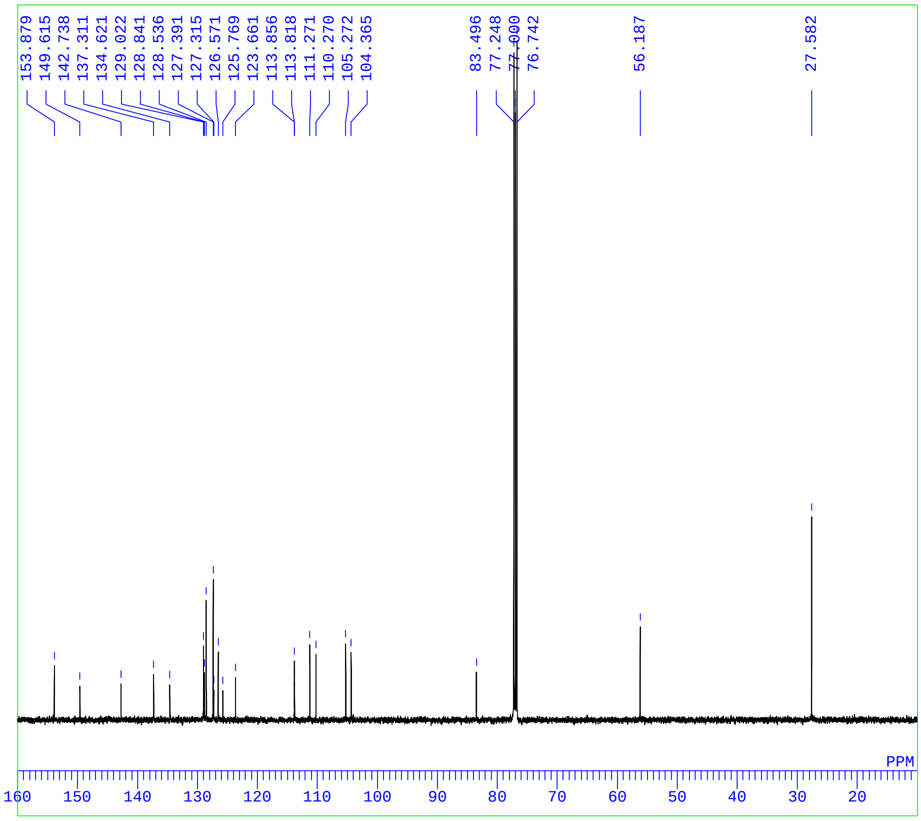

DFILE C-42 u C.als
COMNT
DATIM 2017-01-28 08:11:48
OBNUC 13C
EXMOD single_pulse_dec
OBFRQ 125.77 MHz
OBSET 7.87 KHz
OBFIN 4.21 Hz
POINT 26214
FREQU 31446.06 Hz
SCANS 1024
ACQTM 0.8336 sec
PD 2.0000 sec
PW1 3.67 usec
IRNUC 1H
CTEMP 20.9 c
SLVNT CDCL3
EXREF 77.00 ppm
BF 1.20 Hz
RGAIN 60

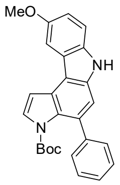

6iF

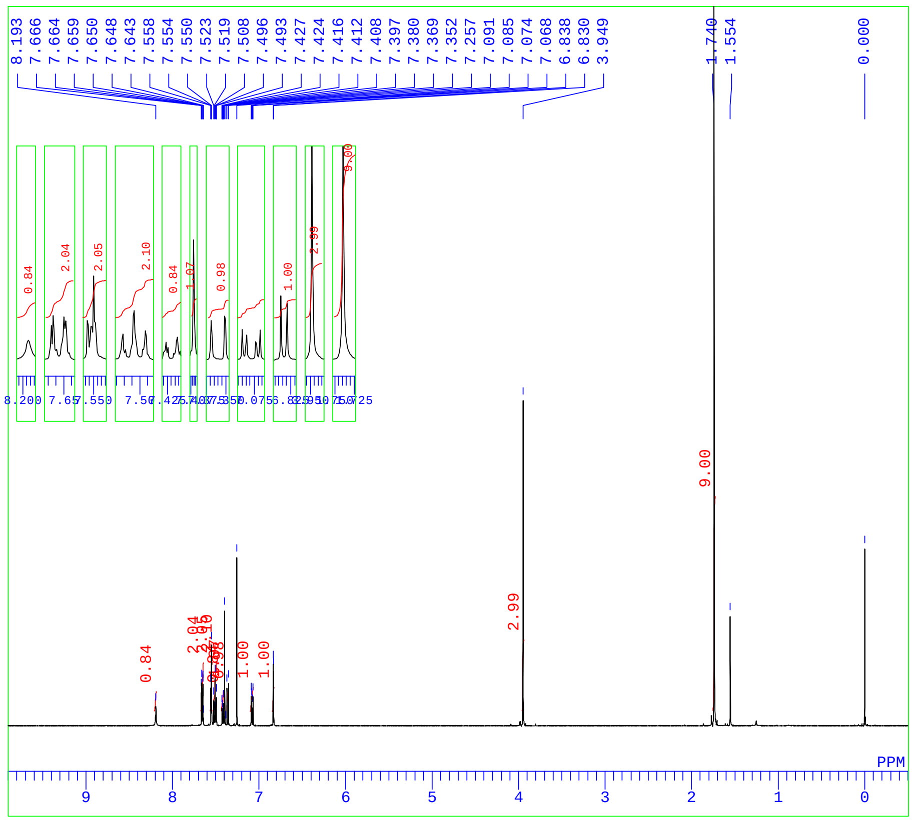

DFILE C-42 d H.als
COMNT single_pulse
DATIM 2017-02-08 13:30:24
OBNUC 1H
EXMOD single_pulse.ex2
OBFRQ 500.16 MHz
OBSET 2.41 KHz
OBFIN 6.01 Hz
POINT 13107
FREQU 7507.39 Hz
SCANS 8
ACQTM 1.7459 sec
PD 5.0000 sec
PW1 7.15 usec
IRNUC 1H
CTEMP 20.4 c
SLVNT CDCL3
EXREF 0.00 ppm
BF 0.12 Hz
RGAIN 50

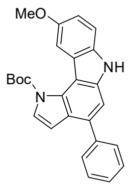

7iF

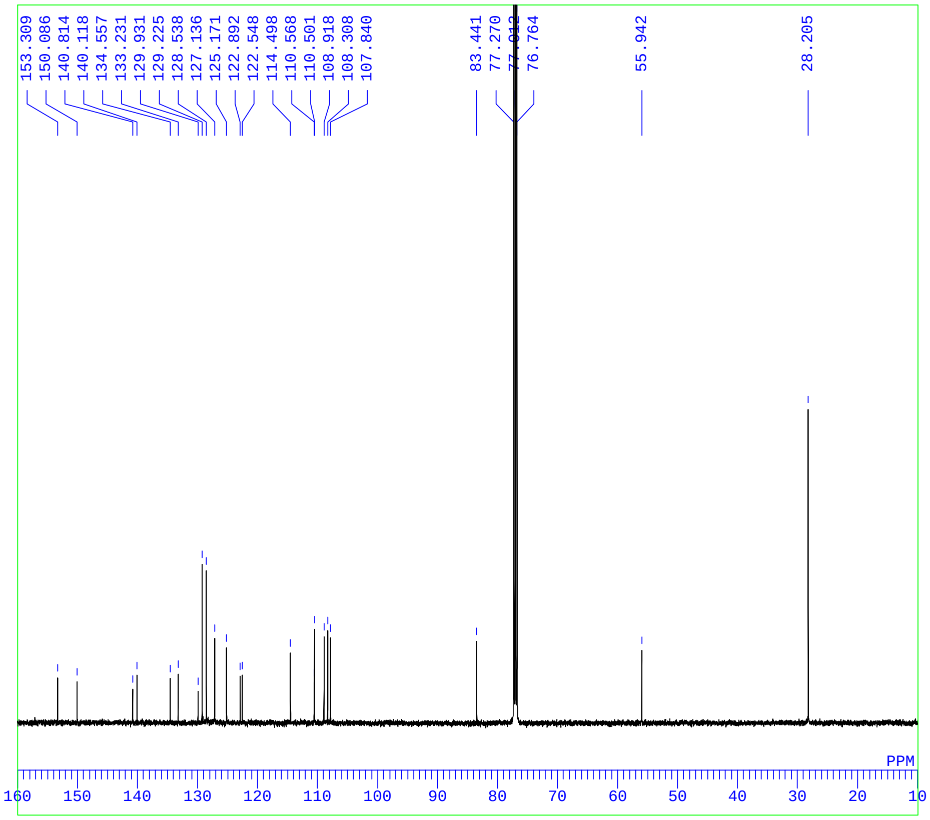

DFILE C-42 d C.als
COMNT
DATIM 2017-01-27 07:50:26
OBNUC 13C
EXMOD single_pulse_dec
OBFRQ 125.77 MHz
OBSET 7.87 KHz
OBFIN 4.21 Hz
POINT 26214
FREQU 31446.06 Hz
SCANS 5120
ACQTM 0.8336 sec
PD 2.0000 sec
PW1 3.67 usec
IRNUC 1H
CTEMP 19.6 c
SLVNT CDCL3
EXREF 0.00 ppm
BF 1.20 Hz
RGAIN 58

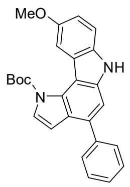

7iF

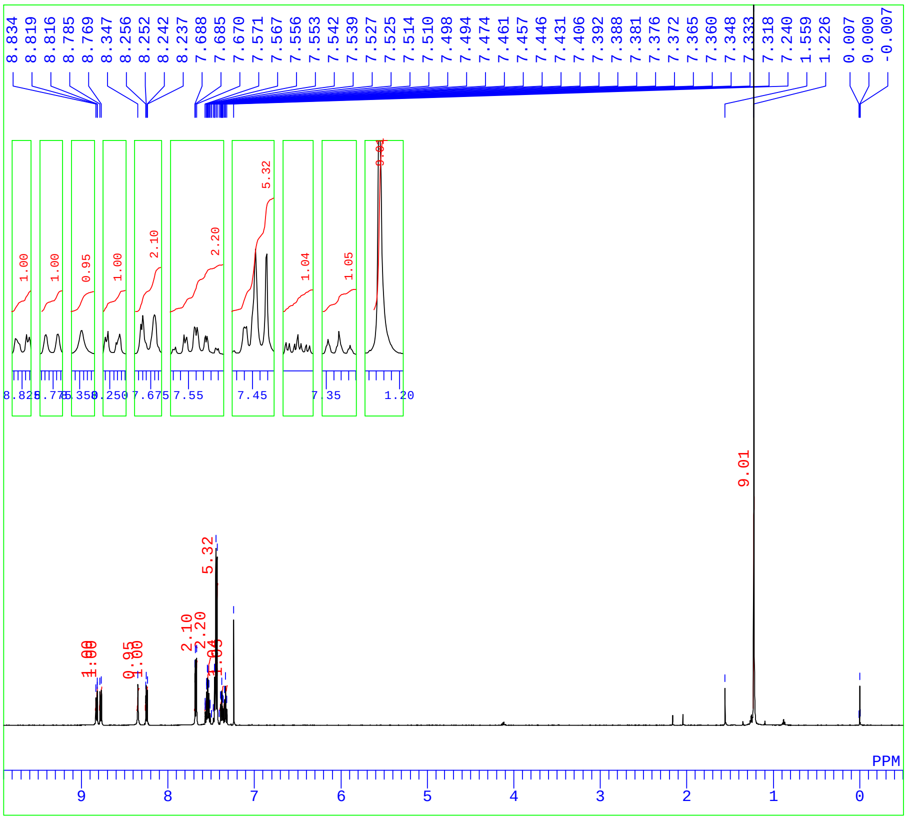

DFILE D-17 H.als
COMNT single_pulse
DATIM 2018-03-07 13:02:42
OBNUC 1H
EXMOD single_pulse.ex2
OBFRQ 500.16 MHz
OBSET 2.41 KHz
OBFIN 6.01 Hz
POINT 13107
FREQU 7507.39 Hz
SCANS 8
ACQTM 1.7459 sec
PD 5.0000 sec
PW1 7.15 usec
IRNUC 1H
CTEMP 19.5 c
SLVNT CDCL3
EXREF 0.00 ppm
BF 0.12 Hz
RGAIN 42

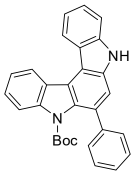

11A

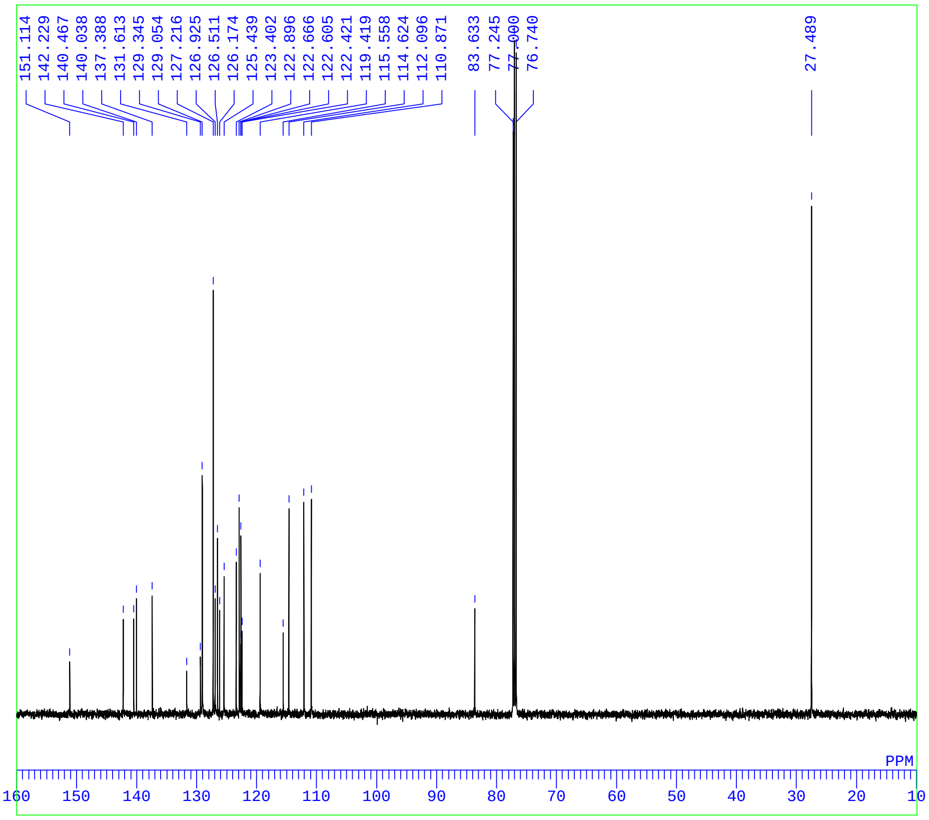

DFILE D-17 C-1.als
COMNT
DATIM 2018-03-08 08:59:34
OBNUC 13C
EXMOD single_pulse_dec
OBFRQ 125.77 MHz
OBSET 7.87 KHz
OBFIN 4.21 Hz
POINT 13107
FREQU 25252.14 Hz
SCANS 1413
ACQTM 0.5190 sec
PD 1.0000 sec
PW1 3.67 usec
IRNUC 1H
CTEMP 19.5 c
SLVNT CDCL3
EXREF 77.00 ppm
BF 1.20 Hz
RGAIN 60

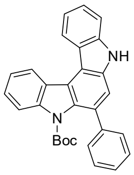

11A

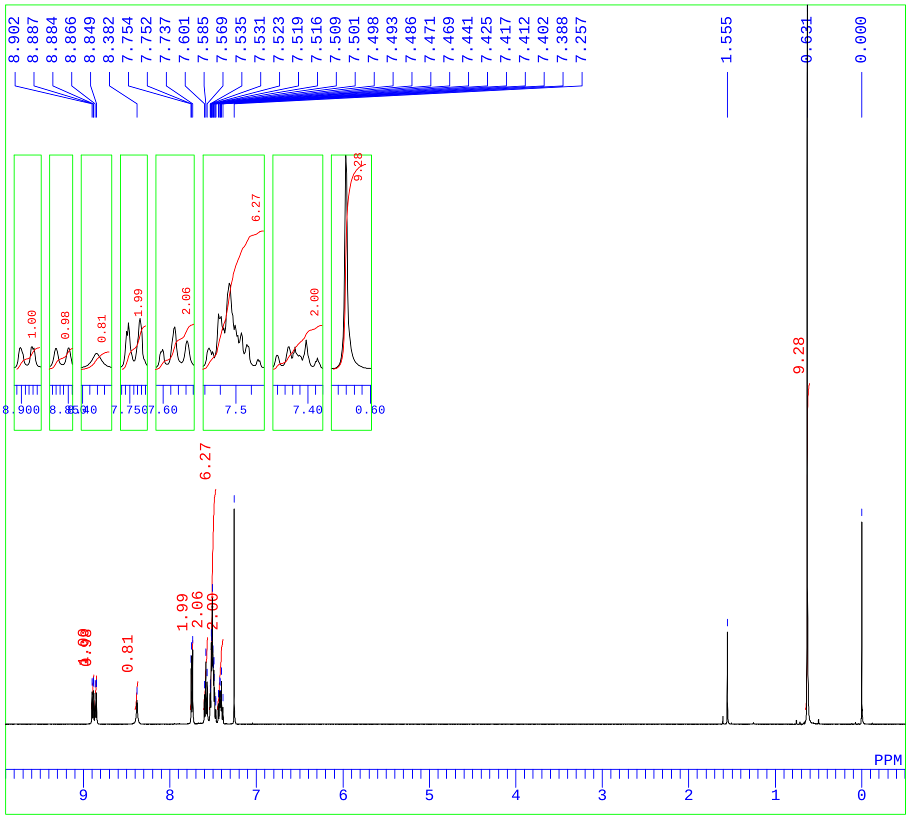

DFILE C-126 H-1.als
COMNT single_pulse
DATIM 2018-03-07 22:40:02
OBNUC 1H
EXMOD single_pulse.ex2
OBFRQ 500.16 MHz
OBSET 2.41 KHz
OBFIN 6.01 Hz
POINT 13107
FREQU 7507.39 Hz
SCANS 8
ACQTM 1.7459 sec
PD 5.0000 sec
PW1 7.15 usec
IRNUC 1H
CTEMP 19.5 c
SLVNT CDCL3
EXREF 0.00 ppm
BF 0.12 Hz
RGAIN 50

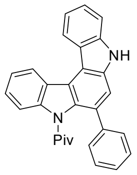

11B

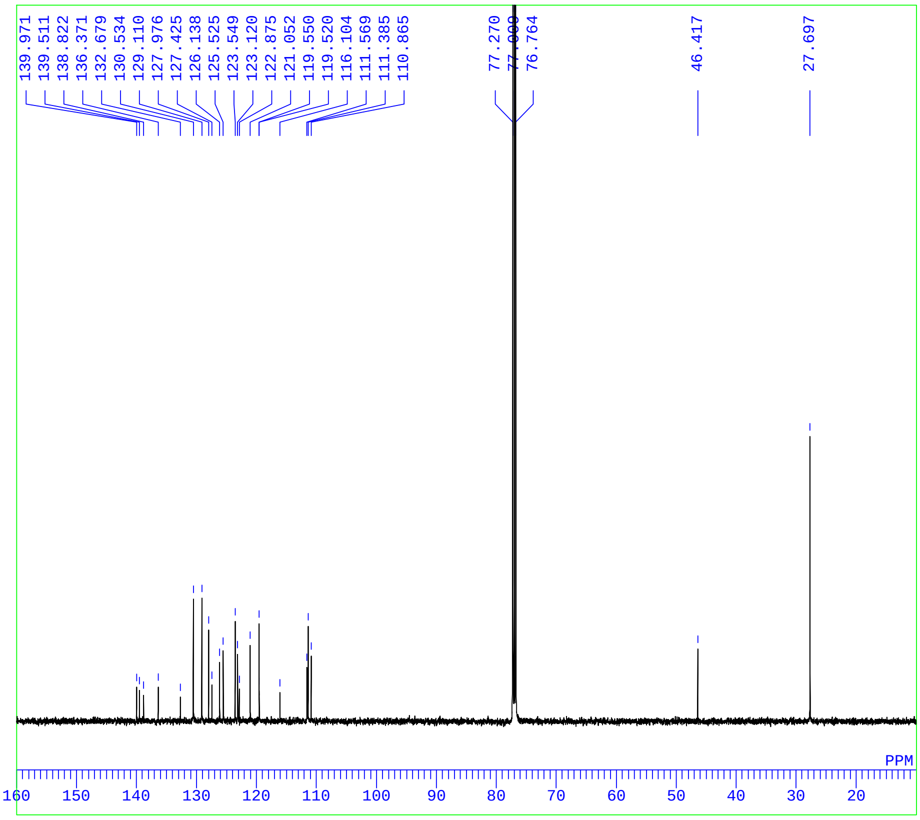

DFILE C-126 C-1.als
COMNT
DATIM 2018-03-08 07:14:32
OBNUC 13C
EXMOD single_pulse_dec
OBFRQ 125.77 MHz
OBSET 7.87 KHz
OBFIN 4.21 Hz
POINT 13107
FREQU 25252.14 Hz
SCANS 7168
ACQTM 0.5190 sec
PD 1.0000 sec
PW1 3.67 usec
IRNUC 1H
CTEMP 19.5 c
SLVNT CDCL3
EXREF 0.00 ppm
BF 1.20 Hz
RGAIN 60

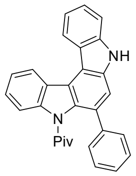

11B

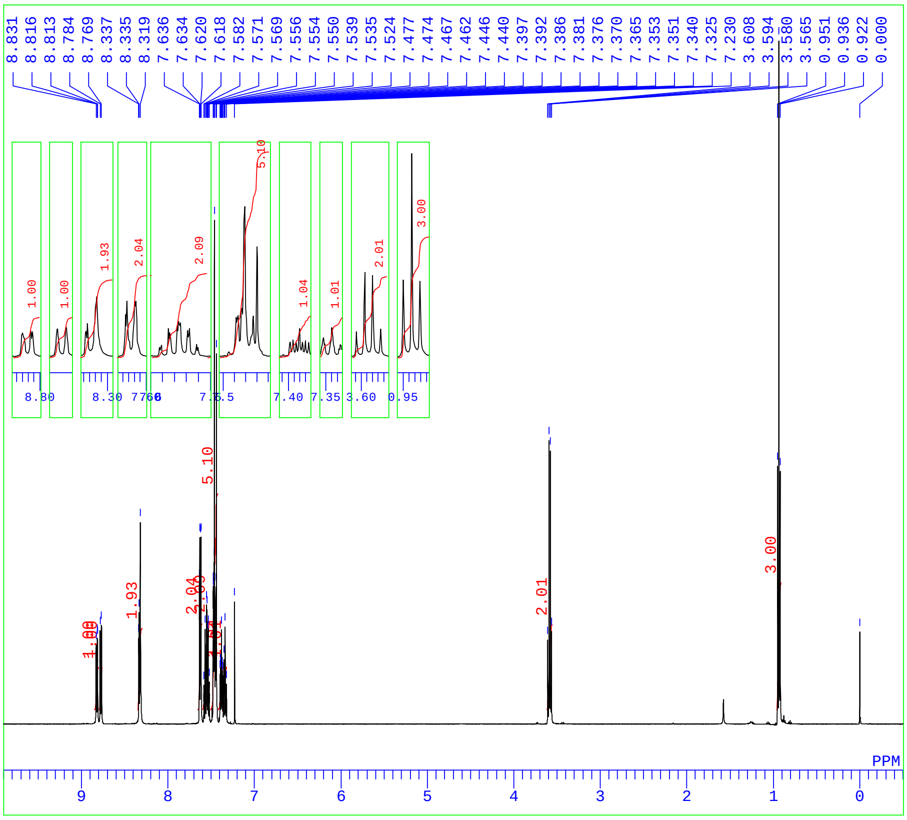

DFILE B-154 H-1.als
COMNT single_pulse
DATIM 2018-03-06 18:20:48
OBNUC 1H
EXMOD single_pulse.ex2
OBFRQ 500.16 MHz
OBSET 2.41 KHz
OBFIN 6.01 Hz
POINT 13107
FREQU 7507.39 Hz
SCANS 8
ACQTM 1.7459 sec
PD 5.0000 sec
PW1 7.15 usec
IRNUC 1H
CTEMP 19.4 c
SLVNT CDCL3
EXREF 0.00 ppm
BF 0.12 Hz
RGAIN 40

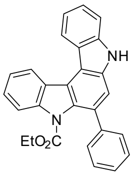

11C

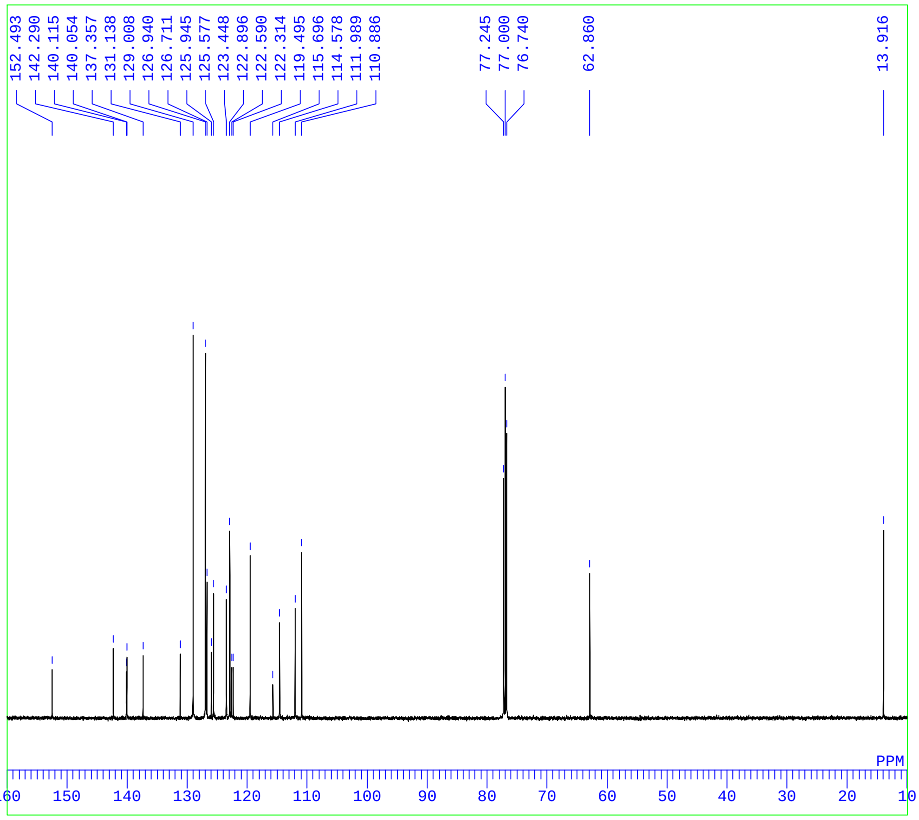

DFILE B-154 C-1.als
COMNT
DATIM 2018-03-07 23:55:47
OBNUC 13C
EXMOD single_pulse_dec
OBFRQ 125.77 MHz
OBSET 7.87 KHz
OBFIN 4.21 Hz
POINT 13107
FREQU 25252.14 Hz
SCANS 2048
ACQTM 0.5190 sec
PD 1.0000 sec
PW1 3.67 usec
IRNUC 1H
CTEMP 19.8 c
SLVNT CDCL3
EXREF 77.00 ppm
BF 1.20 Hz
RGAIN 60

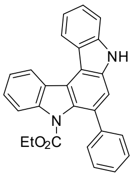

11C

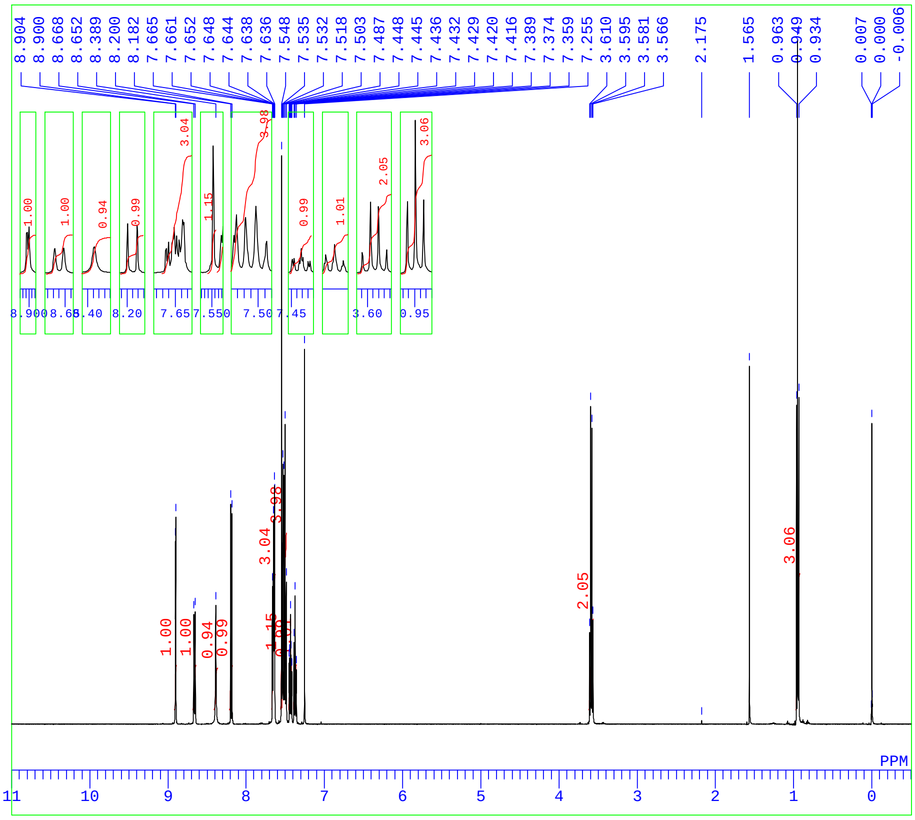

DFILE C-23 H-1.als
COMNT single_pulse
DATIM 2018-03-06 18:28:52
OBNUC 1H
EXMOD single_pulse.ex2
OBFRQ 500.16 MHz
OBSET 2.41 KHz
OBFIN 6.01 Hz
POINT 13107
FREQU 7507.39 Hz
SCANS 8
ACQTM 1.7459 sec
PD 5.0000 sec
PW1 7.15 usec
IRNUC 1H
CTEMP 19.5 c
SLVNT CDCL3
EXREF 0.00 ppm
BF 0.12 Hz
RGAIN 48

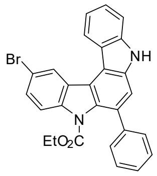

11D

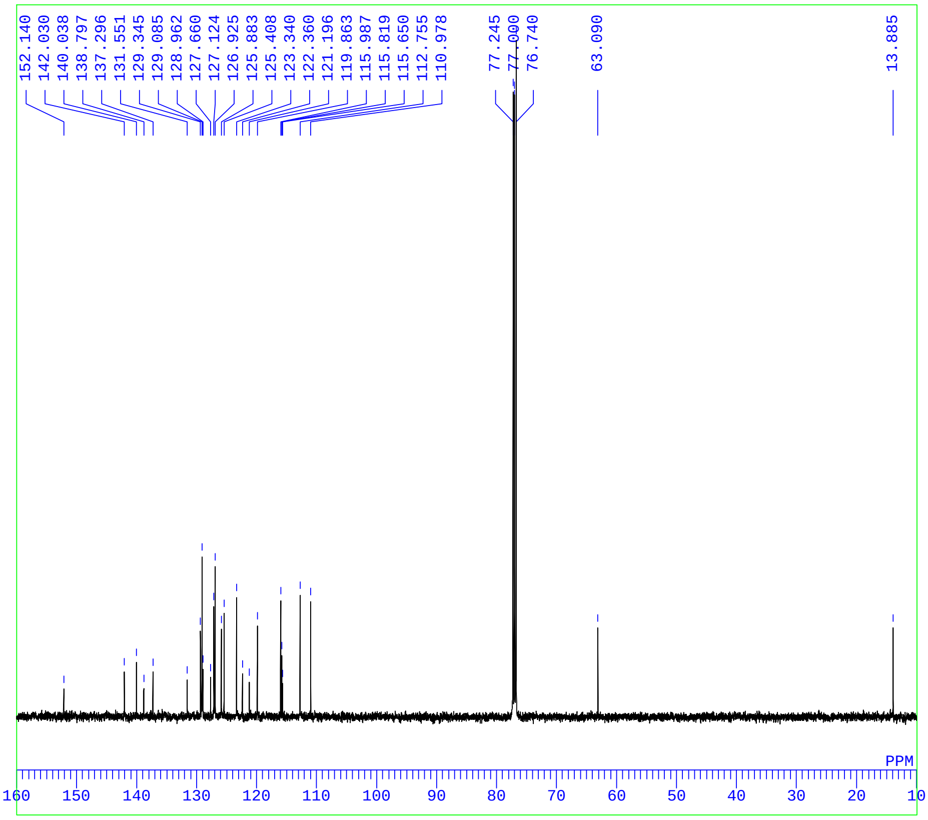

DFILE C-23 C-1.als
COMNT
DATIM 2018-03-08 02:57:26
OBNUC 13C
EXMOD single_pulse_dec
OBFRQ 125.77 MHz
OBSET 7.87 KHz
OBFIN 4.21 Hz
POINT 13107
FREQU 25252.14 Hz
SCANS 2048
ACQTM 0.5190 sec
PD 1.0000 sec
PW1 3.67 usec
IRNUC 1H
CTEMP 19.6 c
SLVNT CDCL3
EXREF 77.00 ppm
BF 1.20 Hz
RGAIN 60

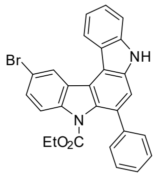

11D

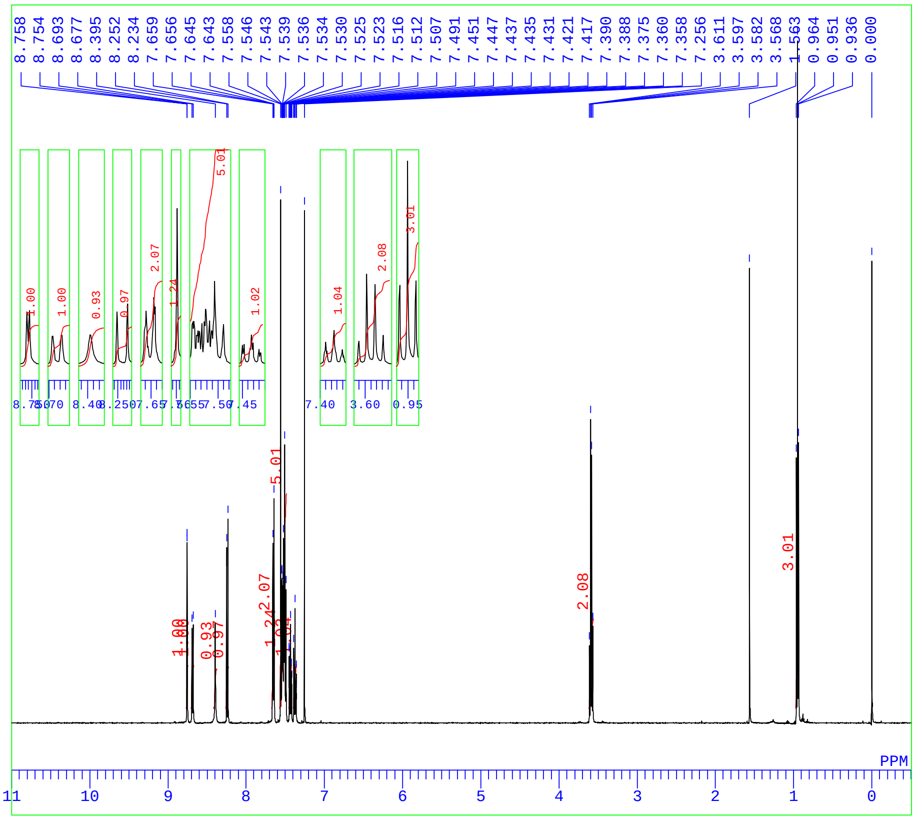

DFILE C-24 H-1.als
COMNT single_pulse
DATIM 2018-03-06 18:32:48
OBNUC 1H
EXMOD single_pulse.ex2
OBFRQ 500.16 MHz
OBSET 2.41 KHz
OBFIN 6.01 Hz
POINT 13107
FREQU 7507.39 Hz
SCANS 8
ACQTM 1.7459 sec
PD 5.0000 sec
PW1 7.15 usec
IRNUC 1H
CTEMP 19.6 c
SLVNT CDCL3
EXREF 0.00 ppm
BF 0.12 Hz
RGAIN 48

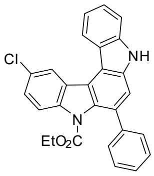

11E

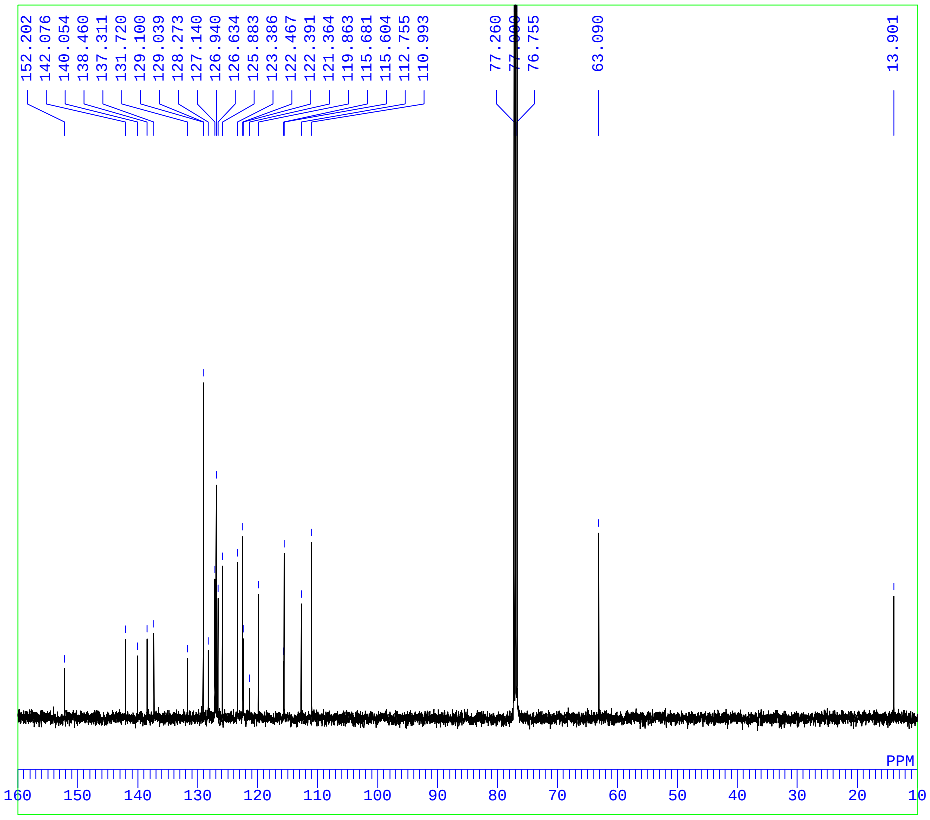

DFILE C-24 C-1.als
COMNT
DATIM 2018-03-08 04:06:05
OBNUC 13C
EXMOD single_pulse_dec
OBFRQ 125.77 MHz
OBSET 7.87 KHz
OBFIN 4.21 Hz
POINT 13107
FREQU 25252.14 Hz
SCANS 2048
ACQTM 0.5190 sec
PD 1.0000 sec
PW1 3.67 usec
IRNUC 1H
CTEMP 19.5 c
SLVNT CDCL3
EXREF 77.00 ppm
BF 1.20 Hz
RGAIN 60

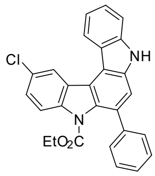

11E

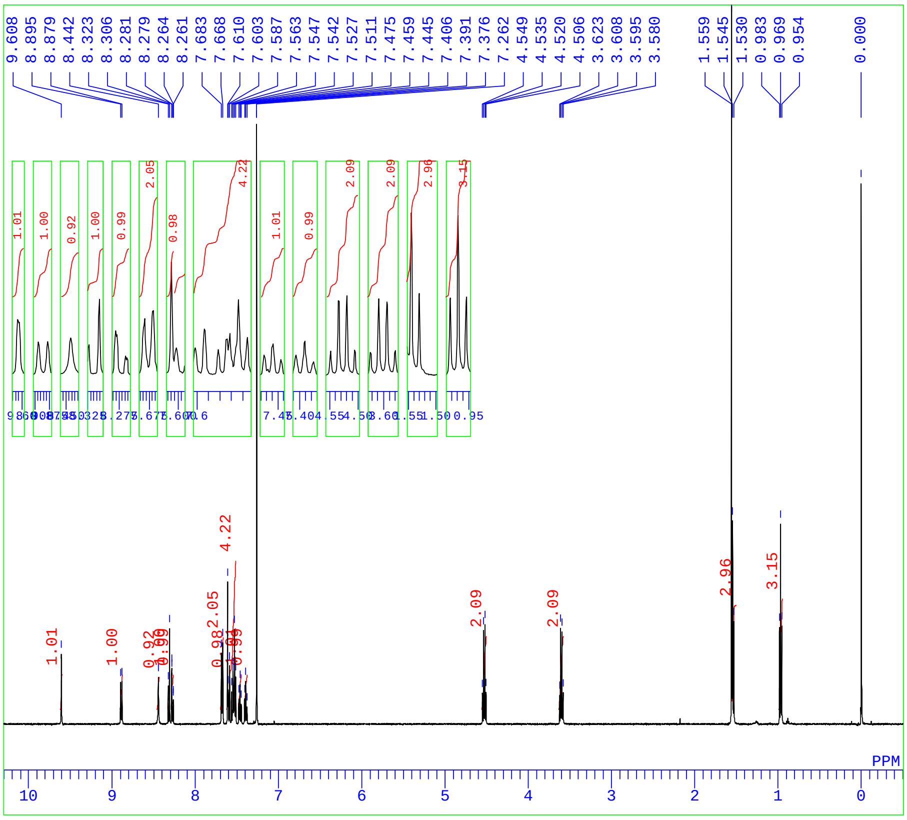

DFILE	B-194 H-1.als
COMNT	single_pulse
DATIM	2018-03-06 18:24:42
OBNUC	1H
EXMOD	single_pulse.ex2
OBFRQ	500.16 MHz
OBSET	2.41 KHz
OBFIN	6.01 Hz
POINT	13107
FREQU	7507.39 Hz
SCANS	8
ACQTM	1.7459 sec
PD	5.0000 sec
PW1	7.15 usec
IRNUC	1H
CTEMP	19.5 c
SLVNT	CDCL3
EXREF	0.00 ppm
BF	0.12 Hz
RGAIN	52

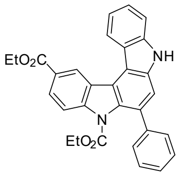

11F

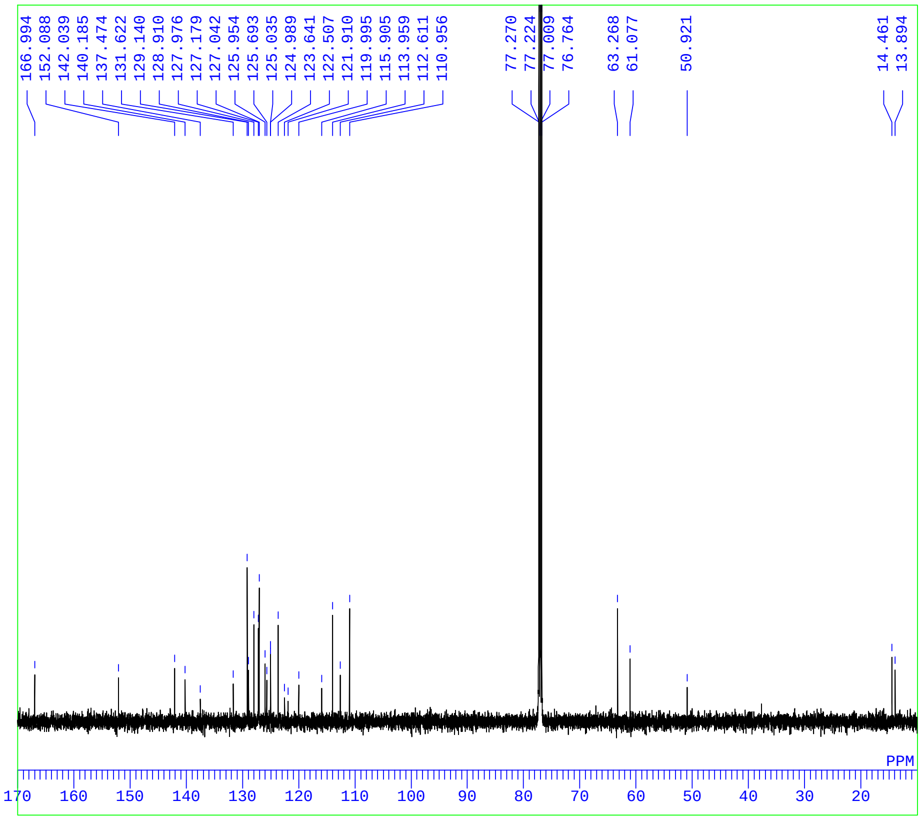

DFILE	A194 C.als
COMNT	
DATIM	2018-04-29 13:25:59
OBNUC	13C
EXMOD	single_pulse_dec
OBFRQ	125.77 MHz
OBSET	7.87 KHz
OBFIN	4.21 Hz
POINT	16384
FREQU	31565.66 Hz
SCANS	18969
ACQTM	0.5190 sec
PD	1.0000 sec
PW1	3.67 usec
IRNUC	1H
CTEMP	21.6 c
SLVNT	CDCL3
EXREF	0.00 ppm
BF	0.12 Hz
RGAIN	60

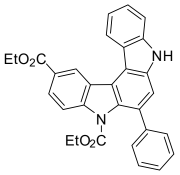

11F

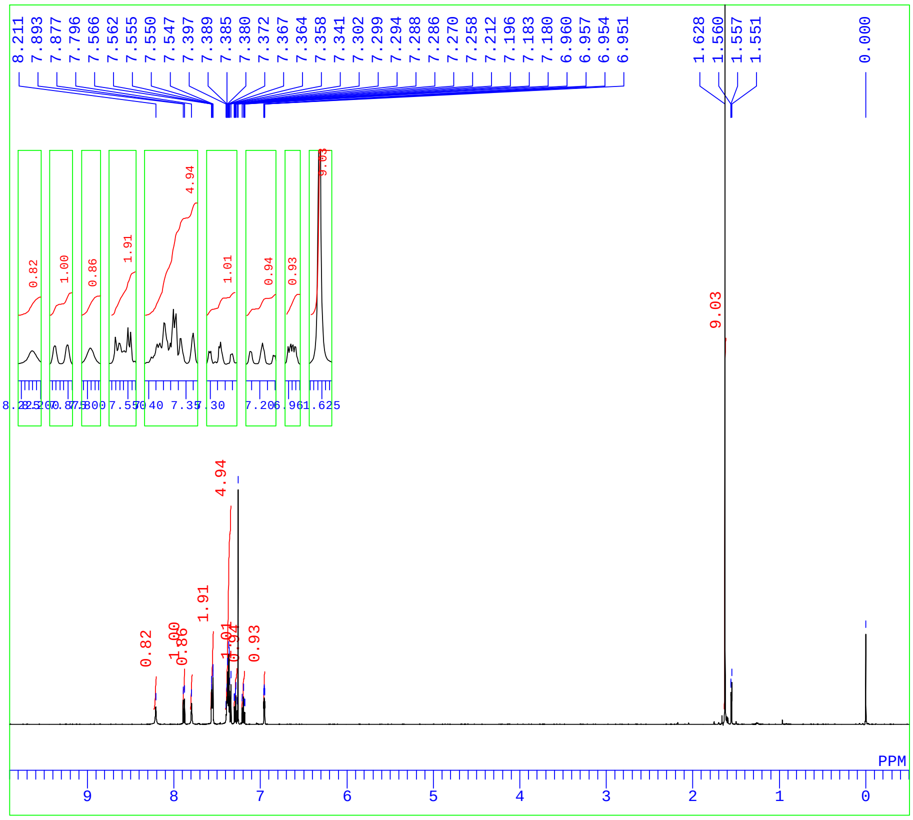

DFILE 4aF H-1.als
COMNT single_pulse
DATIM 2017-02-16 15:06:51
OBNUC 1H
EXMOD single_pulse.ex2
OBFRQ 500.16 MHz
OBSET 2.41 KHz
OBFIN 6.01 Hz
POINT 13107
FREQU 7507.39 Hz
SCANS 8
ACQTM 1.7459 sec
PD 5.0000 sec
PW1 7.15 usec
IRNUC 1H
CTEMP 20.0 c
SLVNT CDCL3
EXREF 0.00 ppm
BF 0.12 Hz
RGAIN 50

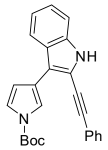

4aF

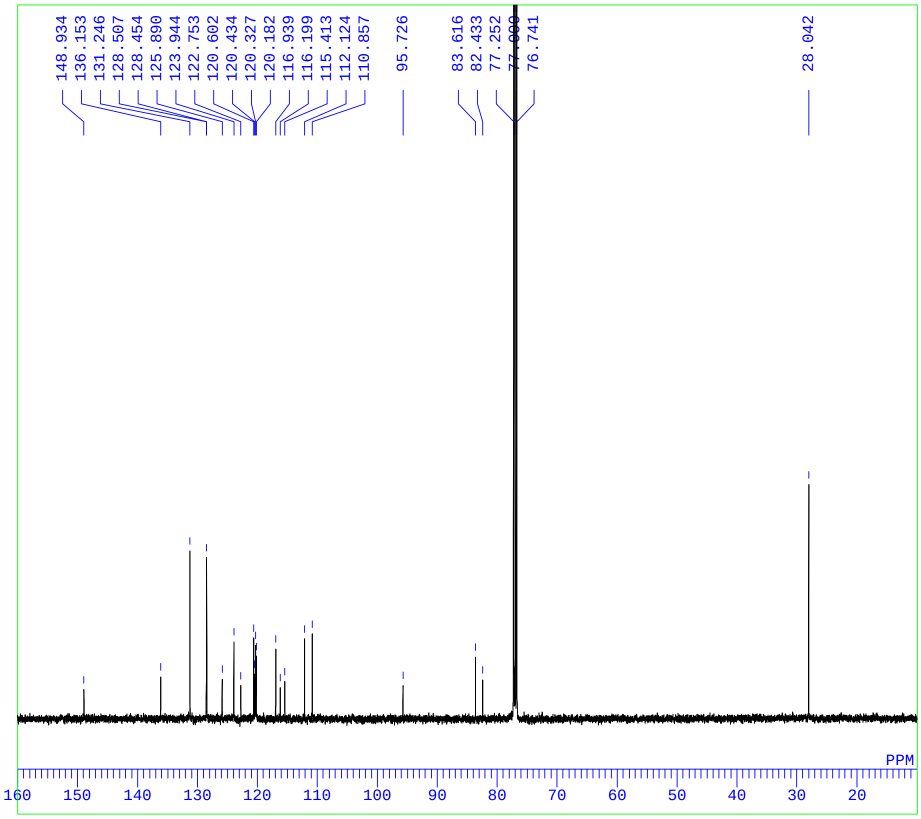

DFILE 4aF C-1.als
COMNT
DATIM 2016-11-10 15:50:03
OBNUC 13C
EXMOD single_pulse_dec
OBFRQ 125.77 MHz
OBSET 7.87 KHz
OBFIN 4.21 Hz
POINT 32768
FREQU 31446.54 Hz
SCANS 1024
ACQTM 1.0420 sec
PD 2.0000 sec
PW1 3.67 usec
IRNUC 1H
CTEMP 22.2 c
SLVNT CDCL3
EXREF 77.00 ppm
BF 1.20 Hz
RGAIN 70

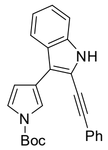

4aF

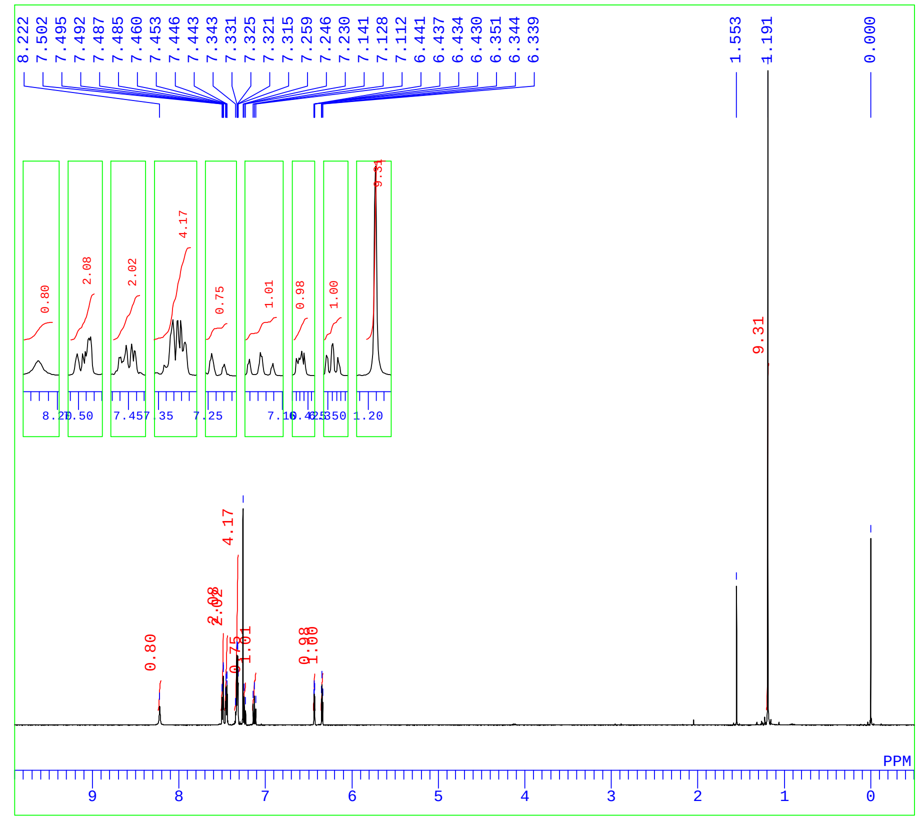

DFILE	5aF H-1.als
COMNT	single_pulse
DATIM	2017-02-16 15:10:30
OBNUC	1H
EXMOD	single_pulse.ex2
OBFRQ	500.16 MHz
OBSET	2.41 KHz
OBFIN	6.01 Hz
POINT	13107
FREQU	7507.39 Hz
SCANS	8
ACQTM	1.7459 sec
PD	5.0000 sec
PW1	7.15 usec
IRNUC	1H
CTEMP	20.2 c
SLVNT	CDCL3
EXREF	0.00 ppm
BF	0.12 Hz
RGAIN	50

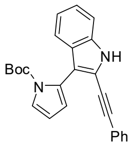

5aF

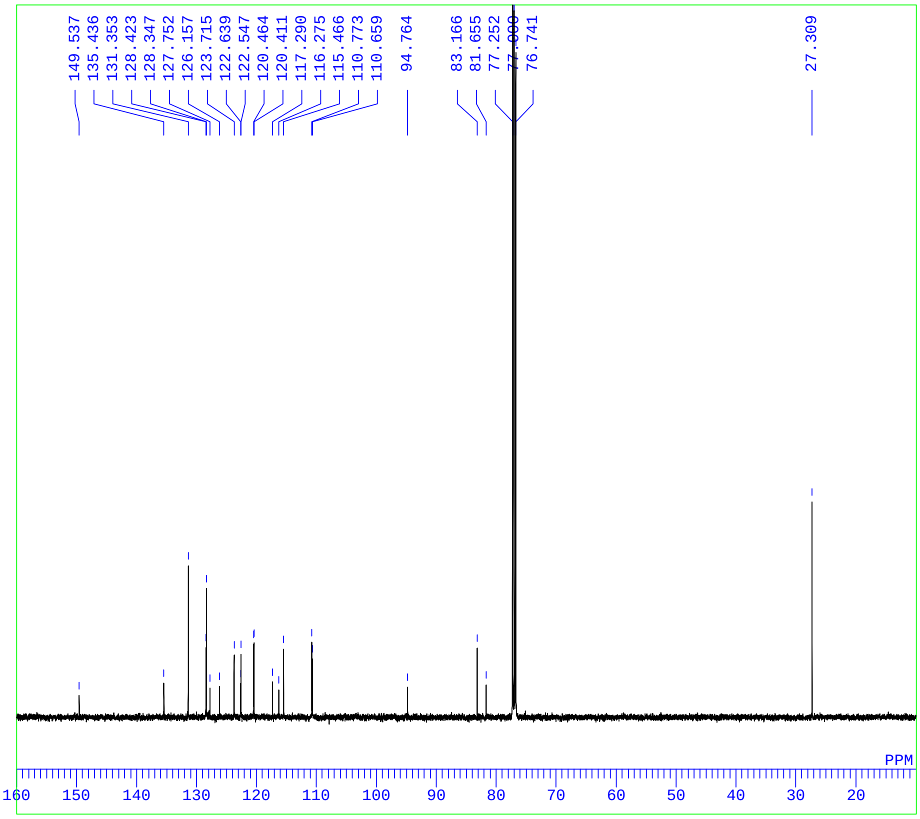

DFILE	5aF C-1.als
COMNT	
DATIM	2016-11-10 19:17:52
OBNUC	13C
EXMOD	single_pulse_dec
OBFRQ	125.77 MHz
OBSET	7.87 KHz
OBFIN	4.21 Hz
POINT	32768
FREQU	31446.54 Hz
SCANS	1024
ACQTM	1.0420 sec
PD	2.0000 sec
PW1	3.67 usec
IRNUC	1H
CTEMP	22.1 c
SLVNT	CDCL3
EXREF	77.00 ppm
BF	1.20 Hz
RGAIN	70

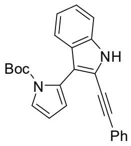

5aF

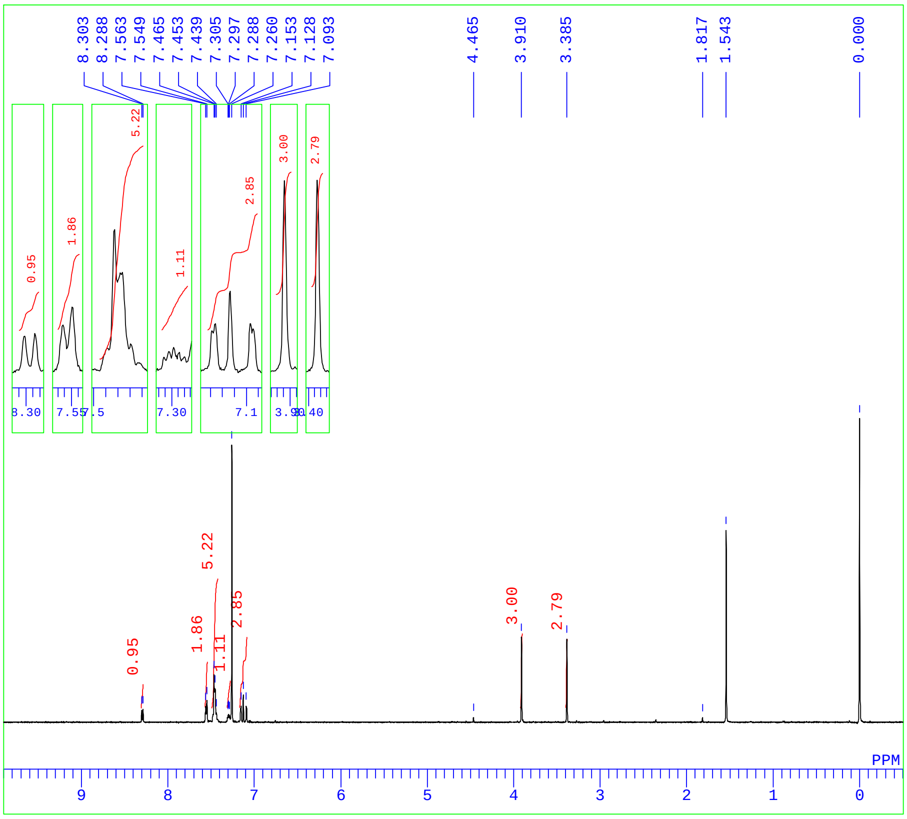

DFILE B-58 u H.als
COMNT single_pulse
DATIM 2016-04-27 14:43:56
OBNUC 1H
EXMOD single_pulse.ex2
OBFRQ 500.16 MHz
OBSET 2.41 KHz
OBFIN 6.01 Hz
POINT 13107
FREQU 7507.39 Hz
SCANS 8
ACQTM 1.7459 sec
PD 5.0000 sec
PW1 6.82 usec
IRNUC 1H
CTEMP 21.7 c
SLVNT CDCL3
EXREF 0.00 ppm
BF 0.12 Hz
RGAIN 56

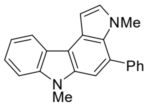

6aF-Me$_2$

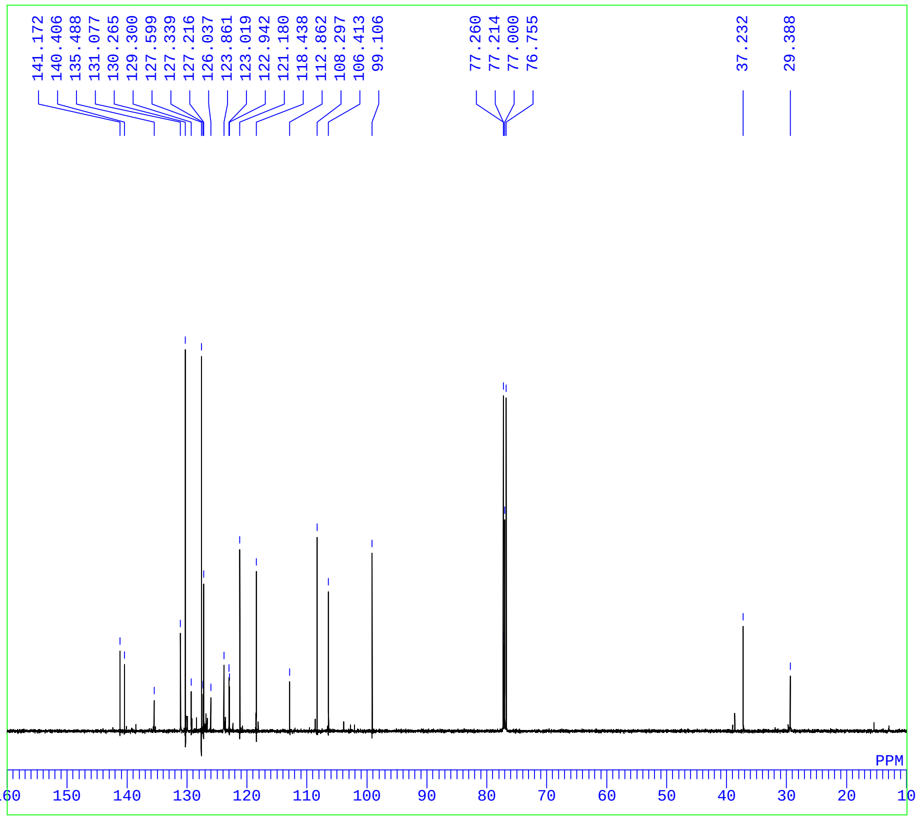

DFILE B58 up C.als
COMNT
DATIM 2018-04-29 16:54:31
OBNUC 13C
EXMOD single_pulse_dec
OBFRQ 125.77 MHz
OBSET 7.87 KHz
OBFIN 4.21 Hz
POINT 13107
FREQU 25252.14 Hz
SCANS 4218
ACQTM 0.5190 sec
PD 1.0000 sec
PW1 3.67 usec
IRNUC 1H
CTEMP 21.5 c
SLVNT CDCL3
EXREF 77.00 ppm
BF 0.12 Hz
RGAIN 60

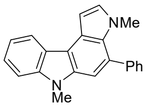

6aF-Me$_2$

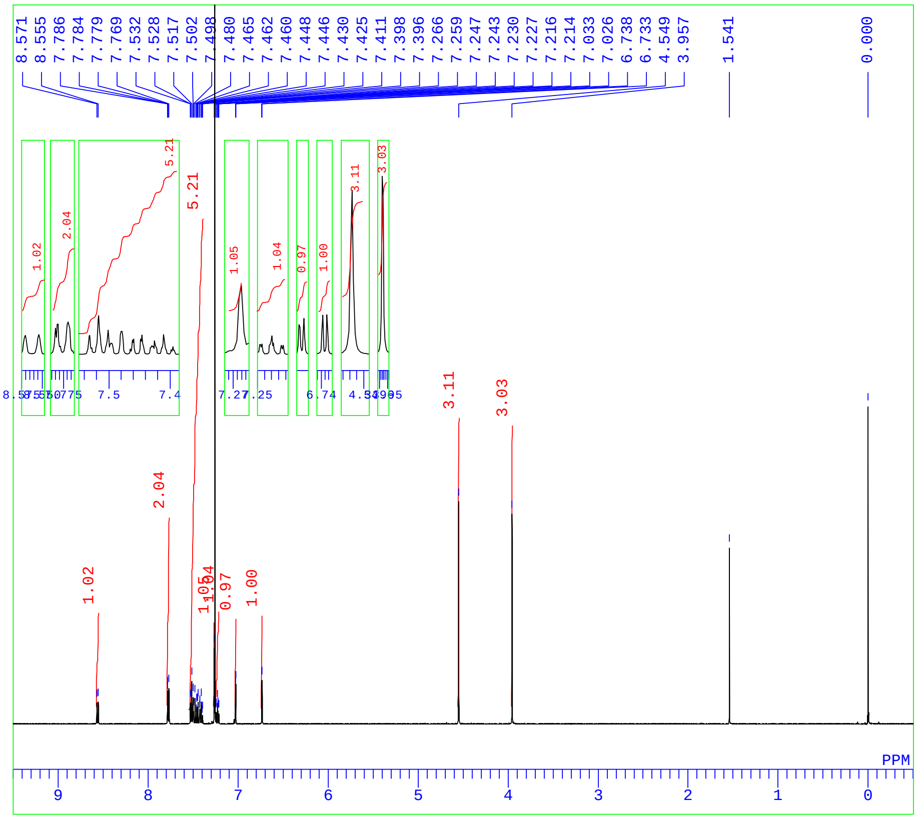

DFILE B-58 d H.als
COMNT single_pulse
DATIM 2016-04-25 21:46:32
OBNUC 1H
EXMOD single_pulse.ex2
OBFRQ 500.16 MHz
OBSET 2.41 KHz
OBFIN 6.01 Hz
POINT 13107
FREQU 7507.39 Hz
SCANS 8
ACQTM 1.7459 sec
PD 5.0000 sec
PW1 6.82 usec
IRNUC 1H
CTEMP 21.2 c
SLVNT CDCL3
EXREF 0.00 ppm
BF 0.12 Hz
RGAIN 54

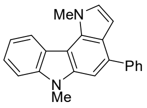

7aF-Me$_2$

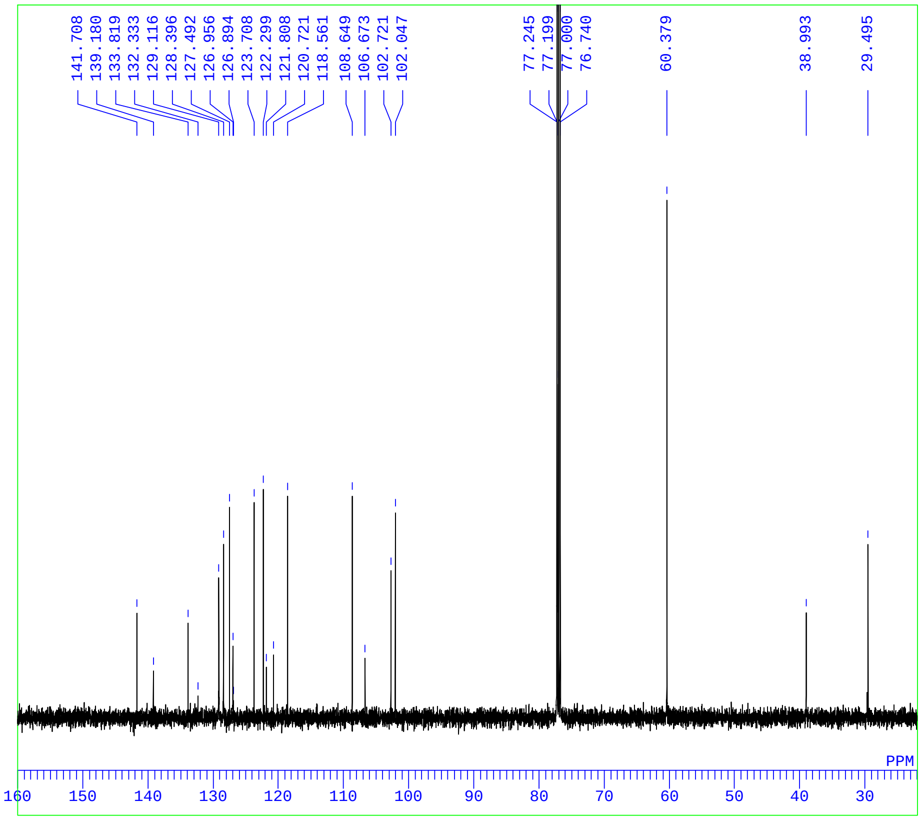

DFILE 7aF-Me2Cc.als
COMNT
DATIM 2018-06-05 17:29:35
OBNUC 13C
EXMOD single_pulse_dec
OBFRQ 125.77 MHz
OBSET 7.87 KHz
OBFIN 4.21 Hz
POINT 16384
FREQU 31565.66 Hz
SCANS 1849
ACQTM 0.5190 sec
PD 1.0000 sec
PW1 3.67 usec
IRNUC 1H
CTEMP 21.2 c
SLVNT CDCL3
EXREF 77.00 ppm
BF 0.12 Hz
RGAIN 58

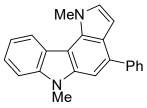

7aF-Me$_2$

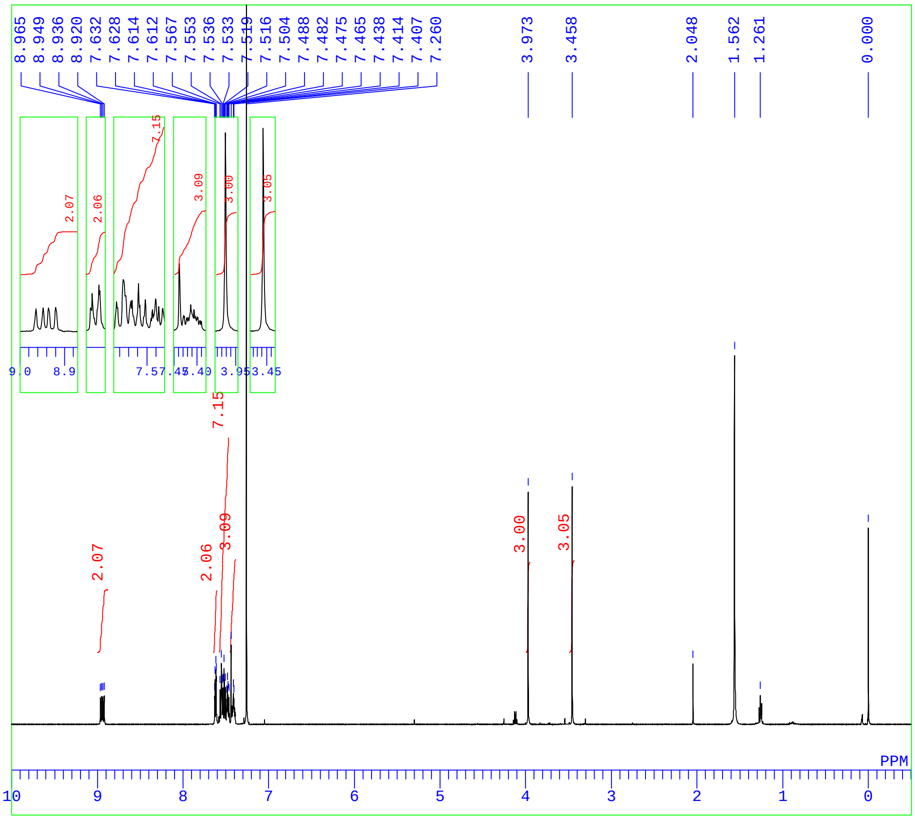

DFILE B190.als
COMNT single_pulse
DATIM 2018-05-23 18:36:49
OBNUC 1H
EXMOD single_pulse.ex2
OBFRQ 500.16 MHz
OBSET 2.41 KHz
OBFIN 6.01 Hz
POINT 13107
FREQU 7507.39 Hz
SCANS 8
ACQTM 1.7459 sec
PD 5.0000 sec
PW1 7.15 usec
IRNUC 1H
CTEMP 20.9 c
SLVNT CDCL3
EXREF 0.00 ppm
BF 0.12 Hz
RGAIN 50

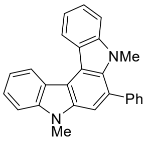

11A-Me$_2$

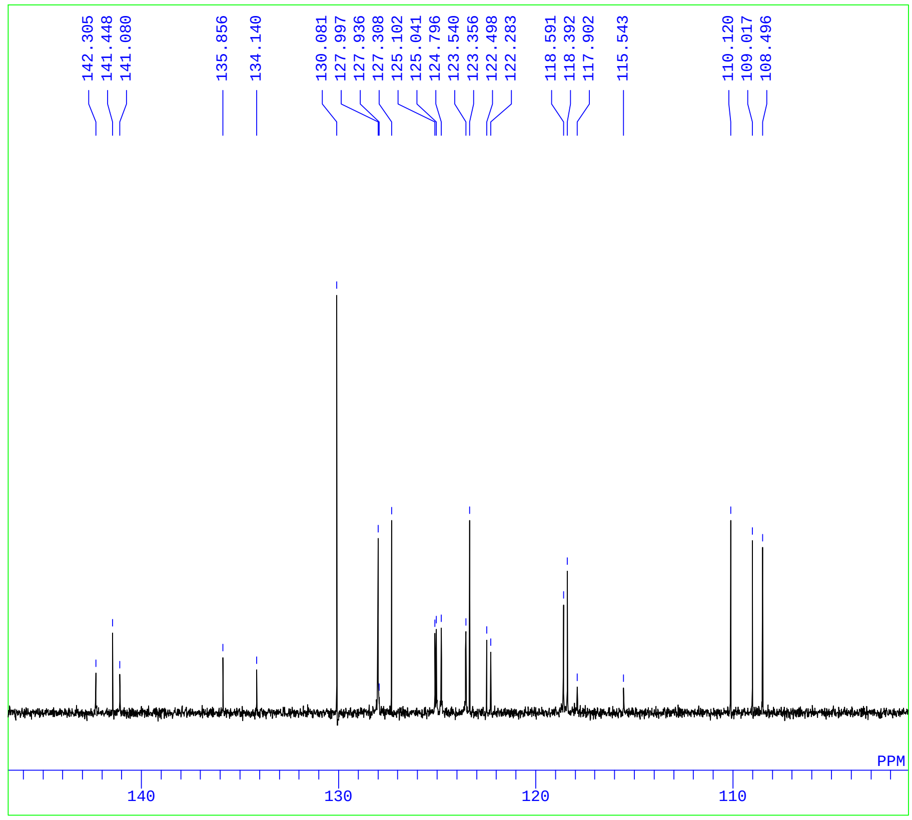

DFILE B190 C.als
COMNT
DATIM 2018-05-23 11:22:42
OBNUC 13C
EXMOD single_pulse_dec
OBFRQ 125.77 MHz
OBSET 7.87 KHz
OBFIN 4.21 Hz
POINT 16384
FREQU 31565.66 Hz
SCANS 1747
ACQTM 0.5190 sec
PD 1.0000 sec
PW1 3.67 usec
IRNUC 1H
CTEMP 21.9 c
SLVNT CDCL3
EXREF 77.00 ppm
BF 0.12 Hz
RGAIN 60

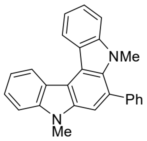

11A-Me$_2$
